# Isothiourea‐Catalysed Acylative Kinetic Resolution of Aryl–Alkenyl (sp^2^ vs. sp^2^) Substituted Secondary Alcohols

**DOI:** 10.1002/chem.201604788

**Published:** 2016-11-30

**Authors:** Stefania F. Musolino, O. Stephen Ojo, Nicholas J. Westwood, James E. Taylor, Andrew D. Smith

**Affiliations:** ^1^ EaStCHEM School of Chemistry University of St Andrews, North Haugh St Andrews KY16 9ST UK

**Keywords:** acylation, isothiourea, kinetic resolution, organocatalysis, renewable resources

## Abstract

The non‐enzymatic acylative kinetic resolution of challenging aryl–alkenyl (sp^2^ vs. sp^2^) substituted secondary alcohols is described, with effective enantiodiscrimination achieved using the isothiourea organocatalyst HyperBTM (1 mol %) and isobutyric anhydride. The kinetic resolution of a wide range of aryl–alkenyl substituted alcohols has been evaluated, with either electron‐rich or naphthyl aryl substituents in combination with an unsubstituted vinyl substituent providing the highest selectivity (*S*=2–1980). The use of this protocol for the gram‐scale (2.5 g) kinetic resolution of a model aryl–vinyl (sp^2^ vs. sp^2^) substituted secondary alcohol is demonstrated, giving access to >1 g of each of the product enantiomers both in 99:1 *e.r*.

## Introduction

**Figure 1:**
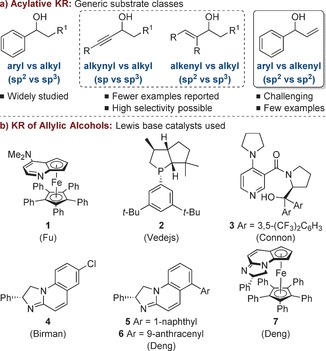
Lewis base‐catalysed KR of secondary alcohols.

Non‐enzymatic, acylative kinetic resolution (KR) is a powerful method for the preparation of enantiomerically enriched alcohols.^1^ In this regard, enantioselective Lewis base‐catalysed acylations are one of the most widely employed methodologies, and various catalyst structures and acyl transfer agents have been developed. In terms of substrate scope, non‐enzymatic acylative KRs are most commonly trialed on benzylic secondary alcohols for which the catalytic acylating agent must differentiate between the enantiomers of alcohols bearing a planar aryl (sp^2^) and a tetrahedral alkyl (sp^3^) substituent in order to obtain high selectivity (Figure 1 a).

Although less common, highly selective methods have also been developed for the KR of both alkynyl–alkyl (sp vs. sp^3^) and alkenyl–alkyl (sp^2^ vs. sp^3^) substituted secondary alcohols. In these systems the acylating agent must differentiate between the enantiomers of alcohols with a planar π‐system and a tetrahedral sp^3^ hybridized substituent. For example, a number of Lewis base organocatalysts have been utilized for the acylative KR of alkenyl–alkyl (sp^2^ vs. sp^3^) allylic alcohols (Figure 1 b).^2^, ^3^, ^4^, ^5^, ^6^, ^7^ Fu used planar‐chiral DMAP‐derived ferrocene catalyst **1** and acetic anhydride for the KR of a range of allylic alcohols, including two that had served as intermediates in natural product synthesis, with high selectivity factors, *S* (up to 80).^2^ Vedejs has also achieved high selectivity for the KR of allylic alcohols using chiral phosphine **2** and isobutyric anhydride (*S* up to 82).^3^ More recently, both Birman^4^ and Deng^5^ have used amidine catalysts **4**–**6** for the acylative KR of alkenyl–alkyl (sp^2^ vs. sp^3^) alcohols with moderate to good selectivity obtained across a range of substrates.

**Scheme 1:**
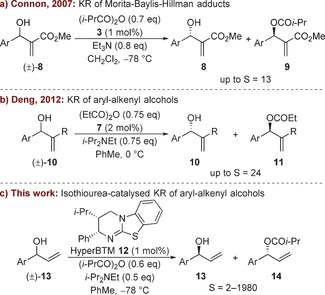
Lewis base‐catalysed acylative KR of aryl–alkenyl alcohols.

To date there are very few examples of the KR of secondary allylic alcohols bearing both planar alkenyl and planar aryl substituents (sp^2^ vs. sp^2^).^8^ This is likely to be due to the challenge of the catalytic acylating agent differentiating between enantiomeric alcohols with two planar sp^2^ hybridized substituents during the selectivity‐determining acylation step. To this end, Connon and co‐workers have studied the KR of a range of Morita–Baylis–Hillman (MBH) adducts **8** bearing aryl substituents, obtaining moderate selectivity (*S* up to 13) using chiral DMAP derivative **3** and isobutyric anhydride (Scheme 1 a).^9^ Mandai and Suga have also reported a single example of the KR of an aryl MBH adduct using a chiral phosphoric acid catalyst alongside acetyl chloride and DABCO (1,4‐diazabicyclo[2.2.2]octane).^10^ Deng and co‐workers have used amidine **7** as a catalyst for the acylative KR of aryl–alkenyl substituted alcohols **10**, with moderate to good selectivity (*S* up to 24) obtained for a range of aryl substituents and simple 1,1‐disubstituted alkenes (Scheme 1 b).^11^

Herein, the challenge of resolving aryl–alkenyl (sp^2^ vs. sp^2^) substituted secondary alcohols is addressed using an isothiourea‐based organocatalyst (Scheme 1 c).^12^, ^13^ Isothioureas have previously been used as catalysts for the acylative KR of various secondary alcohols,^14^ as well as the desymmetrization of *meso*‐diols.^15^ In this report, we demonstrate that the isothiourea HyperBTM **12** can differentiate between the enantiomers of aryl–alkenyl (sp^2^ vs. sp^2^) substituted secondary alcohols. The selectivity of the KR has been assessed across a wide range of allylic alcohols, with good to excellent enantiodiscrimination observed for substrates bearing either electron‐rich or naphthyl substituents alongside an unsubstituted vinyl substituent.

## Results and Discussion

The reaction of (±)‐1‐(4‐methoxyphenyl)prop‐2‐en‐1‐ol **15** with propanoic anhydride (0.5 equiv) and *i*‐Pr_2_NEt (0.5 equiv) in CHCl_3_ was chosen as the starting point to identify suitable reaction conditions for the acylative KR of aryl–alkenyl (sp^2^ vs. sp^2^) substituted alcohols. The commercially available and readily prepared isothiourea HyperBTM **12** (1 mol %) was identified as the most promising in an initial screen of readily available catalysts, giving 44 % conversion into ester **16** with *S*=8,^16^, ^17^, ^18^ whereas both tetramisole **17** and BTM **18** gave poor conversion and lower selectivity (Table 1, entries 1–3). The absolute configuration of the major enantiomer of recovered alcohol (*S*)‐**15** was confirmed by comparison of its specific rotation with literature values.^19^ Further optimization revealed that using isobutyric anhydride and lowering the reaction temperature to −40 °C gave improved selectivity (Table 1, entry 4). A solvent screen showed that both THF (*S*=16) and in particular toluene (*S*=21) gave improvements in selectivity (Table 1, entries 5 and 6). Further lowering the reaction temperature to −78 °C led to the efficient KR of (±)‐**15** with excellent selectivity (*S*=29) considering the challenging aryl–alkenyl (sp^2^ vs. sp^2^) alcohol substitution (Table 1, entry 7). The catalyst loading could also be lowered to 0.25 mol % without an appreciable drop in either conversion or selectivity (Table 1, entry 8), although for practicality 1 mol % HyperBTM **12** was used to assess the reaction scope.

**Table 1:** Reaction optimization.

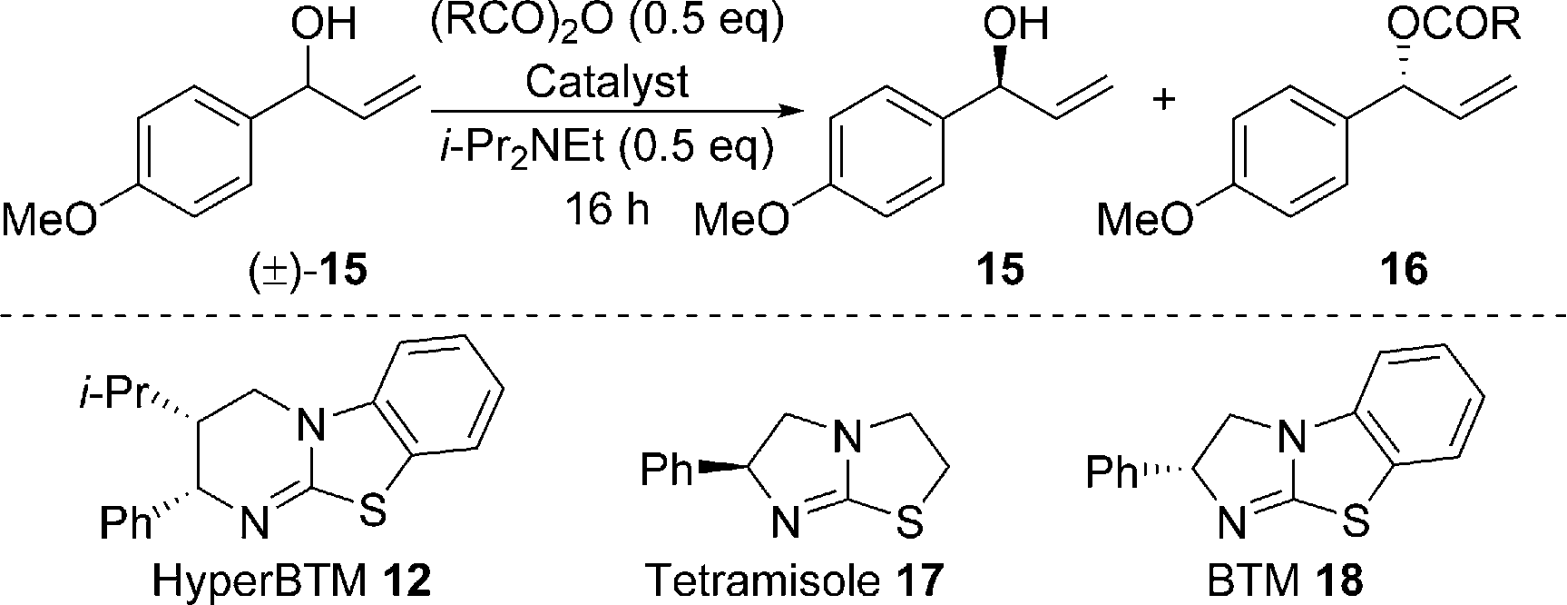								
Entry	Cat. (mol %)	R	Solvent	*T* [°C]	Conv. [%]^[a]^	**15** *e.r*.^[b]^	**16** *e.r*.^[b]^	*S* ^[c]^
1	**12** (1)	Et	CHCl_3_	0	44	76:24	83:17	8
2	**17** (1)	Et	CHCl_3_	0	6	52:48 (*ent*)	68:32 (*ent*)	2
3	**18** (1)	Et	CHCl_3_	0	21	52:48	58:42	2
4^[d]^	**12** (1)	*i*‐Pr	CHCl_3_	−40	52	88:12	86:14	14
5^[d]^	**12** (1)	*i*‐Pr	THF	−40	51	90:10	87:13	16
6^[d]^	**12** (1)	*i*‐Pr	PhMe	−40	50	90:10	90:10	21
7	**12** (1)	*i*‐Pr	PhMe	−78	50	92:8	92:8	29
8	**12** (0.25)	*i*‐Pr	PhMe	−78	53	94:6	89:11	22

[a] Calculated by HPLC analysis. [b] *e.r*. determined by HPLC analysis. [c] Calculated using the equations developed by Kagan.^17^ [d] 0.6 equiv of anhydride used.

The optimized conditions for the KR of (±)‐**15** were then tested for a range of vinyl alcohols bearing various aryl substituents (Tables 2, 3, and 4). Initial investigations probed the effect of varying the steric and electronic nature of the aryl group bearing a single substituent in either the *para*‐, *meta*‐, or *ortho*‐position (Table 2). Unsubstituted and aryl rings bearing electron‐donating methoxy substituents in either the *para*‐, *meta*‐, or *ortho*‐positions worked well, with excellent selectivity obtained in all cases (Table 2, entries 1, 2, 6 and 9, *S*=29–59). In contrast, the presence of an electron‐withdrawing CF_3_ substituent in any of the positions around the aryl ring led to a noticeable drop in selectivity (Table 2, entries 3, 7 and 10, *S*=7–11). For example, although 3‐methoxy substituted alcohol (±)‐**23** gave *S*=59, the analogous 3‐CF_3_ substituted (±)‐**24** gave *S*=11. Various halogen substituents were tolerated, allowing KR of alcohols **21**, **22** and **25** with moderate levels of selectivity (Table 2, entries 4, 5 and 8, *S*=8–17). This observation is consistent with previous proposals for the acylative KR of aryl–alkyl (sp^2^ vs. sp^3^) substituted secondary alcohols using isothioureas, which typically give higher selectivity in the resolution of alcohols bearing electron‐rich aryl substitutents.^14^ In these processes, the aryl unit is thought to be the key recognition motif for enantiodiscrimination, being involved in π‐stacking with an electron‐deficient acyl ammonium intermediate during the acylation step.

**Table 2:** KR of substituted aryl–vinyl (sp^2^ vs. sp^2^) secondary alcohols.

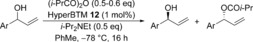					
Entry	Substrate	Conv. [%]^[a]^	Alcohol *e.r*.^[b]^ (yield, %)	Ester *e.r*.^[b]^ (yield, %)	*S* ^[c]^
1	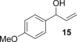	50	92:8 (40)	92:8 (34)	29
2	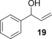	41	82:18 (48)	95:5 (35)	35
3	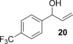	52	83:17 (37)	82:18 (41)	8
4	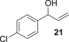	48^[d]^	87:13 (31)	91:9 (30)	17
5	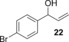	35	68:32 (56)	84:16 (28)	8
6	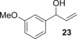	43	86:14 (46)	96:4 (40)	59
7	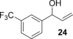	50	86:14 (46)	88:12 (50)	15
8	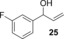	54^[d]^	89:11 (33)	84:16 (34)	12
9	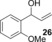	52^[d]^	95:5 (44)	N/D^[e]^ (35)	36
10	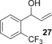	37	68:32 (59)	82:18 (30)	7

[a] Calculated by HPLC analysis. [b] *e.r*. determined by HPLC analysis. [c] Calculated using the equations developed by Kagan.^17^ [d] Conversion determined by ^1^H NMR analysis. [e] Enantiomers of ester inseparable by HPLC.

**Table 3:** KR of poly‐substituted aryl–vinyl (sp^2^ vs. sp^2^) secondary alcohols.

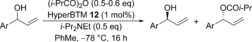					
Entry	Substrate	Conv. [%]^[a]^	Alc. *e.r*.^[b]^ (yield, %)	Ester *e.r*.^[b]^ (yield, %)	*S* ^[c]^
1^[d]^	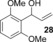	37	78:22 (40)	99:1 (26)	110
2	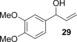	60	>99:1 (39)	80:20 (50)	44
3	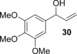	51	94:6 (43)	92:8 (47)	33
4	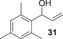	22	61:39 (51)	90:10 (17)	11
5	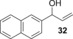	49	97:3 (47)	>99:1 (45)	1980^[e]^
6	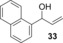	46	92:8 (41)	98:2 (31)	108
7	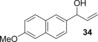	47	72:28 (31)	75:25 (37)	5
8	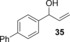	42	78:22 (50)	88:12 (37)	13
9	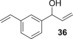	48	89:11 (34)	92:8 (29)	26

[a] Calculated by HPLC analysis. [b] *e.r*. determined by HPLC analysis. [c] Calculated using the equations developed by Kagan.^17^ [d] 48 h reaction time. [e] Determined by linear regression analysis (see text).

Subsequent studies aimed to exploit this observation through testing the KR of aryl–vinyl alcohols bearing either poly‐substituted electron‐rich aryl‐substitutents or extended aromatic naphthyl units (Table 3). Excellent selectivity was observed with electron‐rich 2,6‐dimethoxy substituted aryl–alkenyl alcohol (±)‐**28** (*S*=110), although the presence of two *ortho*‐substituents resulted in lower, but still acceptable, conversion over an extended 48 h reaction time due to the slower rate of acylation (Table 3, entry 1). The methodology was then applied to the KR of lignin‐derived alcohols (±)‐**29** and (±)‐**30** bearing methoxy‐substituted aryl rings (Table 3, entries 2 and 3). Pleasingly, the resolutions proceeded with excellent selectivity in both cases (*S*=44 and 33, respectively), allowing the recovered alcohols **29** and **30** to be isolated with high *e.r*. This demonstrates that the methodology can be used to access enantiomerically pure synthetic building blocks from renewable monomers derived from lignin, which is important for the continued drive for valorization of such feedstocks.^18^ Mesityl‐substituted allylic alcohol (±)‐**31** also gave lower conversion into the corresponding ester, but the KR selectivity was reasonable (Table 3, entry 4, *S*=11). The KR of 2‐naphthyl substituted vinyl alcohol (±)‐**32** gave exceptional selectivity, with the remaining alcohol **32** (97:3 *e.r*.) and the corresponding isobutyric ester (>99:1 *e.r*.) isolated with excellent *e.r*. at 50 % conversion (Table 3, entry 5). The presence of a 1‐naphthyl substituent also led to excellent selectivity (*S*=108) under the standard conditions (Table 3, entry 6). The selectivity observed with naphthyl substituents was surprisingly sensitive to further substitution on the naphthylene ring. For example, 6‐methoxy substituted naphthyl alcohol (±)‐**34** gave dramatically lower selectivity (*S*=5) compared with the unsubstituted analogue (Table 3, entry 7). To probe the origin of the high selectivity using unsubstituted naphthyl alcohols, the KR protocol was tested on aryl substrates (±)‐**35** and (±)‐**36** containing 4‐phenyl and 3‐vinyl substituents, respectively (Table 3, entries 8 and 9). In both cases the KR gave good selectivity (*S*=13 and 26), although neither match the levels of enantiodiscrimination observed with the extended conjugation within the unsubstituted naphthyl examples.

**Figure 2:**
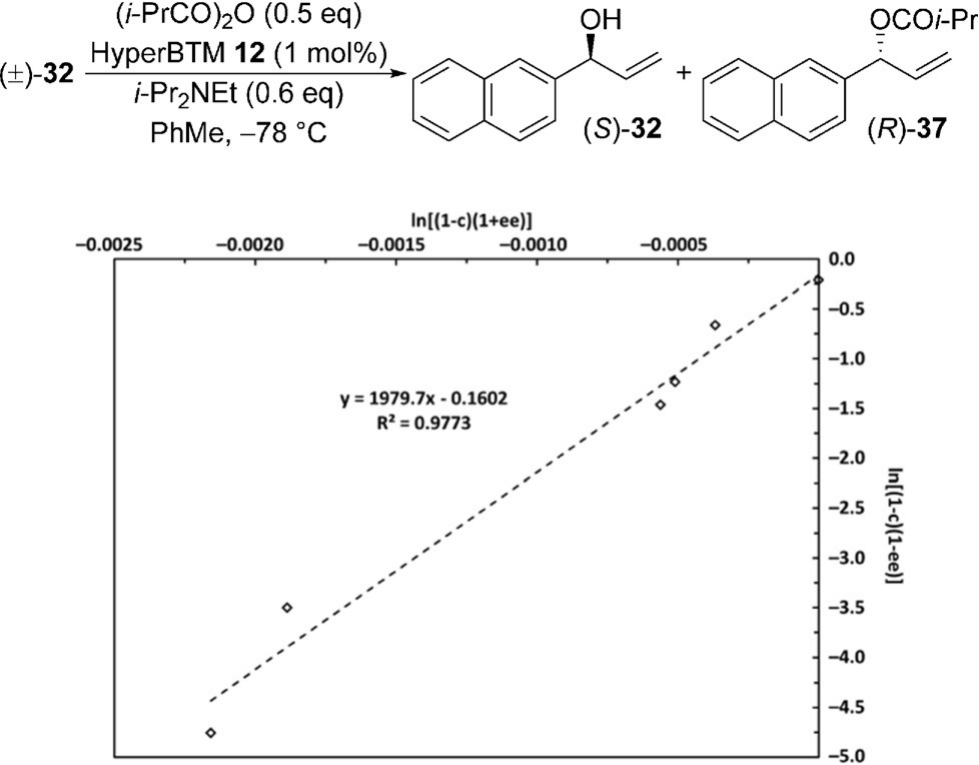
Determination of the selectivity factor for the KR of (±)‐**32** using linear regression.

For the resolution of (±)‐**32**, the exceptionally high selectivity, coupled with the accuracy of the HPLC analysis used to measure the *e.r*. values of both alcohol and ester, makes the calculation of an exact selectivity factor difficult. To validate the reported *S* value, repeat experiments were performed and product enantioselectivities measured at varying reaction conversions. The data obtained was plotted as shown in Figure 2, allowing the selectivity factor to be determined using linear regression.^19^ Good linear correlation of the data over a range of reaction conversions suggests that *S*=1980 for the KR of (±)‐**32**.

Next, the use of heteroaryl–vinyl (sp^2^ vs. sp^2^) secondary alcohols in the KR was briefly assessed. Both 2‐ and 3‐pyridyl substituted alcohols (±)‐**38** and (±)‐**39** gave poor selectivity (Table 4, entries 2 and 3, *S*=3 and 4, respectively), whereas 2‐thiophenyl alcohol (±)‐**40** gave better, but still moderate, results (Table 4, entry 3, *S*=9).

**Table 4:** KR of heteroaryl–vinyl (sp^2^ vs. sp^2^) secondary alcohols.

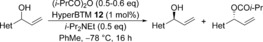					
Entry	Substrate	Conv. [%]^[a]^	Alcohol *e.r*.^[b]^ (yield, %)	Ester *e.r*.^[b]^ (yield, %)	S^[c]^
1	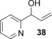	49	67:33 (42)	68:32 (45)	3
2	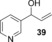	46	69:31 (46)	73:27 (39)	4
3	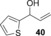	44	76:24 (49)	84:16 (44)	9

[a] Calculated by HPLC analysis. [b] *e.r*. determined by HPLC analysis. [c] Calculated using the equations developed by Kagan.^17^

The effect of substitution on the alkene portion was then explored under the standard conditions (Table 5). The KR of 1,1‐disubstituted alkene (±)‐**41** showed good reactivity and reasonable selectivity (Table 5, entry 1), although the selectivity was lower (*S*=10) than for the corresponding vinyl analogue (±)‐**15** (*S*=29). The reaction with 1,2‐disubstituted alkene (±)‐**42** did not proceed at −78 °C and gave a complex mixture of products when performed at 0 °C. However, the recovered alcohol and ester were both obtained in low *e.r*. so the selectivity is likely to be minimal (Table 5, entry 2). The use of 1,1,2‐trisubstituted alkene (±)‐**43** also gave low levels of selectivity (Table 5, entry 3, *S*=3). As the 2‐naphthyl aryl substituent led to extremely high levels of enantiodiscrimination with unsubstituted allylic alcohol (±)‐**32**, the effect of alkene substitution within this series was also investigated. In this case, 1,1‐disubstituted alkene (±)‐**44** gave higher selectivity (*S*=24, Table 5, entry 4) compared with (±)‐**41**, although again this was significantly lower than for vinyl substituted (±)‐**32**. The reactions of 1,2‐disubstituted (±)‐**45** and 1,1,2‐trisubstituted (±)‐**46** followed the same trend as previously and both gave relatively low selectivity (Table 5, entries 5 and 6, *S*=11 and 8). These results demonstrate that levels of enantiodiscrimination between the two enantiomers of aryl–alkenyl (sp^2^ vs. sp^2^) secondary alcohols decreases with increasing substitution on the alkenyl moiety.

**Table 5:** Effect of alkene substitution.

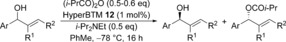					
Entry	Substrate	Conv. [%]^[a]^	Alcohol *e.r*.^[b]^ (yield, %)	Ester *e.r*.^[b]^ (yield, %)	S^[c]^
1	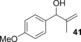	45	79:21 (48)	84:16 (42)	10
2^[d]^	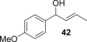	57	73:27 (42)	65:35 (33)	N/D
3	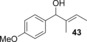	38	62:38 (64)	70:30 (34)	3
4	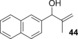	47	86:14 (45)	92:8 (37)	24
5	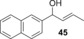	47	81:19 (51)	85:15 (38)	11
6	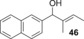	53	84:16 (45)	80:20 (48)	8

[a] Calculated by HPLC analysis. [b] *e.r*. determined by chiral HPLC analysis. [c] Calculated using the equations developed by Kagan.^17^ [d] Reaction performed at 0 °C.

Finally, as the catalytic system can effectively discriminate between the two planar sp^2^ hybridized substituents within aryl–alkenyl alcohols, the KR of some alternative classes of secondary alcohol were compared under the same reaction conditions (Table 6). Interestingly, the KR of aryl–vinyl substituted alcohol (±)‐**32** (sp^2^ vs. sp^2^) gave higher levels of enantiodiscrimination than the analogous aryl–alkyl substituted alcohol (±)‐**47** (sp^2^ vs. sp^3^), although in both cases the selectivity is excellent (Table 6, entries 1 and 2). However, the use of aryl–alkynyl alcohol (±)‐**48** (sp^2^ vs. sp) gave poor selectivity (*S*=3) in the KR process (Table 6, entry 3). The catalytic system was also only poorly selective for the KR of vinyl‐alkyl alcohol (±)‐**49** (sp^2^ vs. sp^3^) (Table 6, entry 4, *S*=3). This suggests that both aryl (sp^2^) and alkynyl (sp) groups are effective recognition motifs for enantiodiscrimination and may interact with the proposed acyl ammonium intermediate (vide infra) during the acylation step. Conversely, vinyl (sp^2^) and alkyl (sp^3^) substituents are poor recognition units and are unlikely to interact with the catalytic intermediate. Consequently, combining an effective recognition motif (such as aryl (sp^2^) and alkynyl (sp) groups) with a poor one (such as vinyl (sp^2^) and alkyl (sp^3^) units) leads to high enantiodiscrimination during KR, whereas alternative combinations result in low selectivity.

**Table 6:** KR of different classes of secondary alcohols.

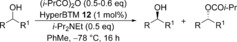					
Entry	Substrate	Conv. [%]^[a]^	Alcohol *e.r*.^[b]^ (yield, %)	Ester *e.r*.^[b]^ (yield, %)	*S* ^[c]^
1	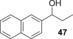	52	>99:1 (37)	96:4 (39)	152
2	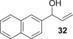	49	97:3 (47)	>99:1 (45)	1980^[d]^
3	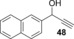	53	66:34 (35)	64:36 (36)	3
4	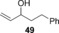	40	61:39 (35)	68:32 (25)	3

[a] Calculated by HPLC analysis. [b] *e.r*. determined by chiral HPLC analysis. [c] Calculated using the equations developed by Kagan.^17^ [d] Determined by linear regression analysis (see text).

**Scheme 2:**
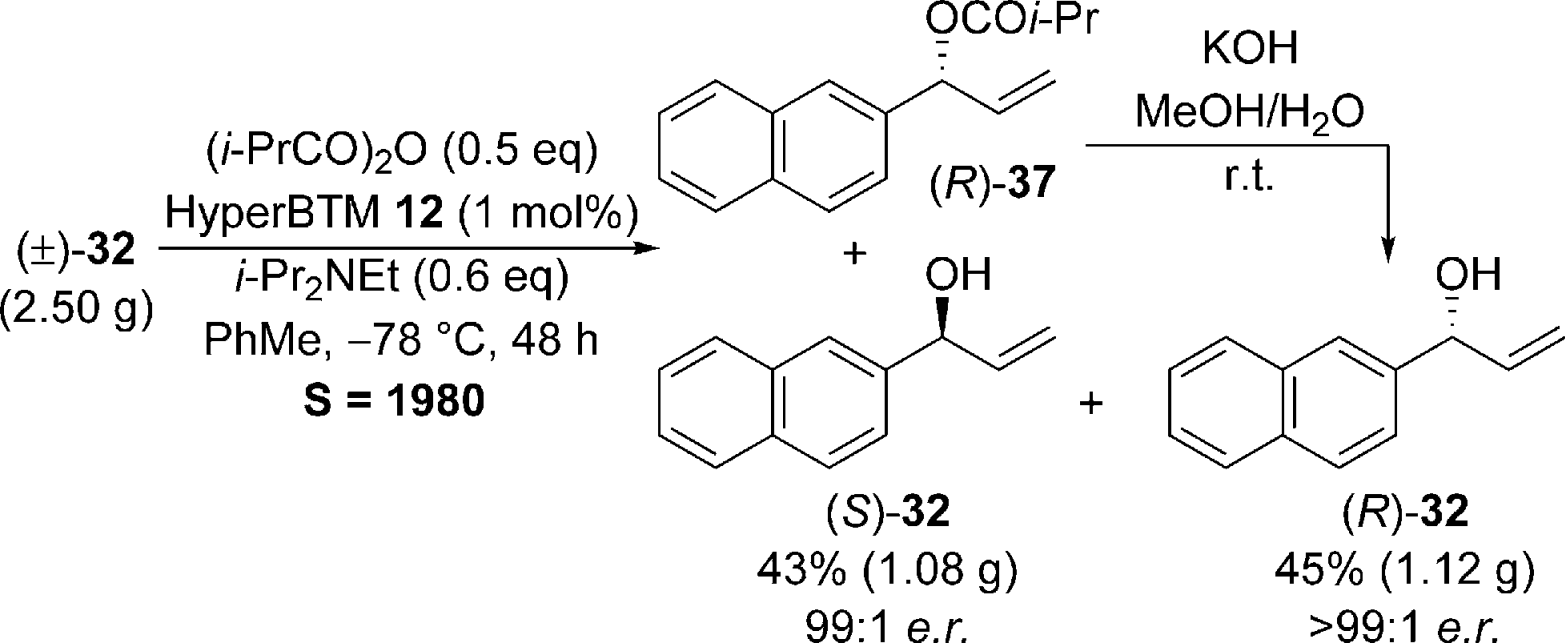
Preparative‐scale KR for the separation of (±)‐**32**.

To demonstrate the synthetic utility of this KR process to facilitate the separation of the two enantiomers of a racemic alcohol, the KR was performed on a preparative laboratory scale using 2.5 g (13.6 mmol) of (±)‐**32** and 1 mol % of HyperBTM (Scheme 2). This highly selective reaction proceeded to 50 % conversion, allowing unreacted (*S*)‐**32** to be recovered in 43 % yield (1.08 g) and 99:1 *e.r*. Isolated ester (*R*)‐**37** was readily hydrolyzed under basic conditions to give (*R*)‐**32** in 45 % yield (1.12 g) over the two steps and >99:1 *e.r*.

**Scheme 3:**
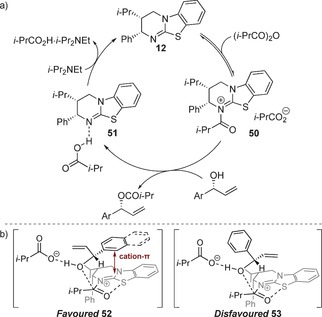
a) Proposed mechanism. b) Stereochemical rationale.

The proposed catalytic cycle starts with a reversible acylation of HyperBTM **12** with isobutyric anhydride to form acyl ammonium intermediate **50** (Scheme 3 a). Turnover‐limiting acylation of the favoured enantiomer of the aryl–alkenyl alcohol is thought to occur with concomitant proton transfer to the carboxylate anion.^20^, ^21^ The *i*‐Pr_2_NEt may possibly act as a shuttle base to regenerate the catalyst and remove isobutyric acid. The sense of enantioselectivity observed can be rationalized by considering the interactions of the incoming alcohol with acyl ammonium **50** during the selectivity‐determining step (Scheme 3 b). Acyl ammonium **50** is thought to be conformationally locked due to a stabilizing non‐bonding O−S interaction (n_O_ to σ*_C−S_),^22^ with the *Re* face blocked by the pseudo‐axial phenyl group. The fast‐reacting enantiomer of the aryl–alkenyl alcohol can adopt a conformation that has a potentially stabilizing aryl π‐cation interaction with the isothiourea (**52**), which is favoured over the potential alkenyl π‐cation interaction in the slow reacting enantiomer (**53**).^23^ This model is consistent with the higher selectivity observed for substrates bearing electron‐rich aryl rings due to the increased strength of the proposed cation‐π interaction in the favoured transition state in these cases.^24^ Conversely, increasing the substitution on the alkene makes this π‐system more electron rich, which decreases the difference in energy between the diastereomeric transition states and accounts for the lower selectivity obtained for these examples. A possible explanation for the enhanced selectivity with naphthyl substituents is the presence of an additional stacking interaction with the benzenoid ring of acyl ammonium **50** for the fast reacting enantiomer. Substitution of the naphthyl ring with electron‐donating substituents may destabilise these additional interactions,^25^ resulting in the observed loss in enantiodiscrimination.

## Conclusion

The isothiourea HyperBTM **12** (1 mol %) can catalyze the acylative KR of a range of aryl–alkenyl (sp^2^ vs. sp^2^) substituted secondary alcohols with isobutyric anhydride. The catalytic system achieves effective enantiodiscrimination between the enantiomers of secondary alcohols bearing two planar sp^2^ hybridized substituents. The efficiency of the KR process has been assessed for a range of substituted aryl and heteroaryl moieties and various alkene substitution patterns. The highest selectivity is obtained when either electron‐rich or naphthyl aryl substituents are present in combination with a vinyl substituent. Conversely, the presence of either electron‐deficient aryl rings or substituted alkenes leads to lower levels of selectivity. The optimized KR process can be used to separate the two enantiomers of synthetically useful aryl–vinyl alcohols with high enantioselectivity (up to >99:1 *e.r*.) on a preparative scale at low catalyst loading (1 mol %). Ongoing work within this laboratory is focused upon the development of practical KR processes of challenging substrates and their applications in synthesis.

## Experimental Section

**General**: For general experimental details, full characterisation data, ^1^H and ^13^C{^1^H} NMR spectra, and HPLC traces, see the Supporting Information.^26^

### Representative procedure for the KR of aryl–alkenyl alcohols

The appropriate alcohol (1 equiv) was dissolved in PhMe (0.35 m) and the solution cooled to −78 °C. HyperBTM **12** (1 mol %), *i*‐Pr_2_NEt (0.6 equiv) and isobutyric anhydride (0.5 equiv) were added and the solution stirred at −78 °C for 16 h. The reaction was quenched with 1 m HCl, the solution diluted with EtOAc and washed successively with 1 m HCl (×2), NaHCO_3_ (×2) and brine. The organic layer was dried over anhydrous Na_2_SO_4_, filtered and concentrated under reduced pressure. The alcohol and ester were purified by column chromatography and analysed by chiral HPLC.

## Supporting information

As a service to our authors and readers, this journal provides supporting information supplied by the authors. Such materials are peer reviewed and may be re‐organized for online delivery, but are not copy‐edited or typeset. Technical support issues arising from supporting information (other than missing files) should be addressed to the authors.

Supplementary
