# Supplementary material for: Isothiourea‐Catalysed Acylative Kinetic Resolution of Aryl–Alkenyl (sp2 vs. sp2) Substituted Secondary Alcohols
Source: Chemistry. 2016 Nov 30;22(52):18916–22. doi: 10.1002/chem.201604788 (PMC6680239; doi:10.1002/chem.201604788)
Supplement: Supplementary file 1 — Supplementary [file CHEM-22-18916-s001.pdf]

CHEMISTRY
A European Journal

Supporting Information

Isothiourea-Catalysed Acylative Kinetic Resolution of Aryl–Alkenyl (sp^2 vs. sp^2) Substituted Secondary Alcohols

Stefania F. Musolino, O. Stephen Ojo, Nicholas J. Westwood, James E. Taylor,* and Andrew D. Smith*[a]

chem_201604788_sm_miscellaneous_information.pdf

Isothiourea-Catalysed Acylative Kinetic Resolution of Aryl-Alkenyl (sp^2 vs sp^2) Substituted Secondary Alcohols

Stefania F. Musolino, O. Stephen Ojo, Nicholas J. Westwood, James E. Taylor*, and Andrew D. Smith*

EaStCHEM, School of Chemistry, University of St Andrews, North Haugh, St Andrews, KY16 9ST, U .K.

E-mail: ads10@st-andrews.ac.uk; jet20@st-andrews.ac.uk

General Information S2

General Procedures S4

Compound Data S5

Preparative Kinetic Resolution S52

Linear Regression Analysis S53

References S54

NMR Spectra S56

HPLC Traces S128

General Information

Reactions involving moisture sensitive reagents were carried out under inert atmosphere (nitrogen) using standard vacuum line techniques in addition to anhydrous solvents. All glassware used was flame dried and cooled under vacuum.

Solvents (THF, CH_2Cl_2, toluene and Et_2O) were obtained anhydrous and purified by an alumina column (Mbraun SPS-800). Petrol is defined as petroleum ether 40-60 °C. All other solvents and commercial reagents were used as supplied without further purification unless stated otherwise.

Room temperature (rt) refers to 20-25 °C. Temperatures of 0 °C and –78 °C were obtained using ice/water and CO_2(s)/acetone baths respectively. Temperatures of 0 °C to –78 °C for overnight reactions were obtained using an immersion cooler (HAAKE EK 90). Reflux conditions were obtained using an oil bath equipped with a contact thermometer. In vacuo refers to the use of a Büchi Rotavapor R-2000 rotary evaporator with a Vacubrand CVC2 vacuum controller or a Heidolph Laborota 4001 rotary evaporator with a vacuum controller.

Analytical thin layer chromatography was performed on pre-coated aluminium plates (Kieselgel 60 F254 silica). TLC visualization was carried out with ultraviolet light (254 nm), followed by staining with a 1% aqueous $KMnO_4$ solution. Flash column chromatography was performed on Kieselgel 60 silica in the solvent system stated.

Melting points were recorded on an Electrothermal 9100 melting point apparatus and are uncorrected.

Optical rotations were measured on a Perkin Elmer Precisely/Model-341 polarimeter operating at the sodium D line with a 100 mm path cell at rt.

HPLC analyses were obtained on a Shimadzu HPLC consisting of a DGU-20A5 degasser, LC-20AT liquid chromatography, SIL-20AHT autosampler, CMB-20A communications bus module, SPD-M20A diode array detector and a CTO-20A column oven which allowed the temperature to be set from 25-40 °C. Separation was achieved using DAICEL CHIRALCEL OD-H and OJ-H columns or DAICEL CHIRALPAK AD-H, AS-H, IA, IB, IC and ID columns. All chiral HPLC traces were compared to the authentic racemic spectrum.

Infrared spectra (vmax/cm^{-1}) were recorded on a Shimadzu IRAffinity-1 using a Pike attenuated total reflectance (ATR) accessory. Only the characteristic peaks are quoted.

^{1}H, ^{13}C and ^{19}F nuclear magnetic resonance (NMR) spectra were acquired on either a Bruker Avance 300 (300 MHz, ^{1}H, 75 MHz ^{13}C, 282 MHz ^{19}F), Bruker Avance II 400 (400 MHz, ^{1}H, 100 MHz ^{13}C, 376 MHz ^{19}F) or a Bruker Avance II 500 (500 MHz, ^{1}H, 125 MHz ^{13}C, 470 MHz ^{19}F) spectrometer at ambient temperature in the deuterated solvent stated. All chemical shifts are quoted in parts per million (ppm) relative to the residual solvent as the internal standard. All coupling constants, J, are quoted in Hz. Multiplicities are indicated by: s (singlet), d (doublet), t (triplet), q (quartet), pent (pentet), hept (heptet), oct (octet), m (multiplet), dd (doublet of doublets), ddd (doublet of doublet of doublets), dt (doublet of triplets), dq (doublet of quartets) and td (triplet of doublets). The abbreviation Ar is used to denote aromatic, Ph to denote phenyl, Bn to denote benzyl and br to denote broad.

Mass spectrometry (m/z) data were acquired by electrospray ionization (ESI), Atmopheric Solids Analysis Probe (ASAP) or nanospray ionization (NSI) the EPSRC National Mass Spectrometry Service Centre, Swansea. At the EPSRC National Mass Spectrometry Service Centre, low resolution NSI MS was carried out on a Micromass Quattro II spectrometer and high resolution NSI MS on a Thermofisher LTQ Orbitrap XL spectrometer.

General Procedures

General Procedure A: *Preparation of racemic alcohols*

The appropriate Grignard reagent (1.5 eq) was added to a solution of aldehyde (1 eq) in anhydrous THF (0.3 M) at 0 °C under a nitrogen atmosphere and the mixture stirred for 3 h. The reaction was quenched with aq. NH_4Cl and the mixture extracted with EtOAc. The organic layer was washed with brine and dried over Na_2SO_4 and concentrated under reduced pressue. The crude product was purified by silica-gel column chromatography.

General Procedure B: *Esterification of alcohols with DMAP*

A flask was charged with DMAP (10 mol%), CH_2Cl_2 (0.3 M) and the appropriate alcohol (1 eq). Once homogeneous, the appropriate anhydride (1 eq) was added and the reaction mixture was stirred at rt for 16 h. The reaction mixture was diluted with CH_2Cl_2 and washed with 1 M HCl, saturated $NaHCO_3$ and brine. The organic layer was dried over anhydrous $MgSO_4$, filtered and concentrated under reduced pressure. The product was clean without any further purification and it was analysed by chiral HPLC.

General Procedure C: *Kinetic resolution with HyperBTM **12***

The appropriate alcohol (1 eq) was dissolved in toluene (0.35 M) and the solution cooled to –78 °C. HyperBTM **12** (1 mol %, from catalyst stock solution (see below for preparation)), *i*-Pr_2NEt (0.6 eq) and isobutyric anhydride (0.5 eq) were added and the solution stirred at –78 °C for 16 h. The reaction was quenched with 1 M HCl, the solution diluted with EtOAc and washed successively with 1 M HCl (×2), $NaHCO_3$ (×2) and brine. The organic layer was dried over anhydrous Na_2SO_4, filtered and concentrated under reduced pressure. The alcohol and ester were purified by column chromatography and analysed by chiral HPLC.

Preparation of Catalyst Stock Solution

HyperBTM **12** (50 mg) and toluene (3 mL) were placed in a 5 mL volumetric flask. Once the mixture was homogeneous toluene was added until the total volume of the mixture had reached 5 mL to give a 0.032 M solution of HyperBTM **12**.

Compound Data

1-(4-Methoxyphenyl)prop-2-en-1-ol 15

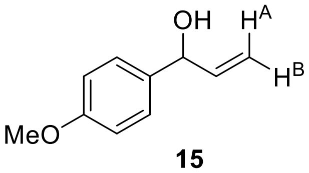

15

Following General Procedure A, vinyl magnesium bromide (78 mL of 0.7 M solution in THF, 55 mmol) added to a solution of anisaldehyde (5 g, 36.7 mmol) in anhydrous THF (100 mL) at 0 °C for 3 h gave, after purification by column chromatography (80:20 hexane : EtOAc, R_f 0.33), the title compound **15** (5 g, 83%) as a yellow oil with spectroscopic data in accordance with the literature.[1] ^{1}H NMR (500 MHz, $CDCl_3$) δ_H: 1.85 (1H, d, *J* 3.6, O*H*), 3.81 (3H, s, OC*H*3), 5.17 (1H, s, C(1)*H*), 5.19 (1H, d, *J* 10.3, C(3)H^B), 5.34 (1H, d, *J* 17.1, C(3)H^A), 6.05 (1H, ddd, *J* 16.8, 10.3, 5.9, C(2)*H*), 6.89 (2H, d, *J* 8.7, Ar(3,5)*H*), 7.30 (2H, d, *J* 8.7, Ar(2,6)*H*).

1-(4-Methoxyphenyl)allyl isobutyrate S1

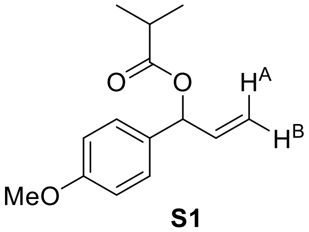

S1

Following General Procedure B, to a solution of DMAP (10 mol %), CH_2Cl_2 (0.1 M) and alcohol **15** (50 mg, 0.312 mmol) was added isobutyric anhydride (50 µL, 0.312 mmol) at room temperature and the reaction mixture was stirred for 16 h. The mixture gave a clean product (no purification) as a coulorless oil (72 mg, 98%) with spectroscopic data in accordance with the literature.[2] ^{1}H NMR (400 MHz, $CDCl_3$) δ_H: 1.16 (3H, d, *J* 7.0, CH(CH_3)), 1.19 (3H, d, *J* 7.0, CH(CH_3)), 2.59 (1H, hept, *J* 7.0, C*H*(CH_3)), 3.80 (3H, s, OCH_3), 5.22 (1H, dt, *J* 10.5, 1.3, C(3)H^B), 5.27 (1H, dt, *J* 17.2, 1.4, C(3)H^A), 6.00 (1H, ddd, *J* 17.1, 10.5, 5.6, C(2)*H*), 6.21 (1H, d, *J* 5.6, C(1)*H*), 6.83–6.92 (2H, m, Ar(3,5)*H*), 7.26–7.30 (2H, m, Ar(2,6)*H*).

Kinetic resolution of 1-(4-methoxyphenyl)prop-2-en-1-ol 15

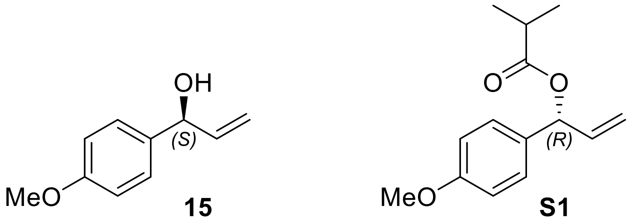

Following General Procedure C, the alcohol **15** (53 mg, 0.32 mmol), HyperBTM **12** (100 µL from stock solution, 3 µmol, 1 mol %), *i*-Pr_2NEt (28 µL, 0.16 mmol) and isobutyric anhydride (26 µL, 0.16 mmol) were reacted in toluene (0.8 mL) for 16 h to give the crude product, which was purified via column chromatography (80:20 Petrol : EtOAc, R_f 0.28 (**15**) and R_f 0.64 (**S1**)) to separate alcohol **15** (21 mg, 0.13 mmol, 40%) and ester **S1** (26 mg, 0.11 mmol, 34%). **Alcohol 15:** $[\alpha]_D^{20}$ –6.5 (*c* 0.4, $CHCl_3$) {Lit.[3] (*ent*) $[\alpha]_D^{25}$ +2.1 (*c* 0.2, $CHCl_3$)}; Chiral HPLC analysis Chiralcel OD-H (95:5 hexane : IPA, flow rate 1.0 mL min^{-1}, 211 nm, 30 °C) t_R (*R*): 11.0 min, t_R (*S*): 13.8 min, 84 % ee. **Ester S1:** $[\alpha]_D^{20}$ +27.5 (*c* 1.0, $CHCl_3$); Chiral HPLC analysis Chiralcel OJ-H (99:1 hexane : IPA, flow rate 1.0 mL min^{-1}, 211 nm, 30 °C) t_R (*R*): 8.7 min, t_R (*S*): 11.2 min, 83 % ee. S = 29.

1-Phenylprop-2-en-1-ol 19

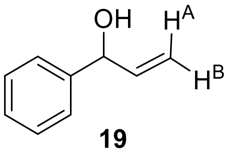

19

Following General Procedure A, phenylmagnesium bromide (1.8 mL of 3.0 M solution in Et_2O, 8.02 mmol) added to a solution of acrolein (300 mg, 5.35 mmol) in anhydrous THF (15 mL) at –78 °C for 12 h gave, after purification by column chromatography (80:20 Petrol : EtOAc, R_f 0.33), the title compound **19** (308 mg, 43%) as a yellow oil with spectroscopic data in accordance with the literature.[4] ^{1}H NMR (400 MHz, $CDCl_3$) δ_H: 1.94 (1H, s, O*H*), 5.23 (3H, dt, *J* 10.3, 1.3, C(3)H^B), 5.24 (1H, s, C(1)*H*), 5.39 (1H, dt, *J* 17.2, 1.3, C(3)H^A), 6.08 (1H, ddd, *J* 17.1, 10.3, 6.1, C(2)*H*), 7.29–7.43 (5H, m, Ph*H*).

1-Phenylallyl isobutyrate S2

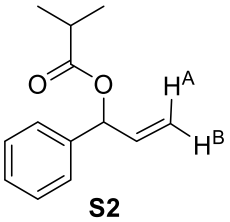

S2

Following General Procedure B, to a solution of DMAP (10 mol %), CH_2Cl_2 (0.1 M) and alcohol **19**(57 mg, 0.43 mmol) was added isobutyric anhydride (70 µL, 0.43 mmol) at room temperature and the reaction mixture was stirred for 16 h. The mixture gave a clean product (67 mg, 77%) as a colourless oil with spectroscopic data in accordance with the literature.[2] ^{1}H NMR (500 MHz, $CDCl_3$) δ_H: 1.18 (3H, d, *J* 7.0, CH(C*H*$_3$)), 1.20 (3H, d, *J* 7.0, CH(C*H*$_3$)), 2.62 (1H, hept, *J* 7.0, C*H*(CH$_3$)), 5.24 (1H, d, *J* 10.4, C(3)*H*^{B}), 5.29 (1H, d, *J* 17.1, C(3)*H*^{A}), 6.00 (1H, ddd, *J* 16.7, 10.4, 5.9, C(2)*H*), 6.25 (1H, d, *J* 5.9, C(1)*H*), 7.27–7.40 (5H, m, Ph*H*).

Kinetic resolution of 1-phenylprop-2-en-1-ol 19

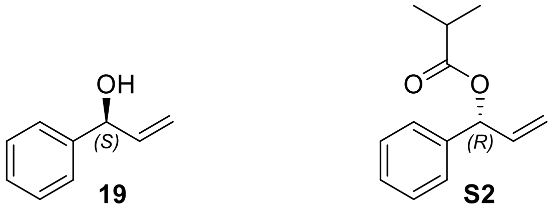

19 S2

Following General Procedure C, the alcohol **19** (65 mg, 0.48 mmol), HyperBTM **12** (151 µL from stock solution, 5 µmol, 1 mol %), *i*-Pr$_2$NEt (50 µL, 0.29 mmol) and isobutyric anhydride (39 µL, 0.24 mmol) were reacted in toluene (2 mL) for 16 h to give the crude product, which was purified via column chromatography (80:20 Petrol : EtOAc, R_f 0.33 (**19**) and R_f 0.69 (**S2**)) to separate alcohol **19** (31 mg, 0.23 mmol, 48%) and ester **S2** (35 mg, 0.17 mmol, 35%). **Alcohol 19:** $[\alpha]_D^{20}$ –2.4 (*c* 0.7, $CHCl_3$); Chiral HPLC analysis Chiralpak IB (95:5 hexane : IPA, flow rate 0.8 mL min^{-1}, 211 nm, 30 °C) t_R (*R*): 8.6 min, t_R (*S*): 9.4 min, 64% ee. **Ester S2:** $[\alpha]_D^{20}$ +49.1 (*c* 1.0, $CHCl_3$); Chiral HPLC analysis Chiralcel OJ-H (99:1 hexane : IPA, flow rate 1.0 mL min^{-1}, 220 nm, 30 °C) t_R (*R*): 5.8 min, t_R (*S*): 6.6 min, 90 % ee. S = 35.

1-(4-(Trifluoromethyl)phenyl)prop-2-en-1-ol 20

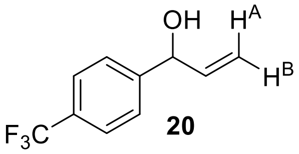

20

Following General Procedure A, vinylmagnesium bromide (4.0 mL of 0.7 M solution in THF, 2.58 mmol) added to a solution of 4-trifluoromethylbenzaldehyde (300 mg, 1.72 mmol) in

anhydrous THF (10 mL) at –78 °C for 16 h gave, after purification by column chromatography (80:20 Petrol : EtOAc, R_f 0.33), the title compound **20** (348 mg, 100%) as a yellow oil with spectroscopic data in accordance with the literature.[5] ^{1}H NMR (400 MHz, $CDCl_3$) δ_H: 2.02 (1H, br s, O*H*), 5.25 (1H, dt, *J* 10.3, 1.2, C(3)H^B), 5.28 (1H, s, C(1)*H*), 5.38 (1H, dt, *J* 17.1, 1.3, C(3)H^A), 6.02 (1H, ddd, *J* 17.1, 10.3, 6.3, C(2)*H*), 7.45–7.54 (2H, m, Ar*H*), 7.62 (2H, d, *J* 8.1, Ar*H*).

1-(4-(Trifluoromethyl)phenyl)allyl isobutyrate S3

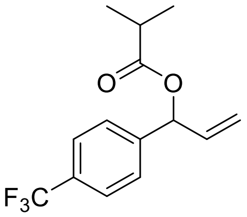

S3

Following General Procedure B, to a solution of DMAP (10 mol %), CH_2Cl_2 (0.1 M) and alcohol **20** (31 mg, 0.15 mmol) was added isobutyric anhydride (25 µL, 0.15 mmol) at room temperature and the reaction mixture was stirred for 16 h. The mixture gave a clean product (42 mg, 100%) as a yellow oil. ν_{max} (film) 1740 (C=O); ^{1}H NMR ($CDCl_3$, 400 MHz) δ 1.19 (3H, d, *J* 7.0, CH(CH_3)), 1.21 (3H, d, *J* 7.0, CH(CH_3)), 2.63 (1H, hept, *J* 7.0, C*H*(CH_3)$_2$), 5.24–5.36 (2H, m, C(3)H_2), 5.97 (1H, ddd, *J* 17.1, 10.4, 5.9, C(2)*H*), 6.28 (1H, d, *J* 5.9, C(1)*H*), 7.35–7.54 (2H, m, Ar*H*), 7.62 (2H, d, *J* 8.1, Ar*H*); ^{13}C{^{1}H} NMR (126 MHz, $CDCl_3$) δ_C: 19.0 (CH(*C*H_3)$_2$), 34.3 (*C*H(CH_3)$_2$), 75.3 (*C*(1)), 117.8 (*C*(3)), 124.1 (d, $^1J_{CF}$ 272.1, *C*F_3), 125.7 (q, $^3J_{CF}$ 3.6, Ar*C*(3,5)), 127.3 (Ar*C*(2,6)), 130.3 (q, $^2J_{CF}$ 32.4, Ar*C*(4)), 135.9 (*C*(2)), 143.2 (Ar*C*(1)), 176.0 (*C*=O); ^{19}F NMR (376 MHz, $CDCl_3$) δ_F: –62.6 ; HRMS could not be obtained due to significant fragmentation.

Kinetic resolution of 1-(4-(trifluoromethyl)phenyl)prop-2-en-1-ol 20

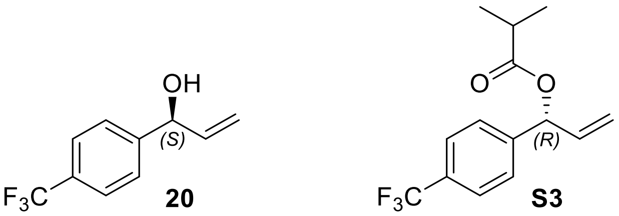

Following General Procedure C, the alcohol **20** (57 mg, 0.28 mmol), HyperBTM **12** (87 µL from stock solution, 3 µmol, 1 mol %), *i*-Pr_2NEt (25 µL, 0.14 mmol) and isobutyric anhydride (27 µL, 0.17 mmol) were reacted in toluene (1.6 mL) for 16 h to give the crude product, which was purified via column chromatography (80:20 Hexane : EtOAc, R_f 0.33

(**20**) and R$_f$ 0.69 (**S3**)) to separate alcohol **20** (19 mg, 0.09 mmol, 37%) and ester **S3** (31 mg, 0.11 mmol, 41%). **Alcohol 20:** $[\alpha]_D^{20}$ +9.6 (*c* 0.5, $CHCl_3$) {Lit.[3] (*ent*) $[\alpha]_D^{25}$ –12.9 (*c* 0.3, $CHCl_3$)}; Chiral HPLC analysis Chiralpak AD-H (99:1 hexane : IPA, flow rate 1.0 mL min^{-1}, 211 nm, 30 °C) t_R (*R*): 20.5 min, t_R (*S*): 22.5 min, 66% ee. **Ester S3:** $[\alpha]_D^{20}$ +16.0 (*c* 1.0, $CHCl_3$); Chiral HPLC analysis Chiralpak AD-H (99:1 hexane : IPA, flow rate 1.0 mL min^{-1}, 211 nm, 30 °C) t_R (*R*): 4.0 min, t_R (*S*): 5.2 min, 64% ee. S = 8.

1-(4-Chlorophenyl)prop-2-en-1-ol 21

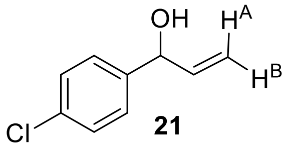

21

Following General Procedure A, vinylmagnesium bromide (5.0 mL of 0.7 M solution in THF, 3.20 mmol) added to a solution of 4-chlorobenzaldehyde (300 mg, 2.13 mmol) in anhydrous THF (10 mL) at –78 °C for 16 h gave, after purification by column chromatography (80:20 Petrol : EtOAc, R$_f$ 0.35), the title compound **21** (323 mg, 90%) as a yellow oil with spectroscopic data in accordance with the literature.[4] ^{1}H NMR (500 MHz, $CDCl_3$) δ_H: 1.94 (1H, s, O*H*), 5.19 (1H, d, *J* 6.1, C(1)*H*), 5.22 (2H, dt, *J* 10.3, 1.3, C(3)H^B), 5.35 (1H, dt, *J* 17.1, 1.3, C(3)H^A), 6.01 (1H, ddd, *J* 17.1, 10.3, 6.1, C(2)*H*), 7.30–7.35 (4H, m, Ar*H*).

1-(4-Chlorophenyl)allyl isobutyrate S4

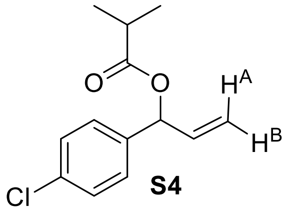

S4

Following General Procedure B, to a solution of DMAP (10 mol %), CH_2Cl_2 (0.2 M) and alcohol **21** (42 mg, 0.42 mmol) was added isobutyric anhydride (24 μL, 0.42 mmol) at room temperature and the reaction mixture was stirred for 16 h. The mixture gave a clean product (45 mg, 77%) as a yellow oil with spectroscopic data in accordance with the literature.[2] ^{1}H NMR (400 MHz, $CDCl_3$) δ_H: 1.19 (3H, d, *J* 7.0, CH(CH_3)), 1.22 (3H, d, *J* 7.0, CH(CH_3)), 2.63 (1H, hept, *J* 7.0, C*H*(CH_3)), 5.17–5.38 (2H, m, C(3)H_2), 5.99 (1H, ddd, *J* 17.1, 10.4, 5.8, C(2)*H*), 6.23 (1H, d, *J* 5.8, C(1)*H*), 7.29–7.38 (4H, m, Ar*H*).

Kinetic resolution of 1-(4-chlorophenyl)prop-2-en-1-ol 21

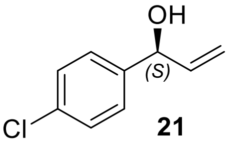

21

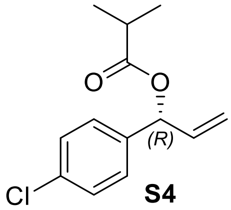

S4

Following General Procedure C, the alcohol **21** (64 mg, 0.38 mmol), HyperBTM **12** (118 µL from stock solution, 4 µmol, 1 mol %), *i*-Pr_2NEt (40 µL, 0.23 mmol) and isobutyric anhydride (31 µL, 0.19 mmol) were reacted in toluene (1 mL) for 16 h to give the crude product, which was purified via column chromatography (80:20 Petrol : EtOAc, R_f 0.35 (**21**) and R_f 0.60 (**S4**)) to separate alcohol **21** (20 mg, 0.12 mmol, 31%) and ester **S4** (27 mg, 0.11 mmol, 30%). **Alcohol 21:** $[\alpha]^{20}_{D}$ +8.0 (*c* 0.6, $CHCl_3$); 74% ee.[6] **Ester S4:** $[\alpha]^{20}_{D}$ +30.6 (*c* 1.0, $CHCl_3$); Chiral HPLC analysis Chiralcel OJ-H (95:5 hexane : IPA, flow rate 1.0 mL min^{-1}, 220 nm, 30 °C) t_R (*R*): 4.5 min, t_R (*S*): 4.9 min, 81% ee.S = 17.

1-(4-Bromophenyl)prop-2-en-1-ol 22

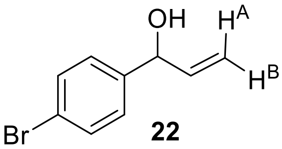

22

Following General Procedure A, vinylmagnesium bromide (4.6 mL of 0.7 M solution in THF, 3.24 mmol) added to a solution of 4-bromobenzaldehyde (400 mg, 2.16 mmol) in anhydrous THF (10 mL) at –78 °C for 16 h gave, after purification by column chromatography (70:30 Hexane : EtOAc, R_f 0.48), the title compound **22** (352 mg, 76%) as a yellow oil with spectroscopic data in accordance with the literature.[7] ^{1}H NMR (400 MHz, $CDCl_3$) δ_H: 1.95 (1H, d, *J* 3.4, O*H*), 5.14–5.21 (1H, m, C(1)*H*), 5.21 (1H, dt, *J* 10.3, 1.3, C(3)H^B), 5.35 (1H, dt, *J* 17.1, 1.3, C(3)H^A), 6.00 (1H, ddd, *J* 17.1, 10.3, 6.1, C(2)*H*), 7.21–7.30 (2H, m, Ar*H*), 7.45–7.52 (2H, m, Ar*H*).

1-(4-Bromophenyl)allyl isobutyrate S5

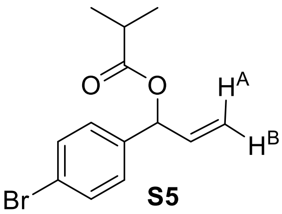

S5

Following General Procedure B, to a solution of DMAP (10 mol %), CH_2Cl_2 (0.1 M) and alcohol **21** (47 mg, 0.22 mmol) was added isobutyric anhydride (40 µL, 0.22 mmol) at room

temperature and the reaction mixture was stirred for 16 h. The mixture gave a clean product (39 mg, 74%) as a yellow oil. ν_{max} (film) 1736 (C=O); ^{1}H NMR (500 MHz, $CDCl_3$) δ_H: 1.17 (3H, d, *J* 7.0, CH(C*H*$_3$)), 1.19 (3H, d, *J* 7.0, CH(C*H*$_3$)), 2.60 (1H, hept, *J* 7.0, C*H*(CH_3)$_2$), 5.25 (1H, d, *J* 10.5, C(3)H^B), 5.28 (1H, d, *J* 16.9, C(3)H^A), 5.96 (1H, ddd, *J* 16.9, 10.5, 5.8, C(2)*H*), 6.19 (1H, d, *J* 5.8, C(1)*H*), 7.22 (2H, d, *J* 8.4, Ar*H*), 7.48 (2H, d, *J* 8.4, Ar*H*); ^{13}C{^{1}H} NMR (126 MHz, $CDCl_3$) δ_C: 19.0 (CH(*C*H$_3$)$_2$), 34.3 (*C*H(CH$_3$), 75.2 (*C*(1)), 117.3 (*C*(3)), 122.2 (Ar*C*(4)), 128.9 (Ar*C*(2,6)), 131.8 (Ar*C*(3,5)), 136.1 (*C*(2)), 138.3 (Ar*C*(1)), 176.0 (*C*=O); HRMS (FTMS + NSI$^+$) $C_{13}H_{15}BrO_2$ [M+H]$^+$ found 283.0331, requires 283.0328 (+ 1.0 ppm).

Kinetic resolution of 1-(4-bromophenyl)prop-2-en-1-ol 22

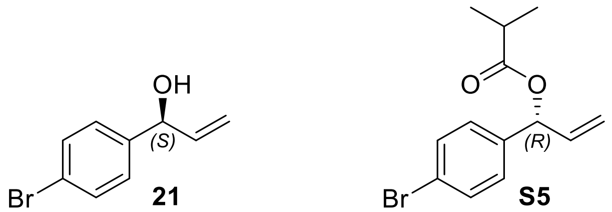

21 **S5**

Following General Procedure C, the alcohol **21** (78 mg, 0.36 mmol), HyperBTM **12** (114 µL from stock solution, 4 µmol, 1 mol %), *i*-Pr_2NEt (38 µL, 0.48 mmol) and isobutyric anhydride (30 µL, 0.18 mmol) were reacted in toluene (1.2 mL) for 16 h to give the crude product, which was purified via column chromatography (80:20 Petrol : EtOAc, R_f 0.40 (**21**) and R_f 0.60 (**S5**)) to separate alcohol **21** (43 mg, 0.20 mmol, 56%) and ester **S5** (29 mg, 0.10 mmol, 28%). **Alcohol 21:** $[\alpha]_D^{20}$ +10.0 (*c* 0.5, $CHCl_3$); Chiral HPLC analysis Chiralpak AD-H (98:2 hexane : IPA, flow rate 1.0 mL min^{-1}, 254 nm, 30 °C) t_R (*R*): 17.8 min, t_R (*S*): 18.9 min, 37% ee. **Ester S5:** $[\alpha]_D^{20}$ +30.8 (*c* 0.5, $CHCl_3$); Chiral HPLC analysis Chiralpak AD-H (99.8:0.2 hexane : IPA, flow rate 1.0 mL min^{-1}, 220 nm, 30 °C) t_R (*R*): 6.2 min, t_R (*S*): 7.5 min, 68% ee, S = 8.

1-(3-Methoxyphenyl)prop-2-en-1-ol 23

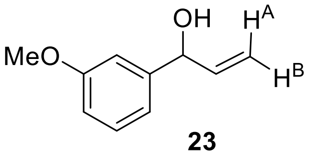

23

Following General Procedure A, vinylmagnesium bromide (5 mL of 0.7 M solution in THF, 3.30 mmol) added to a solution of 3-methoxybenzaldehyde (300 mg, 2.20 mmol) in anhydrous THF (15 mL) at 0 °C for 3 h gave, after purification by column chromatography (80:20 Hexane : EtOAc, R_f 0.28), the title compound **23** (144 mg, 40%) as a colourless oil

with spectroscopic data in accordance with the literature.[8] ^{1}H NMR (500 MHz, $CDCl_3$) δ_H: 1.92 (1H, s, O*H*), 3.82 (3H, s, OCH_3), 5.18 (1H, s, C(1)*H*), 5.20 (1H, dt, *J* 10.2, 1.3, C(3)H^B), 5.36 (1H, dt, *J* 17.0, 1.3, C(3)H^A), 6.04 (1H, ddd, *J* 17.0, 10.2, 6.1, C(2)*H*), 6.83 (2H, ddd, *J* 8.3, 2.5, 1.1, Ar*H*), 6.92–6.99 (3H, m, Ar*H*), 7.23–7.32 (2H, m, Ar*H*).

1-(3-Methoxyphenyl)allyl isobutyrate S6

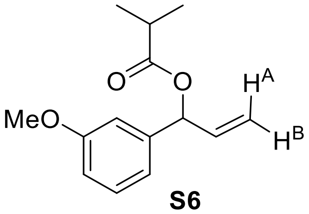

S6

Following General Procedure B, to a solution of DMAP (10 mol %), CH_2Cl_2 (0.1 M) and alcohol **23** (51 mg, 0.31 mmol) was added isobutyric anhydride (50 µL, 0.31 mmol) at room temperature and the reaction mixture was stirred for 16 h. The mixture gave a clean product (no purification) as a colourless oil (52 mg, 72%). ν_{max} (film) 1734 (C=O); ^{1}H NMR (500 MHz, $CDCl_3$) δ_H: 1.19 (3H, d, *J* 7.0, CH(CH_3)), 1.21 (3H, d, *J* 7.0, CH(CH_3)), 2.62 (1H, hept, *J* 7.0, C*H*(CH_3)$_2$), 3.81 (3H, s, OCH_3), 5.23 (1H, dt, *J* 10.4, 1.3, C(3)H^B), 5.30 (1H, dt, *J* 17.1, 1.3, C(3)H^A), 5.98 (1H, ddd, *J* 17.1, 10.4, 5.9, C(2)*H*), 6.22 (1H, d, *J* 5.9, C(1)*H*), 6.84 (1H, ddd, *J* 8.2, 2.6, 0.8, Ar*H*)), 6.87–6.90 (1H, m, Ar*H*), 6.94 (1H, d, *J* 7.6, Ar*H*), 7.27 (2H, t, *J* 7.9, Ar*H*); ^{13}C{^{1}H} NMR (126 MHz, $CDCl_3$) δ_C: 19.1 (C(*C*H_3)$_2$), 34.3 (*C*(CH_3)$_2$), 55.4 (O*C*H_3), 75.8 (*C*(1)), 112.6 (Ar*C*(2)), 113.5 (Ar*C*(4)), 116.9 (*C*(3)), 119.4 (Ar*C*(6)), 129.7 (Ar*C*(5)), 136.5 (*C*(2)), 140.9 (Ar*C*(1)), 159.8 (Ar*C*(3)), 176.1 (*C*=O); HRMS (FTMS + NSI$^+$) $C_{14}H_{18}O_3$ [M+Na]$^+$ found 257.1149, requires 257.1148 (+ 0.3 ppm).

Kinetic resolution of 1-(3-methoxyphenyl)prop-2-en-1-ol 23

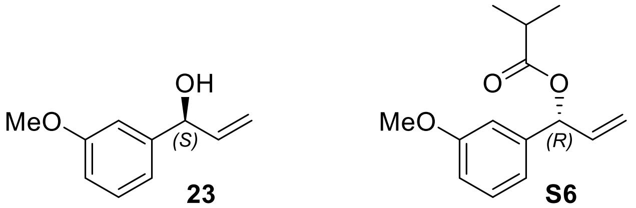

23 **S6**

Following General Procedure C, the alcohol **23** (162 mg, 0.99 mmol), HyperBTM **12** (3 mg, 10 mmol, 1 mol %), *i*-Pr_2NEt (103 µL, 0.59 mmol) and isobutyric anhydride (80 µL, 0.49 mmol) were reacted in toluene (1.2 mL) for 16 h to give the crude product, which was purified via column chromatography (80:20 Petrol : EtOAc, R_f 0.24 (**23**) and R_f 0.67 (**S6**)) to separate alcohol **23** (75 mg, 0.46 mmol, 46%) and ester **S6** (93 mg, 0.40 mmol, 40%). **Alcohol 23:** $[\alpha]_D^{20}$ +5.1 (*c* 1.0, $CHCl_3$); Chiral HPLC analysis Chiralcel OD-H (97.5:2.5

hexane : IPA, flow rate 0.5 mL min-1, 220 nm, 30 °C) t_R (*R*): 46.7 min, t_R (*S*): 50.3 min, 71% ee. **Ester S6:** $[\alpha]_D^{20}$ +43.5 (*c* 1.0, $CHCl_3$); Chiral HPLC analysis Chiralcel OJ-H (99:1 hexane : IPA, flow rate 1.0 mL min-1, 220 nm, 30 °C) t_R (*R*): 7.1 min, t_R (*S*): 8.1 min, 93 % ee. S = 59.

1-(3-(Trifluoromethyl)phenyl)prop-2-en-1-ol 24

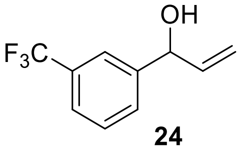

24

Following General Procedure A, vinylmagnesium bromide (6.9 mL of 0.7 M solution in THF, 4.82 mmol) added to a solution of 3-trifluoromethylbenzaldehyde (700 mg, 4.02 mmol) in anhydrous THF (30 mL) at –78 °C for 16 h gave, after purification by column chromatography (80:20 Hexane : EtOAc, R_f 0.40), the title compound **24** (566 mg, 70%) as a yellow oil with spectroscopic data in accordance with the literature.[9] ^{1}H NMR (400 MHz, $CDCl_3$) δ_H: 2.02 (1H, br s, O*H*), 5.25 (1H, dt, *J* 10.3, 1.2, C(3)H^B), 5.28 (1H, s, C(1)*H*), 5.38 (1H, dt, *J* 17.1, 1.3, C(3)H^A), 6.02 (1H, ddd, *J* 17.1, 10.3, 6.3, C(2)*H*), 7.45–7.54 (2H, m, Ar*H*), 7.62 (2H, d, *J* 8.1, Ar*H*); ^{19}F NMR (376 MHz, $CDCl_3$) δ_F: –62.6.

1-(3-(Trifluoromethyl)phenyl)allyl isobutyrate S7

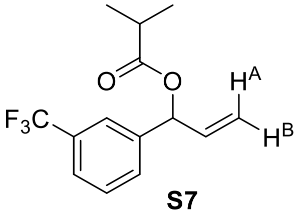

S7

Following General Procedure B, to a solution of DMAP (10 mol %), CH_2Cl_2 (0.1 M) and alcohol **24**(73 mg, 0.36 mmol) was added isobutyric anhydride (59 µL, 0.36 mmol) at room temperature and the reaction mixture was stirred for 16 h. The mixture gave a clean product (84 mg, 87%)as a yellow oil. ν_{max} ($CHCl_3$) 1738 (C=O); ^{1}H NMR ($CDCl_3$, 500 MHz) δ_H: 1.19 (3H, d, *J* 7.0, CH(C*H*3)), 1.21 (3H, d, *J* 7.0, CH(C*H*3)), 2.64 (1H, hept, *J* 7.0, C*H*(CH_3)$_2$), 5.29 (1H, dt, *J* 10.4, 1.2, C(3)H^B), 5.30–5.35 (1H, m, C(3)H^A), 5.98 (1H, ddd, *J* 16.6, 10.4, 5.9, C(2)*H*), 6.29 (1H, d, *J* 5.9, C(1)*H*), 7.48 (1H, t, *J* 7.7, Ar*H*), 7.51–7.58 (2H, m, Ar*H*), 7.60 (1H, s, Ar*H*); ^{13}C{^{1}H} NMR (126 MHz, $CDCl_3$) δ_C: 19.0 (CH(*C*H_3)$_2$), 34.2 (*C*H(CH_3)$_2$), 75.2 (*C*(1)), 117.8 (*C*(3)), 123.8 (q, $^3J_{CF}$ 3.5, Ar*C*(4)), 125.02 (q, $^3J_{CF}$ 3.4, Ar*C*(2)), 125.20 (q,$^1J_{CF}$ 272.3,*C*F_3), 129.2(Ar*C*(5)), 130.5 (Ar*C*(6)), 131.1 (q, $^2J_{CF}$ 32.4, Ar*C*(3)), 135.9 (*C*2), 140.3 (Ar*C*(1)), 176.0 (*C*=O); ^{19}F NMR (376 MHz, $CDCl_3$) δ_F: –62.7; HRMS (ASAP+) $C_{14}H_{15}F_3O_2$ [M]+, found 272.1024, requires 272.1021 (–1.1 ppm).

Kinetic resolution of 1-(3-(trifluoromethyl)phenyl)prop-2-en-1-ol 24

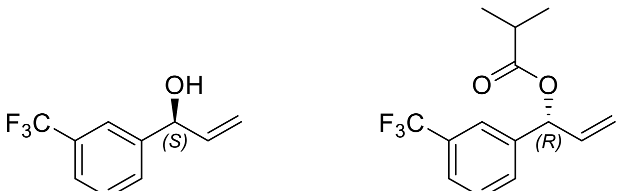

24 **S7**

Following General Procedure C, the alcohol **24** (72 mg, 0.35 mmol), HyperBTM **12** (111 µL from stock solution, 3 µmol, 1 mol %), *i*-Pr_2NEt (37 µL, 0.21 mmol) and isobutyric anhydride (32 µL, 0.19 mmol) were reacted in toluene (1.0 mL) for 16 h to give the crude product, which was purified via column chromatography (80:20 Petrol : EtOAc, R_f 0.40 (**24**) and R_f 0.74 (**S7**)) to separate alcohol **24** (22 mg, 0.11 mmol, 30%) and ester **S7** (28 mg, 0.10 mmol, 29%). **Alcohol 59a:** $[\alpha]_D^{20}$ +10.2 (*c* 1.0, $CHCl_3$); Chiral HPLC analysis Chiralcel OJ-H (99.8:0.2 hexane : IPA, flow rate 1.0 mL min^{-1}, 220 nm, 30 °C) t_R (*S*): 30.5 min, t_R (*R*): 35.0 min, 72% ee. **Ester 59e:** $[\alpha]_D^{20}$ +20.3 (*c* 1.0, $CHCl_3$); Chiral HPLC analysis Chiralpak AD-H (99.8:0.2 hexane : IPA, flow rate 1.0 mL min^{-1}, 211 nm, 30 °C) t_R (*R*): 4.9 min, t_R (*S*): 5.2 min, 75% ee. S = 15.

1-(3-Fluorophenyl)prop-2-en-1-ol 25

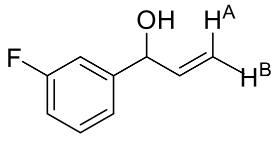

25

Following General Procedure A, vinylmagnesium bromide (8.7 mL of 0.7 M solution in THF, 6.04 mmol) added to a solution of 3-fluorobenzaldehyde (500 mg, 4.03 mmol) in anhydrous THF (15 mL) at 0 °C for 16 h gave, after purification by column chromatography (70:30 Hexane : EtOAc, R_f 0.44), the title compound **25** (393 mg, 64%) as a colourless oil with spectroscopic data in accordance with the literature.[10] ^{1}H NMR (400 MHz, $CDCl_3$) δ_H: 1.96 (1H, s, O*H*), 5.19–5.21 (1H, m, C(1)*H*), 5.23 (1H, dt, *J* 10.2, 1.3, C(3)H^B), 5.37 (1H, dt, *J* 17.0, 1.3, C(3)H^A), 6.02 (1H, ddd, *J* 17.0, 10.2, 6.2, C(2)*H*), 6.93–7.02 (1H, m, Ar*H*), 7.11 (1H, dt, *J* 9.8, 2.1, Ar*H*), 7.14 (1H, d, *J* 7.7, Ar*H*), 7.32 (1H, td, *J* 7.9, 5.9, Ar*H*); ^{13}C{^{1}H} NMR (126 MHz, $CDCl_3$) δ_C: 74.9 (*C*(1)), 113.3 (d, $^2J_{CF}$ 22.0, (Ar*C*(2)), 114.7 (d, $^2J_{CF}$ 21.2, Ar*C*(4)), 116.0 (*C*(3)), 122.0 (d, $^4J_{CF}$ 2.8, Ar*C*(6)), 130.2 (d, $^3J_{CF}$ 8.1, Ar*C*(5)), 139.9 (*C*(2)), 145.2 (d, $^3J_{CF}$ 6.8, Ar*C*(1)), 163.1 (d, $^1J_{CF}$ 246.1, Ar*C*(3)); ^{19}F NMR (377 MHz, $CDCl_3$) δ_F: –112.8.

1-(3-Fluorophenyl)allyl isobutyrate S8

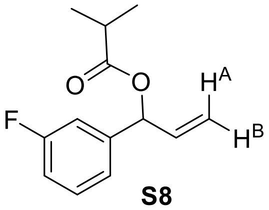

S8

Following General Procedure B, to a solution of DMAP (10 mol %), CH_2Cl_2 (0.1 M) and alcohol **25** (71 mg, 0.47 mmol) was added isobutyric anhydride (76 µL, 0.47 mmol) at room temperature and the reaction mixture was stirred for 16 h. The mixture gave a clean product (87 mg, 84%) as a colourless oil. ν_{max} (film) 1738 (C=O); ^{1}H NMR (500 MHz, $CDCl_3$) δ_H: 1.21 (3H, d, *J* 7.0, CH(C*H*$_3$)$_2$), 1.23 (3H, d, *J* 7.0, CH(C*H*$_3$)$_2$), 2.65 (1H, dq, *J* 14.0, 7.0, C*H*(CH$_3$)$_2$), 5.29 (1H, dt, *J* 10.4, 1.2, C(3)H^B), 5.33 (1H, dt, *J* 17.1, 1.2, C(3)H^A), 5.99 (1H, ddd, *J* 17.1, 10.4, 5.9, C(2)*H*), 6.25 (1H, d, *J* 5.9, C(1)*H*), 6.99–7.04 (1H, m, Ar*H*), 7.08 (1H, dt, *J* 9.7, 2.2, Ar*H*), 7.14 (1H, d, *J* 7.7, Ar*H*), 7.34 (1H, td, *J* 8.0, 5.9, Ar*H*); ^{13}C{^{1}H} NMR (126 MHz, $CDCl_3$) δ_C 19.0 (CH(*C*H$_3$)$_2$), 34.3 (*C*H(CH$_3$)$_2$) , 75.2 (*C*(1)), 114.0 (d, $^{2}J_{CF}$ 22.3, Ar*C*(2)), 115.1 (d, $^{2}J_{CF}$ 21.2, Ar*C*(4)), 117.5 (*C*(3)), 122.7 (d, $^{4}J_{CF}$ 2.7, Ar*C*(6)), 130.2 (d, $^{3}J_{CF}$ 8.2, Ar*C*(5)), 136.0 (*C*(2)), 141.8 (d, $^{3}J_{CF}$ 7.1, Ar*C*(1)), 163.0 (d, $^{1}J_{CF}$ 246.2, Ar*C*(3)), 176.0 (*C*=O); ^{19}F NMR (377 MHz, $CDCl_3$) δ_F: –112.7; HRMS could not be obtained due to significant fragmentation.

Kinetic resolution of 1-(3-fluorophenyl)prop-2-en-1-ol 25

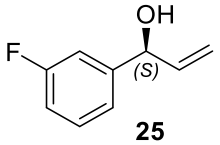

25

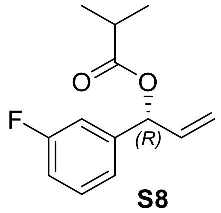

S8

Following General Procedure C, the alcohol **25** (73 mg, 0.48 mmol), HyperBTM **12** (151 µL from stock solution, 5 µmol, 1 mol %), *i*-Pr$_2$NEt (50 µL, 0.29 mmol) and isobutyric anhydride (39 µL, 0.24 mmol) were reacted in toluene (1.3 mL) for 16 h to give the crude product, which was purified via column chromatography (70:30 Hexane : EtOAc, R_f 0.44 (**25**) and R_f 0.80 (**S8**)) to separate alcohol **25** (24 mg, 0.16 mmol, 33%) and ester **S8** (37 mg, 0.17 mmol, 34%). **Alcohol 25:** $[\alpha]^{20}_{D}$ +7.0 (*c* 0.4, $CHCl_3$); 78% ee.[6] **Ester S8:** $[\alpha]^{20}_{D}$ +24.8 (*c* 1.0, $CHCl_3$); Chiral HPLC analysis Chiralpak AD-H (99.8:0.2 hexane : IPA, flow rate 1.0 mL min^{-1}, 211 nm, 30 °C) t_R (*R*): 5.3 min, t_R (*S*): 5.7 min, 67 % ee. S = 12.

1-(2-Methoxyphenyl)prop-2-en-1-ol 26

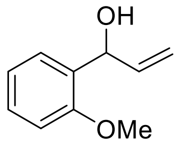

26

Following General Procedure A, vinylmagnesium bromide (5 mL of 0.7M solution in THF, 3.30 mmol) added to a solution of 2-methoxybenzaldehyde (300 mg, 2.20 mmol) in anhydrous THF (15 mL) at 0°C for 3 h gave, after purification by column chromatography (80:20 Petrol : EtOAc, R_f0.28), the title compound **26** (300 mg, 83%) as a yellow oilwith spectroscopic data in accordance with the literature.[8] ^{1}H NMR (400 MHz, $CDCl_3$) δ_H: 2.76 (1H, d, *J* 6.2, O*H*), 3.87 (3H, s, OCH_3), 5.17 (1H, dt, *J* 10.4, 1.5, C(3)H^B), 5.31 (1H, dt, *J* 17.2, 1.6, C(3)H^A), 5.40 (1H, d, *J* 5.3, C(1)*H*), 6.14 (1H, ddd, *J* 17.2, 10.4, 5.5, C(2)*H*), 6.87–6.94 (1H, m, Ar*H*), 6.96 (1H, td, *J* 7.5, 1.1, Ar*H*), 7.22–7.34 (2H, m, Ar*H*).

1-(2-Methoxyphenyl)allyl isobutyrate S9

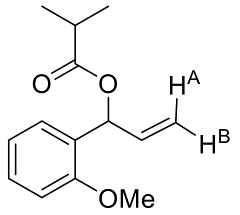

S9

Following General Procedure B, to a solution of DMAP (10 mol %), CH_2Cl_2 (0.1 M) and alcohol **26** (57 mg, 0.35 mmol) was added isobutyric anhydride (56 µL, 0.35 mmol) at room temperature and the reaction mixture was stirred for 16 h. The mixture gave a clean product (no purification) as a orange oil (55 mg, 67%) with spectroscopic data in accordance with the literature.[11] ^{1}H NMR (500 MHz, $CDCl_3$) δ_H: 1.19 (3H, d, *J* 7.0, CH(CH_3)), 1.21 (3H, d, *J* 7.0, CH(CH_3)), 2.62 (1H, hept, *J* 7.0, C*H*(CH_3)), 3.84 (3H, s, OCH_3), 5.14–5.20 (1H, m, C(3)H^B), 5.24 (1H, d, *J* 17.1, C(3)H^A), 6.02 (1H, ddd, *J* 17.1, 10.5, 5.5, C(2)*H*), 6.64 (1H, d, *J* 5.5, C(1)*H*), 6.88 (1H, d, *J* 8.2, ArC(2)*H*), 6.96 (1H, t, *J* 7.5, Ar*H*), 7.26–7.32 (1H, m, Ar*H*), 7.34 (1H, dd, *J* 7.6, 1.5, ArC(6)*H*).

Kinetic resolution of 1-(2-methoxyphenyl)prop-2-en-1-ol 26

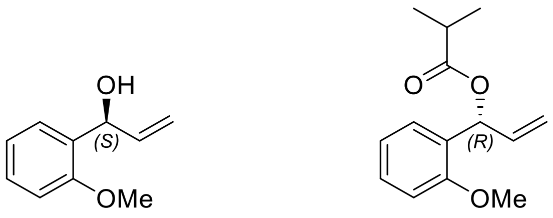

26 **S9**

Following General Procedure C, the alcohol **26** (58 mg, 0.35 mmol), HyperBTM **12** (109 µL from stock solution, 3 µmol, 1 mol %), *i*-Pr_2NEt (31 µL, 0.18 mmol) and isobutyric anhydride (34 µL, 0.21 mmol) were reacted in toluene (2 mL) for 16 h to give the crude product, which was purified via column chromatography (80:20 Petrol : EtOAc, R_f 0.28 (**26**) and R_f 0.58 (**S9**)) to separate alcohol **26** (25 mg, 0.16 mmol, 44%) and ester **S9** (32 mg, 0.13 mmol, 35%). **Alcohol 26:** $[\alpha]_D^{20}$ –5.6 (*c* 0.8, $CHCl_3$); Chiral HPLC analysis Chiralcel OD-H (95:5 hexane : IPA, flow rate 0.5 mL min^{-1}, 270 nm, 30 °C) t_R (*R*): 28.808 min, t_R (*S*): 21.9 min, 90 % ee. **Ester S9:** $[\alpha]_D^{20}$ +26.2 (*c* 1.8, $CHCl_3$); S = 36.

1-(2-(Trifluoromethyl)phenyl)prop-2-en-1-ol 27

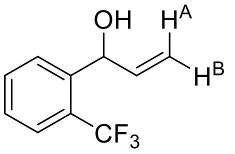

27

Following General Procedure A, vinylmagnesium bromide (6.2 mL of 0.7 M solution in THF, 4.31 mmol) added to a solution of 2-trifluoromethylbenzaldehyde (500 mg, 2.87 mmol) in anhydrous THF (20 mL) at –78 °C for 16 h gave, after purification by column chromatography (80:20 Hexane : EtOAc, R_f 0.36), the title compound **27** (479 mg, 83%) as a colourless oil. ν_{max} (film) 3361 (O-H); ^{1}H NMR (500 MHz, $CDCl_3$) δ_H: 2.04 (1H, d, *J* 3.4, O*H*), 5.22 (1H, dt, *J* 10.5, 1.3, C(3)H^B), 5.40 (1H, dt, *J* 17.1, 1.3, C(3)H^A), 5.62–5.71 (1H, m, C(1)*H*), 6.03 (1H, ddd, *J* 17.2, 10.5, 5.1, C(2)*H*), 7.39 (1H, t, *J* 7.7, Ar(4)*H*), 7.59 (1H, t, *J* 7.6, Ar(5)*H*), 7.64 (1H, d, *J* 7.9, Ar(6)*H*), 7.73 (1H, d, *J* 7.8, Ar(3)*H*); $^{13}C\{^1H\}$ NMR (126 MHz, $CDCl_3$) δ_C: 70.1 (*C*(1)), 115.3 (*C*(3)), 124.5 (q, $^1J_{CF}$ 274.4, CF_3), 125.6 (q, $^3J_{CF}$ 5.9, Ar*C*(3)). 127.4 (q, $^2J_{CF}$ 31.0, Ar*C*(2)).127.9 (Ar*C*), 128.6 (Ar*C*), 132.5 (Ar*C*), 139.5 (*C*(2)), 141.7 (Ar*C*(1)); ^{19}F NMR (376 MHz, $CDCl_3$) δ_F: –58.0; HRMS could not be obtained due to significant fragmentation.

1-(2-(Trifluoromethyl)phenyl)allyl isobutyrate S10

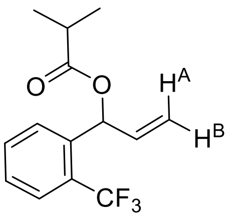

S10

Following General Procedure B, to a solution of DMAP (10 mol %), CH_2Cl_2 (0.1 M) and alcohol **27** (74 mg, 0.37 mmol) was added isobutyric anhydride (60 µL, 0.37 mmol) at room temperature and the reaction mixture was stirred for 16 h. The mixture gave a clean product (77 mg, 77%) as a yellow oil. ν_{max} ($CHCl_3$) 1740 (C=O); ^{1}H NMR ($CDCl_3$, 500 MHz) δ_H: 1.17 (3H, d, *J* 7.0, CH(C*H*$_3$)), 1.20 (3H, d, *J* 7.0, CH(C*H*$_3$)), 2.62 (1H, hept, *J* 7.0, C*H*(CH$_3$)$_2$), 5.21–5.25 (1H, m, C(3)*H*), 5.24–5.26 (1H, m, C(3)*H*), 5.98 (1H, ddd, *J* 17.3, 10.4, 5.2, C(2)*H*), 6.62 (1H, d, *J* 5.1, C(1)*H*), 7.41 (1H, t, *J* 7.6, Ar(4)*H*), 7.57 (1H, t, *J* 7.6, Ar(5)*H*), 7.61 (1H, d, *J* 7.8, Ar(6)*H*), 7.66 (1H, d, *J* 7.9, Ar(3)*H*); ^{13}C{^{1}H} NMR (126 MHz, $CDCl_3$) δ_C: 19.0 (CH(*C*H$_3$)), 19.0 (CH(*C*H$_3$)), 34.2 (*C*H(CH$_3$)$_2$), 71.5 (*C*(1)), 116.9 (*C*(3)), 124.2 (q, $^1J_{CF}$ 274.7, *C*F$_3$), 126.0 (q, $^3J_{CF}$ 5.8, Ar*C*(3)), 127.9 (q, $^2J_{CF}$ 31.7, Ar*C*(2)), 128.2 (Ar*C*), 128.7 (Ar*C*), 132.3 (Ar*C*), 136.1 (*C*(2)), 138.3 (Ar*C*(1)), 175.6 (*C*=O); ^{19}F NMR (376 MHz, $CDCl_3$) δ_F: –58.6; HRMS (NSI$^+$) $C_{14}H_{15}F_3O_2$ [M+NH$_4$]$^+$ found 290.1358, requires 290.1362 (− 1.5 ppm).

Kinetic resolution of 1-(2-(trifluoromethyl)phenyl)prop-2-en-1-ol 27

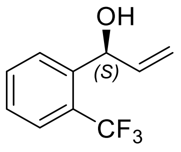

27

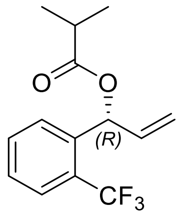

S10

Following General Procedure C, the alcohol **27** (52 mg, 0.25 mmol), HyperBTM **12** (80 µL from stock solution, 2 µmol, 1 mol %), *i*-Pr$_2$NEt (27 µL, 0.15 mmol) and isobutyric anhydride (23 µL, 0.14 mmol) were reacted in toluene (0.7 mL) for 16 h to give the crude product, which was purified via column chromatography (90:10 Petrol : EtOAc, R$_f$ 0.26 (**27**) and R$_f$ 0.58 (**S10**)) to separate alcohol **27** (49 mg, 0.24 mmol, 59%) and ester **S10** (33 mg, 0.12 mmol, 30%). **Alcohol 27:** $[\alpha]_D^{20}$ –21.0 (*c* 1.0, $CHCl_3$); Chiral HPLC analysis Chiralcel OJ-H (95:5 hexane : IPA, flow rate 1.0 mL min^{-1}, 220 nm, 30 °C) t_R (*S*): 6.3 min, t_R (*R*): 7.0 min, 37% ee. **Ester S10:** $[\alpha]_D^{20}$ +36.1 (*c* 1.8, $CHCl_3$); Chiral HPLC analysis Chiralpak AD-H

(99.8:0.2 hexane : IPA, flow rate 1.0 mL min^{-1}, 211 nm, 30 °C) t_R (*R*): 4.2 min, t_R (*S*): 4.5 min, 64% ee. S = 7.

1-(2,6-Dimethoxyphenyl)prop-2-en-1-ol 28

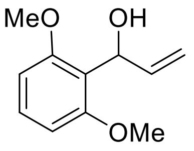

28

To a solution of 1,3-dimethoxybenzene (500 mg, 3.61 mmol) in anhydrous THF at –78 °C under N_2, was added *n*-BuLi (1.8 mL of 1.6 M solution in hexanes, 2.9 mmol). The reaction was stirred at –78 °C for 20 min and then at rt for 2 h. After cooling at –78 °C, acrolein (0.26 mL, 3.61 mmol) was added to the mixture and it was allowed to warm at rt gradually and the reaction was stirred overnight. The mixture was quenched with NH_4Cl and it was extracted with EtOAc (× 3) and the combined organic layers were washed with brine and dried over Na_2SO_4. After evaporation of volatiles, the crude product was purified by silica-gel column chromatography (80:20 Hexane : EtOAc, R_f 0.24), the title compound **28** (467 mg, 67%) as a colourless oil. ν_{max} (film) 2938 (O-H), 1593 (C=C); ^{1}H NMR (500 MHz, $CDCl_3$) δ_H: 3.84 (6H, s, Ar(2,6)OCH_3), 3.98 (1H, d, *J* 11.5, O*H*), 5.05 (1H, dt, *J* 10.3, 1.6, C(3)H^B), 5.18 (1H, dt, *J* 17.2, 1.6, C(3)H^A), 5.67 (1H, ddt, *J* 11.5, 5.4, 1.6, C(1)*H*), 6.17 (1H, ddd, *J* 17.2, 10.3, 5.5, C(2)*H*), 6.57 (2H, d, *J* 8.4, Ar(3,5)*H*), 7.20 (1H, t, *J* 8.4, Ar(4)*H*); ^{13}C{^{1}H} NMR (126 MHz, $CDCl_3$) δ_C: 56.0 (Ar(2,6)O*C*H$_3$), 68.5 (*C*(1)), 104.6 (Ar*C*(3,5)), 113.4 (Ar*C*(1)), 118.5 (*C*(3)), 128.8 (Ar*C*(4)), 140.3 (*C*(2)), 157.8 (Ar*C*(2,6)); HRMS (NSI$^+$) $C_{11}H_{14}O_3$ [M+Na]$^+$ found 217.0834, requires 217.0835 (− 0.5 ppm).

1-(2,6-Dimethoxyphenyl)allyl isobutyrate S11

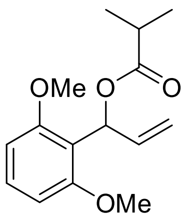

S11

Following General Procedure B, to a solution of DMAP (10 mol %), CH_2Cl_2 (0.1 M) and alcohol **28** (46 mg, 0.24 mmol) was added isobutyric anhydride (39 µL, 0.24 mmol) at room temperature and the reaction mixture was stirred for 16 h. The mixture gave a clean product (41 mg, 88%)as a yellow oil. ν_{max} (film) 1730 (C=O), 1595 (C=C);^{1}H NMR (400 MHz,

$CDCl_3$) δ_H: 1.12 (3H, d, *J* 7.0, CH(C*H*$_3$)), 1.15 (3H, d, *J* 7.0, CH(C*H*$_3$)), 2.55 (1H, hept, *J* 7.0, C*H*(CH$_3$)$_2$), 3.83 (6H, s, Ar(2,6)OC*H*$_3$), 5.12 (1H, dt, *J* 10.3, 1.4, C(3)H^B), 5.25 (1H, dt, *J* 17.3, 1.4, C(3)H^A), 6.29 (1H, ddd, *J* 17.3, 10.3, 6.7, C(2)*H*), 6.55 (2H, d, *J* 8.4, Ar(3,5)*H*), 6.80 (1H, dt, *J* 6.7, 1.3, C(1)*H*), 7.21 (1H, t, *J* 8.4, Ar(4)*H*);$^{13}C\{^1H\}$ NMR (126 MHz, $CDCl_3$) δ_C: 19.0 (CH(*C*H$_3$)), 19.1 (CH(*C*H$_3$)), 34.2 (*C*H(CH$_3$)), 56.1 (O*C*H$_3$), 69.5 (*C*(1)), 104.6 (Ar*C*(3,5)), 115.6 (Ar*C*(1)), 116.4 (*C*(3)), 129.7 (Ar*C*(4)), 136.0 (*C*(2)), 158.8 (Ar*C*(2,6)), 176.5 (*C*=O); HRMS (FTMS + NSI$^+$) $C_{15}H_{20}O_4$ [M+Na]$^+$ found 287.1255, requires 287.1254 (+ 0.4 ppm).

Kinetic resolution of 1-(2,6-dimethoxyphenyl)prop-2-en-1-ol 28

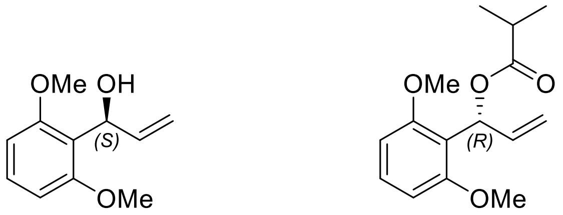

28 **S11**

Following General Procedure C, the alcohol **28** (84 mg, 0.43 mmol), HyperBTM **12** (135 µL from stock solution, 4 µmol, 1 mol %), *i*-Pr$_2$NEt (45 µL, 0.26 mmol) and isobutyric anhydride (35 µL, 0.21 mmol) were reacted in toluene (1.2 mL) for 48 h to give the crude product, which was purified via column chromatography (80:20 Petrol : EtOAc, R_f 0.16 (**28**) and R_f 0.54 (**S11**)) to separate alcohol **28** (33 mg, 0.17 mmol, 40%) and ester **S11** (30 mg, 0.11 mmol, 26%). **Alcohol 28:** $[\alpha]_D^{20}$ +1.1 (*c* 1.8, $CHCl_3$); Chiral HPLC analysis Chiralcel OD-H (95:5 hexane : IPA, flow rate 1.0 mL min^{-1}, 211 nm, 30 °C) t_R (*R*): 14.2 min, t_R (*S*): 24.9 min, 56% ee. **Ester S11:** $[\alpha]_D^{20}$ +2.7 (*c* 0.6, $CHCl_3$); Chiral HPLC analysis Chiralcel OJ-H (99:1 hexane : IPA, flow rate 1.0 mL min^{-1}, 211 nm, 30 °C) t_R (*S*): 6.9 min, t_R (*R*): 8.5 min, 97 % ee. S = 110.

1-(3,4-Dimethoxyphenyl)prop-2-en-1-one S13

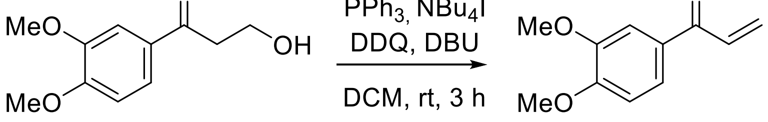

S12 **S13**

DDQ (1.08 g, 4.76 mmol, 2.0 eq) was added to a stirring solution of PPh_3 (1.25 g, 4.76 mmol, 2.0 eq) and NBu_4I (1.76 g, 4.76 mmol, 2.0 eq) in CH_2Cl_2 (18 mL) at room temperature. The resulting mixture was left to stir for 10 min. Afterwards, a solution of **S12**[12] (0.5 g, 2.38 mmol, 1.0 eq) in CH_2Cl_2 (5 mL) was added at room temperature and left to stir for a further 2

h. Upon completion (TLC control), DBU (0.72 g, 4.76 mmol, 2.0 eq) was added at room temperature and the reaction stirred for a further 1 h. Crude **S13** (0.30 g, 1.56 mmol, 66%) was used immediately without further purification.

1-(3,4-Dimethoxyphenyl)prop-2-en-1-ol 29

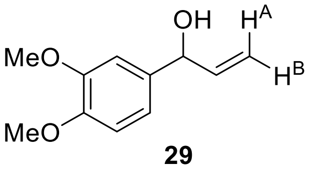

29

$NaBH_4$ (0.29 g, 7.81mmol, 5.0 eq) was added, gradually, to a cooled stirring solution of $CeCl_3 \cdot 7H_2O$ (0.69 g, 1.87mmol, 1.2 eq) and **S13** (0.30 g, 1.56 mmol, 1.0 eq) in MeOH (15 mL). Purification by column chromatography (90:10 Petrol : EtOAc) gave (±)-**29** (0.27 g, 1.39mmol, 89%) as yellow oil with spectroscopic data in accordance with the literature.[13] ^{1}H NMR (300 MHz, $CDCl_3$) δ_H: 1.98 (1H, br s, O*H*), 3.86 (3H, s, OC*H*$_3$), 3.87 (3H, s, OC*H*$_3$), 5.14 (1H, d, *J* 5.8, C(1)*H*), 5.18 (1H, dt, *J* 10.3, 1.4, C(3)H^B), 5.33 (1H, dt, *J* 17.1, 1.4, C(3)H^A), 6.03 (1H, ddd, *J* 16.1, 10.3, 5.8, C(2)*H*), 6.81–6.88 (2H, m, Ar*H*), 6.91 (1H, s, Ar*H*).

1-(3,4-Dimethoxyphenyl)allyl isobutyrate S14

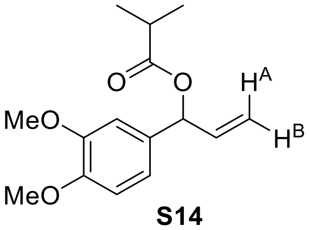

S14

Following General Procedure B, to a solution of DMAP (10 mol%), CH_2Cl_2 (0.2 M) and alcohol **29** (103 mg, 0.53 mmol) was added isobutyric anhydride (87 µL, 0.53 mmol) at room temperature and the reaction mixture was stirred for 16 h. The mixture gave a clean product (97 mg, 69%) as colourless oil. ν_{max} (film) 1728 (C=O), 1512 (C=C); ^{1}H NMR (500 MHz, $CDCl_3$) δ_H: 1.17 (3H, d, *J* 7.0, CH(C*H*$_3$)), 1.20 (3H, d, *J* 7.0, CH(C*H*$_3$)), 2.61 (1H, hept, *J* 7.0, C*H*(CH_3)$_2$), 3.88 (6H, d, *J* 3.5, Ar(3,4)OC*H*$_3$), 5.23 (1H, dt, *J* 10.5, 1.3, C(3)H^B), 5.29 (1H, dt, *J* 17.1, 1.4, C(3)H^A), 6.00 (1H, ddd, *J* 17.1, 10.5, 5.6, C(2)*H*), 6.21 (1H, d, *J* 5.6, C(1)*H*), 6.84 (1H, d, *J* 8.2, Ar(5)*H*), 6.86 (1H, d, *J* 2.0, Ar(2)*H*), 6.92 (1H, dd, *J* 8.3, 1.9, Ar(6)*H*); ^{13}C{^{1}H} NMR (126 MHz, $CDCl_3$) δ_C: 19.1 (CH(*C*H$_3$)$_2$), 34.3 (*C*H(CH_3)$_2$), 56.0 (ArC(3)O*C*H$_3$), 56.0 (ArC(4)O*C*H$_3$), 75.7 (*C*(1)) , 110.4 (Ar*C*(2)), 111.1 (Ar*C*(5)), 116.5 (*C*(3)), 119.8 (Ar*C*(6)), 131.8 (Ar*C*(1)), 136.6 (*C*(2)), 149.0 (Ar*C*(3)), 149.1 (Ar*C*(4)), 176.1 (*C*=O); HRMS (NSI$^+$) $C_{15}H_{20}O_4$ [M+Na]$^+$ found 287.1255, requires 287.1254 (+0.4 ppm).

Kinetic resolution of 1-(3,4-dimethoxyphenyl)prop-2-en-1-ol 29

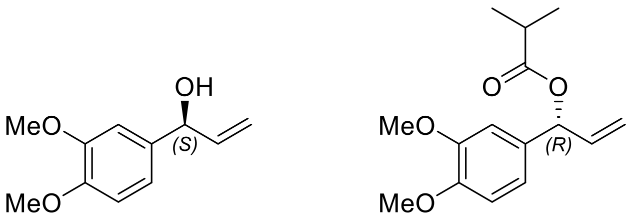

29 S14

Following General Procedure C, the alcohol **29** (92 mg, 0.47 mmol), HyperBTM **12** (148 µL from stock solution, 5 µmol, 1 mol %), *i*-Pr_2NEt (49 µL, 0.28 mmol) and isobutyric anhydride (42 µL, 0.26 mmol) were reacted in toluene (1.5 mL) for 16 h to give the crude product, which was purified via column chromatography (70:30 Petrol : EtOAc, R_f 0.19 (**29**) and R_f 0.67 (**S14**)) to separate alcohol **29** (36 mg, 0.18 mmol, 39%) and ester **S14** (62 mg, 0.24 mmol, 50%). **Alcohol 29:** $[\alpha]_D^{20}$ –1.2 (*c* 1.0, $CHCl_3$); Chiral HPLC analysis Chiralcel OJ-H (92:8 hexane : IPA, flow rate 1.0 mL min^{-1}, 220 nm, 30 °C) t_R : 32.1 min, t_R : 35.2 min, >99% ee. **Ester S14:** $[\alpha]_D^{20}$ +42.0 (*c* 2.0, $CHCl_3$); Chiral HPLC analysis Chiralcel OJ-H (99:1 hexane : IPA, flow rate 1.0 mL min^{-1}, 211 nm, 30 °C) t_R : 15.4 min, t_R : 18.3 min, 60% ee. S = 44.

1-(3,4,5-Trimethoxyphenyl)prop-2-en-1-one S16

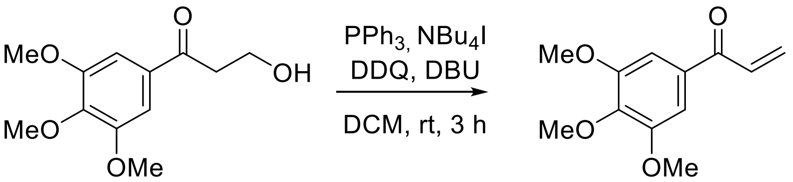

S15 S16

DDQ (0.94 g, 4.17 mmol, 2.0 eq) was added to a stirring solution of PPh_3 (1.09 g, 4.17 mmol, 2.0 eq) and NBu_4I (1.54 g, 4.17 mmol, 2.0 eq) in CH_2Cl_2 (15 mL) at room temperature. The resulting mixture was left to stir for 10 min. Afterwards, a solution of **S15**[12] (0.5 g, 2.08 mmol, 1.0 eq) in CH_2Cl_2 (5 mL) was added at room temperature and left to stir for a further 2 h. Upon completion (TLC control), DBU (0.63 g, 4.17 mmol, 2.0 eq) was added at room temperature and stirred for a further 1 h. Afterwards, the mixture was neutralised with a saturated aqueous solution of NH_4Cl (150 mL) and the aqueous layer was further extracted with CH_2Cl_2 (100 mL). The combined organic layers were washed with water (50 mL), brine (60 mL), dried with $MgSO_4$, filtered and concentrated in vacuo. Purification by silica gel chromatography (98:2 to 95:5 Petrol : EtOAc) gave **S16** (0.40 g, 1.80 mmol, 87%) as colourless oil with spectroscopic data in accordance with the literature.[14] ^1H NMR (500

MHz, $CDCl_3$) δ_H: 3.91 (9H, s, Ar(3,4,5)OC*H*$_3$), 5.91 (1H, dd, *J* 10.6, 1.7, C(3)H^B), 6.43 (1H, dd, *J* 17.0, 1.6, C(3)H^A), 7.14 (1H, dd, *J* 17.1, 10.5, C(2)*H*), 7.20 (2H, s, Ar(2,6)*H*).

1-(3,4,5-Trimethoxyphenyl)prop-2-en-1-ol 30

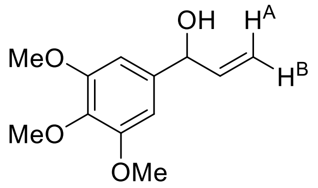

30

$NaBH_4$ (0.26 g, 6.75mmol, 5.0 eq) was added, gradually, to a cooled stirring solution of $CeCl_3.7H_2O$ (0.60 g, 1.62mmol, 1.2 eq) and **S16** (0.30 g, 1.35 mmol, 1.0 eq) in MeOH (13 mL). The resulting mixture was left to stir at 0 °C for 1 h. Afterwards, the mixture was quenched with a saturated aqueous solution of ammonium chloride (50 mL) and extracted with ethyl acetate twice (2 x 75 mL). The combined organic layers were washed with water (40 mL), brine (50 mL), dried with $MgSO_4$, filtered and concentrated in vacuo. Purification by silica gel chromatography using (90:10 to 80:20 Petrol : EtOAc) gave (±)-**30** (0.26 g, 1.16 mmol, 85%) as light-yellow oil with spectroscopic data in accordance with the literature.[15] ^{1}H NMR (500 MHz, $CDCl_3$) δ_H: 2.63 (1H, br s, O*H*), 3.76 (3H, s, Ar(4)OC*H*$_3$), 3.79 (6H, s, Ar(3,5)OC*H*$_3$), 5.05 (1H, d, *J* 5.9, C(1)*H*), 5.13 (1H, dt, *J* 10.3, 1.1, C(3)H^B), 5.29 (1H, dt, *J* 17.1, 1.1, C(3)H^A), 5.96 (1H, ddd, *J* 16.7, 10.3, 5.9, C(2)*H*), 6.53 (2H, s, Ar(2,6)*H*).

1-(3,4,5-Trimethoxyphenyl)allyl isobutyrate S17

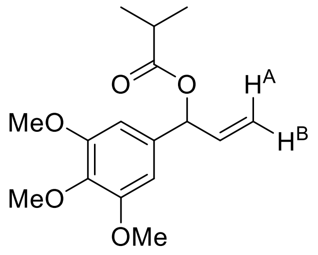

S17

Following General Procedure B, to a solution of DMAP (10 mol%), CH_2Cl_2 (0.2 M) and alcohol **30** (85 mg, 0.38 mmol) was added isobutyric anhydride (62 µL, 0.38 mmol) at room temperature and the reaction mixture was stirred for 16 h. The mixture gave a clean product (94 mg, 85%) as colourless oil. ν_{max} (film) 1728 (C=O), 1589 (C=C); ^{1}H NMR (400 MHz, $CDCl_3$) δ_H: 1.21 (6H, t, *J* 7.0, CH(C*H*$_3$)$_2$), 2.63 (1H, hept, *J* 7.0, C*H*(CH_3)$_2$), 3.84 (3H, s, OC*H*$_3$), 3.86 (6H, s, OC*H*$_3$), 5.25 (1H, dt, *J* 10.4, 1.3, C(3)H^B), 5.31 (1H, dt, *J* 17.1, 1.4, C(3)H^A), 5.99 (1H, ddd, *J* 17.1, 10.4, 5.8, C(2)*H*), 6.18 (1H, d, *J* 5.8, C(1)*H*), 6.56 (2H, s,

Ar(2,6)*H*); $^{13}C\{^{1}H\}$ NMR (101 MHz, $CDCl_3$) δ_C: 19.1 (CH(*C*H_3)), 19.1 (CH(*C*H_3)), 34.3 (*C*H(CH_3)$_2$), 56.2 (ArC(3,5)O*C*H_3), 61.0 (ArC(4)O*C*H_3), 75.9 (*C*(1)), 104.1 (Ar*C*(2,6)), 116.9 (*C*(3)), 134.9 (Ar*C*(1)), 136.4 (*C*(2)), 137.8 (Ar*C*(4)), 153.4 (Ar*C*(3,5)), 176.1 (C=O); HRMS (ASAP) $C_{16}H_{22}O_5$ [M] found 294.1459, requires 294.1467 (– 2.7 ppm).

Kinetic resolution of 1-(3,4,5-trimethoxyphenyl)prop-2-en-1-ol 30

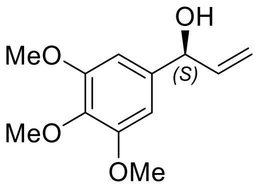

30

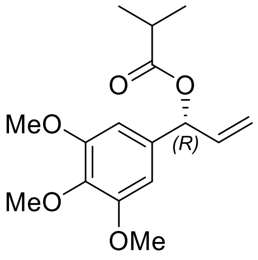

S17

Following General Procedure C, the alcohol **30** (81 mg, 0.36 mmol), HyperBTM **12** (113 µL from stock solution, 4 µmol, 1 mol %), *i*-Pr_2NEt (38 µL, 0.22 mmol) and isobutyric anhydride (32 µL, 0.20 mmol) were reacted in toluene (1.5 mL) for 16 h to give the crude product, which was purified via column chromatography (70:30 Petrol : EtOAc, R_f 0.16 (**30**) and R_f 0.57 (**S17**)) to separate alcohol **30** (35 mg, 0.15 mmol, 43%) and ester **S17** (50 mg, 0.17 mmol, 47%). **Alcohol 30:** $[\alpha]_D^{20}$ –5.2 (*c* 0.5, $CHCl_3$); Chiral HPLC analysis Chiralpak AD-H (95:5 hexane : IPA, flow rate 1.0 mL min^{-1}, 211 nm, 30 °C) t_R (*S*): 22.2 min, t_R (*R*): 27.0 min, 87% ee. **Ester S17:** $[\alpha]_D^{20}$ +32.2 (*c* 1.0, $CHCl_3$); Chiral HPLC analysis Chiralpak AD-H (99:1 hexane : IPA, flow rate 1.0 mL min^{-1}, 254 nm, 30 °C) t_R (*R*): 20.7 min, t_R (*S*): 29.9 min, 84% ee. S = 33.

1-Mesitylprop-2-en-1-ol 31

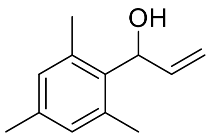

31

Following General Procedure A, mesitylmagnesium bromide (17.2 mL of 1.0 M solution in Et_2O, 17.12 mmol) added to a solution of acrolein (800 mg, 14.27 mmol) in anhydrous THF (50 mL) at –78 °C for 16 h gave, after purification by column chromatography (90:10 Hexane : EtOAc, R_f 0.30), the title compound **31** (1.77 g, 70%) as a white solid with spectroscopic data in accordance with the literature.[16] mp 47–48 °C {Lit.[16] 52–54 °C}; ^{1}H NMR (500 MHz, $CDCl_3$) δ_H: 1.82 (1H, d, *J* 3.6, O*H*), 2.26 (3H, s, Ar(4)C*H*$_3$), 2.38 (6H, s, Ar(2,6)C*H*$_3$), 5.16 (1H, dt, *J* 10.5, 1.8, C(3)H^B), 5.21 (1H, dt, *J* 17.3, 1.7, C(3)H^A), 5.70 (1H,

tt, *J* 4.2, 2.0, C(1)*H*), 6.15 (1H, ddd, *J* 17.3, 10.5, 4.2, C(2)*H*), 6.84 (2H, s, 3,5-Ar*H*).

1-Mesitylallyl isobutyrate S18

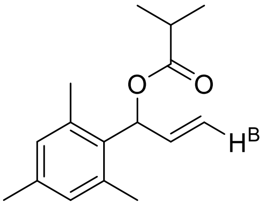

S18

Following General Procedure B, to a solution of DMAP (10 mol %), CH_2Cl_2 (0.2 M) and alcohol **31** (98 mg, 0.56 mmol) was added isobutyric anhydride (91 µL, 0.56 mmol) at room temperature and the reaction mixture was stirred for 16 h. The mixture gave a clean **S18** (98 mg, 72%) as a colourless oil. ν_{max} ($CHCl_3$) 1732 (C=O); ^{1}H NMR (500 MHz, $CDCl_3$) δ_H: 1.15 (3H, d, *J* 7.0, CH(C*H*$_3$)), 1.20 (3H, d, *J* 7.0, CH(C*H*$_3$)), 2.25 (3H, s, Ar(4)C*H*$_3$), 2.40 (6H, s, Ar(2,6)C*H*$_3$), 2.59 (1H, hept, *J* 7.0, C*H*(CH_3)$_2$), 5.09 (1H, dt, *J* 17.3, 1.6, C(3)H^A), 5.17 (1H, dt, *J* 10.6, 1.6, C(3)H^B), 6.05 (1H, ddd, *J* 17.3, 10.6, 4.3, C(2)*H*), 6.68 (1H, dt, *J* 4.3, 2.1, C(1)*H*), 6.83 (2H, s, Ar(3,5)*H*); ^{13}C{^{1}H} NMR (126 MHz, $CDCl_3$) δ_C: 19.0 (CH(*C*H$_3$)), 19.2 (CH(*C*H$_3$)), 20.6 (Ar(2,6)*C*H$_3$), 21.0 (Ar(4)*C*H$_3$), 34.3 (CO*C*H), 72.7 (*C*(1)), 115.9 (*C*(3)), 129.9 (Ar*C*(3,5)), 131.9 (Ar*C*(4)), 135.7 (Ar*C*(2,6)), 137.2 (Ar*C*(1)), 137.6 (*C*(2)), 176.2 (*C*=O); HRMS (NSI$^+$) $C_{16}H_{22}O_2$ [M+Na]$^+$ found 269.1512, requires 269.1512 (–0.0 ppm).

Kinetic resolution of 1-mesitylprop-2-en-1-ol 31

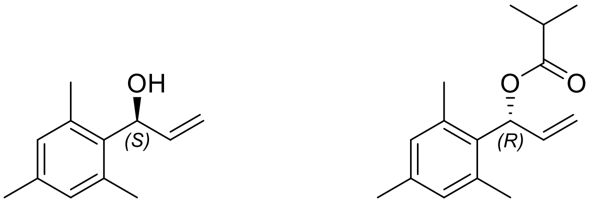

31 **S18**

Following General Procedure C, the alcohol **31** (84 mg, 0.48 mmol), HyperBTM **12** (149 µL from stock solution, 5 µmol, 1 mol %), *i*-Pr_2NEt (50 µL, 0.18 mmol) and isobutyric anhydride (43 µL, 0.16 mmol) were reacted in toluene (1.4 mL) for 16 h to give the crude product, which was purified via column chromatography (80:20 Hexane : EtOAc, R_f 0.49 (**31**) and R_f 0.80 (**S18**)) to separate alcohol **31** (42 mg, 0.24 mmol, 51%) and ester **S18** (20 mg, 0.08 mmol, 17%). **Alcohol 31:** $[\alpha]_D^{20}$ –7.5 (*c* 1.7, $CHCl_3$); Chiral HPLC analysis Chiralcel OD-H (99:1 hexane : IPA, flow rate 1.0 mL min^{-1}, 211 nm, 30 °C) t_R (*R*): 16.1 min, t_R (*S*): 18.8 min, 22% ee. **Ester S18:** $[\alpha]_D^{20}$ +28.7 (*c* 0.9, $CHCl_3$); Chiral HPLC analysis Chiralpak

AD-H (99.5:0.5 hexane : IPA, flow rate 1.0 mL min^{-1}, 220 nm, 30 °C) t_R (*R*): 4.1 min, t_R (*S*): 4.5 min, 79 % ee. S = 11.

1-(Naphthalen-2-yl)prop-2-en-1-ol 32

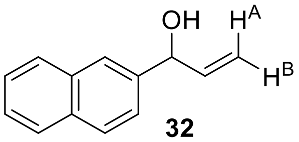

32

Following General Procedure A, vinylmagnesium bromide (5.5 mL of 0.7 M solution in THF, 3.84 mmol) added to a solution of 2-naphthaldehyde (400 mg, 2.56 mmol) in anhydrous THF (13 mL) at –78 °C for 16 h gave, after purification by column chromatography (80:20 Hexane : EtOAc, R_f 0.32), the title compound **32** (439 mg, 93%) as a yellow oil with spectroscopic data in accordance with the literature.[7] ^{1}H NMR (500 MHz, $CDCl_3$) δ_H: 2.01 (1H, d, *J* 3.8, O*H*), 5.25 (1H, dt, *J* 10.3, 1.3 Hz C(3)H^B), 5.39 (1H, d, *J* 6.0, C(1)*H*), 5.42 (1H, dt, *J* 17.1, 1.3, C(3)H^A), 6.13 (1H, ddd, *J* 17.0, 10.3, 6.0, C(2)*H*), 7.49 (3H, ddd, *J* 7.9, 5.3, 1.6, Ar*H*), 7.84 (4H, dt, *J* 6.0, 3.1, Ar*H*).

1-(Naphthalen-2-yl)allyl isobutyrate 37

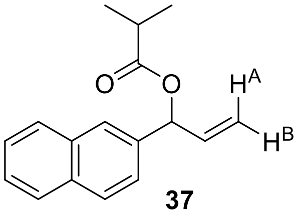

37

Following General Procedure B, to a solution of DMAP (10 mol %), CH_2Cl_2 (0.2 M) and alcohol **32** (71 mg, 0.39 mmol) was added isobutyric anhydride (63 µL, 0.39 mmol) at room temperature and the reaction mixture was stirred for 16 h. The mixture gave a clean product (99 mg, 100%) as a yellow oil. ν_{max} ($CHCl_3$) 1734 (C=O); ^{1}H NMR (500 MHz, $CDCl_3$) δ_H: 1.19 (3H, d, *J* 7.0, CH(CH_3)), 1.22 (3H, d, *J* 7.0, CH(CH_3)), 2.65 (1H, hept, *J* 7.0, C*H*(CH_3)), 5.28 (1H, dt, *J* 10.5, 1.3, C(3)H^B), 5.35 (1H, dt, *J* 17.2, 1.3, C(3)H^A), 6.09 (1H, ddd, *J* 17.2, 10.5, 5.7, C(2)*H*), 6.42 (1H, d, *J* 5.7, C(1)*H*), 7.43–7.53 (3H, m, Ar*H*), 7.79–7.88 (4H, m, Ar*H*); ^{13}C{^{1}H} NMR (101 MHz, $CDCl_3$) δ_C: 19.1 (CH(CH_3)$_2$), 34.4 (*C*H(CH_3)$_2$), 76.0 (*C*(1)), 117.1 (*C*(3)), 125.0 (Ar*C*), 126.3 (Ar*C*), 126.3 (Ar*C*), 126.4 (Ar*C*), 127.8 (Ar*C*), 128.2 (Ar*C*), 128.5 (Ar*C*), 133.2 (Ar*C*), 133.3 (Ar*C*), 136.5 (Ar*C*), 136.6 (*C*(2)), 176.1 (*C*=O); HRMS (NSI^+) $C_{17}H_{18}NO_2$ $[M+Na]^+$ found 277.1198, requires 277.1199 (–0.4 ppm).

Kinetic resolution of 1-(naphthalen-2-yl)prop-2-en-1-ol 32

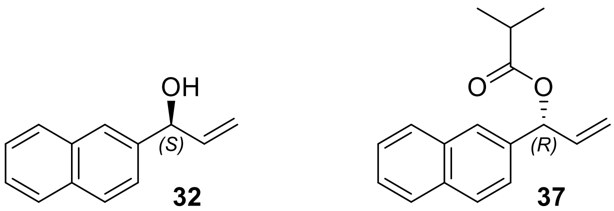

Following General Procedure C, the alcohol **32** (101 mg, 0.55 mmol), HyperBTM **12** (171 µL from stock solution, 5 µmol, 1 mol %), *i*-Pr_2NEt (57 µL, 0.33 mmol) and isobutyric anhydride (45 µL, 0.27 mmol) were reacted in toluene (1.9 mL) for 16 h to give the crude product, which was purified via column chromatography 80:20 Petrol : EtOAc, R_f 0.25 (**32**) and R_f 0.70 (**37**)) to separate alcohol **32** (48 mg, 0.26 mmol, 47%) and ester **37** (63 mg, 0.25 mmol, 45%). **Alcohol 32:** $[\alpha]_D^{20}$ +10.1 (*c* 0.8, $CHCl_3$) {Lit.[3] (ent) $[\alpha]_D^{25}$ –3.0 (*c* 0.3, $CHCl_3$)}; Chiral HPLC analysis Chiralcel OJ-H (80:20 hexane : IPA, flow rate 1.0 mL min^{-1}, 254 nm, 30 °C) t_R (*S*): 11.7 min, t_R (*R*): 14.1 min, 94% ee. **Ester 37:** $[\alpha]_D^{20}$ +54.0 (*c* 1.0, $CHCl_3$); Chiral HPLC analysis Chiralcel OJ-H (95:5 hexane : IPA, flow rate 0.5 mL min^{-1}, 270 nm, 30 °C) t_R (*S*): 13.4 min, t_R (*R*): 15.8 min, >99 % ee. S = 1854.

1-(Naphthalen-1-yl)prop-2-en-1-ol 33

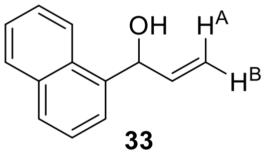

Following General Procedure A, vinylmagnesium bromide (6.9 mL of 0.7 M solution in THF, 4.8 mmol) added to a solution of 1-naphthaldehyde (500 mg, 3.20 mmol) in anhydrous THF (12 mL) at –78 °C for 16 h gave, after purification by column chromatography (70:30 Hexane : EtOAc, R_f 0.48), the title compound **33** (415 mg, 70%) as a yellow oil with spectroscopic data in accordance with the literature.[17] ^{1}H NMR (500 MHz, $CDCl_3$) δ_H: 2.05 (1H, s, O*H*), 5.29 (1H, d, *J* 10.4, C(3)H^B), 5.46 (1H, d, *J* 17.2, C(3)H^A), 5.96 (1H, d, *J* 5.1, C(1)*H*), 6.26 (1H, ddd, *J* 17.1, 10.4, 5.4, C(2)*H*), 7.45–7.57 (3H, m, Ar*H*), 7.63 (1H, d, *J* 7.1, Ar*H*), 7.79–7.93 (2H, m, Ar*H*), 8.20 (1H, d, *J* 8.3, Ar*H*).

1-(Naphthalen-1-yl)allyl isobutyrate S19

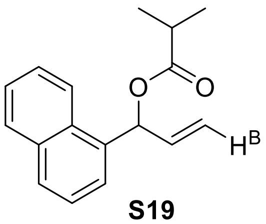

S19

Following General Procedure B, to a solution of DMAP (10 mol %), CH_2Cl_2 (0.2 M) and alcohol **33** (78 mg, 0.42 mmol) was added isobutyric anhydride (69 µL, 0.42 mmol) at room temperature and the reaction mixture was stirred for 16 h. The mixture gave a clean product (83 mg, 78%) as a yellow oil. ν_{max} ($CHCl_3$) 2974 (C=C), 1732 (C=O); ^{1}H NMR (400 MHz, $CDCl_3$) δ_H: 1.17 (3H, d, *J* 7.0, CH(C*H3*)), 1.22 (3H, d, *J* 7.0, CH(C*H3*)), 2.54–2.73 (1H, m, C*H*(CH_3)), 5.24–5.35 (2H, m, C(3)*H2*), 6.19 (1H, ddd, *J* 15.7, 10.5, 5.3, C(2)*H*), 6.97 (1H, d, *J* 3.6, C(1)*H*), 7.38–7.55 (3H, m, Ar*H*), 7.54–7.65 (1H, m, Ar*H*), 7.85 (2H, dd, *J* 17.6, 7.8, Ar*H*), 8.12 (1H, d, *J* 7.8, Ar*H*); ^{13}C{^{1}H} NMR (126 MHz, $CDCl_3$) δ_C: 19.1 (CH(*C*H_3)$_2$), 34.4 (*C*H(CH_3)$_2$) , 73.5 (*C*(1)), 117.2 (*C*(3)), 124.0 (Ar*C*), 125.4 (Ar*C*), 125.5 (Ar*C*), 125.9 (Ar*C*), 126.4 (Ar*C*), 128.9 (Ar*C*), 129.1 (Ar*C*), 130.9 (Ar*C*), 134.0 (Ar*C*), 134.8 (Ar*C*), 136.2 (*C*(2)), 176.2 (*C*=O); HRMS (NSI$^+$) $C_{17}H_{18}O_2$ [M+Na]$^+$ found 277.1191, requires 277.1199 (–2.9 ppm).

Kinetic Resolution of 1-(naphthalen-1-yl)prop-2-en-1-ol 33

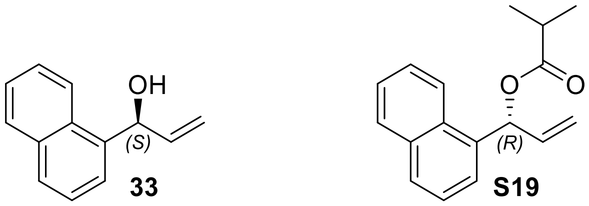

Following General Procedure C, the alcohol **33** (74 mg, 0.40 mmol), HyperBTM **12** (125 µL from stock solution, 4 µmol, 1 mol %), *i*-Pr_2NEt (42 µL, 0.24 mmol) and isobutyric anhydride (33 µL, 0.20 mmol) were reacted in toluene (1.2 mL) for 16 h to give the crude product, which was purified via column chromatography 70:30 Petrol : EtOAc, R_f 0.48 (**33**) and R_f 0.78 (**S19**)) to separate alcohol **33** (30 mg, 0.16 mmol, 41%) and ester **S19** (22 mg, 0.13 mmol, 31%). **Alcohol 33:** $[\alpha]_D^{20}$ –29.2 (*c* 1.0, $CHCl_3$) {Lit.[3] (ent) $[\alpha]_D^{25}$ +36.9 (*c* 0.4, $CHCl_3$)}; Chiral HPLC analysis Chiralcel OJ-H (80:20 hexane : IPA, flow rate 1.0 mL min^{-1}, 211 nm, 30 °C) t_R (*S*): 9.2 min, t_R (*R*): 11.8 min, 84% ee. **Ester S19:** $[\alpha]_D^{20}$ +56.6 (*c* 0.8, $CHCl_3$); Chiral HPLC analysis Chiralcel OJ-H (95:5 hexane : IPA, flow rate 0.5 mL min^{-1}, 211 nm, 30 °C) t_R (*R*): 12.3 min, t_R (*S*): 13.5 min, 96 % ee. S = 108.

1-(6-Methoxynaphthalen-2-yl)prop-2-en-1-ol 34

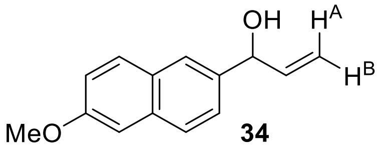

34

To a suspension of magnesium turnings (437 mg, 18 mmol) in anhydrous THF (18 mL) stirred under N_2 was added drop wise a solution of 2-bromo-6-methoxynaphtalene (2.84 g, 12 mmol) in anhydrous THF. The reaction was left for 1 h stirring and after it was heated under reflux for 1 h and then cooled to rt. The product, 2-bromo-6-methoxynaphtalylmagnesium bromide (15 mL of 0.4 M solution in THF fresh-made, 6 mmol) was added drop wise to a solution of acrolein (280 mg, 5.00 mmol) in anhydrous THF at –78 °C. The reaction was stirred at –78 °C for 3 h, then it was quenched with NH_4Cl and it was extracted with EtOAc (× 3) and the combined organic layers were washed with brine and dried over Na_2SO_4. After evaporation of volatiles, the crude product was purified by silica-gel column chromatography (80:20 Hexane : EtOAc, R_f 0.23), the title compound **34** (630 mg, 59%) as a white solid. mp 52–54 °C; ν_{max} (film) 3383 (O-H); 1607 (C=C); ^{1}H NMR (500 MHz, $CDCl_3$) δ_H: 1.99 (1H, d, *J* 3.8, O*H*), 3.92 (3H, s, OC*H₃*), 5.24 (1H, dt, *J* 10.3, 1.4, C(3)H^B), 5.33–5.37 (1H, m, C(1)*H*), 5.41 (1H, dt, *J* 17.1, 1.4, C(2)H^A), 6.13 (1H, ddd, *J* 17.1, 10.3, 5.9, C(2)*H*), 7.12–7.17 (2H, m, Ar(5,7)*H*), 7.45 (1H, dd, *J* 8.5, 1.7, Ar(3)*H*), 7.73 (2H, dd, *J* 8.5, 3.7, Ar(4,8)*H*), 7.76 (1H, s, Ar(1)*H*); ^{13}C{^{1}H} NMR (126 MHz, $CDCl_3$) δ_C: 55.5 (O*C*H₃), 75.6 (*C*(1)), 105.8 (Ar*C*(5)), 115.4 (*C*(3)), 119.2 (Ar*C*(7)), 125.1 (Ar*C*(4)), 125.2 (Ar*C*(1)), 127.4 (Ar*C*(3)), 128.9 (Ar*C*(8a)), 129.6 (Ar*C*(8)), 134.3 (Ar*C*(4a)), 137.8 (Ar*C*(1)), 140.4 (*C*(2)), 157.9 (Ar*C*(6)); HRMS (NSI$^+$) $C_{14}H_{14}O_2$ [M+Na]$^+$ found 237.0884, requires 237.0886 (–0.8 ppm).

1-(6-Methoxynaphthalen-2-yl)allyl isobutyrate S20

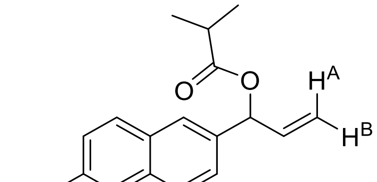

S20

Following General Procedure B, to a solution of DMAP (10 mol %), CH_2Cl_2 (0.2 M) and alcohol **34** (74 mg, 0.35 mmol) was added isobutyric anhydride (57 µL, 0.35 mmol) at room temperature and the reaction mixture was stirred for 16 h. The mixture gave a clean product (69 mg, 70%) as a yellow solid. mp 28–30 °C; ν_{max} (film) 1732 (C=O), 1607 (C=C); ^{1}H NMR (500 MHz, $CDCl_3$) δ_H: 1.18 (3H, d, *J* 7.0, CH(C*H₃*)), 1.21 (3H, d, *J* 7.0, CH(C*H₃*)), 2.63 (1H,

hept, *J* 7.0, C*H*(CH_3)$_2$), 3.92 (3H, s, OCH_3), 5.27 (1H, dt, *J* 10.5, 1.3, C(3)H^B), 5.33 (1H, dt, *J* 17.2, 1.3, C(3)H^A), 6.08 (1H, ddd, *J* 17.2, 10.5, 5.7, C(2)*H*), 6.39 (1H, d, *J* 5.7, C(1)*H*), 7.12 (1H, d, *J* 2.4, Ar(5)*H*), 7.15 (1H, dd, *J* 8.9, 2.4, Ar(7)*H*), 7.42 (1H, dd, *J* 8.4, 1.6, Ar(3)*H*), 7.73 (2H, d, *J* 8.6, Ar(4,8)*H*), 7.75–7.74 (1H, m, Ar(1)*H*); $^{13}C\{^1H\}$ NMR (126 MHz, $CDCl_3$) δ_C: 19.1 (CH(*C*H_3)), 19.1 (CH(*C*H_3)), 34.3 (*C*H(CH_3)$_2$), 55.5 (O*C*H_3), 76.0 (*C*(1)), 105.8 (Ar*C*), 116.8 (*C*(3)), 119.2 (Ar*C*), 125.6 (Ar*C*), 126.3 (Ar*C*), 127.3 (Ar*C*), 128.7 (Ar*C*), 129.7 (Ar*C*), 134.3 (Ar*C*), 134.4 (Ar*C*), 136.6 (*C*(2)), 158.1 (Ar*C*(6)), 176.2 (*C*=O); HRMS (NSI^+) $C_{18}H_{20}O_3Na$ $[M+Na]^+$ found 307.1305, requires 307.1305 (+0.1 ppm).

1-(6-Methoxynaphthalen-2-yl)prop-2-en-1-ol 34

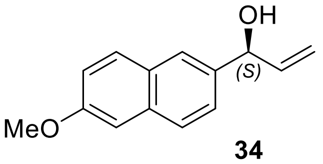

34

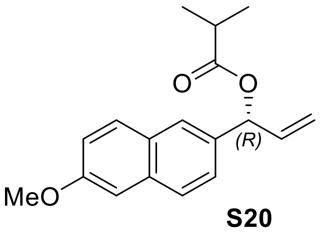

S20

Following General Procedure C, the alcohol **34** (73 mg, 0.34 mmol), HyperBTM **12** (106 µL from stock solution, 4 µmol, 1 mol %), *i*-Pr_2NEt (35 µL, 0.20 mmol) and isobutyric anhydride (27 µL, 0.17 mmol) were reacted in toluene (1.0 mL) for 16 h to give the crude product, which was purified via column chromatography (80:20 Petrol : EtOAc, R_f 0.20 (**34**) and R_f 0.45 (**S20**)) to separate alcohol **34** (22 mg, 0.10 mmol, 31%) and ester **S20** (31 mg, 0.13 mmol, 37%). **Alcohol 34:** $[\alpha]_D^{20}$ +0.5 (*c* 1.6, $CHCl_3$); Chiral HPLC analysis Chiralcel OJ-H (80:20 hexane : IPA, flow rate 1.0 mL min^{-1}, 211 nm, 30 °C) t_R (*S*): 23.7 min, t_R (*R*): 36.9 min, 44% ee. **Ester S20:** $[\alpha]_D^{20}$ +41.1 (*c* 1.5, $CHCl_3$); Chiral HPLC analysis Chiralpak AD-H (99:1 hexane : IPA, flow rate 1.0 mL min^{-1}, 220 nm, 30 °C) t_R (*R*): 9.5 min, t_R (*S*): 12.6 min, 50% ee. S = 5.

1-([1,1'-Biphenyl]-4-yl)prop-2-en-1-ol 35

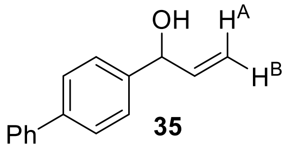

35

Following General Procedure A, vinylmagnesium bromide (4.7 mL of 0.7 M solution in THF, 3.29 mmol) added to a solution of 4-biphenylcarboxaldehyde (400 mg, 2.19 mmol) in anhydrous THF (30 mL) at –78 °C for 16 h gave, after purification by column chromatography (80:20 Petrol : EtOAc, R_f 0.29), the title compound **35** (137 mg, 30%) as a white solid with spectroscopic data in accordance with the literature.[4] mp 47–48 °C {Lit.[4]

55–56 °C}; ^{1}H NMR (400 MHz, $CDCl_3$) δ_H: 1.95 (1H, s, O*H*), 5.24 (1H, dt, *J* 10.3, 1.3, C(3)H^B), 5.27 (1H, d, *J* 5.9, C(1)*H*), 5.40 (1H, dt, *J* 17.1, 1.3, C(3)H^A), 6.10 (1H, ddd, *J* 17.1, 10.3, 5.9, C(2)*H*), 7.30–7.39 (1H, m, Ar*H*), 7.39–7.50 (4H, m, Ar*H*), 7.55–7.63 (4H, m, Ar*H*).

1-([1,1'-Biphenyl]-4-yl)allyl isobutyrate S21

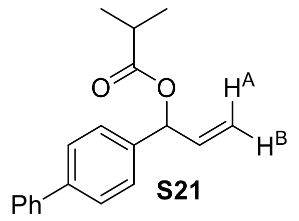

Following General Procedure B, to a solution of DMAP (10 mol %), CH_2Cl_2 (0.2 M) and alcohol **35** (43 mg, 0.20 mmol) was added isobutyric anhydride (34 µL, 0.20 mmol) at room temperature and the reaction mixture was stirred for 16 h. The mixture gave a clean product (45 mg, 81%) as a yellow oil. ν_{max} ($CHCl_3$) 1736 (C=O); ^{1}H NMR (500 MHz, $CDCl_3$) δ_H: 1.20 (3H, d, *J* 7.0, CH(CH_3)), 1.22 (3H, d, *J* 7.0, CH(CH_3)), 2.64 (1H, hept, *J* 7.0, C*H*(CH_3)$_2$), 5.27 (1H, dt, *J* 10.4, 1.3, C(3)H^B), 5.34 (1H, dt, *J* 17.1, 1.3, C(3)H^A), 6.04 (1H, ddd, *J* 17.1, 10.4, 5.9, C(2)*H*), 6.30 (1H, d, *J* 5.9, C(1)*H*), 7.32–7.38 (1H, m, Ar*H*), 7.40–7.47 (4H, m, Ar*H*), 7.55–7.61 (4H, m, Ar*H*); ^{13}C{^{1}H} NMR (126 MHz, $CDCl_3$) δ_C: 19.1 (CH(CH$_3$)), 19.1 (CH(CH$_3$)), 34.3 (CH(CH_3)$_2$), 75.7 (*C*(1)), 117.0 (*C*(3)), 127.3 (Ar*C*), 127.5 (Ar*C*), 127.5 (Ar*C*), 127.6 (Ar*C*), 128.9 (Ar*C*), 136.5 (Ar*C*), 138.3 (*C*(2)), 140.8 (Ar*C*), 141.1 (Ar*C*), 176.1 (*C*=O); HRMS (NSI$^+$) $C_{19}H_{20}O_2$ [M+Na]$^+$ found 303.1356, requires 303.1356 (+0.2 ppm).

Kinetic resolution of 1-([1,1'-biphenyl]-4-yl)prop-2-en-1-ol 35

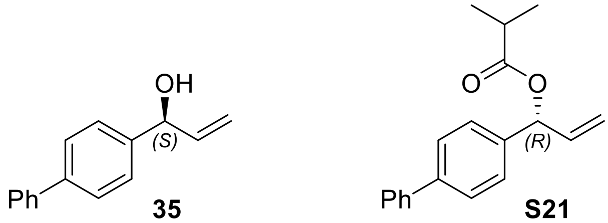

Following General Procedure C, the alcohol **35** (43 mg, 0.20 mmol), HyperBTM **12** (63 µL from stock solution, 2 µmol, 1 mol %), *i*-Pr_2NEt (21 µL, 0.12 mmol) and isobutyric anhydride (16 µL, 0.10 mmol) were reacted in toluene (0.6 mL) for 16 h to give the crude product, which was purified via column chromatography 80:20 Petrol : EtOAc, R_f 0.29 (**35**) and R_f 0.67 (**S21**)) to separate alcohol **35** (21 mg, 0.10 mmol, 50%) and ester **S21** (21 mg, 0.07 mmol, 37%). **Alcohol 35:** $[\alpha]_D^{20}$ +7.1 (*c* 1.0, $CHCl_3$); Chiral HPLC analysis Chiralcel OD-H (95:5 hexane : IPA, flow rate 1.0 mL min^{-1}, 254 nm, 30 °C) t_R (*R*): 13.6 min, t_R (*S*): 14.7

min, 56% ee. **Ester S21:** $[\alpha]_D^{20}$ +49.3 (*c* 1.0, $CHCl_3$); Chiral HPLC analysis Chiralcel OD-H (99:1 hexane : IPA, flow rate 1.0 mL min^{-1}, 254 nm, 30 °C) t_R (*R*): 5.4 min, t_R (*S*): 6.3 min, 76 % ee. S = 13.

1-(3-Vinylphenyl)prop-2-en-1-ol 36

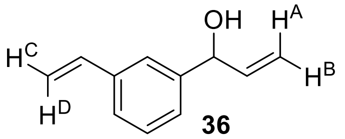

36

To a suspension of magnesium turnings (365 mg, 15 mmol) in anhydrous THF (15 mL) stirred under N_2 was added dropwise a solution of 4-bromostyrene (1.83 g, 12 mmol) in anhydrous THF. The reaction was left for 1 h stirring and before it was heated at reflux for 1 h and then cooled to rt. The product, 4-bromostyrylmagnesium bromide (20 mL of 0.4 M fresh-made solution in THF, 8 mmol) was added dropwise to a solution of acrolein (500 mg, 6.7 mmol) in anhydrous THF at –78 °C. The reaction was stirred at –78 °C for 3 h, before being quenched with NH_4Cl and extracted with EtOAc (× 3). The combined organic layers were washed with brine and dried over Na_2SO_4. After evaporation of volatiles, the crude product was purified by silica-gel column chromatography (80:20 Hexane : EtOAc, R_f 0.37), the title compound **36** (522 mg, 49%) as a colourless oil. ν_{max} (film) 3372 (O-H); ^{1}H NMR (500 MHz, $CDCl_3$) δ_H: 1.93 (1H, d, *J* 3.8, O*H*), 5.20–5.24 (2H, m, C(1)*H* and C(3)H^B), 5.26 (1H, dd, *J* 10.9, 0.7, Ar(3)C(2)H^C), 5.37 (1H, dt, *J* 17.0, 1.3, C(3)H^A), 5.77 (1H, dd, *J* 17.6, 0.7, Ar(3)C(2)H^D), 6.01–6.11 (1H, m, C(2)*H*), 6.72 (1H, dd, *J* 17.6, 10.9, Ar(3)C(1)*H*), 7.24–7.30 (1H, m, Ar*H*), 7.30–7.37 (2H, m, Ar*H*), 7.42 (1H, s, Ar(4)*H*); ^{13}C{^{1}H} NMR (126 MHz, $CDCl_3$) δ_C: 75.5 (*C*(1)), 114.3 (Ar(3)*C*(2)), 115.5 (*C*(3)), 124.3 (Ar*C*), 125.8 (Ar*C*), 125.9 (Ar*C*), 128.9 (Ar*C*(5)), 136.8 (Ar(3)*C*(1)), 138.0 (Ar*C*(3)), 140.2 (*C*(2)), 143.0 (Ar*C*(1)); HRMS (ASAP) $C_{11}H_{12}O$ [M-H] found 159.0810, requires 159.0810 (0.0 ppm).

1-(3-Vinylphenyl)allyl isobutyrate S22

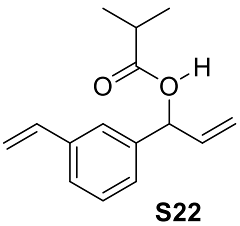

S22

Following General Procedure B, to a solution of DMAP (10 mol %), CH_2Cl_2 (0.2 M) and alcohol **36** (68 mg, 0.42 mmol) was added isobutyric anhydride (69 µL, 0.42 mmol) at room temperature and the reaction mixture was stirred for 16 h. The mixture gave a clean product

(67 mg, 68%) as a white gel. ν_{max} (film) 1732 (C=O); ^{1}H NMR (500 MHz, $CDCl_3$) δ_H: 1.18 (3H, d, *J* 7.0, CH(C*H*$_3$)), 1.21 (2H, d, *J* 7.0, CH(C*H*$_3$)), 2.62 (1H, hept, *J* 7.0, C*H*(CH_3)$_2$), 5.22–5.28 (2H, m, C(3)*H*$_2$), 5.30 (1H, dt, *J* 17.2, 1.3, Ar(3)C(2)*H*), 5.75 (1H, d, *J* 17.6, C(2)*H*), 5.96–6.04 (1H, m, Ar(3)C(2)*H*), 6.25 (1H, d, *J* 5.8, C(1)*H*), 6.67–6.76 (1H, m, Ar(3)C(1)*H*), 7.24 (1H, d, *J* 7.4, Ar*H*), 7.31 (1H, t, *J* 7.5, Ar*H*), 7.33–7.39 (2H, m, Ar*H*); ^{13}C{^{1}H} NMR (126 MHz, $CDCl_3$) δ_C: 19.1 (CH(*C*H_3)), 19.1 (CH(*C*H_3)), 34.3 (*C*H(CH_3)$_2$), 75.8 (C(1)), 114.4 (Ar(3)*C*(2)), 117.0 (*C*(3)), 125.0 (Ar*C*), 126.0 (Ar*C*), 126.6 (Ar*C*), 128.9 (Ar*C*), 136.5 (Ar*C*), 136.7 (Ar*C*), 138.0 (Ar(3)*C*(1)), 139.6 (*C*(2)), 176.1 (*C*=O); HRMS could not be obtained due to significant fragmentation.

Kinetic resolution of 1-(3-vinylphenyl)prop-2-en-1-ol 36

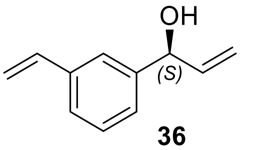

36

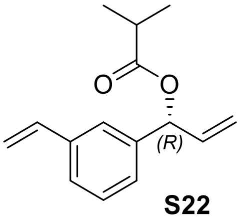

S22

Following General Procedure C, the alcohol **36** (76 mg, 0.47 mmol), HyperBTM **12** (148 µL from stock solution, 5 µmol, 1 mol %), *i*-Pr_2NEt (50 µL, 0.28 mmol) and isobutyric anhydride (39 µL, 0.22 mmol) were reacted in toluene (1.3 mL) for 16 h to give the crude product, which was purified via column chromatography (70:30 Petrol : EtOAc, R_f 0.33 (**36**) and R_f 0.67 (**S22**)) to separate alcohol **36** (25 mg, 0.16 mmol, 34%) and ester **S22** (32 mg, 0.14 mmol, 29%). **Alcohol 36:** $[\alpha]_D^{20}$ –1.4 (*c* 1.0, $CHCl_3$); Chiral HPLC analysis Chiralcel OJ-H (80:20 hexane : IPA, flow rate 1.0 mL min^{-1}, 211 nm, 30 °C) t_R (*S*): 8.5 min, t_R (*R*): 9.6 min, 77% ee. **Ester S22:** $[\alpha]_D^{20}$ +22.3 (*c* 1.4, $CHCl_3$); Chiral HPLC analysis Chiralcel OJ-H (90:10 hexane : IPA, flow rate 1.0 mL min^{-1}, 211 nm, 30 °C) t_R (*R*): 4.4 min, t_R (*S*): 4.9 min, 83% ee. S = 26.

1-(Pyridin-2-yl)prop-2-en-1-ol 38

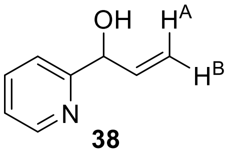

38

Following General Procedure A, vinylmagnesium bromide (10.0 mL of 0.7 M solution in THF, 7.0 mmol) added to a solution of 2-pyridinecarboxaldehyde (500 mg, 4.67 mmol) in anhydrous THF (30 mL) at –78 °C for 16 h gave, after purification by column chromatography (50:50 Hexane : EtOAc, R_f 0.20), the title compound **38** (288 mg, 46%) as a

yellow oil with spectroscopic data in accordance with the literature.[18] ^{1}H NMR (500 MHz, $CDCl_3$) δ_H: 4.68 (1H, s, O*H*), 5.18 (1H, d, *J* 6.8, C(1)*H*), 5.25 (1H, dt, *J* 10.2, 1.3, C(3)H^{B}), 5.47 (1H, dt, *J* 17.1, 1.3, C(3)H^{A}), 5.96 (1H, ddd, *J* 17.0, 10.2, 6.8, C(2)*H*), 7.19–7.24 (1H, m, Ar(4)*H*), 7.27–7.32 (1H, m, Ar(6)*H*), 7.70 (1H, td, *J* 7.7, 1.7, Ar(5)*H*), 8.55 (1H, d, *J* 4.7, Ar(3)*H*).

1-(Pyridin-2-yl)allyl isobutyrate S23

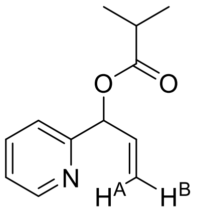

S23

Following General Procedure B, to a solution of DMAP (10 mol %), CH_2Cl_2 (0.2 M) and alcohol **38** (68 mg, 0.50 mmol) was added isobutyric anhydride (82 µL, 0.50 mmol) at room temperature and the reaction mixture was stirred for 16 h. The mixture gave a clean product (78 mg, 76%) as a yellow oil. ν_{max} ($CHCl_3$) 1734 (C=O); ^{1}H NMR (400 MHz, $CDCl_3$) δ_H: 1.21 (3H, d, *J* 7.0, CH(CH_3)), 1.23 (3H, d, *J* 7.0, CH(CH_3)), 2.68 (1H, hept, *J* 7.0, C*H*(CH_3)$_2$), 5.29 (1H, dt, *J* 10.4, 1.3, C(3)H^{B}), 5.39 (1H, dt, *J* 17.2, 1.3, C(3)H^{A}), 6.11 (1H, ddd, *J* 17.2, 10.4, 6.1, C(2)*H*), 6.31 (1H, d, *J* 6.1, C(1)*H*), 7.21 (1H, ddd, *J* 7.5, 4.9, 1.1, Ar*H*), 7.35 (1H, d, *J* 7.9, Ar*H*), 7.69 (1H, td, *J* 7.7, 1.8, Ar*H*), 8.60 (1H, ddd, *J* 4.8, 1.8, 0.9, ArC(6)*H*); ^{13}C{^{1}H} NMR (126 MHz, $CDCl_3$) δ_C: 19.1 (CH(*C*H_3)), 19.1 (CH(*C*H_3)), 34.2 (*C*H(CH_3)$_2$), 78.8 (*C*(1)), 117.7 (*C*(3)), 121.1 (Ar*C*), 122.9 (Ar*C*), 135.4 (Ar*C*), 137.0 (*C*(2)), 149.6 (Ar*C*(6)), 158.6 (Ar*C*(2)), 176.0 (*C*=O); HRMS (NSI^{+}) $C_{12}H_{16}NO_2$ $[M+H]^{+}$ found 206.1174, requires 206.1176 (–0.8 ppm).

Kinetic resolution of 1-(pyridin-2-yl)prop-2-en-1-ol 38

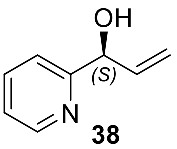

38

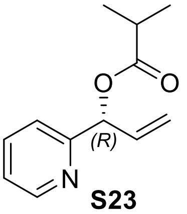

S23

Following General Procedure C, the alcohol **38** (109 mg, 0.81 mmol), HyperBTM **12** (252 µL from stock solution, 8 µmol, 1 mol %), *i*-Pr_2NEt (84 µL, 0.48 mmol) and isobutyric anhydride (65 µL, 0.40 mmol) were reacted in toluene (1.8 mL) for 16 h to give the crude

product, which was purified via column chromatography 50:50 Petrol : EtOAc, R_f 0.24 (**38**) and R_f 0.51 (**S23**)) to separate alcohol **38** (46 mg, 0.34 mmol, 42%) and ester **S23** (75 mg, 0.37 mmol, 45%). **Alcohol 38:** $[\alpha]_D^{20}$ +5.6 (*c* 1.0, $CHCl_3$); Chiral HPLC analysis Chiralcel OD-H (99:1 hexane : IPA, flow rate 1.0 mL min^{-1}, 254 nm, 30 °C) t_R (*R*): 17.4 min, t_R (*S*): 21.1 min, 34% ee. **Ester S23:** $[\alpha]_D^{20}$ +3.5 (*c* 1.0, $CHCl_3$); Chiral HPLC analysis Chiralcel OD-H (99:1 hexane : IPA, flow rate 1.0 mL min^{-1}, 254 nm, 30 °C) t_R (*R*): 8.0 min, t_R (*S*): 9.4 min, 36% ee. S = 3.

1-(Pyridin-3-yl)prop-2-en-1-ol 39

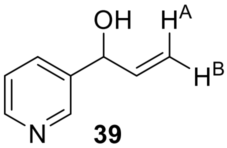

39

Following General Procedure A, vinylmagnesium bromide (16.0 mL of 0.7 M solution in THF, 11.0 mmol) added to a solution of 3-pyridinecarboxaldehyde (800 mg, 7.47 mmol) in anhydrous THF (40 mL) at 0 °C for 16 h gave, after purification by column chromatography (80:20 Petrol : EtOAc, R_f 0.22), the title compound **39** (541 mg, 54%) as a yellow oil with spectroscopic data in accordance with the literature.[19] ^{1}H NMR (500 MHz, $CDCl_3$) δ_H: 5.23–5.27 (2H, m, C(3)H^B and O*H*), 5.38 (1H, d, *J* 17.1, C(3)H^A), 6.03 (1H, ddd, *J* 17.1, 10.4, 6.0, C(2)*H*), 7.28 (1H, dd, *J* 7.8, 4.9, Ar(5)*H*), 7.72 (1H, dt, *J* 7.9, 1.7, Ar(6)*H*), 8.45–8.52 (1H, m, Ar(4)*H*), 8.56 (1H, d, *J* 1.7, Ar(2)*H*).

1-(Pyridin-3-yl)allyl isobutyrate S24

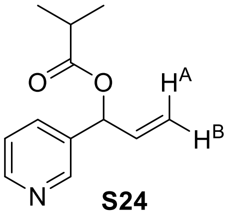

S24

Following General Procedure B, to a solution of DMAP (10 mol %), CH_2Cl_2 (0.2 M) and alcohol **39** (92 mg, 0.68 mmol) was added isobutyric anhydride (110 µL, 0.68 mmol) at room temperature and the reaction mixture was stirred for 16 h. The mixture gave a clean product (131 mg, 94%) as a colourless oil. ν_{max} ($CHCl_3$) 1736 (C=O); ^{1}H NMR (400 MHz, $CDCl_3$) δ_H: 1.17 (3H, d, *J* 7.0, CH(CH_3)), 1.20 (3H, d, *J* 7.0, CH(CH_3)), 2.62 (1H, hept, *J* 7.0, C*H*(CH_3)$_2$), 5.29 (1H, dt, *J* 5.3, 1.2, C(3)H^B), 5.33 (1H, dt, *J* 12.0, 1.2, C(3)H^A), 5.99 (1H, ddd, *J* 17.1, 10.5, 5.8, C(2)*H*), 6.28 (1H, d, *J* 5.8, C(1)*H*), 7.29 (1H, ddd, *J* 7.9, 4.8, 0.8, Ar(5)*H*), 7.66 (1H, dt, *J* 7.9, 1.8, Ar(6)*H*), 8.56 (1H, dd, *J* 4.8, 1.6, Ar(4)*H*), 8.62 (1H, d, *J*

2.2, Ar(2)*H*); $^{13}C\{^1H\}$ NMR (101 MHz, $CDCl_3$) δ_C: 19.0 (CH(CH$_3$)$_2$), 34.3(C(CH$_3$)$_2$), 73.8 (C(1)), 117.9 (C(2)), 123.6 (ArC), 134.8 (ArC), 135.6 (C(2)), 148.9 (ArC), 149.6 (ArC), 175.9 (C=O); HRMS (NSI^+) $C_{12}H_{15}NO_2$ $[M+H]^+$ found 206.1172, requires 206.1176 (+1.7 ppm).

Kinetic resolution of 1-(pyridin-3-yl)prop-2-en-1-ol 39

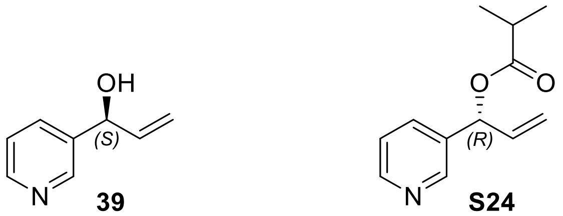

39 **S24**

Following General Procedure C, the alcohol **39** (127 mg, 0.94 mmol), HyperBTM **12** (293 µL from stock solution, 9 µmol, 1 mol %), *i*-Pr_2NEt (98 µL, 0.56 mmol) and isobutyric anhydride (76 µL, 0.47 mmol) were reacted in toluene (1.3 mL) for 16 h to give the crude product, which was purified via column chromatography (20:80 Petrol : EtOAc, R_f 0.25 (**39**) and R_f 0.69 (**S24**)) to separate alcohol **39** (50 mg, 0.37 mmol, 40%) and ester **S24** (88 mg, 0.43 mmol, 46%). **Alcohol 39:** $[\alpha]_D^{20}$ +3.4 (*c* 1.0, $CHCl_3$); Chiral HPLC analysis Chiralcel OJ-H (90:10 hexane : IPA, flow rate 1.0 mL min^{-1}, 211 nm, 30 °C) t_R (*S*): 7.7 min, t_R (*R*): 9.1 min, 38% ee. **Ester S24:** $[\alpha]_D^{20}$ +21.8 (*c* 1.0, $CHCl_3$); Chiral HPLC analysis Chiralcel OJ-H (99:1 hexane : IPA, flow rate 1.0 mL min^{-1}, 254 nm, 30 °C) t_R (*R*): 8.4 min, t_R (*S*): 9.6 min, 46% ee. S = 4.

1-(Thiophen-2-yl)prop-2-en-1-ol 40

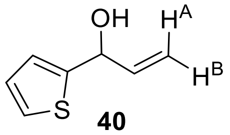

40

A stirring solution of thiophene (1.0 g, 11.9 mmol) in anhydrous THF was cooled to –15 °C, and *n*-BuLi (5.7 mL of 2.5 M solution in hexanes, 14.3 mmol) was added. After being stirred for 1 h at 0 °C, the acrolein (0.8 mL, 11.9 mmol) was added dropwise and the reaction mixture was stirred overnight. The mixture was quenched with NH_4Cl and it was extracted with EtOAc (×3) and the combined organic layers were washed with brine and dried over Na_2SO_4. After evaporation of volatiles, the crude product was purified by silica-gel column chromatography (70:30 Petrol : Et_2O, R_f 0.37), the title compound **40** (1.43 g, 86%) as a yellowish oil with spectroscopic data in accordance with the literature.[20] ^1H NMR (400 MHz, $CDCl_3$) δ_H: 2.06 (1H, s, O*H*), 5.26 (1H, dt, *J* 10.3, 1.2, C(3)H^B), 5.42 (1H, dt, *J* 17.1,

1.3, C(3)H^A), 5.46 (1H, br s, C(1)*H*), 6.14 (1H, ddd, *J* 17.1, 10.3, 6.0, C(2)*H*), 6.95–7.03 (2H, m, Ar*H*), 7.28 (1H, dd, *J* 4.8, 1.5, Ar*H*).

1-(Thiophen-2-yl)allyl isobutyrate S25

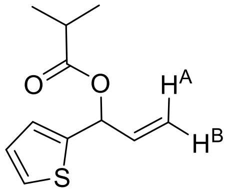

S25

Following General Procedure B, to a solution of DMAP (10 mol %), CH_2Cl_2 (0.2 M) and alcohol **40** (78 mg, 0.55 mmol) was added isobutyric anhydride (90 µL, 0.55 mmol) at room temperature and the reaction mixture was stirred for 16 h. The mixture gave a clean product (64 mg, 56%) as a yellowish oil. ν_{max} ($CHCl_3$) 1732 (C=O); ^{1}H NMR (500 MHz, $CDCl_3$) δ_H: 1.17 (3H, d, *J* 7.0, CH(CH_3)), 1.19 (3H, d, *J* 7.0, CH(CH_3)), 2.60 (1H, hept, *J* 7.0, C*H*(CH_3)), 5.30 (1H, d, *J* 10.4, C(3)H^B), 5.40 (1H, d, *J* 17.1, C(3)H^A), 6.08 (1H, ddd, *J* 17.1, 10.4, 5.9, C(2)*H*), 6.52 (1H, d, *J* 5.9, C(1)*H*), 6.98 (1H, dd, *J* 5.0, 3.6, Ar*H*), 7.04 (1H, d, *J* 3.4, Ar*H*), 7.29 (1H, dd, *J* 5.0, 1.1, Ar*H*); ^{13}C{^{1}H} NMR (126 MHz, $CDCl_3$) δ_C: 19.0 (CH(*C*H_3)$_2$), 34.3 (*C*H(CH_3)$_2$), 71.3 (*C*(1)), 117.4 (*C*(3)), 126.0 (Ar*C*(5)), 126.2 (Ar*C*(4)), 126.8 (Ar*C*(3)), 135.7 (*C*(2)), 142.3 (Ar*C*(1)), 176.0 (*C*=O); HRMS could not be obtained due to significant fragmentation.

Kinetic resolution of 1-(thiophen-2-yl)prop-2-en-1-ol 40

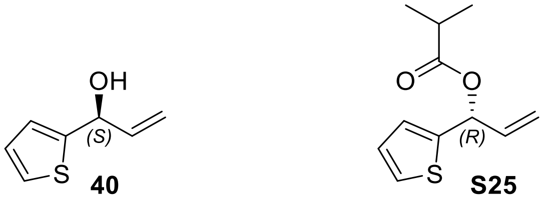

Following General Procedure C, the alcohol **40** (53 mg, 0.37 mmol), HyperBTM **12** (117 µL from stock solution, 4 µmol, 1 mol %), *i*-Pr_2NEt (39 µL, 0.22 mmol) and isobutyric anhydride (30 µL, 0.19 mmol) were reacted in toluene (1.0 mL) for 16 h to give the crude product, which was purified via column chromatography 70:30 Petrol : Et_2O, R_f 0.37 (**40**) and R_f 0.69 (**S25**)) to separate alcohol **40** (58 mg, 0.41 mmol, 49%) and ester **S25** (78 mg, 0.37 mmol, 44%). **Alcohol 40:** $[\alpha]_D^{20}$ +64.8 (*c* 0.5, $CHCl_3$); Chiral HPLC analysis Chiralcel OJ-H (99:1 hexane : IPA, flow rate 1.0 mL min^{-1}, 254 nm, 30 °C) t_R (*S*): 37.7 min, t_R (*R*): 43.7 min, 51% ee. **Ester S25:** $[\alpha]_D^{20}$ +2.5 (*c* 1.0, $CHCl_3$); Chiral HPLC analysis Chiralcel AD-H (99.8:0.2 hexane : IPA, flow rate 0.5 mL min^{-1}, 220 nm, 30 °C) t_R (*R*): 11.1 min, t_R (*S*): 12.1

min, 68% ee. S = 9.

1-(4-Methoxyphenyl)-2-methylprop-2-en-1-ol 41

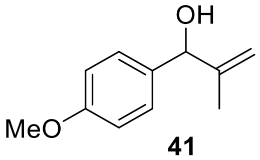

41

Following General Procedure A, isopropylmagnesium bromide (22 mL of 0.5 M solution in THF, 11 mmol) added to a solution of anisaldehyde (1.0 g, 73 mmol) in anhydrous THF (100 mL) at 0 °C for 3 h gave, after purification by column chromatography (80:20 Petrol : EtOAc, R_f 0.32), the title compound **41** (1.30 g, 99%) as a yellow oil with spectroscopic data in accordance with the literature.[1] ^{1}H NMR (500 MHz, $CDCl_3$) δ_H: ^{1}H NMR (500 MHz, CDCl3) δ_H: 1.60 (3H, s, C*H*$_3$), 3.81 (3H, s, OC*H*$_3$), 4.91–4.99 (1H, m, C(3)*H*), 5.08 (1H, s, C(3)*H*), 5.20 (1H, s, C(1)*H*), 6.86–6.90 (2H, m, Ar(3,5)*H*), 7.27–7.31 (2H, m, Ar(2,6)*H*).

1-(4-Methoxyphenyl)-2-methylallyl isobutyrate S26

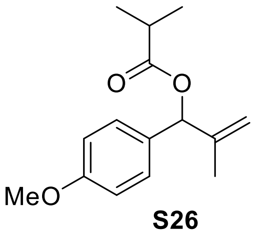

S26

Following General Procedure B, to a solution of DMAP (10 mol %), CH_2Cl_2 (0.1 M) and alcohol **41** (60 mg, 0.34 mmol) was added isobutyric anhydride (56 µL, 0.36 mmol) at room temperature and the reaction mixture was stirred for 16 h. The mixture gave a clean product (no purification) as a yellowish oil (65 mg, 78%). ν_{max} (film) 1734 (C=O); ^{1}H NMR (400 MHz, $CDCl_3$) δ_H: 1.17 (3H, d, *J* 7.0, CH(C*H*$_3$)), 1.20 (3H, d, *J* 7.0, CH(C*H*$_3$)), 1.64 (3H, s, C(2)C*H*$_3$), 2.61 (1H, hept, *J* 7.0, C*H*(CH_3)$_2$), 3.80 (1H, s, OC*H*$_3$), 4.90–4.97 (1H, m, C(3)*H*), 5.08 (1H, s, C(3)*H*), 6.09 (1H, s, C(1)*H*), 6.85–6.87 (1H, m, Ar*H*), 6.87–6.90 (1H, m, Ar*H*), 7.26–7.33 (2H, m, ArC(2,6)*H*); ^{13}C{^{1}H} NMR (126 MHz, $CDCl_3$) δ_C: 18.9 (C(2)*C*H_3), 19.0 (CH(*C*H_3)), 19.1 (CH(*C*H_3)), 34.2 (*C*H(CH_3)$_2$), 55.3 (O*C*H_3), 77.6 (*C*(1)), 111.8 (*C*(3)), 113.7 (Ar*C*), 113.8 (Ar*C*), 128.5 (Ar*C*(1)), 130.7 (Ar*C*(2,6)), 143.5 (*C*(2)), 159.3 (Ar*C*(4)), 175.9 (*C*=O); HRMS (NSI$^+$) $C_{15}H_{20}O_3$ [M+Na]$^+$ found 271.1306, requires 271.1305 (+0.5 ppm).

Kinetic resolution of 1-(4-methoxyphenyl)-2-methylprop-2-en-1-ol 41

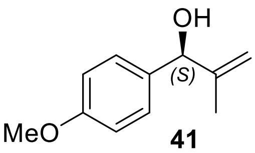

41

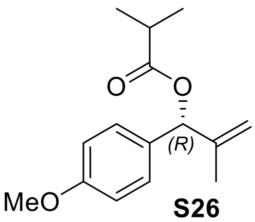

S26

Following General Procedure C, the alcohol **41** (40 mg, 0.23 mmol), HyperBTM **12** (70 µL from stock solution, 3 µmol, 1 mol %), *i*-Pr_2NEt (24 µL, 0.14 mmol) and isobutyric anhydride (22 µL, 0.14 mmol) were reacted in toluene (0.7 mL) for 16 h to give the crude product, which was purified via column chromatography (80:20 Petrol : EtOAc, R_f 0.32 (**41**) and R_f 0.66 (**S26**)) to separate alcohol **41** (19 mg, 0.11 mmol, 48%) and ester **S26** (24 mg, 0.10 mmol, 42%). **Alcohol 41:** $[\alpha]^{20}_{D}$ –24.0 (*c* 0.5, $CHCl_3$); Chiral HPLC analysis Chiralcel OD-H (95:5 hexane : IPA, flow rate 1.0 mL min^{-1}, 211 nm, 30 °C) t_R (*R*): 10.3 min, t_R (*S*): 12.0 min, 58% ee. **Ester S26:** $[\alpha]^{20}_{D}$ +60.1 (*c* 1.0, $CHCl_3$); Chiral HPLC analysis Chiralcel OJ-H (99:1 hexane : IPA, flow rate 1.0 mL min^{-1}, 211 nm, 30 °C) t_R (*R*): 7.4 min, t_R (*S*): 12.2 min, 67 % ee. S = 10.

(*E*)-1-(4-Methoxyphenyl)but-2-en-1-ol 42

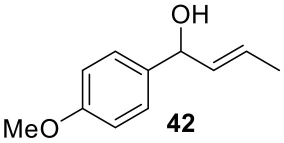

42

Following General Procedure A, 4-methoxyphenylmagnesium bromide (11 mL of 0.5 M solution in THF, 8.91 mmol) added to a solution of trans-crotonaldehyde (250 mg, 3.57 mmol) in anhydrous THF (15 mL) at 0°C for 3 h gave, after purification by column chromatography (80:20 Hexane : EtOAc, R_f 0.26), the title compound **42** (400 mg, 63%) as a yellow oil with spectroscopic data in accordance with the literature.[21] ^{1}H NMR (500 MHz, $CDCl_3$) δ_H: 1.72 (3H, d, *J* 5.9, C(4)H_3), 1.79 (1H, d, *J* 3.4, O*H*), 3.80 (4H, s, OCH_3), 5.09–5.15 (1H, m, C(1)*H*), 5.65–5.79 (2H, m, C(2,3)*H*), 6.83–6.94 (2H, m, Ar(3,5)*H*), 7.27–7.36 (2H, m, Ar(2,6)*H*).

(*E*)-1-(4-Methoxyphenyl)but-2-en-1-yl isobutyrate S27

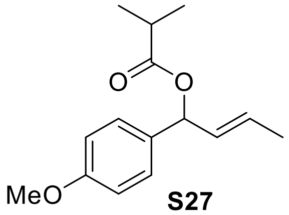

S27

Following General Procedure B, to a solution of DMAP (10 mol %), CH_2Cl_2 (0.1 M) and alcohol **42** (118 mg, 0.23 mmol) was added isobutyric anhydride (37 µL, 0.66 mmol) at room temperature and the reaction mixture was stirred for 16 h. The mixture gave a clean product (no purification) as a coulorless oil (104 mg, 63%). ^{1}H NMR (400 MHz, $CDCl_3$) δ_H 1.20 (6H, dd, *J* 7.0, 3.1, CH(C*H*$_3$)$_2$), 1.41 (3H, d, *J* 6.5, C(4)*H*$_3$), 2.57 (1H, hept, *J* 7.0, C*H*(CH_3)$_2$), 3.83 (3H, s, OC*H*$_3$), 5.53 (1H, pd, *J* 6.5, 1.1, C(1)*H*), 6.08 (1H, dd, *J* 15.9, 6.8, C(2)*H*), 6.57 (1H, d, *J* 15.9, C(3)*H*), 6.84–6.91 (2H, m, Ar(3,5)*H*), 7.31–7.38 (2H, m, Ar(2,6)*H*); ^{13}C{^{1}H} NMR (101 MHz, $CDCl_3$) δ_C: 19.1 (CH(*C*H_3)), 19.2 (CH(*C*H_3)), 20.6 (*C*(4)), 34.4 (*C*H(CH_3)$_2$), 55.4 (O*C*H_3), 70.9 (*C*(1)), 114.1 (Ar*C*(2,5)), 126.9 (*C*(3)), 127.9 (Ar*C*(2,6)), 129.3 (Ar*C*(1)), 131.1 (*C*(2)), 159.5 (Ar*C*(4)), 176.6 (*C*=O); HRMS (NSI^{+}) $C_{15}H_{20}O_3$ [M+Na]$^{+}$ found 271.1306, requires 271.1305 (+0.5 ppm).

Kinetic resolution of (*E*)-1-(4-methoxyphenyl)but-2-en-1-ol 42

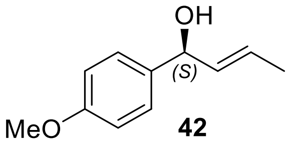

42

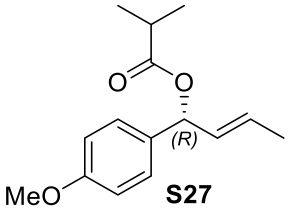

S27

The alcohol **42** (53 mg, 0.30 mmol), HyperBTM **12** (93 µL from stock solution, 3 µmol, 1 mol %), *i*-Pr_2NEt (31 µL, 0.18 mmol) and isobutyric anhydride (24 µL, 0.15 mmol) were reacted in toluene (0.8 mL) at 0°C. The solution was stirred for 16 h before being quenched with brine (The reaction is very sentive to the use of acids). The crude product was purified via column chromatography (80:20 Petrol : EtOAc, R_f 0.35 (**42**) and R_f 0.80 (**S27**)) to separate alcohol **42** (53 mg, 0.30 mmol, 59%) and ester **S27** (38 mg, 0.15 mmol, 30%). **Alcohol 42:** $[\alpha]_D^{20}$ +19.4 (*c* 0.5, $CHCl_3$); Chiral HPLC analysis Chiralcel OD-H (95:5 hexane : IPA, flow rate 1.0 mL min^{-1}, 211 nm, 30 °C) t_R (*R*): 11.2 min, t_R (*S*): 13.6 min, 46% ee. **Ester S27:** $[\alpha]_D^{20}$ +14.0 (*c* 1.0, $CHCl_3$); Chiral HPLC analysis Chiralcel OJ-H (99:1 hexane : IPA, flow rate 1.0 mL min^{-1}, 254 nm, 30 °C) t_R (*S*): 18.9 min, t_R (*R*): 21.9 min, 30% ee. S could not be calculated as more than two products were observed by TLC.

(*E*)-1-(4-Methoxyphenyl)-2-methylbut-2-en-1-ol 43

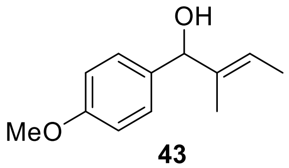

43

Following General Procedure A, 4-methoxyphenylmagnesium bromide (18 mL of 0.5 M solution in THF, 8.91 mmol) added to a solution of (*E*)-2-methyl-2-butenal (0.5 g, 5.94 mmol) in anhydrous THF (15 mL) at 0 °C for 3 h gave, after purification by column chromatography (80:20 Hexane : EtOAc, R_f 0.38), the title compound **43** (334 mg, 30%) as a colourless oil. ν_{max} (film) 3414 (O-H), 1610 (C=C); ^{1}H NMR (400 MHz, $CDCl_3$) δ_H: 1.45–1.51 (3H, m, C(2)C*H*3), 1.66 (3H, d, *J* 6.7, C(3)C*H*3), 1.75–1.78 (2H, m, O*H*), 3.80 (3H, s, OC*H*3), 5.09 (1H, s, C(1)*H*), 5.66–5.75 (2H, m, ArC(3,5)*H*), 6.84–6.90 (2H, m, ArC(2,6)*H*); ^{13}C{^{1}H} NMR (126 MHz, $CDCl_3$) δ_C: 12.0 (C(2)*C*H3), 13.3 (C(3)*C*H3), 55.4 (O*C*H3), 79.0 (*C*(1)), 113.7 (Ar*C*(3,5)), 120.8 (*C*(3)), 127.6 (Ar*C*(2,6)), 134.8 (Ar*C*(1)), 137.8 (*C*(2)), 159.0 (Ar*C*(4)); HRMS (ASAP) $C_{12}H_{16}O_2$ [M-H] found 191.1076, requires 191.1072 (+2.1 ppm).

(*E*)-1-(4-Methoxyphenyl)-2-methylbut-2-en-1-yl isobutyrate S28

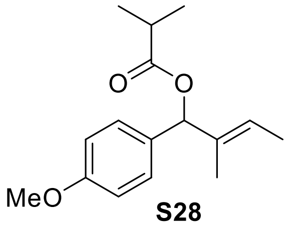

S28

Following General Procedure B, to a solution of DMAP (10 mol %), CH_2Cl_2 (0.1 M) and alcohol **43** (43 mg, 0.23 mmol) was added isobutyric anhydride (37 µL, 0.23 mmol) at room temperature and the reaction mixture was stirred for 16 h. The mixture gave a clean product (no purification) as a yellowish oil (43 mg, 72%). ν_{max} (film) 1732 (C=O); ^{1}H NMR (400 MHz, $CDCl_3$) δ_H: 1.19 (6H, t, *J* 7.1, CH(C*H*3)2), 1.53 (3H, s, C(2)C*H*3), 1.64 (3H, d, *J* 6.7, C(3)C*H*3), 2.60 (1H, dq, *J* 14.0, 7.0, C(2)*H*), 3.80 (3H, s, OC*H*3), 5.62 (1H, q, *J* 6.8, C(3)*H*), 6.12 (1H, s, C(1)*H*), 6.86 (2H, d, *J* 8.7, Ar(3,5)*H*), 7.22 (2H, d, *J* 8.7, Ar(2,6)*H*); ^{13}C{^{1}H} NMR (101 MHz, $CDCl_3$) δ_C: 12.6 (C(2)*C*H3), 13.4 (C(3)*C*H3), 19.1 (CH(*C*H3)), 19.2 (CH(*C*H3)), 34.5(*C*(CH3)2) , 55.4 (O*C*H3), 79.4 (*C*(1)), 113.8 (Ar*C*(3,5)), 122.4 (*C*(3)), 128.1 (Ar*C*(2,6)), 131.7 (Ar*C*(1)), 134.4 (*C*(2)), 159.1 (Ar*C*(4)), 176.1 (*C*=O); HRMS (NSI^{+}) $C_{16}H_{22}O_3$ [M-H] found 285.1460, requires 285.1461 (–0.4 ppm).

Kinetic resolution of (*E*)-1-(4-methoxyphenyl)-2-methylbut-2-en-1-ol 43

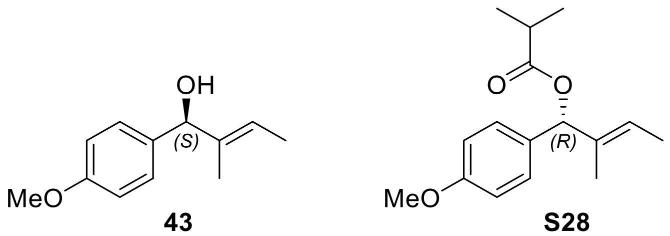

43 **S28**

Following General Procedure C, the alcohol **43**(87 mg, 0.45 mmol), HyperBTM **12** (142 µL from stock solution, 5 µmol, 1 mol%), *i*-Pr_2NEt (47 µL, 0.27 mmol) and isobutyric anhydride (40 µL, 0..24 mmol) were reacted in toluene (1.0 mL) for 16 h to give the crude product, which was purified via column chromatography (80:20 Petrol : EtOAc, R_f 0.38 (**43**) and R_f 0.66 (**S28**)) to separate alcohol **43** (56 mg, 0.21 mmol, 64%) and ester **S28** (41 mg, 0.15 mmol, 34%). **Alcohol 43:** $[\alpha]_D^{20}$ +5.8 (*c* 1.0, $CHCl_3$); Chiral HPLC analysis Chiralcel OD-H (95:5 hexane : IPA, flow rate 1.0 mL min^{-1}, 211 nm, 30 °C) t_R (*R*): 10.6 min, t_R (*S*): 12.6 min, 24% ee. **Ester S28:** $[\alpha]_D^{20}$ +7.8 (*c* 1.0, $CHCl_3$); Chiral HPLC analysis Chiralcel OJ-H (99:1 hexane : IPA, flow rate 1.0 mL min^{-1}, 220 nm, 30 °C) t_R (*R*): 6.7 min, t_R (*S*): 8.5 min, 40% ee. S = 3.

2-Methyl-1-(naphthalen-2-yl)prop-2-en-1-ol 44

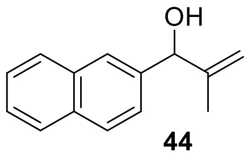

44

Following General Procedure A, isopropenylmagnesium bromide (1.5 mL of 0.5 M solution in THF, 4.80 mmol) added to a solution of 2-naphthaldehyde (500 mg, 3.21 mmol) in anhydrous THF (20 mL) at –78 °C for 16 h gave, after purification by column chromatography (70:30 Petrol : EtOAc, R_f 0.46), the title compound **44** (555 mg, 87%) as a white solid with spectroscopic data in accordance with the literature.[22] mp 49–50 °C; ^{1}H NMR (500 MHz, $CDCl_3$) δ_H: 1.64 (3H, s, C*H*$_3$), 2.04 (1H, d, *J* 3.0, O*H*), 5.01 (1H, s, C(1)*H*), 5.27–5.34 (2H, m, C(3)*H*$_2$), 7.45–7.51 (3H, m, Ar*H*), 7.80–7.88 (4H, m, Ar*H*); ^{13}C{^{1}H} NMR (126 MHz, $CDCl_3$) δ_C 18.4 (*C*H$_3$), 78.1 (*C*(1)) , 111.7 (*C*(3)), 124.6 (Ar*C*) , 125.4 (Ar*C*), 126.1 (Ar*C*), 126.3 (Ar*C*), 127.8 (Ar*C*), 128.1 (Ar*C*), 128.3 (Ar*C*), 133.1 (Ar*C*(9)), 133.4 (Ar*C*(4)), 139.4 (Ar*C*(2)), 146.8 (*C*(2)).

2-Methyl-1-(naphthalen-2-yl)allyl isobutyrate S29

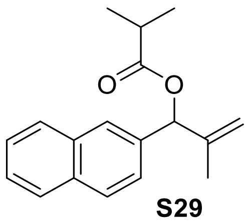

Following General Procedure B, to a solution of DMAP (10 mol %), CH_2Cl_2 (0.2 M) and alcohol **44** (96 mg, 0.48 mmol) was added isobutyric anhydride (79 µL, 0.48 mmol) at room temperature and the reaction mixture was stirred for 16 h. The mixture gave a clean product (92 mg, 71%) as a colourless oil. ν_{max} (film) 1732 (C=O); ^{1}H NMR (500 MHz, $CDCl_3$) δ_H: 1.20 (3H, d, *J* 7.0, CH(C*H₃*)), 1.24 (3H, d, *J* 7.0, CH(C*H₃*)), 1.68 (3H, s, C(2)C*H₃*), 2.67 (1H, hept, *J* 7.0, C*H*(CH_3)$_2$), 5.00–5.06 (1H, m, C(3)*H*), 5.18 (1H, s, C(3)*H*), 6.32 (1H, s, C(1)*H*), 7.43–7.53 (3H, m, Ar*H*), 7.78–7.87 (4H, m, Ar*H*); ^{13}C{^{1}H} NMR (126 MHz, $CDCl_3$) δ_C: 19.1 (CH(*C*H_3)), 19.1 (CH(*C*H_3)), 19.2 (C(2)*C*H_3), 34.4 (*C*H(CH_3)$_2$), 78.3 (*C*(1)), 112.8 (*C*(3)), 124.9 (Ar*C*), 126.3 (Ar*C*), 126.3 (Ar*C*), 126.4 (Ar*C*), 127.8 (Ar*C*), 128.2 (Ar*C*), 128.3 (Ar*C*), 133.2 (Ar*C*), 136.2 (Ar*C*), 143.3 (*C*(2)), 176.0 (*C*=O); HRMS (ASAP$^+$) $C_{17}H_{20}O_3$ [M+Na]$^+$ found 268.1463, requires 268.1455 (–3.0 ppm).

Kinetic Resolution of 2-methyl-1-(naphthalen-2-yl)prop-2-en-1-ol 44

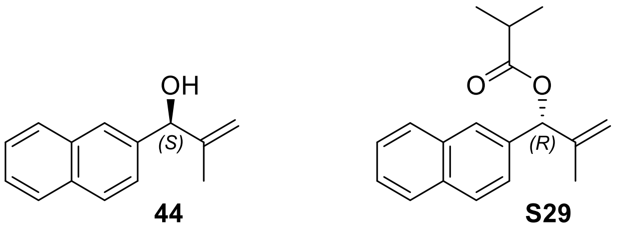

Following General Procedure C, the alcohol **44** (131 mg, 0.66 mmol), HyperBTM **12** (208 µL from stock solution, 7 µmol, 1 mol %), *i*-Pr_2NEt (69 µL, 0.40 mmol) and isobutyric anhydride (53 µL, 0.33 mmol) were reacted in toluene (1.9 mL) for 16 h to give the crude product, which was purified via column chromatography (90:10 Petrol : EtOAc, R_f 0.28 (**44**) and R_f 0.82 (**S29**)) to separate alcohol **44** (59 mg, 0.30 mmol, 45%) and ester **S29** (66 mg, 0.25 mmol, 37%). **Alcohol 44:** $[\alpha]_D^{20}$ +90.0 (*c* 1.0, $CHCl_3$); Chiral HPLC analysis Chiralcel OJ-H (80:20 hexane : IPA, flow rate 1.0 mL min^{-1}, 270 nm, 30 °C) t_R (*S*): 9.9 min, t_R (*R*): 10.9 min, 73% ee. **Ester S29:** $[\alpha]_D^{20}$ –12.8 (*c* 1.1, $CHCl_3$); Chiral HPLC analysis Chiralcel OJ-H (80:20 hexane : IPA, flow rate 1.0 mL min^{-1}, 211 nm, 30 °C) t_R (*R*): 5.1 min, t_R (*S*): 7.0 min, 83 % ee. S = 24.

(*E*)-1-(Naphthalen-2-yl)but-2-en-1-ol 45

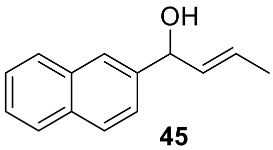

45

Naphthylmagnesium bromide (32 mL of 0.27 M fresh-made solution in THF, 8.5 mmol) was added drop wise to a solution of crotonaldehyde (500 mg, 10 mmol) in anhydrous THF at –78 °C. The reaction was stirred at –78 °C for 3 h, then it was quenched with NH_4Cl and it was extracted with EtOAc (× 3) and the combined organic layers were washed with brine and dried over Na_2SO_4. After evaporation of volatiles, the crude product was purified by silica-gel column chromatography (80:20 Petrol : EtOAc, R_f 0.32), the title compound **45** (489 mg, 25%) as a colourless oil with spectroscopic data in accordance with the literature.[22] ^{1}H NMR (400 MHz, $CDCl_3$) δ_H: 1.73–1.77 (3H, m, C(4)*H*$_3$), 1.96 (1H, d, *J* 3.5, O*H*), 5.28–5.39 (1H, m, C(1)*H*), 5.70–5.88 (2H, m, C(2,3)*H*), 7.48 (3H, ddd, *J* 9.5, 5.2, 1.9, Ar*H*), 7.78–7.89 (4H, m, Ar*H*).

(*E*)-1-(Naphthalen-2-yl)but-2-en-1-yl isobutyrate S30

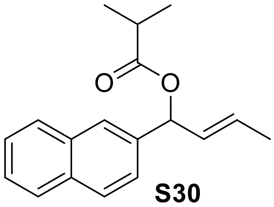

S30

Following General Procedure B, to a solution of DMAP (10 mol%), CH_2Cl_2 (0.2 M) and alcohol **45** (94 mg, 0.47 mmol) was added isobutyric anhydride (76 µL, 0.47 mmol) at room temperature and the reaction mixture was stirred for 16 h. The mixture gave a clean product (115 mg, 91%) as yellow oil. ν_{max} (film) 1728 (C=O); ^{1}H NMR (400 MHz, $CDCl_3$) δ_H: 1.18 (3H, d, *J* 7.0, CH(C*H*$_3$)), 1.21 (3H, d, *J* 7.0, CH(C*H*$_3$)), 1.71–1.76 (3H, m, C(4)*H*$_3$), 2.63 (1H, hept, *J* 7.0, C*H*(CH$_3$)$_2$), 5.65–5.86 (2H, m, C(2,3)*H*), 6.37 (1H, d, *J* 5.7, C(1)*H*), 7.36–7.55 (3H, m, Ar*H*), 7.76–7.89 (4H, m, Ar*H*); ^{13}C{^{1}H} NMR (101 MHz, $CDCl_3$) δ_C: 18.0 (*C*(4)), 19.1 (CH(*C*H$_3$)), 19.1 (CH(*C*H$_3$)), 34.4(*C*H(CH$_3$)$_2$), 76.2 (*C*(1)), 124.9 (Ar*C*), 125.8 (*C*(3)), 126.2 (Ar*C*), 126.3 (Ar*C*), 127.8 (Ar*C*), 128.2 (Ar*C*), 128.4 (*C*(2)), 129.7 (Ar*C*), 129.8 (Ar*C*), 133.1 (Ar*C*), 133.3 (Ar*C*), 137.5 (Ar*C*), 176.2 (*C*=O); HRMS (NSI$^+$) $C_{18}H_{20}O_2$ [M+Na]$^+$ found 291.1356, requires 291.1356 (–0.2 ppm).

Kinetic resolution of (*E*)-1-(naphthalen-2-yl)but-2-en-1-ol 45

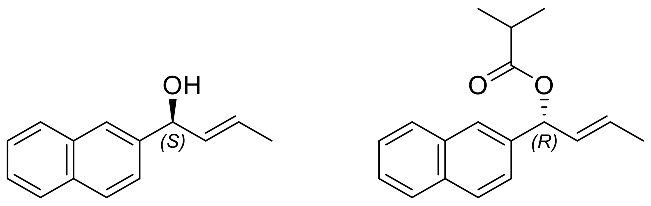

45 **S30**

Following General Procedure C, the alcohol **45** (74 mg, 0.37 mmol), HyperBTM **12** (116 µL from stock solution, 4 µmol, 1 mol %), *i*-Pr_2NEt (39 µL, 0.22 mmol) and isobutyric anhydride (33 µL, 0.20 mmol) were reacted in toluene (1.5 mL). The solution was stirred for 16 h before being quenched with brine. (The reaction is very sentive to the use of acids, which can generate a 1,3-transposition of the alcohol). The solution was diluted with EtOAc and the organic layer was dried over anhydrous Na_2SO_4, filtered and concentrated under reduced pressure. The crude compound was purified via column chromatography (80:20 Petrol : EtOAc, R_f 0.42 (**45**) and R_f 0.69 (**S30**)) to give alcohol **45** (38 mg, 0.19 mmol, 51%) and ester **S30** (38 mg, 0.13 mmol, 38%). **Alcohol 45:** $[\alpha]_D^{20}$ +19.8 (*c* 1.4, $CHCl_3$); Chiral HPLC analysis Chiralcel OJ-H (80:20 hexane : IPA, flow rate 1.0 mL min^{-1}, 211 nm, 30 °C) t_R (*S*): 8.12 min, t_R (*R*): 10.1 min, 63% ee. **Ester S30:** $[\alpha]_D^{20}$ −1.0 (*c* 1.0, $CHCl_3$); Chiral HPLC analysis Chiralcel OJ-H (95:5 hexane : IPA, flow rate 0.5 mL min^{-1}, 211 nm, 30 °C) t_R (*R*): 11.1 min, t_R (*S*): 12.8 min, 70% ee.. S = 11.

(*E*)-2-Methyl-1-(naphthalen-2-yl)but-2-en-1-ol 46

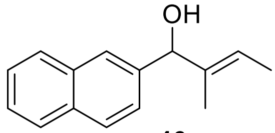

46

Naphthylmagnesium bromide (33 mL of 0.27 M fresh-made solution in THF, 8.8 mmol) was added drop wise to a solution of trans-2-methyl-2-butenal (500 mg, 5.9 mmol) in anhydrous THF at –78 °C. The reaction was stirred at –78 °C for 3 h, then it was quenched with NH_4Cl and it was extracted with EtOAc (× 3) and the combined organic layers were washed with brine and dried over Na_2SO_4. After evaporation of volatiles, the crude product was purified by silica-gel column chromatography (70:30 Petrol : EtOAc, R_f 0.42), the title compound **46** (683 mg, 54%) as a colourless oil. ν_{max} (film) 3356 (O-H), 1605 (C=C); ^1H NMR (400 MHz, $CDCl_3$) δ_H: 1.48–1.53 (3H, m, C(2)C*H₃*), 1.69 (3H, d, *J* 6.8, C(4)*H₃*), 1.92 (1H, d, *J* 3.3, O*H*), 5.31 (1H, d, *J* 2.9, C(3)*H*), 5.73–5.86 (1H, m, C(1)*H*), 7.40–7.44 (1H, m, Ar*H*), 7.44–7.49 (2H, m, Ar*H*), 7.78–7.88 (4H, m, Ar*H*); $^{13}C\{^1H\}$ NMR (126 MHz, $CDCl_3$) δ_C: 11.9 (*C*H_3),

13.4 (CH_3), 79.6 (*C*(1)), 121.9 (*C*(3)), 124.7 (Ar*C*), 124.8 (Ar*C*), 125.9 (Ar*C*), 126.2 (Ar*C*), 127.8 (Ar*C*), 128.0 (Ar*C*), 128.2 (Ar*C*), 132.9 (Ar*C*), 133.4 (Ar*C*), 137.6 (Ar*C*), 140.0 (*C*(2)); HRMS (NSI^+) $C_{15}H_{16}O$ $[M+Na]^+$ found 235.1094, requires 235.1093 (+0.3 ppm).

(*E*)-2-Methyl-1-(naphthalen-2-yl)but-2-en-1-yl isobutyrate S31

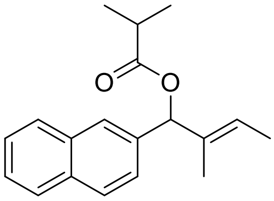

S31

Following General Procedure B, to a solution of DMAP (10 mol%), CH2Cl2 (0.2 M) and alcohol **46** (50 mg, 0.18 mmol) was added isobutyric anhydride (28 µL, 0.17 mmol) at room temperature and the reaction mixture was stirred for 16 h. The mixture gave a clean product (40 mg, 78%) as yellow oil. ν_{max} (film) 1736 (C=O); ^{1}H NMR (500 MHz, $CDCl_3$) δ_H: 1.24 (6H, t, *J* 7.3, CH(C*H*$_3$)$_2$), 1.56 (3H, s, C(2)C*H*$_3$), 1.67 (3H, d, *J* 6.3, C(4)*H*$_3$), 2.68 (1H, p, *J* 7.0, C*H*(CH_3)$_2$), 5.72 (1H, q, *J* 6.4, C(3)*H*), 6.34 (1H, s, C(1)*H*), 7.41 (1H, d, *J* 8.4, Ar*H*), 7.43–7.55 (2H, m, Ar*H*), 7.71–7.91 (4H, m, Ar*H*); ^{13}C{^{1}H} NMR (101 MHz, $CDCl_3$) δ_C: 12.5 (CH_3), 13.5 (CH_3), 19.2 (CH(CH_3)), 19.2 (CH(CH_3)), 34.5 (*C*H(CH_3)$_2$), 79.9 (*C*(1)), 123.3 (*C*(3)), 124.8 (Ar*C*), 125.6 (Ar*C*), 126.1 (Ar*C*), 126.2 (Ar*C*), 127.8 (Ar*C*), 128.1 (Ar*C*), 128.2 (Ar*C*), 133.0 (Ar*C*), 133.2 (Ar*C*), 134.2 (Ar*C*), 137.0 (*C*(2)), 176.1 (*C*=O); HRMS could not be obtained due to significant fragmentation.

Kinetic resolution of (*E*)-2-methyl-1-(naphthalen-2-yl)but-2-en-1-ol 46

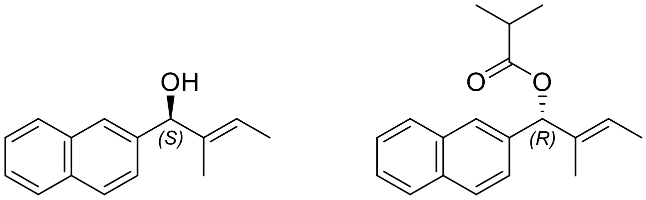

46 **S31**

The alcohol **46** (173 mg, 0.81 mmol), HyperBTM **12** (254 µL from stock solution, 8 µmol, 1 mol %), *i*-Pr_2NEt (85 µL, 0.49 mmol) and isobutyric anhydride (66 µL, 0.41 mmol) were reacted in toluene (2.0 mL). The solution was stirred for 16 h before being quenched with brine. (The reaction is very sentive to the use of acids, which can generate a 1,3-transposition of the alcohol). The solution was diluted with EtOAc and the organic layer was dried over anhydrous Na_2SO_4, filtered and concentrated under reduced pressure. The crude compound was purified via column chromatography (75:25 Petrol : EtOAc, R_f 0.52 (**46**) and R_f 0.80

(**S31**)) to separate alcohol **46** (78 mg, 0.37 mmol, 45%) and ester **S31** (122 mg, 0.43 mmol, 48%). **Alcohol 46:** $[\alpha]_D^{20}$ +49.0 (*c* 0.5, $CHCl_3$); Chiral HPLC analysis Chiralcel OJ-H (80:20 hexane : IPA, flow rate 1.0 mL min^{-1}, 211 nm, 30 °C) t_R (*S*): 7.6 min, t_R (*R*): 8.4 min, 68% ee. **Ester S31:** $[\alpha]_D^{20}$ −1.0 (*c* 1.0, $CHCl_3$); Chiral HPLC analysis Chiralcel OJ-H (95:5 hexane : IPA, flow rate 0.5 mL min^{-1}, 211 nm, 30 °C) t_R (*R*): 11.0 min, t_R (*S*): 14.9 min, 60% ee. S = 8.

1-(Naphthalen-2-yl)propan-1-ol 47

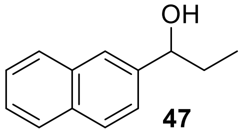

47

Following General Procedure A, ethylmagnesium bromide (1.7 mL of 3.0 M solution in Et_2O, 5.09 mmol) added to a solution of 2-naphthaldehyde (530 mg, 3.39 mmol) in anhydrous Et_2O (20 mL) at –78 °C for 16 h gave, after purification by column chromatography (70:30 Hexane : EtOAc, R_f 0.42), the title compound **47** (548 mg, 87%) as a white solid with spectroscopic data in accordance with the literature.[23] mp 38–39 °C {Lit.[23] 37–38 °C}; ^{1}H NMR (300 MHz, $CDCl_3$) δ_H: 0.95 (3H, t, *J* 7.4, C(3)H_3), 1.78–1.95 (3H, m, C(2)H_2 and O*H*), 4.78 (1H, td, *J* 6.6, 3.3, C(1)*H*), 7.42–7.53 (3H, m, Ar*H*), 7.78 (1H, s, Ar*H*), 7.84 (3H, dd, *J* 7.3, 2.3, Ar*H*).

1-(Naphthalen-2-yl)propyl isobutyrate S32

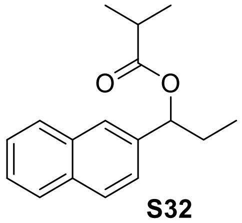

S32

Following General Procedure B, to a solution of DMAP (10 mol %), CH_2Cl_2 (0.2 M) and alcohol **47** (83 mg, 0.48 mmol) was added isobutyric anhydride (79 µL, 0.48 mmol) at room temperature and the reaction mixture was stirred for 16 h. The mixture gave a clean product (91 mg, 73%) as a colourless oil. ν_{max} (film) 1730 (C=O), 1606 (C=C); ^{1}H NMR (500 MHz, $CDCl_3$) δ_H: 0.92 (3H, t, *J* 7.0, CH(CH_3)), 1.16 (3H, d, *J* 7.0, CH(CH_3)), 1.21 (3H, d, *J* 7.0, CH(CH_3)), 1.91 (1H, ddd, *J* 14.8, 7.3, 6.4, C(2)*H*), 2.00 (1H, dq, *J* 14.8, 7.3, C(2)*H*), 2.61 (1H, hept, *J* 7.0, C*H*(CH_3)$_2$), 5.79–5.85 (1H, m, C(1)*H*), 7.42–7.51 (3H, m, Ar*H*), 7.77 (1H, s, Ar(1)*H*), 7.82 (3H, dd, *J* 7.7, 4.1, Ar*H*); ^{13}C{^{1}H} NMR (126 MHz, $CDCl_3$) δ_C: 10.1 (*C*(3)), 19.1 (CH(*C*H_3)), 19.2 (CH(*C*H_3)), 29.5 (*C*(2)), 34.4 (*C*H(CH_3)), 77.2 (*C*(1)), 124.4 (Ar*C*), 125.7 (Ar*C*), 126.1 (Ar*C*), 126.3 (Ar*C*), 127.8 (Ar*C*), 128.1 (Ar*C*), 128.3 (Ar*C*), 133.1 (Ar*C*),

133.3 (Ar*C*), 138.3 (Ar*C*), 176.6 (*C*=O); HRMS (NSI$^+$) $C_{17}H_{20}O_2$ [M+Na]$^+$ found 279.1354, requires 279.1356 (–0.5 ppm).

Kinetic resolution of 1-(naphthalen-2-yl)propan-1-ol 47

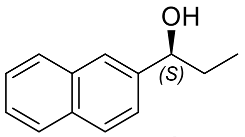

47

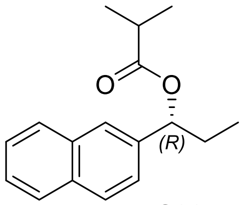

S32

Following General Procedure C, the alcohol **47** (66 mg, 0.38 mmol), HyperBTM **12** (120 µL from stock solution, 4 µmol, 1 mol %), *i*-Pr_2NEt (40 µL, 0.23 mmol) and isobutyric anhydride (31 µL, 0.19 mmol) were reacted in toluene (1.0 mL) for 16 h to give the crude product, which was purified via column chromatography (80:20 Hexane : EtOAc, R_f 0.27 (**47**) and R_f 0.55 (**S32**)) to separate alcohol **47** (24.5 mg, 0.14 mmol, 37%) and ester **S32** (38 mg, 0.15 mmol, 39%). **Alcohol 47:** $[\alpha]_D^{20}$ –35.2 (*c* 1.0, $CHCl_3$); Chiral HPLC analysis Chiralcel OJ-H (80:20 hexane : IPA, flow rate 1.0 mL min^{-1}, 254 nm, 30 °C) t_R (*S*): 8.7 min, t_R (*R*): 11.3 min, >99% ee. **Ester S32:** $[\alpha]_D^{20}$ +81.2 (*c* 2.5, $CHCl_3$); Chiral HPLC analysis Chiralcel OJ-H (90:10 hexane : IPA, flow rate 0.8 mL min^{-1}, 220 nm, 30 °C) t_R (*R*): 7.2 min, t_R (*S*): 9.3 min, 93 % ee. S = 152.

1-(Naphthalen-2-yl)prop-2-yn-1-ol 48

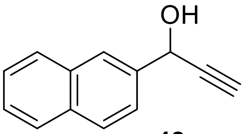

48

Following the procedure proposed by Gong,[24] to a solution of trimethylsilyl acetylene (346 mg, 3.52 mmol) in anhydrous THF at –78 °C under N_2, was added ^{n}BuLi (1.8 mL of 1.92 M solution in hexanes, 3.5 mmol) dropwise. The reaction was stirred at –78 °C for 1 h and then 2-naphtaldehyde (500 mg, 3.20 mmol) was added. The mixture was stirred for 30 min at –78 °C and it was allowed to warm at rt for 30 min. The mixture was quenched with water and extracted with EtOAc (× 3) and the combined organic layers were washed with brine and dried over Na_2SO_4. After evaporation of volatiles, K_2CO_3 (1.5 g, 10.6 mmol) was added to the crude in MeOH and the solution was stirring at rt for 2 h. After completion, the reaction was filtered and washed with CH_2Cl_2. The filtrate was washed with NH_4Cl, brine and dried with Na_2SO_4. After evaporation of volatiles, the crude product was purified by silica-gel column chromatography (80:20 Petrol : EtOAc, R_f 0.31), the title compound **48** (281 mg,

48%) as a white solid with spectroscopic data in accordance with the literature.[25] mp 58–59 °C {Lit.[25] 54–55 °C}; ^{1}H NMR (500 MHz, $CDCl_3$) δ_H: 2.27 (1H, d, *J* 6.3, O*H*), 2.74 (1H, d, *J* 2.2, C(3)*H*), 5.61–5.68 (1H, m, C(1)*H*), 7.47–7.55 (2H, m, Ar*H*), 7.66 (1H, dd, *J* 8.5, 1.6, Ar*H*), 7.80–7.92 (3H, m, Ar*H*), 8.02 (1H, s, Ar*H*).

1-(Naphthalen-2-yl)prop-2-yn-1-yl isobutyrate S33

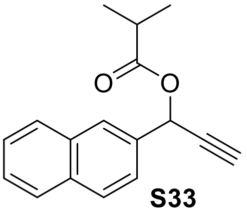

S33

Following General Procedure B, to a solution of DMAP (10 mol %), CH_2Cl_2 (0.2 M) and alcohol **48** (75 mg, 0.41 mmol) was added isobutyric anhydride (67 µL, 0.41 mmol) at room temperature and the reaction mixture was stirred for 16 h. The mixture gave a clean product (86 mg, 84%) as a white solid. mp 68–69 °C; ν_{max} (film) 3289 (C-H); 1736 (C=O); ^{1}H NMR (400 MHz, $CDCl_3$) δ_H: 1.17 (3H, d, *J* 7.0, CH(C*H₃*)), 1.22 (3H, d, *J* 7.0, CH(C*H₃*)), 2.63 (1H, hept, *J* 7.0, C*H*(CH_3)$_2$) 2.70 (1H, d, *J* 2.3, C(3)*H*), 6.62 (1H, d, *J* 2.3, C(1)*H*), 7.52 (2H, dt, *J* 6.3, 3.4, Ar*H*), 7.60 (1H, dd, *J* 8.5, 1.7, Ar*H*), 7.82–7.91 (3H, m, Ar*H*), 7.99–8.03 (1H, m, Ar*H*); ^{13}C{^{1}H} NMR (126 MHz, $CDCl_3$) δ_C: 18.9 (CH(*C*H_3), 19.0 (CH(*C*H_3), 34.1 (*C*H(CH_3)$_2$, 65.3 (*C*(1)), 75.7 (*C*(3)), 80.5 (*C*(2)), 125.0 (Ar*C*), 126.6 (Ar*C*), 126.8 (Ar*C*), 127.1 (Ar*C*), 127.8 (Ar*C*), 128.4 (Ar*C*), 128.8 (Ar*C*), 133.1 (Ar*C*), 133.6 (Ar*C*), 134.1 (Ar*C*), 176.0 (*C*=O); HRMS (NSI$^+$) $C_{17}H_{16}O_2$ [M+Na]$^+$ found 275.1044, requires 275.1043 (+0.5 ppm).

Kinetic resolution of 1-(naphthalen-2-yl)prop-2-yn-1-ol 48

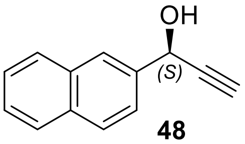

48

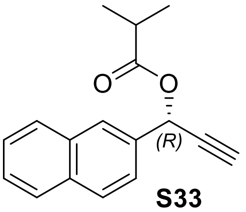

S33

Following General Procedure C, the alcohol **48** (52 mg, 0.28 mmol), HyperBTM **12** (89 µL from stock solution, 3 µmol, 1 mol %), *i*-Pr_2NEt (30 µL, 0.17 mmol) and isobutyric anhydride (23 µL, 0.14 mmol) were reacted in toluene (0.8 mL) for 16 h to give the crude product, which was purified via column chromatography (80:20 Petrol : EtOAc, R_f 0.31 (**48**) and R_f 0.67 (**S33**)) to separate alcohol **48** (19 mg, 0.10 mmol, 35%) and ester **S33** (26 mg, 0.10 mmol, 36%). **Alcohol 48:** $[\alpha]_D^{20}$ +5.1 (*c* 1.0, $CHCl_3$); Chiral HPLC analysis Chiralcel OJ-

H (80:20 hexane : IPA, flow rate 1.0 mL min^{-1}, 211 nm, 30 °C) t_R (*S*): 22.1 min, t_R (*R*): 25.5 min, 32% ee. **Ester S33:** $[\alpha]_D^{20}$ +47.0 (*c* 0.3, $CHCl_3$); Chiral HPLC analysis Chiralcel OJ-H (90:10 hexane : IPA, flow rate 1.0 mL min^{-1}, 270 nm, 30 °C) t_R (*R*): 21.4 min, t_R (*S*): 23.4 min, 28% ee. S = 3.

5-Phenylpent-1-en-3-ol 49

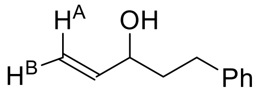

49

Following General Procedure A, vinylmagnesium bromide (8.0 mL of 0.7M solution in THF, 5.59 mmol) added to a solution of 3-phenylpropionaldehyde (500 mg, 3.72 mmol) in anhydrousTHF (20 mL) at –78 °C for 16 h gave, after purification by column chromatography (80:20 Hexane : EtOAc, R_f0.32), the title compound **49** (340 mg, 56%) as a yellow oil with spectroscopic data in accordance with the literature.[26] ^{1}H NMR (500 MHz, $CDCl_3$) δ_H: 1.51 (1H, d, *J* 4.2, O*H*), 1.80–1.93 (2H, m, C(4)H_2), 2.65–2.80 (2H, m, C(5)H_2), 4.10–4.17 (1H, m,C(3)*H*), 5.14 (1H, dt, *J* 10.4, 1.4, C(1)H^B), 5.25 (1H, dt, *J* 17.1, 1.4, C(1)H^A), 5.91 (1H, ddd, *J* 17.1, 10.4, 6.1, C(2)*H*), 7.17–7.23 (3H, m, Ph*H*), 7.27–7.32 (2H, m, Ph*H*); ^{13}C{^{1}H} NMR (126 MHz, $CDCl_3$) δ_C: 31.8 (*C*(4)), 38.6 (*C*(5)), 72.6 (*C*(3)), 115.1 (*C*(1)), 126.0 (Ph*C*(4)), 128.5 (Ph*C*(2,6)), 128.6 (Ph*C*(3,5)), 141.1 (*C*(2)), 142.0 (Ph*C*(1)).

5-Phenylpent-1-en-3-yl isobutyrate S34

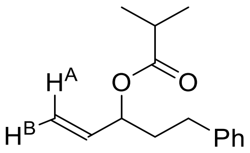

S34

Following General Procedure B, to a solution of DMAP (10 mol%), CH_2Cl_2 (0.2 M) and alcohol **49** (74 mg, 0.45 mmol) was added isobutyric anhydride (74 µL, 0.45 mmol) at room temperature and the reaction mixture was stirred for 16 h. The mixture gave a clean product (68 mg, 65%) as a yellow oil. ν_{max} (film) 1732 (C=O); ^{1}H NMR (500 MHz, $CDCl_3$) δ_H: 1.19 (3H, d, *J* 5.5, CH(CH_3)), 1.20 (3H, d, *J* 5.5, CH(CH_3)), 1.86–1.95 (1H, m, C(5)*H*), 1.95–2.03 (1H, m, C(5)*H*), 2.56 (1H, dq, *J* 14.0, 7.0, C*H*(CH_3)$_2$), 2.60–2.72 (2H, m, C(4)H_2), 5.19 (1H, dt, *J* 10.6, 1.3, C(1)H^B), 5.26 (1H, dt, *J* 17.0, 1.3, C(1)H^A), 5.27 (1H, q, *J* 6.3, C(3)*H*), 5.81 (1H, ddd, *J* 17.0, 10.6, 6.3, C(2)*H*), 7.15–7.21 (3H, m, Ph*H*), 7.28 (2H, t, *J* 7.5, Ph*H*); ^{13}C{^{1}H} NMR (126 MHz, $CDCl_3$) δ_C: 19.1 (CH(*C*H_3)), 19.2 (CH(*C*H_3)), 31.6 (*C*(5)), 34.4

($CH(CH_3)_2$), 36.1 (*C*(4)), 73.9 (*C*(3)), 116.8 (*C*(1)), 126.1 (Ph*C*(4)), 128.5 (Ph*C*), 128.6 (Ph*C*), 136.6 (*C*(2)), 141.5 (Ph*C*(1)), 176.5 (*C*=O); HRMS (FTMS + NSI^+) $C_{15}H_{20}O_2$ $[M+NH_4]^+$ found 250.1802, requires 250.1802 (+ 0.2 ppm).

Kinetic resolution of 5-phenylpent-1-en-3-ol 49

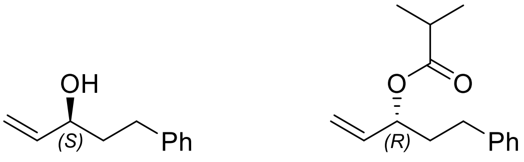

49 **S34**

Following General Procedure C, the alcohol **49** (86 mg, 0.53 mmol), HyperBTM **12** (165 µL from stock solution, 5 µmol, 1 mol %), *i*-Pr_2NEt (55 µL, 0.32 mmol) and isobutyric anhydride (43 µL, 0.26 mmol) were reacted in toluene (1.5 mL) for 16 h to give the crude product, which was purified via column chromatography (80:20 Petrol : EtOAc, R_f 0.29 (**49**) and R_f 0.60 (**S34**)) to separate alcohol **49** (30 mg, 0.19 mmol, 35%) and ester **S34** (31 mg, 0.13 mmol, 25%). **Alcohol 49:** $[\alpha]_D^{20}$ –2.6 (*c* 1.7, $CHCl_3$) {Lit.[26] $[\alpha]_D^{21}$ –5.8 (*c* 1.3, $CHCl_3$)}; Chiral HPLC analysis Chiralcel OD-H (88:12 hexane : IPA, flow rate 1.0 mL min^{-1}, 211 nm, 35 °C) t_R (*S*): 6.2 min, t_R (*R*): 7.6 min, 21% ee. **Ester S34:** $[\alpha]_D^{20}$ –1.0 (*c* 1.2, $CHCl_3$); Chiral HPLC analysis Chiralcel OJ-H (99:1 hexane : IPA, flow rate 1.0 mL min^{-1}, 220 nm, 30 °C) t_R (*S*): 6.2 min, t_R (*R*): 7.1 min, 36% ee. S = 3.

Preparative Kinetic Resolution

Kinetic resolution of 1-(naphthalen-2-yl)prop-2-en-1-ol 32

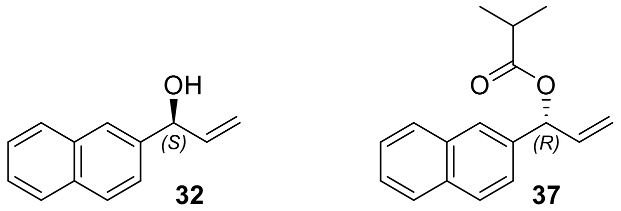

Following General Procedure C, the alcohol **32** (2.5 g, 13.6 mmol), HyperBTM **12** (4.9 mg, 135.8 µmol, 1 mol %), *i*-Pr_2NEt (1.42 mL, 8.15 mmol) and isobutyric anhydride (1.10 mL, 6.79 mmol) were reacted in toluene (20 mL) for 48 h to give the crude product, which was purified via column chromatography to separate alcohol **32** (1.08 g, 5.86 mmol, 43%) and ester **37**(1.54 g, 6.05 mmol, 45%). **Alcohol 32:** $[\alpha]_D^{20}$ +5.0 (*c* 0.7, $CHCl_3$); Chiral HPLC analysis Chiralcel OJ-H (80:20 hexane : IPA, flow rate 1.0 mL min^{-1}, 254 nm, 30 °C) t_R (*S*): 11.7 min, t_R (*R*): 14.1 min, 98% ee. **Ester 37:** $[\alpha]_D^{20}$ +54.0 (*c* 1.0, $CHCl_3$); Chiral HPLC analysis Chiralcel OJ-H (95:5 hexane : IPA, flow rate 0.5 mL min^{-1}, 270 nm, 30 °C) t_R (*R*): 14.2 min, t_R (*S*): 18.0 min, >99% ee. S = 2203.

Hydrolysis of 1-(naphthalen-2-yl)allyl isobutyrate

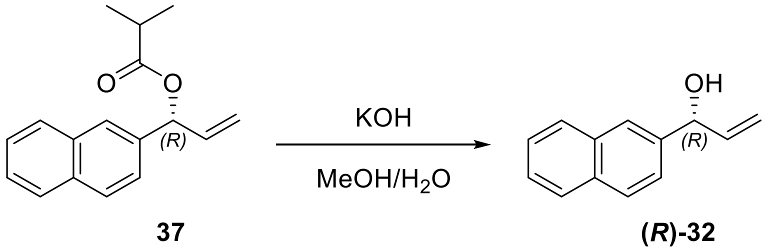

To a solution of ester **37** (1.54 g, 6.05 mmol, >99% ee) in MeOH was added dropwise a solution of KOH (333 mg, 5.94 mmol) in MeOH/H_2O (5:2) at room temperature and the reaction mixture was stirred for 16 h. After, H_2O was added and the mixture was extracted with CH_2Cl_2. The organic layer was dried over anhydrous $MgSO_4$, filtered and concentrated under reduced pressure. The product (1.12 g, 100%) was obtained as a clean white solid without any purification and it was analysed by chiral HPLC (>99% ee); $[\alpha]_D^{20}$ –6.3 (*c* 0.7, $CHCl_3$).

Linear Regression Analysis

A more accurate selectivity factor for the kinetic resolution of (±)-**32** was calculated by linear regression analysis of multiple experiments that were stopped at different reaction conversions (Table S1). The experiments (Table 1, entries 1-6) were performed following General Procedure C using alcohol **32**, HyperBTM **12** (1 mol% from stock solution), *i*-Pr_2NEt (0.6 eq) and isobutyric anhydride (0.5 eq) in toluene (0.6 mL) at –78 °C. The reactions were quenched at different times with HCl and the organic layer was concentrated under reduced pressure. The crude product was analysed by chiral HPLC. The data from Table S1 was plotted as shown in Figure S1, with the selectivity factor determined as the gradient of the line of best fit (R^2 = 0.98) through the points.

Table S1. Data for linear regression analysis for the kinetic resolution of (±)-**32**.

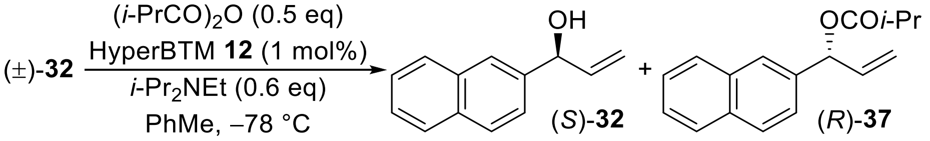

Entry	Conv (%)	ee **32**	er **32**	ee **37**	er **37**	S
1	10	10.564	55:45	99.999	>99.5:0.5	221955
2	24	31.970	66:34	99.848	>99:1	1798
3	35	54.850	77:23	99.856	>99:1	2414
4	38	62.378	81:19	99.854	>99:1	2605
5	48	94.112	97:3	99.612	>99:1	1854
6	50	98.290	99:1	99.566	>99:1	2203

Figure S1. Linear regression analysis plot.

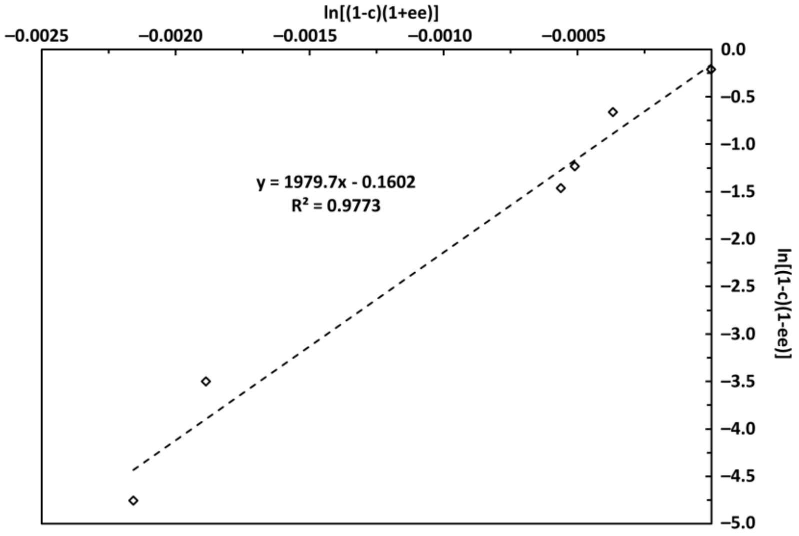

References

[1] a) G. Smitha, C. Sanjeeva Reddy, *Synth. Commun.* **2006**, *36*, 1795-1800; b) F. Wu, H. Li, R. Hong, L. Deng, *Angew. Chem. Int. Ed.* **2006**, *45*, 947-950.

[2] D. S. Hamilton, D. A. Nicewicz, *J. Am. Chem. Soc.* **2012**, *134*, 18577-18580.

[3] N. Kanbayashi, K. Onitsuka, *Angew. Chem. Int. Ed.* **2011**, *50*, 5197-5199.

[4] J. Lehmann, G. C. Lloyd-Jones, *Tetrahedron* **1995**, *51*, 8863-8874.

[5] C. M. Latham, A. J. Blake, W. Lewis, M. Lawrence, S. Woodward, *Eur. J. Org. Chem.* **2012**, *2012*, 699-707.

[6] Calculated from the enantiomeric excess of the corresponding ester and the reaction conversion as determined by ^{1}H NMR.

[7] A. W. J. Logan, J. S. Parker, M. S. Hallside, J. W. Burton, *Org. Lett.* **2012**, *14*, 2940-2943.

[8] B. Schmidt, *J. Org. Chem.* **2004**, *69*, 7672-7687.

[9] B. Goument, L. Duhamel, R. Mauge, *Tetrahedron* **1994**, *50*, 171-188.

[10] I. Lyothier, C. Defieber, E. M. Carreira, *Angew. Chem. Int. Ed.* **2006**, *45*, 6204-6207.

[11] M. Ihara, M. Toyota, M. Abe, Y. Ishida, K. Fukumoto, T. Kametani, *J. Chem. Soc., Perkin Trans. 1* **1986**, 1543-1549.

[12] C. S. Lancefield, O. S. Ojo, F. Tran, N. J. Westwood, *Angew. Chem. Int. Ed.* **2015**, *54*, 258-262.

[13] P.-S. Wang, X.-L. Zhou, L.-Z. Gong, *Org. Lett.* **2014**, *16*, 976-979.

[14] a) P. K. Tiwari, I. S. Aidhen, *Synlett* **2013**, *24*, 1777-1780; b) Y. Zhang, B. Liu, X. Wu, R. Li, X. Ning, Y. Liu, Z. Liu, Z. Ge, R. Li, Y. Yin, *Bioorg. Med. Chem.* **2015**, *23*, 4815-4823.

[15] a) M. Vellakkaran, M. M. S. Andappan, K. Nagaiah, J. B. Nanubolu, *Eur. J. Org. Chem.* **2016**, *2016*, 3575-3583; b) J. D. Lambert, J. E. Rice, J. Hong, Z. Hou, C. S. Yang, *Bioorg. Med. Chem. Lett.* **2005**, *15*, 873-876; c) A. Briot, C. Baehr, R. Brouillard, A. Wagner, C. Mioskowski, *J. Org. Chem.* **2004**, *69*, 1374-1377.

[16] A. Bouziane, M. Hélou, B. Carboni, F. Carreaux, B. Demerseman, C. Bruneau, J.-L.

Renaud, *Chem. Eur. J.* **2008**, *14*, 5630-5637.

[17] R. A. Fernandes, P. Kattanguru, *Helv. Chem. Acta* **2015**, *98*, 92-107.

[18] E. Kim, S. Choi, H. Kim, E. J. Cho, *Chem. Eur. J.* **2013**, *19*, 6209-6212.

[19] J. Štambaský, A. V. Malkov, P. Kočovský, *J. Org. Chem.* **2008**, *73*, 9148-9150.

[20] C. Morrill, G. L. Beutner, R. H. Grubbs, *J. Org. Chem.* **2006**, *71*, 7813-7825.

[21] S. Akai, R. Hanada, N. Fujiwara, Y. Kita, M. Egi, *Org. Lett.* **2010**, *12*, 4900-4903.

[22] J. Barluenga, F. J. Fañanás, R. Sanz, C. Marcos, M. Trabada, *Org. Lett.* **2002**, *4*, 1587-1590.

[23] B. L. H. Taylor, E. C. Swift, J. D. Waetzig, E. R. Jarvo, *J. Am. Chem. Soc.* **2011**, *133*, 389-391.

[24] F. Shi, S.-W. Luo, Z.-L. Tao, L. He, J. Yu, S.-J. Tu, L.-Z. Gong, *Org. Lett.* **2011**, *13*, 4680-4683.

[25] Y. Maeda, N. Kakiuchi, S. Matsumura, T. Nishimura, T. Kawamura, S. Uemura, *J. Org. Chem.* **2002**, *67*, 6718-6724.

[26] I. Sato, N. Asakura, T. Iwashita, *Tetrahedron: Asymmetry* **2007**, *18*, 2638-2642.

NMR Spectra

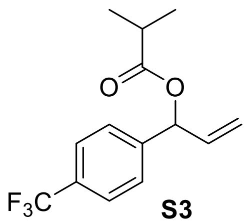

S3

^{1}H, $CDCl_3$, 400 MHz

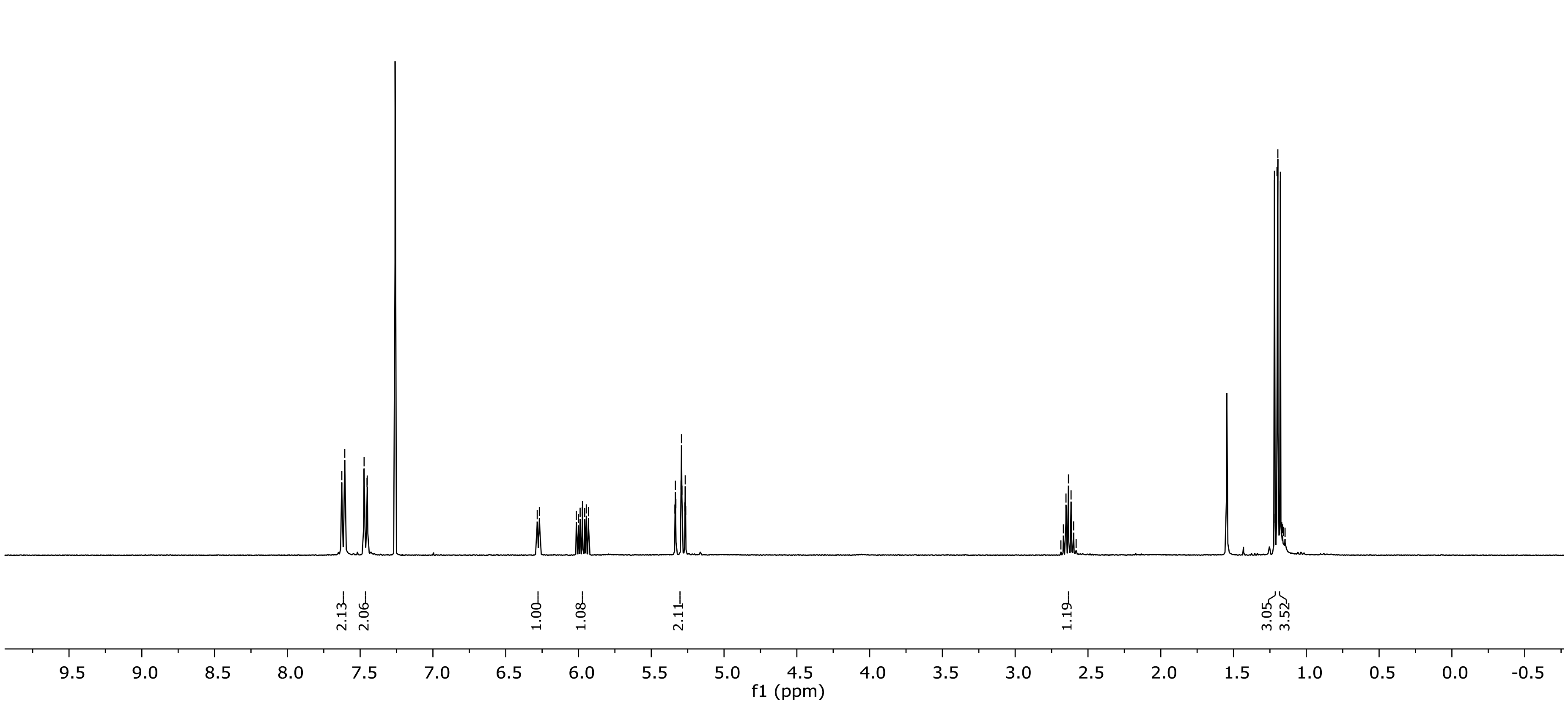

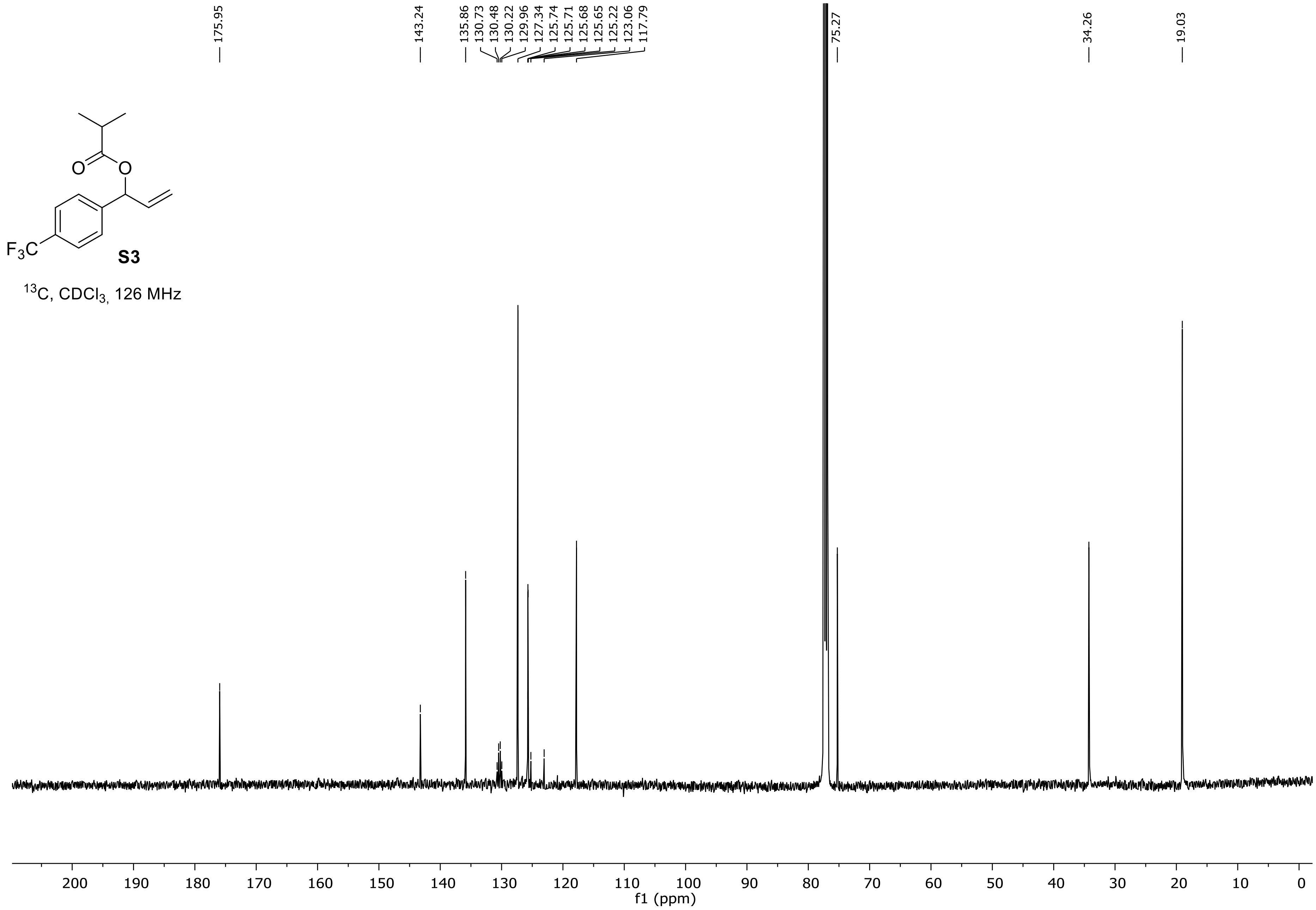
S3
^{13}C, $CDCl_3$, 126 MHz
175.95
143.24
135.86
130.73
130.48
130.22
129.96
127.34
125.74
125.71
125.68
125.65
125.22
123.06
117.79
75.27
34.26
19.03
200 190 180 170 160 150 140 130 120 110 100 90 80 70 60 50 40 30 20 10 0
f1 (ppm)

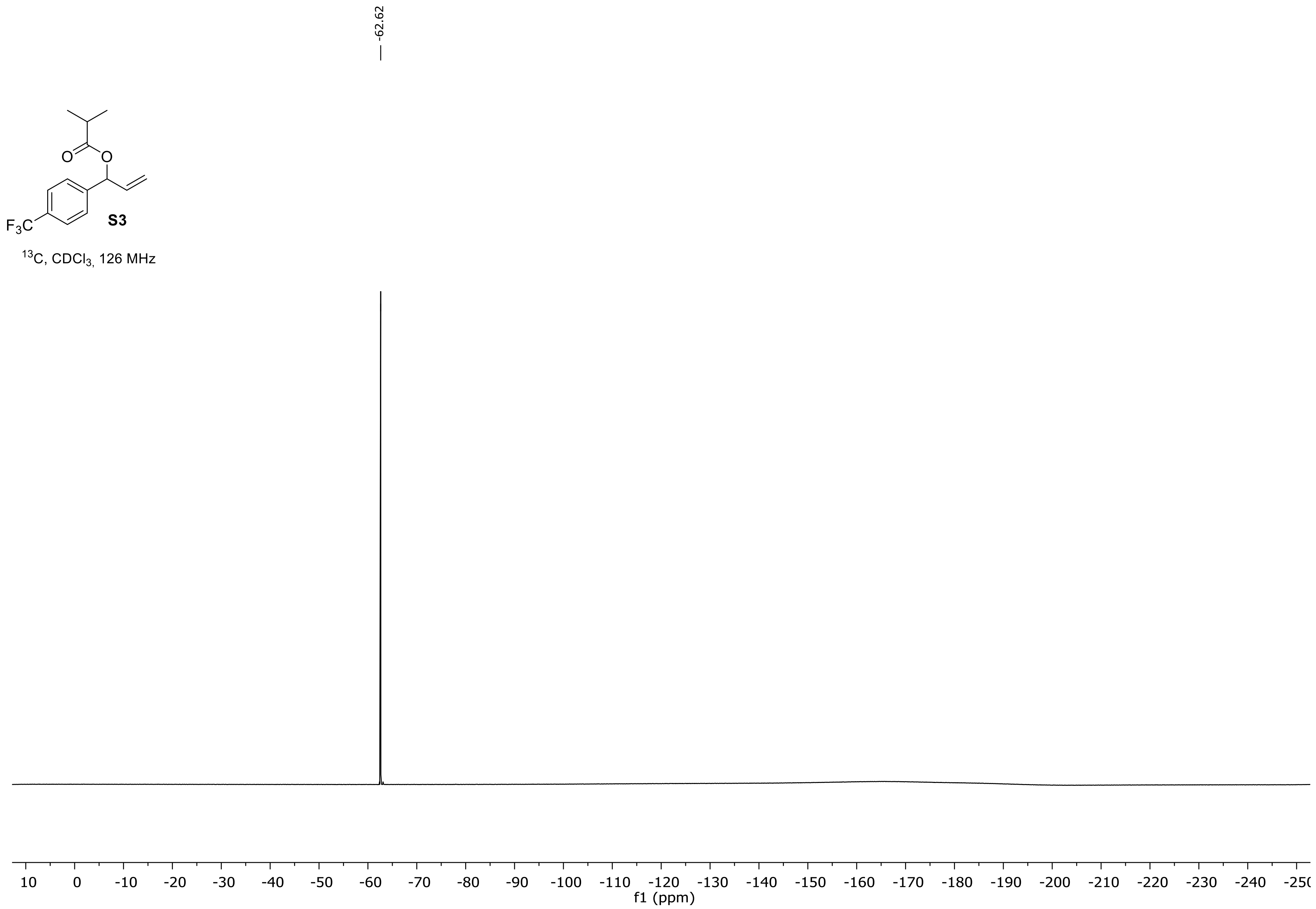

^{13}C, $CDCl_3$, 126 MHz

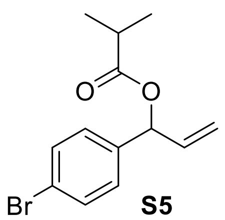

S5

^{1}H, $CDCl_3$, 500 MHz

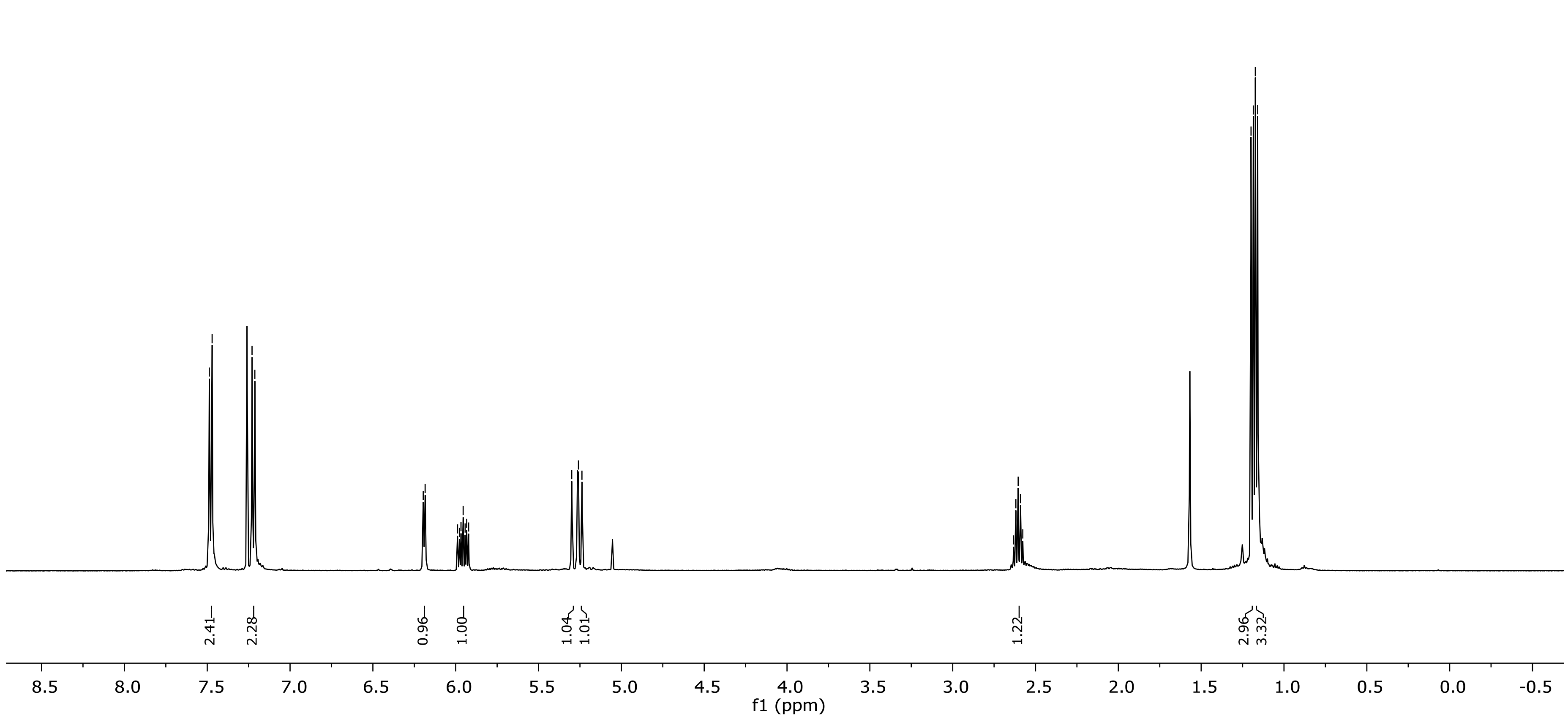

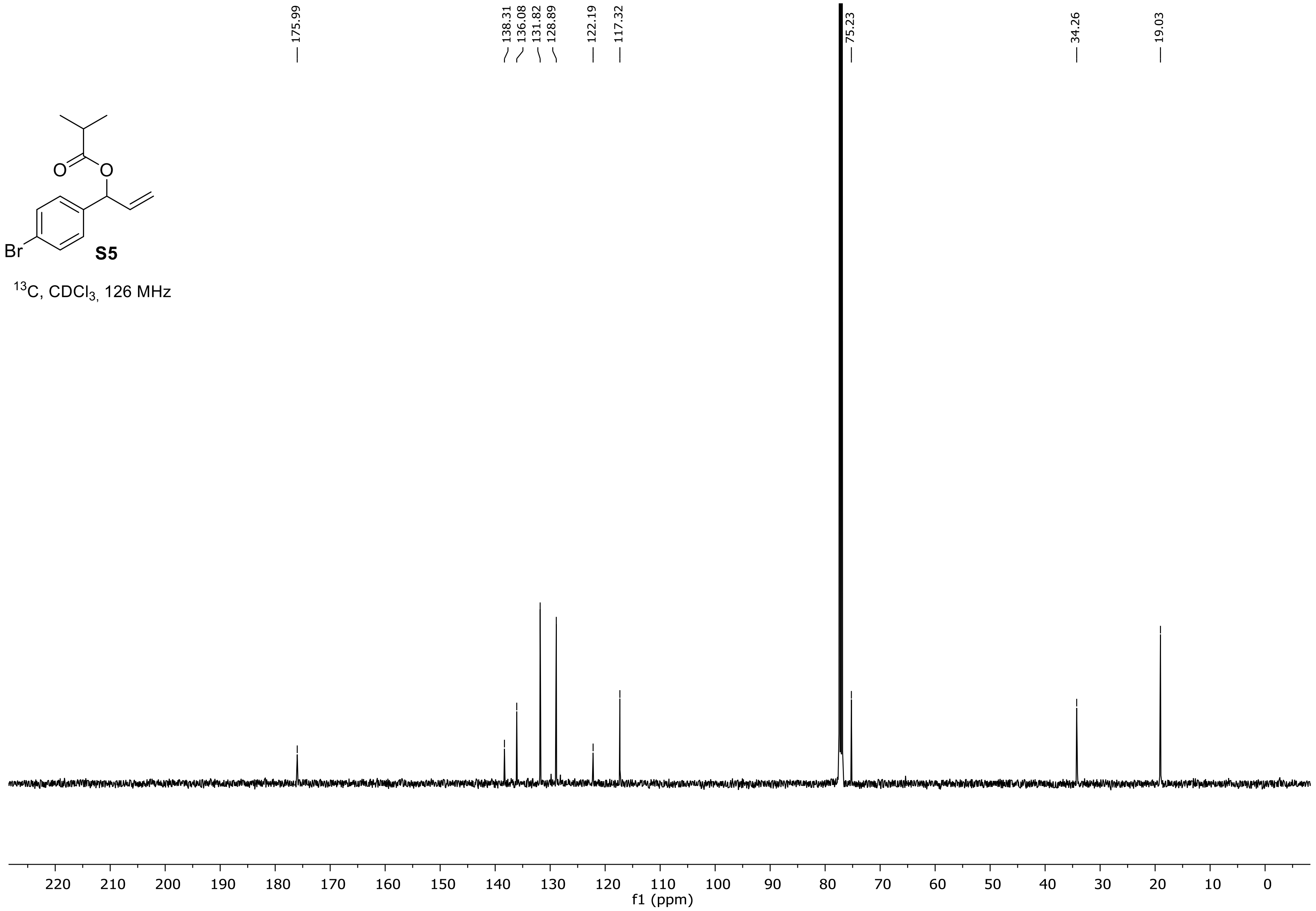
175.99
138.31
136.08
131.82
128.89
122.19
117.32
75.23
34.26
19.03
Br
S5
^{13}C, $CDCl_3$, 126 MHz
f1 (ppm)

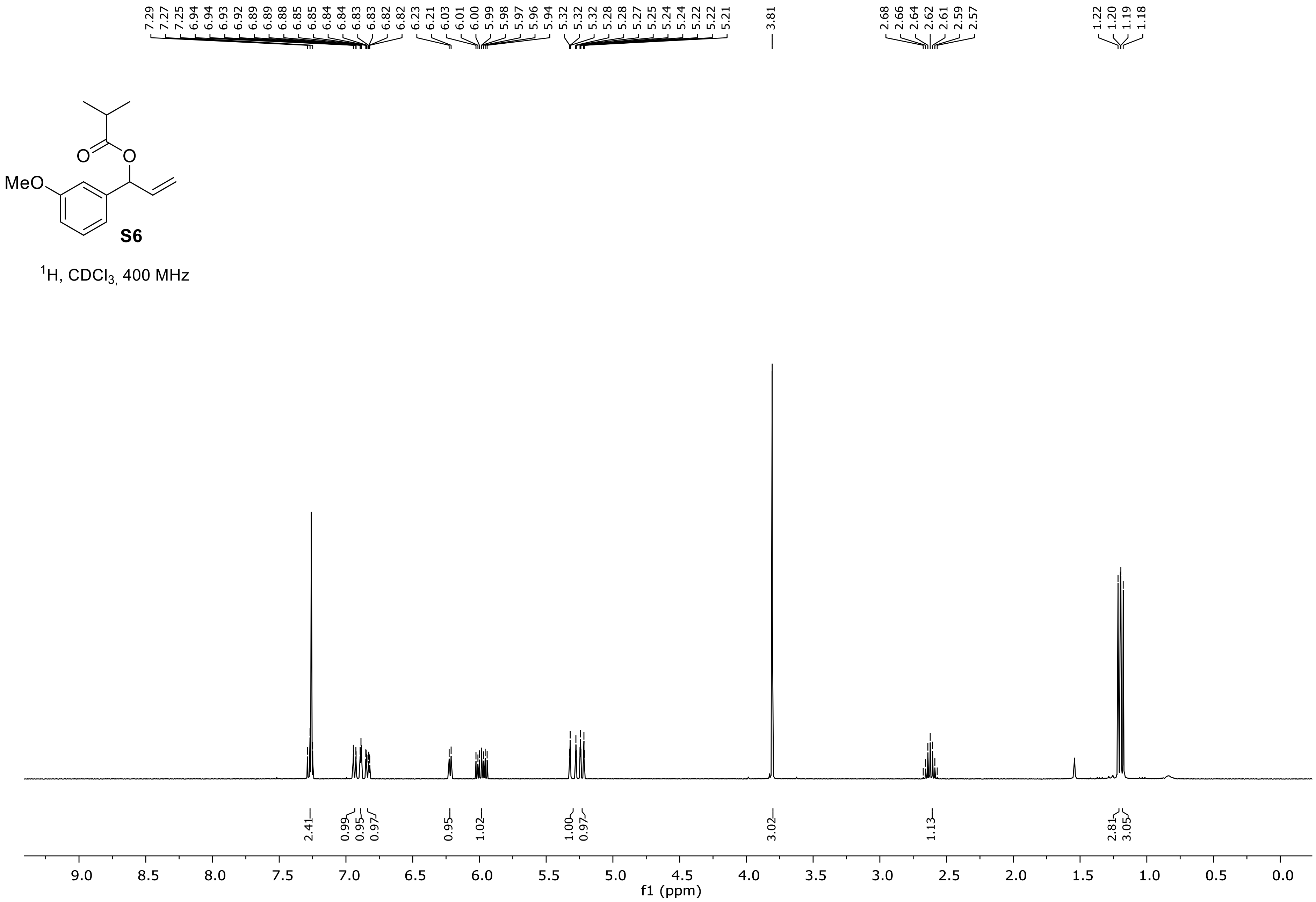
7.29 7.27 7.25 6.94 6.93 6.92 6.89 6.88 6.85 6.85 6.84 6.84 6.83 6.83 6.82 6.82 6.23 6.21 6.03 6.01 6.00 5.99 5.98 5.97 5.96 5.94 5.32 5.32 5.32 5.28 5.28 5.27 5.25 5.24 5.24 5.22 5.22 5.21
3.81
2.68 2.66 2.64 2.62 2.61 2.59 2.57
1.22 1.20 1.19 1.18
MeO
O
O
S6
^{1}H, CDCl$_3$, 400 MHz
2.41 0.99 0.95 0.97 0.95 1.02 1.00 0.97 3.02 1.13 2.81 3.05
9.0 8.5 8.0 7.5 7.0 6.5 6.0 5.5 5.0 4.5 4.0 3.5 3.0 2.5 2.0 1.5 1.0 0.5 0.0
f1 (ppm)

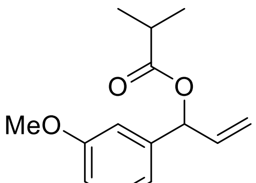

S6

^{13}C, $CDCl_3$, 126 MHz

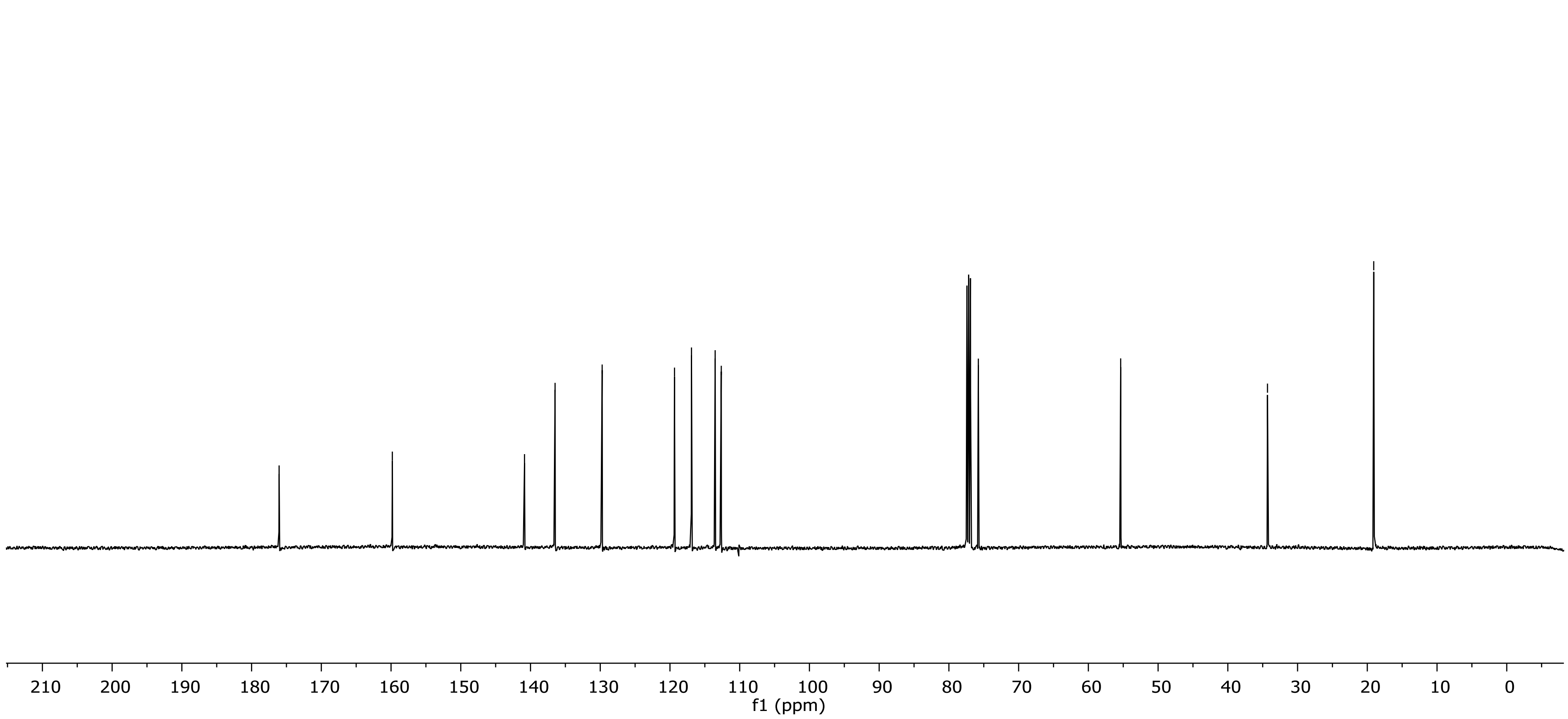

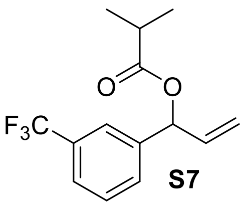

^{1}H, $CDCl_3$, 500 MHz

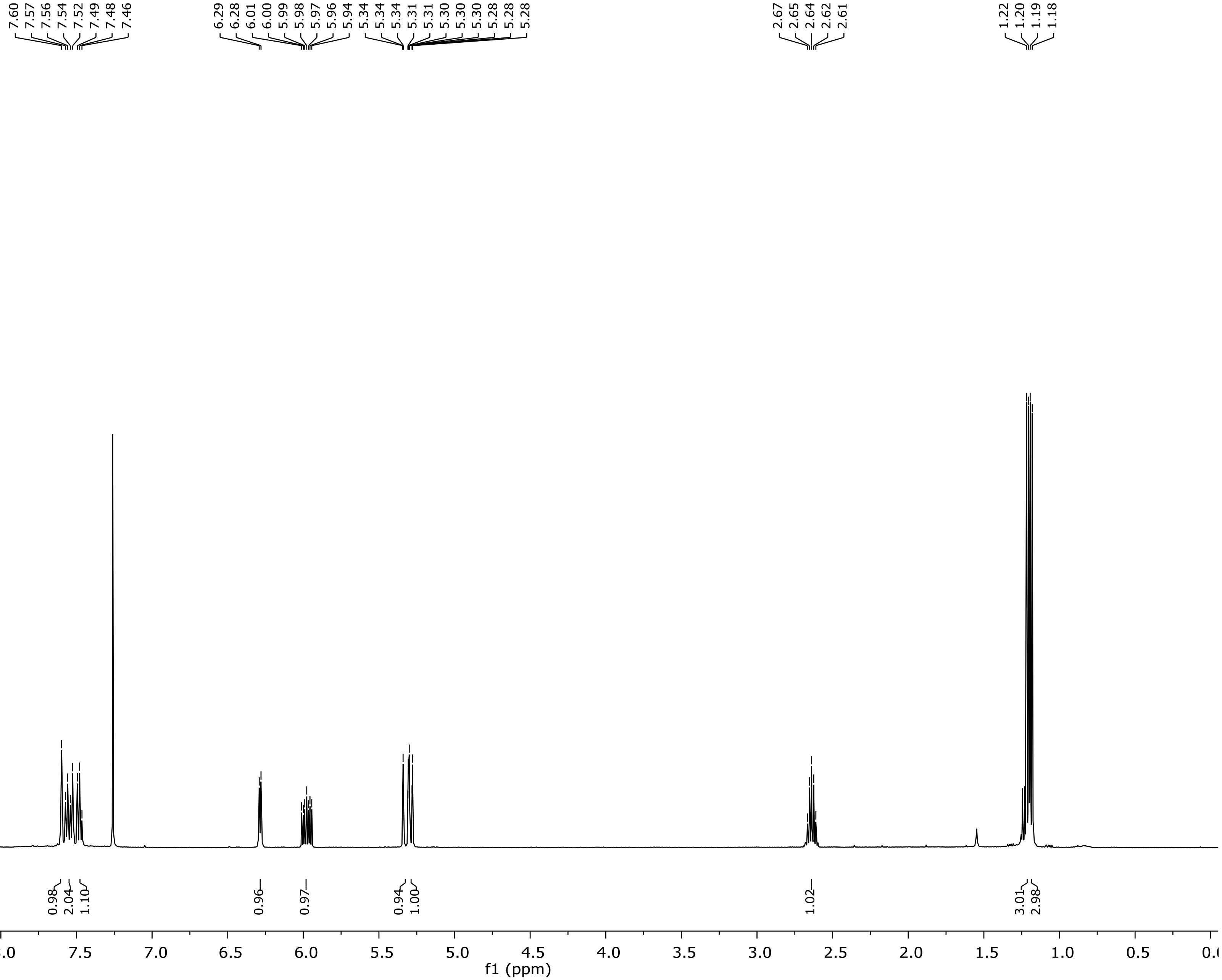

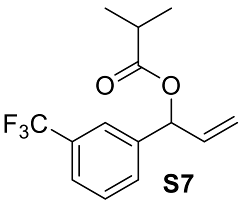

S7

^{13}C, $CDCl_3$, 126 MHz

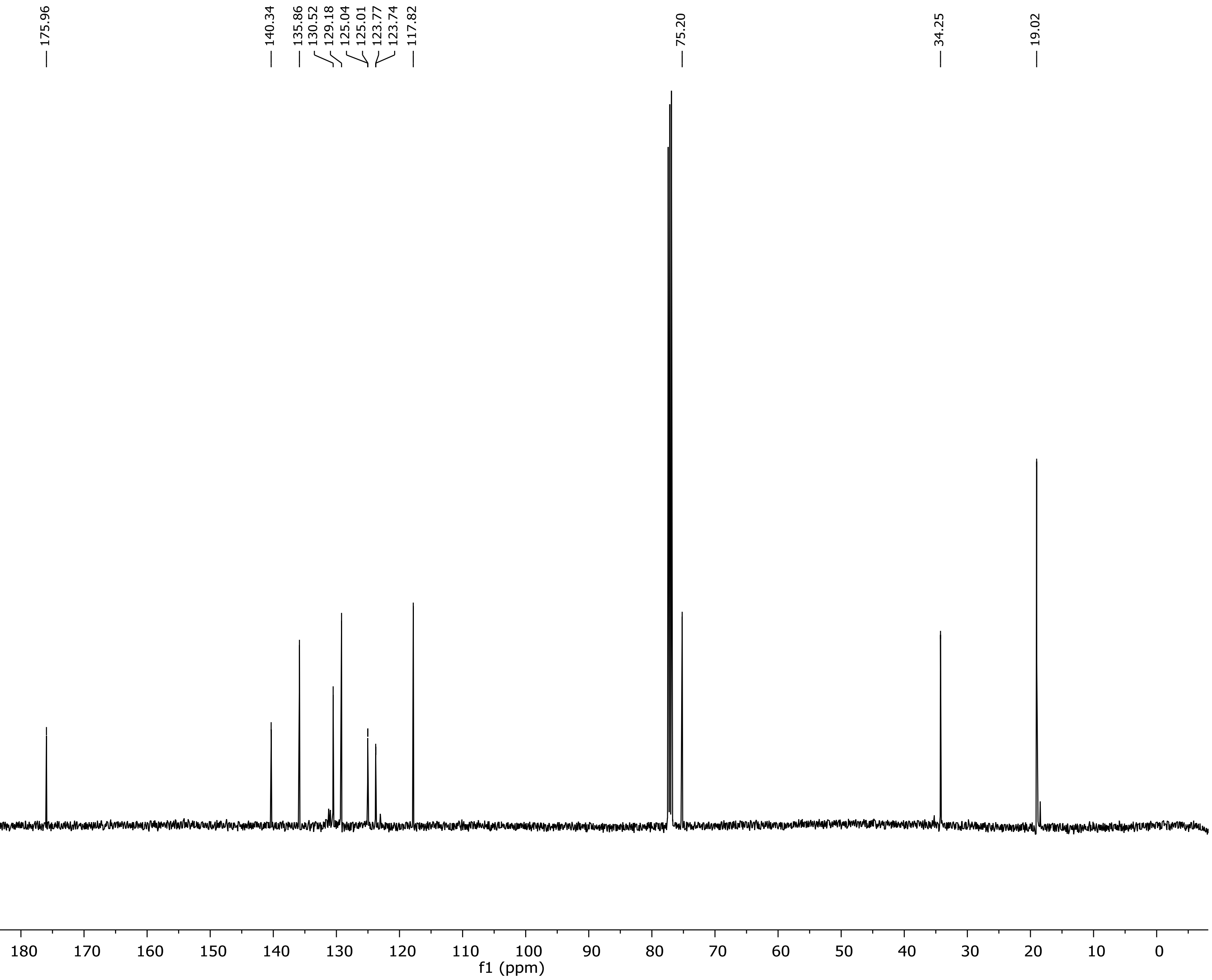

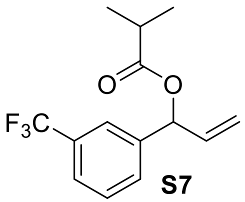

S7

^{19}F, $CDCl_3$, 376 MHz

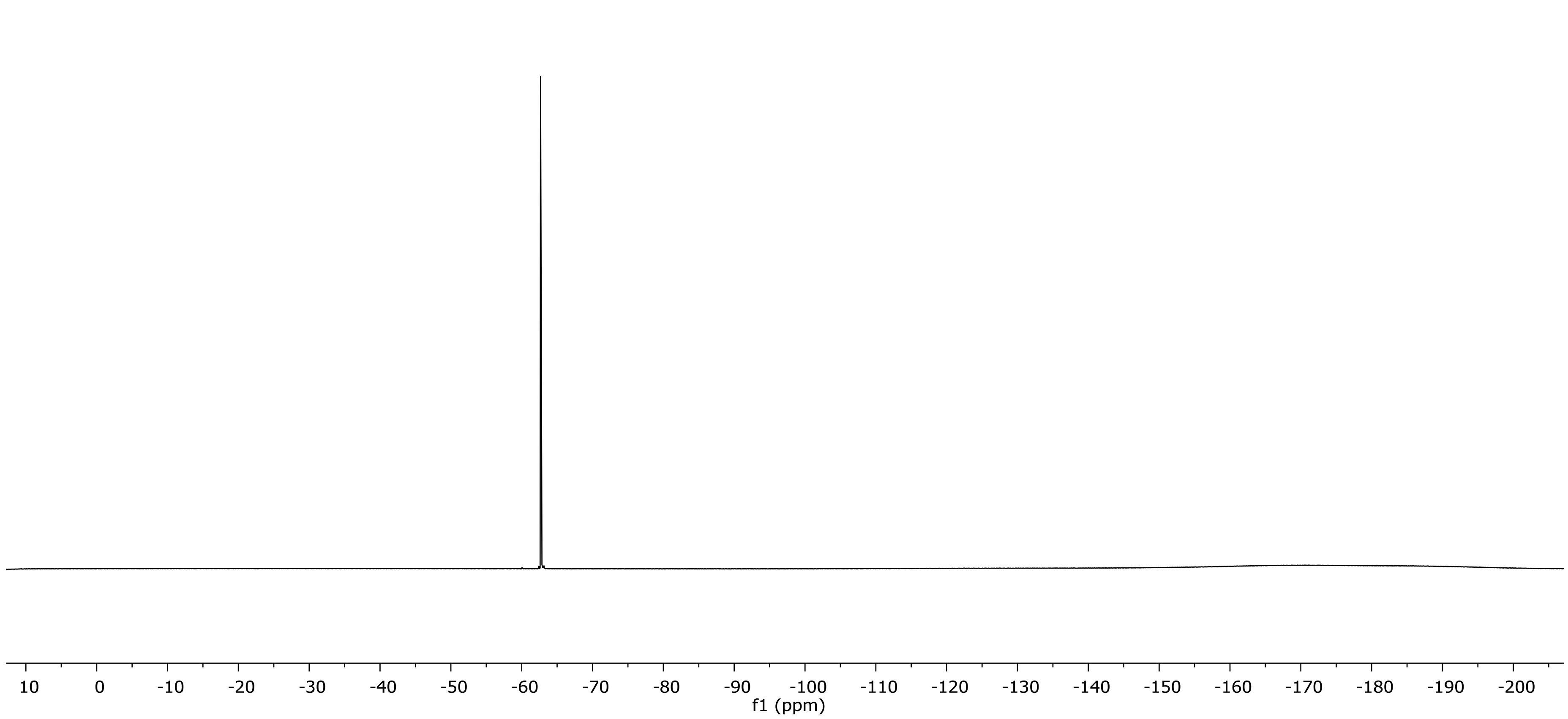

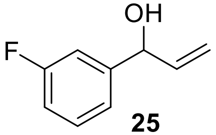

25

^{1}H, $CDCl_3$, 400 MHz

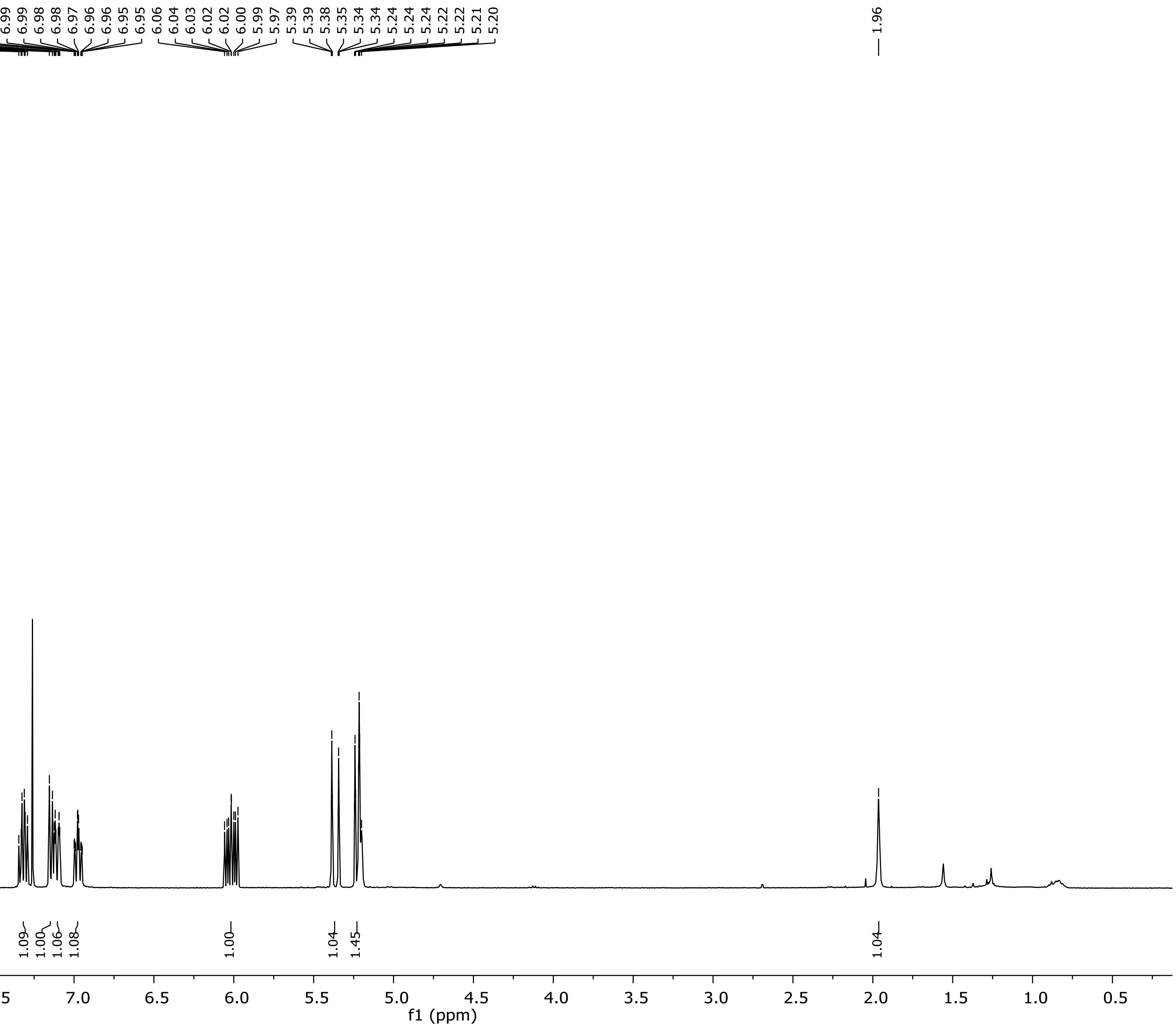

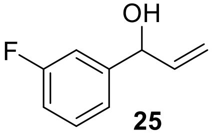

25

^{13}C, $CDCl_3$, 126 MHz

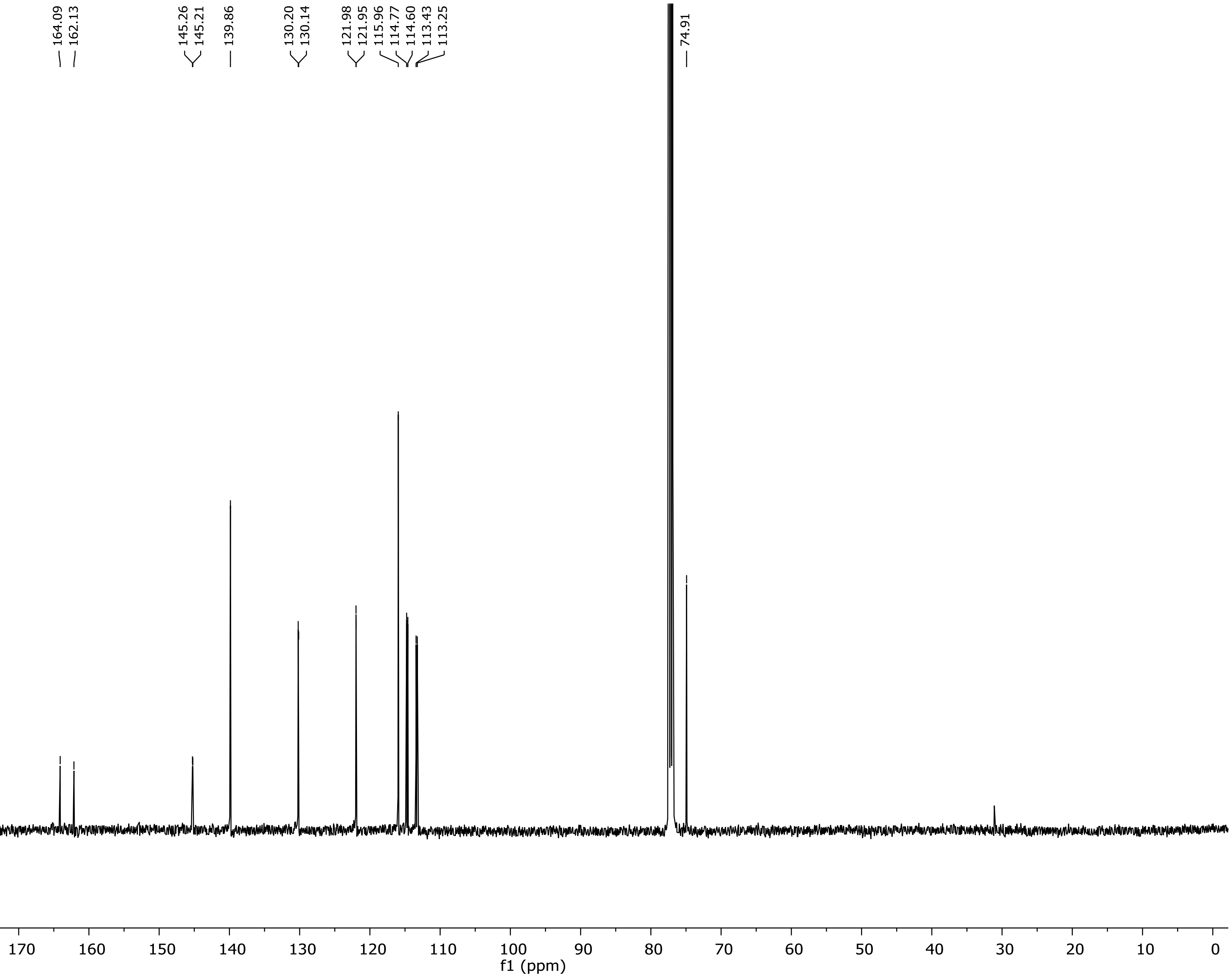

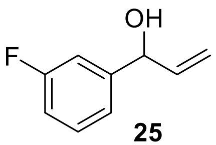

25

^{19}F, $CDCl_3$, 377 MHz

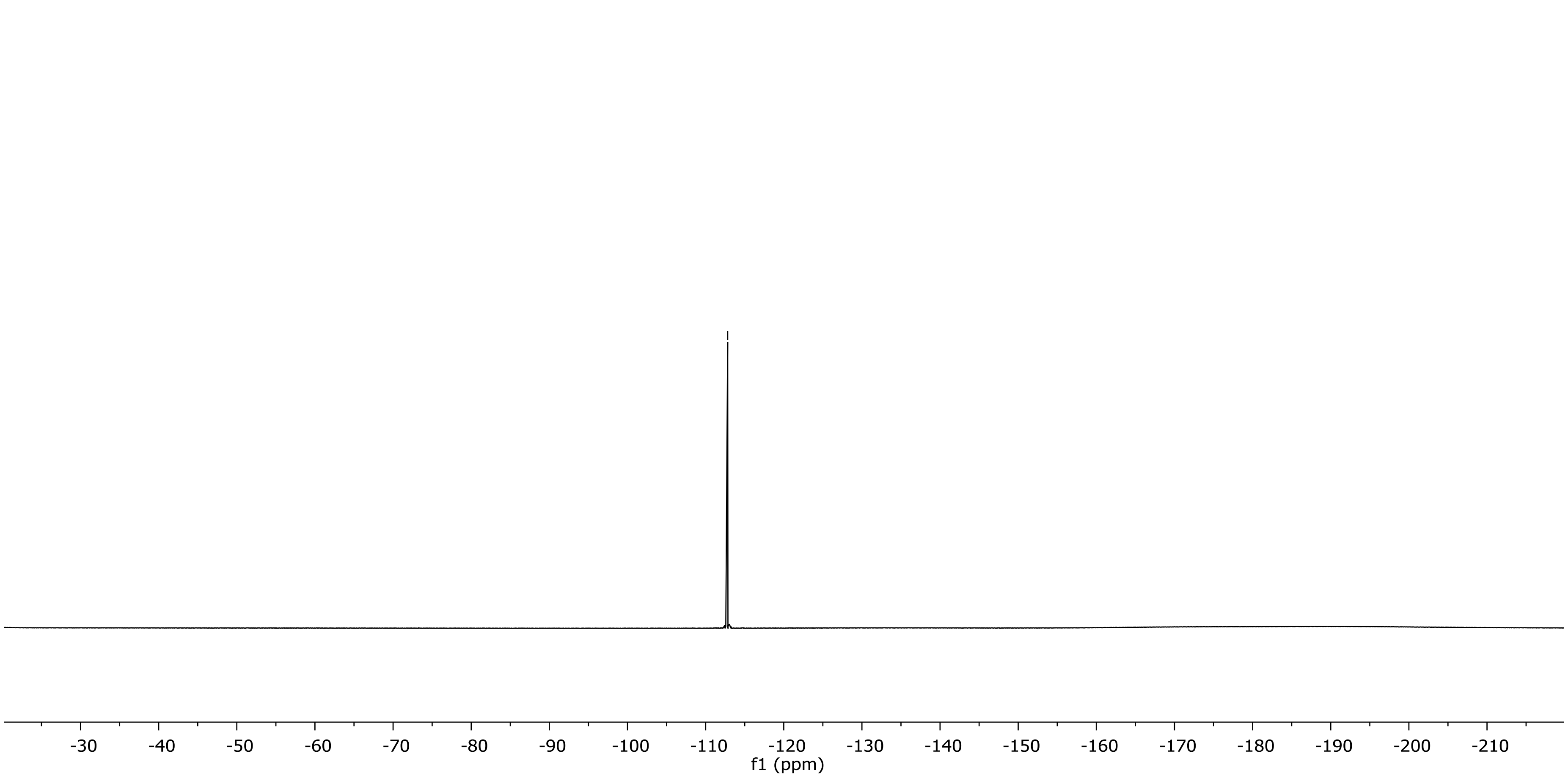

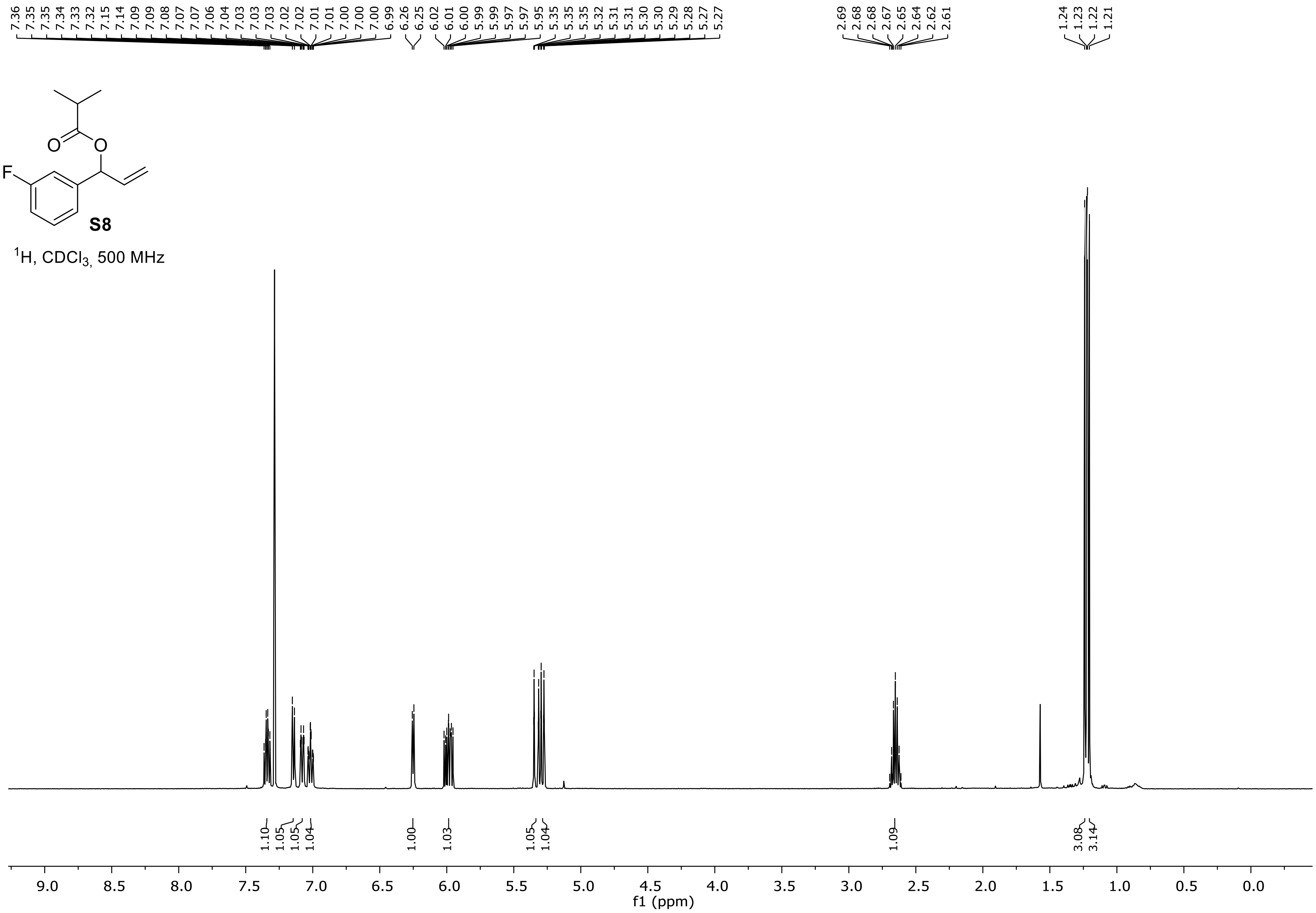
7.36 7.35 7.35 7.34 7.33 7.32 7.15 7.14 7.09 7.09 7.08 7.07 7.07 7.06 7.04 7.03 7.03 7.03 7.02 7.02 7.01 7.01 7.00 7.00 7.00 6.99 6.26 6.25 6.02 6.01 6.00 5.99 5.99 5.97 5.97 5.95 5.35 5.35 5.35 5.32 5.31 5.31 5.30 5.30 5.29 5.28 5.27 5.27
2.69 2.68 2.68 2.67 2.65 2.64 2.62 2.61
1.24 1.23 1.22 1.21
F
O
O
S8
1H, CDCl3, 500 MHz
1.10 1.05 1.05 1.04 1.00 1.03 1.05 1.04 1.09 3.08 3.14
9.0 8.5 8.0 7.5 7.0 6.5 6.0 5.5 5.0 4.5 4.0 3.5 3.0 2.5 2.0 1.5 1.0 0.5 0.0
f1 (ppm)

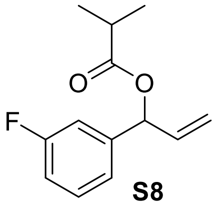

^{13}C, $CDCl_3$, 126 MHz

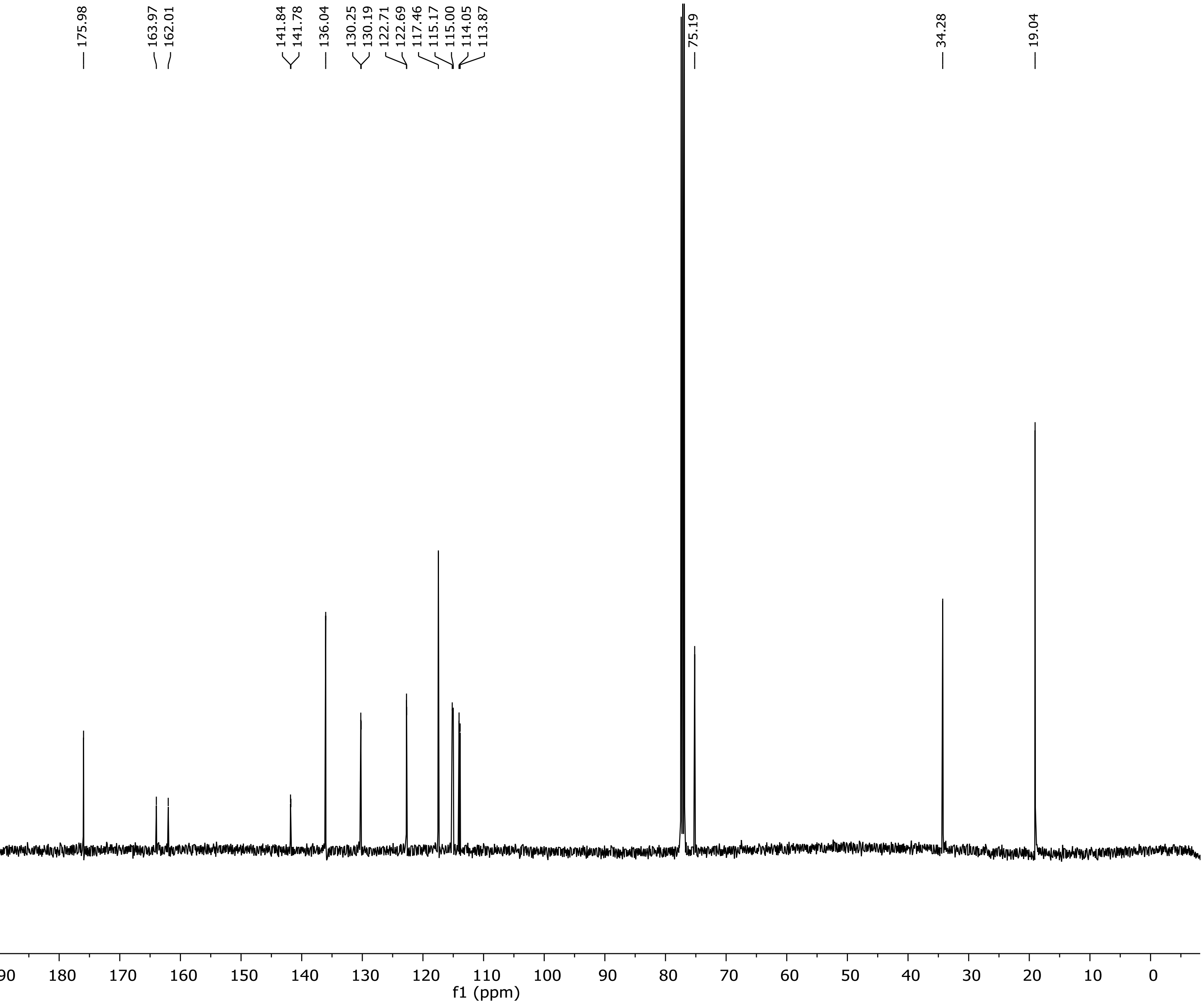

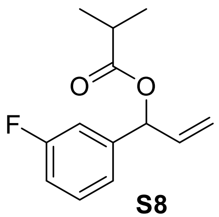

S8

^{19}F, $CDCl_3$, 376 MHz

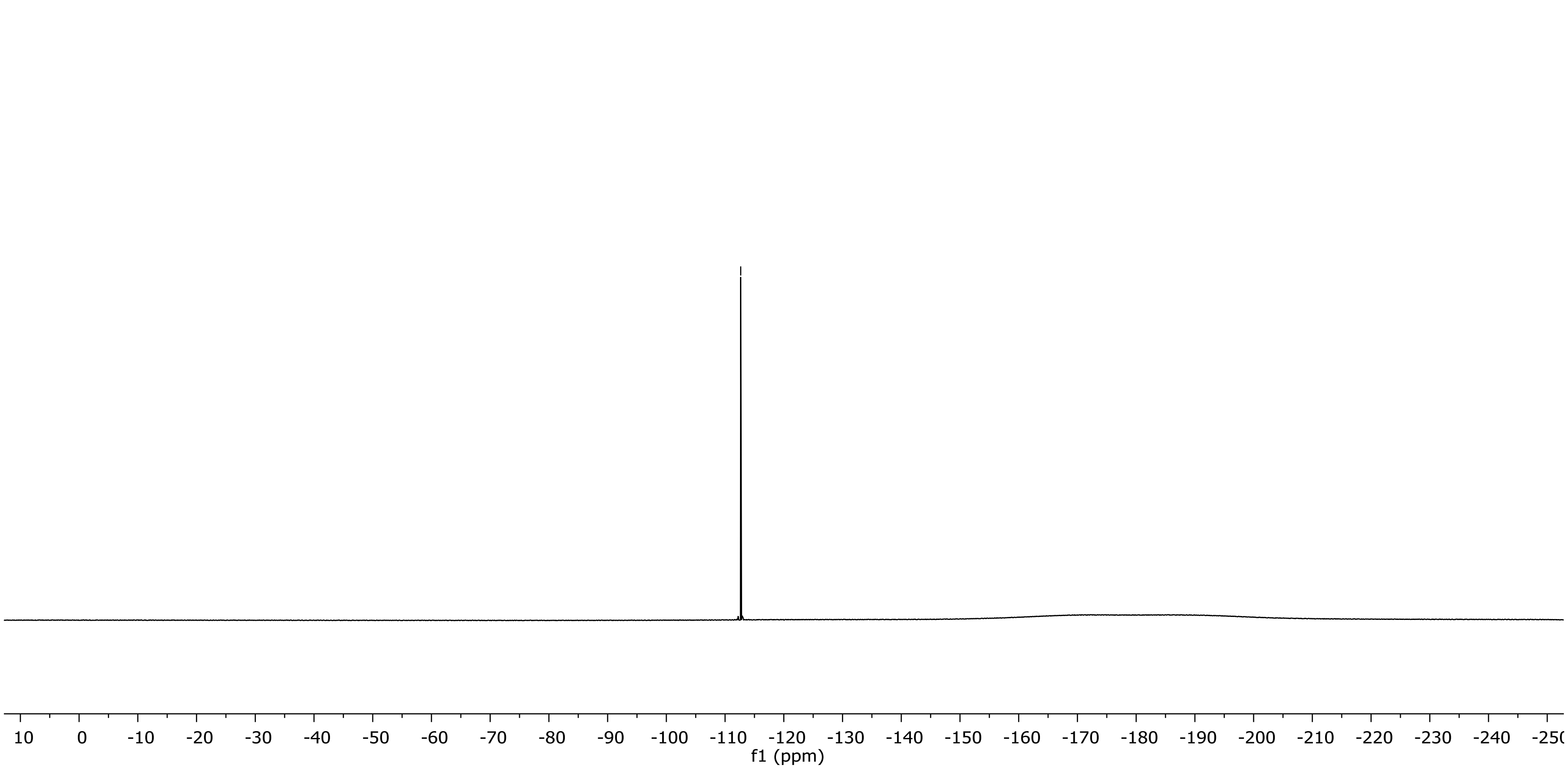

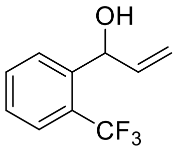

S10

^{1}H, $CDCl_3$, 500 MHz

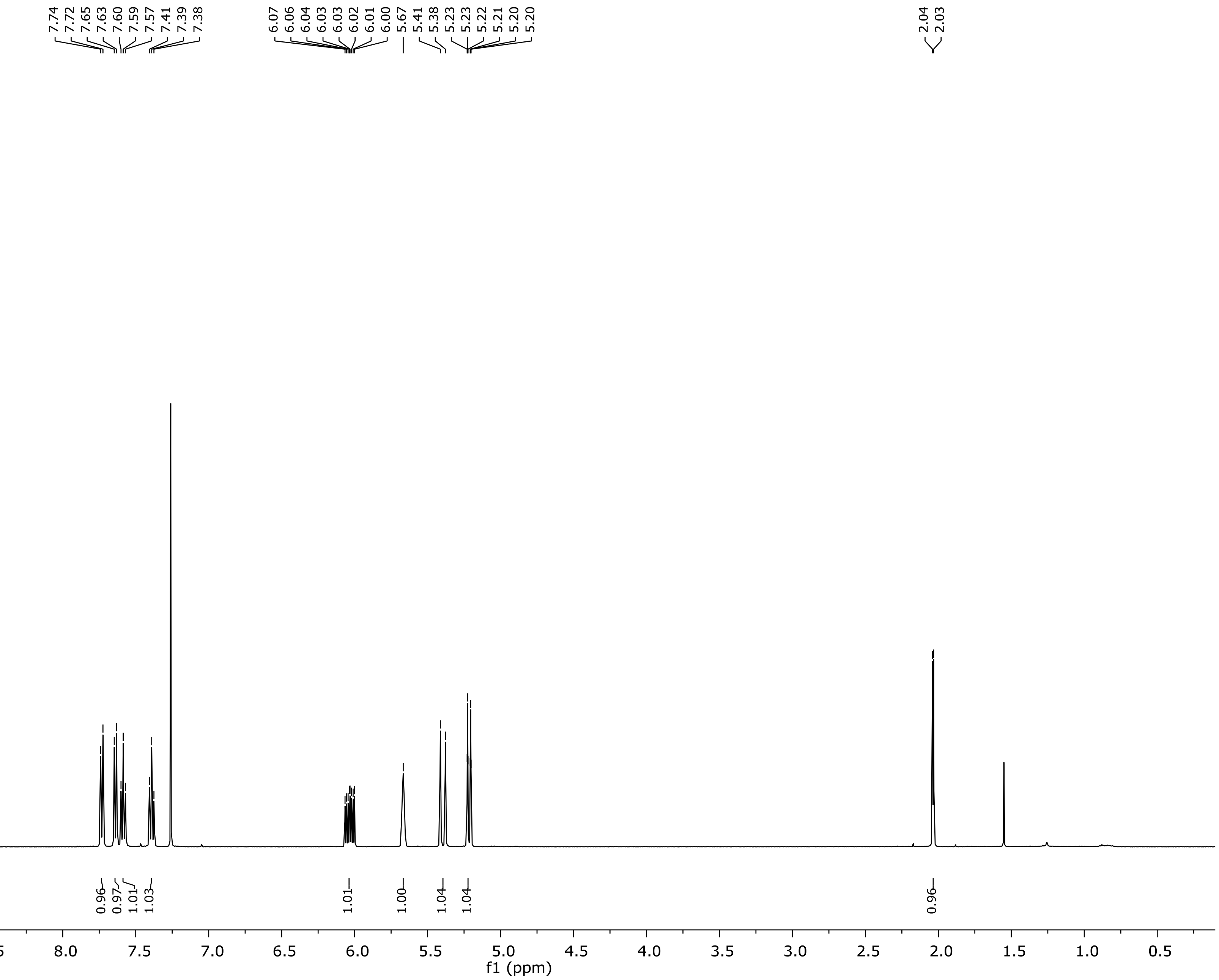

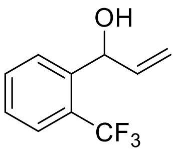

S10

^{13}C, $CDCl_3$, 126 MHz

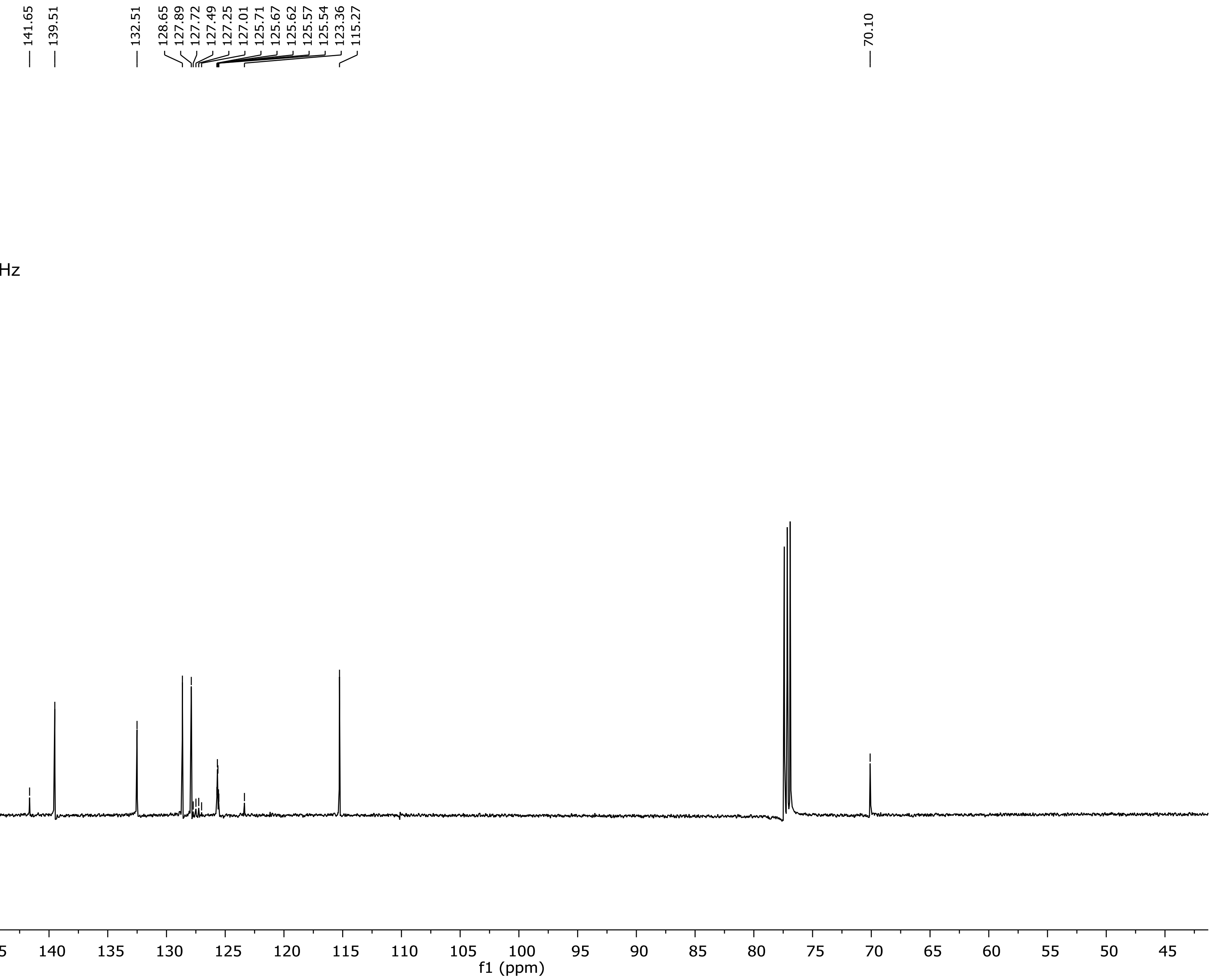

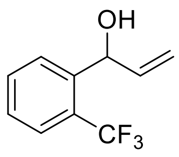

S10

^{19}F, $CDCl_3$, 376 MHz

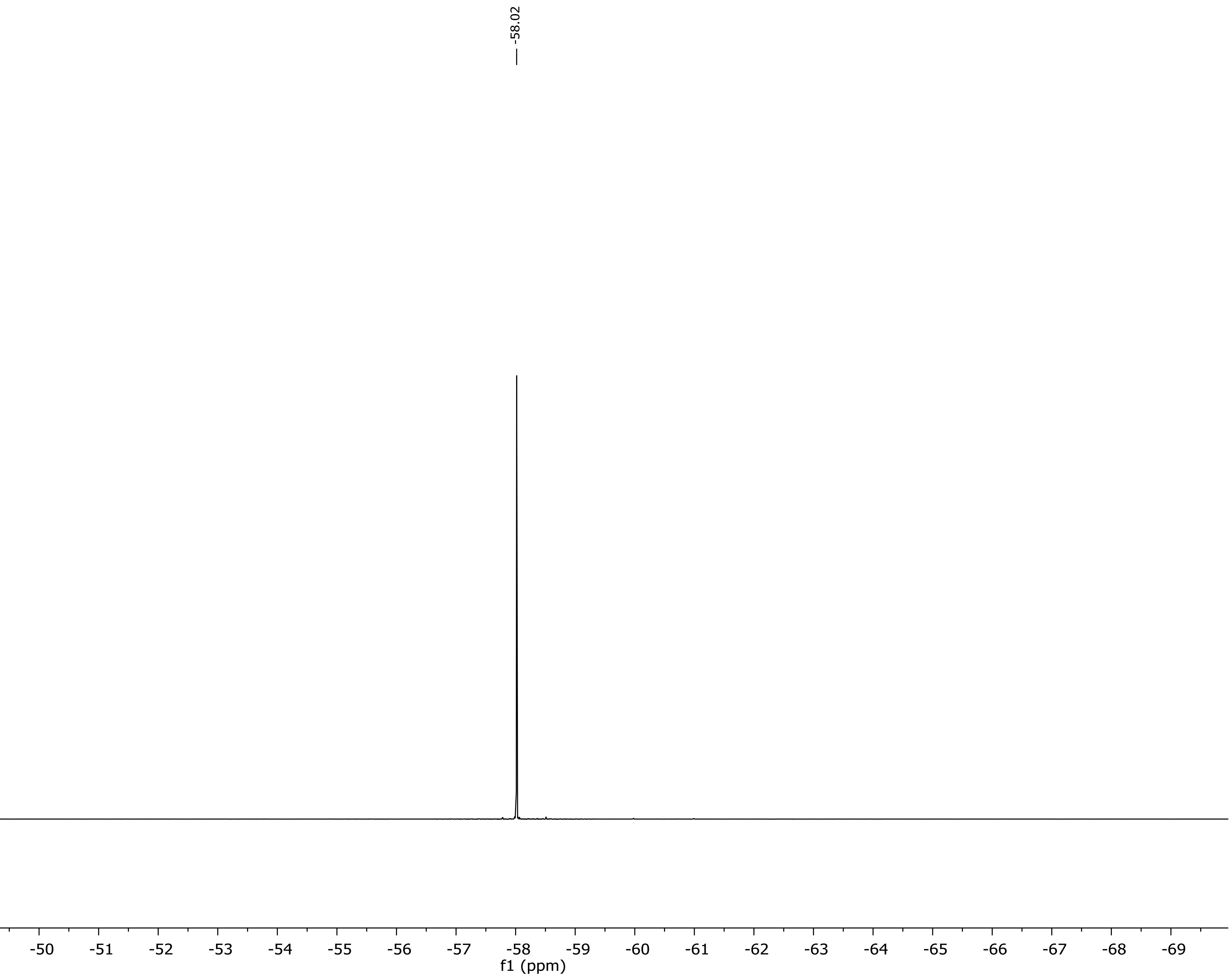

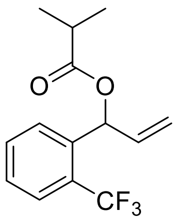

S10

^{1}H, $CDCl_3$, 500 MHz

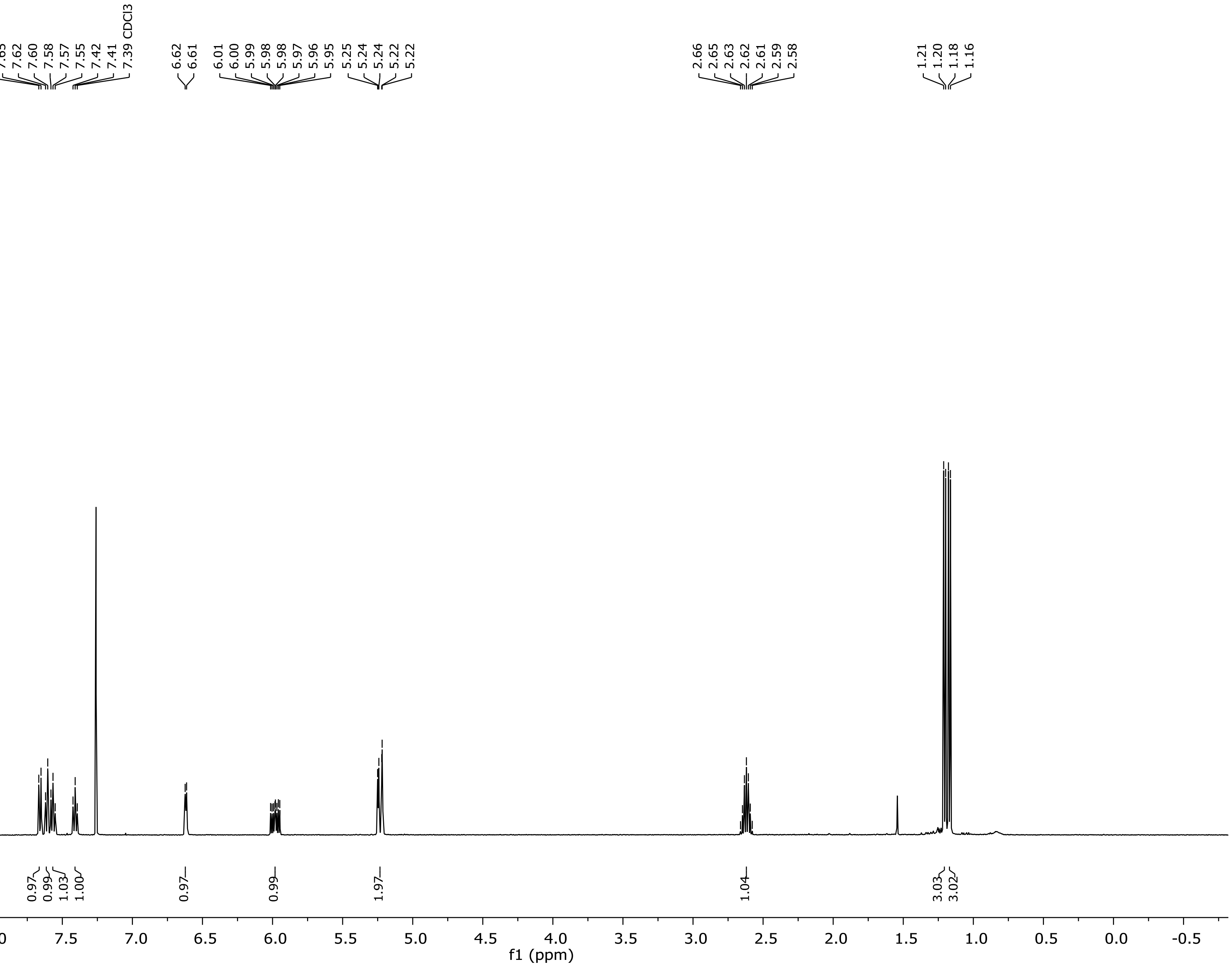

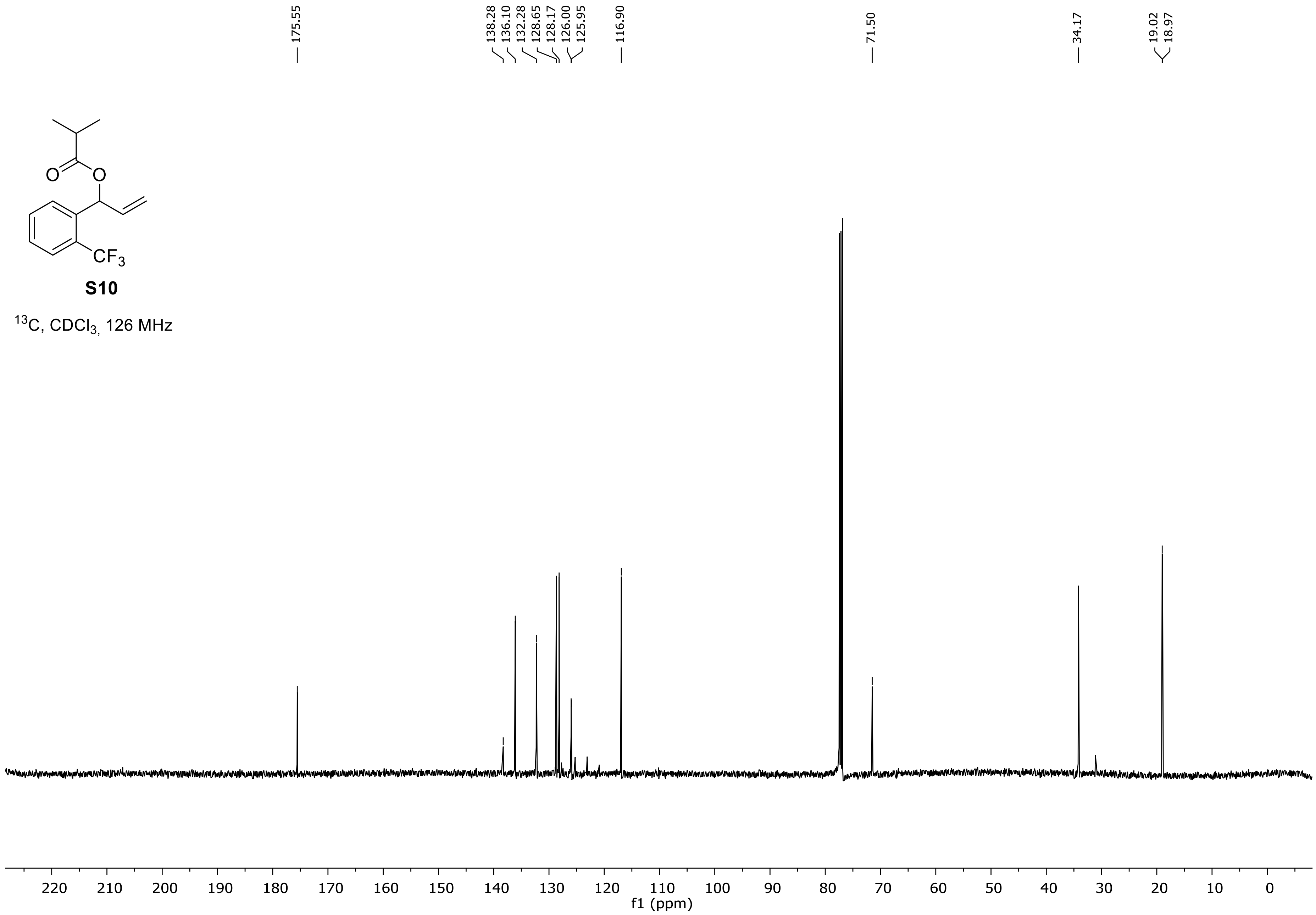
175.55
138.28
136.10
132.28
128.65
128.17
126.00
125.95
116.90
71.50
34.17
19.02
18.97
O
O
CF3
S10
13C, CDCl3, 126 MHz
220 210 200 190 180 170 160 150 140 130 120 110 100 90 80 70 60 50 40 30 20 10 0
f1 (ppm)

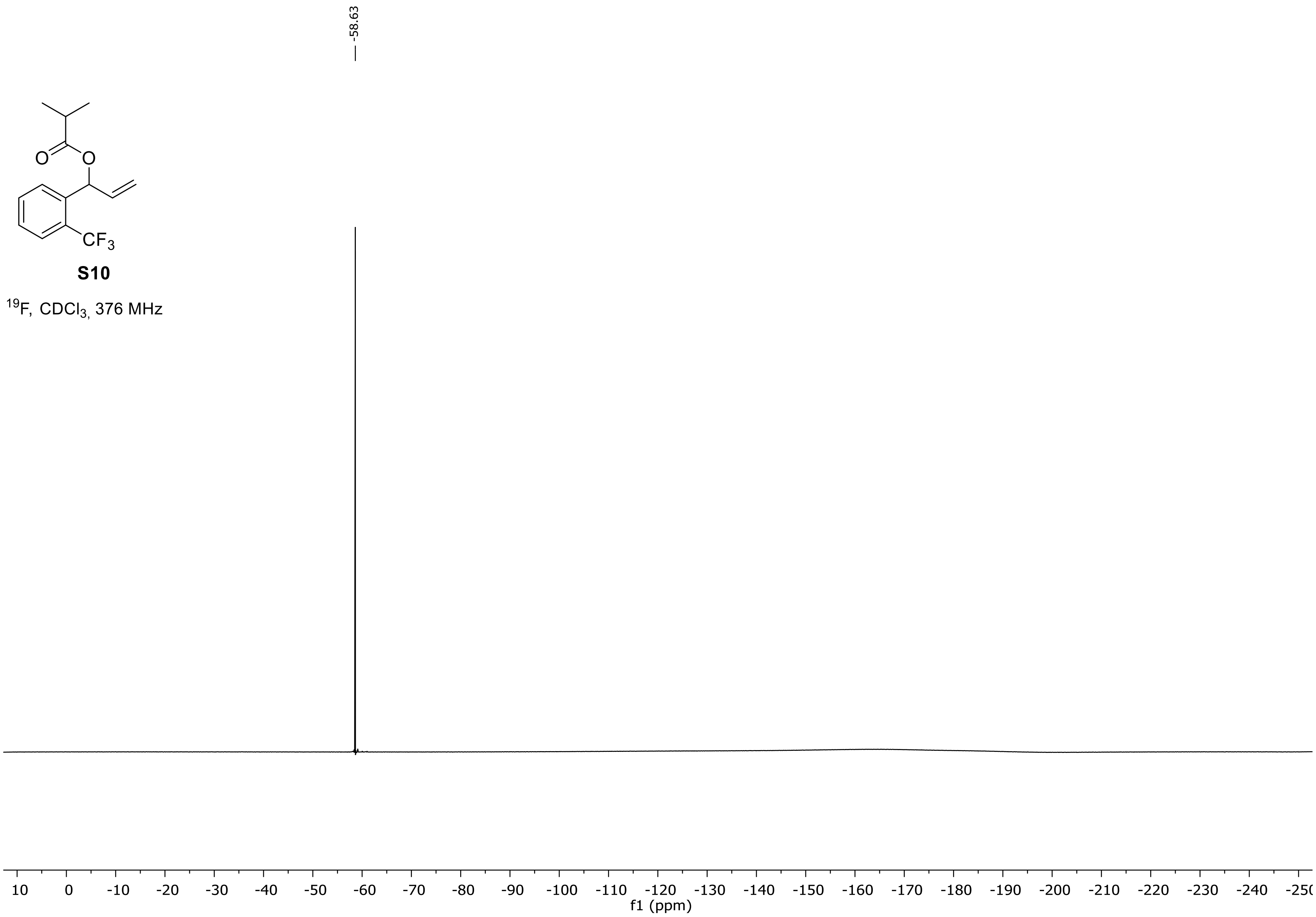

S10

^{19}F, $CDCl_3$, 376 MHz

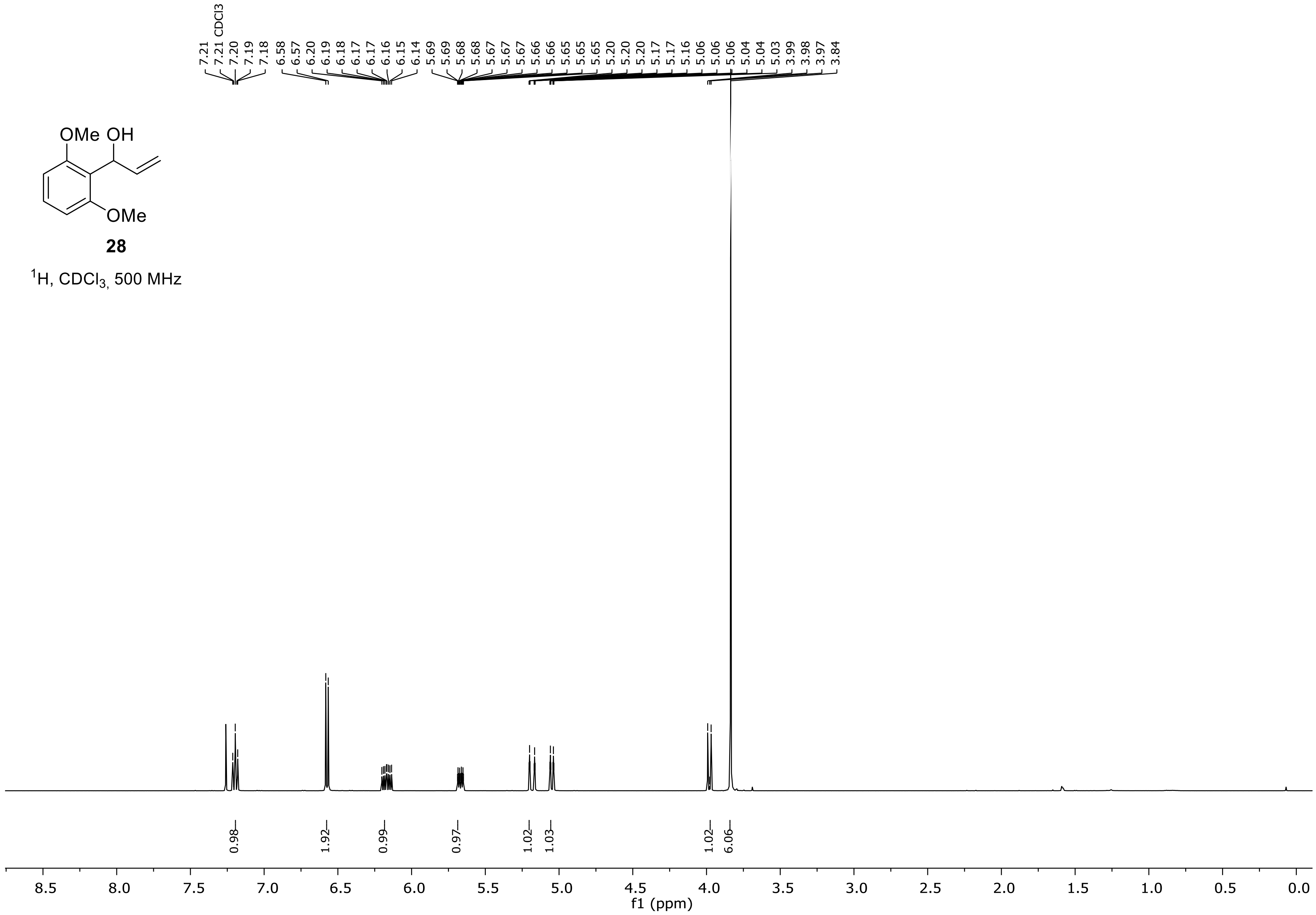

28

^{1}H, $CDCl_3$, 500 MHz

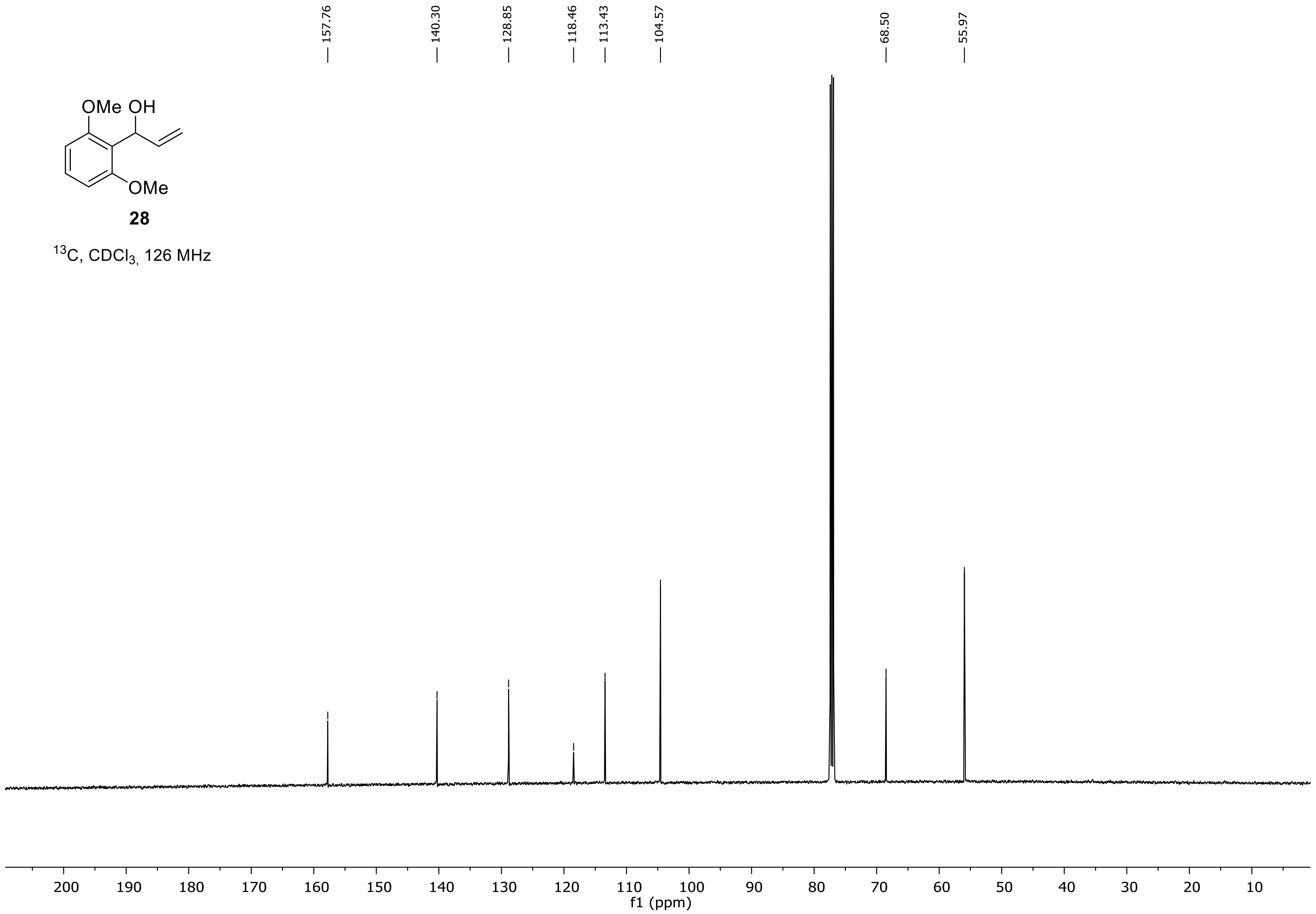

157.76
140.30
128.85
118.46
113.43
104.57
68.50
55.97
OMe OH
OMe
28
^{13}C, CDCl$_3$, 126 MHz
200 190 180 170 160 150 140 130 120 110 100 90 80 70 60 50 40 30 20 10
f1 (ppm)

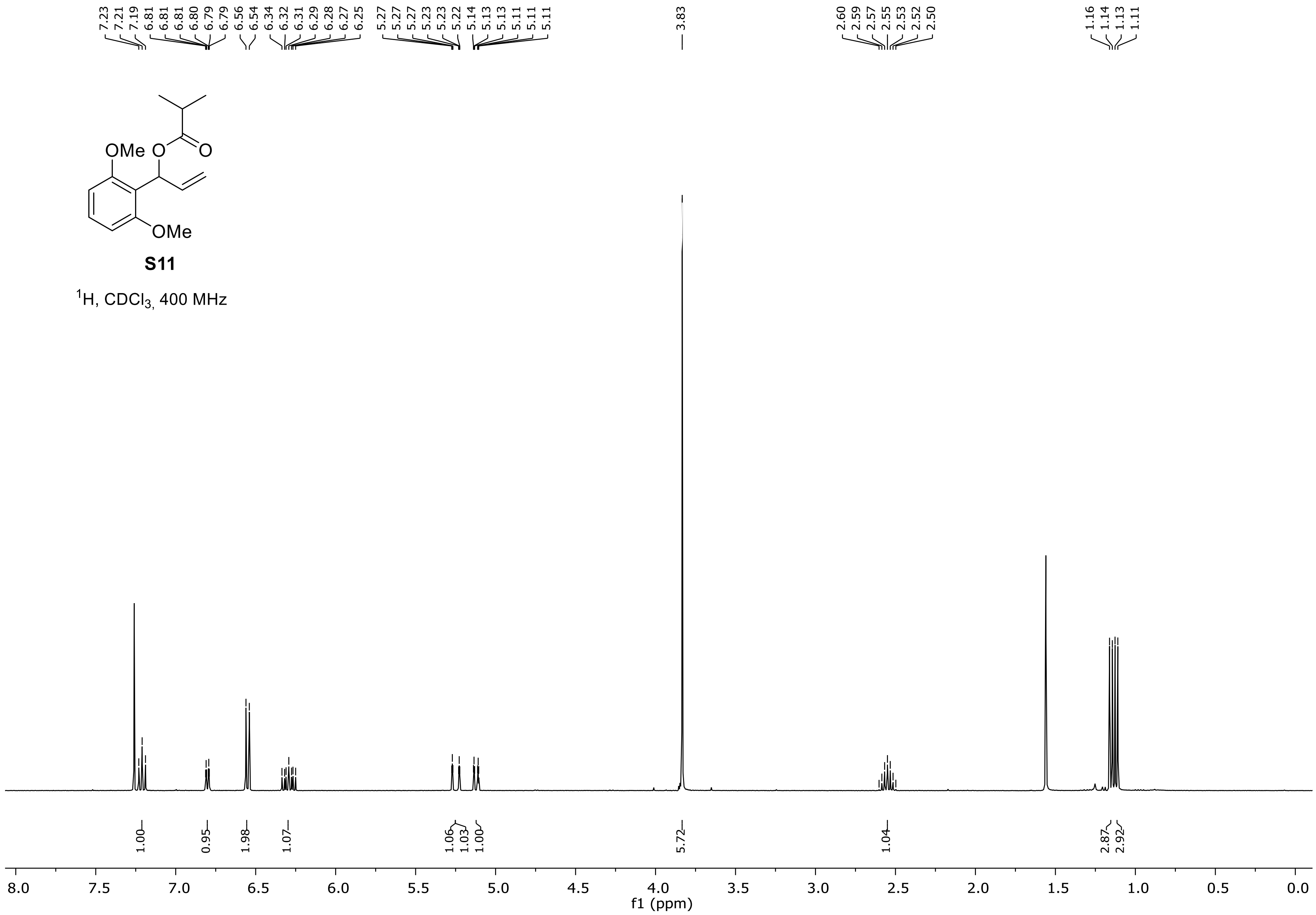
7.23
7.21
7.19
6.81
6.81
6.80
6.79
6.79
6.56
6.54
6.34
6.32
6.31
6.29
6.28
6.27
6.25
5.27
5.27
5.27
5.23
5.23
5.22
5.14
5.13
5.13
5.11
5.11
5.11
3.83
2.60
2.59
2.57
2.55
2.53
2.52
2.50
1.16
1.14
1.13
1.11
OMe
O
O
OMe
S11
^{1}H, $CDCl_3$, 400 MHz
1.00
0.95
1.98
1.07
1.06
1.03
1.00
5.72
1.04
2.87
2.92
8.0
7.5
7.0
6.5
6.0
5.5
5.0
4.5
4.0
3.5
3.0
2.5
2.0
1.5
1.0
0.5
0.0
f1 (ppm)

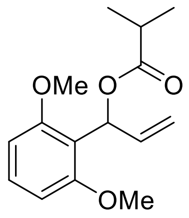

S11

^{13}C, $CDCl_3$, 126 MHz

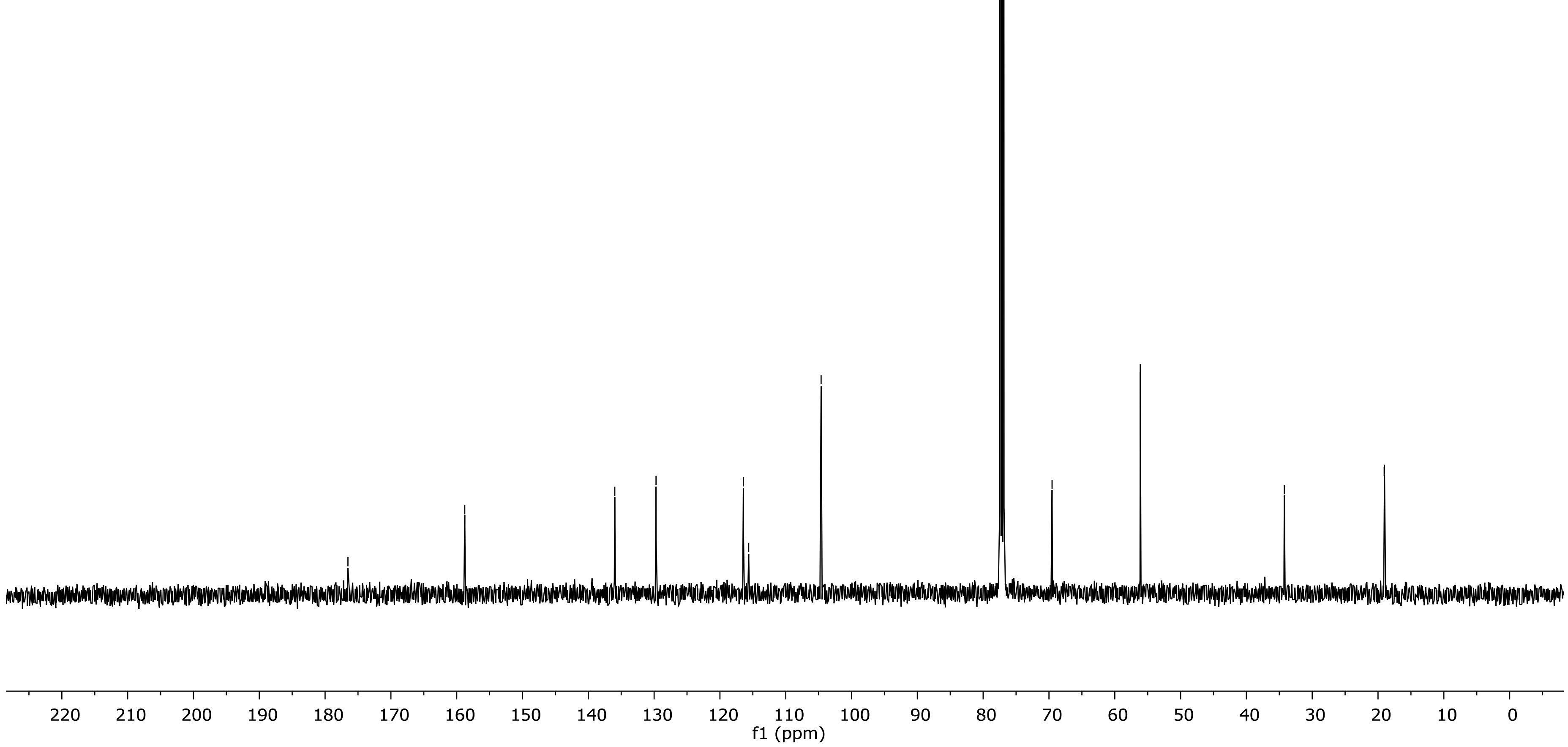

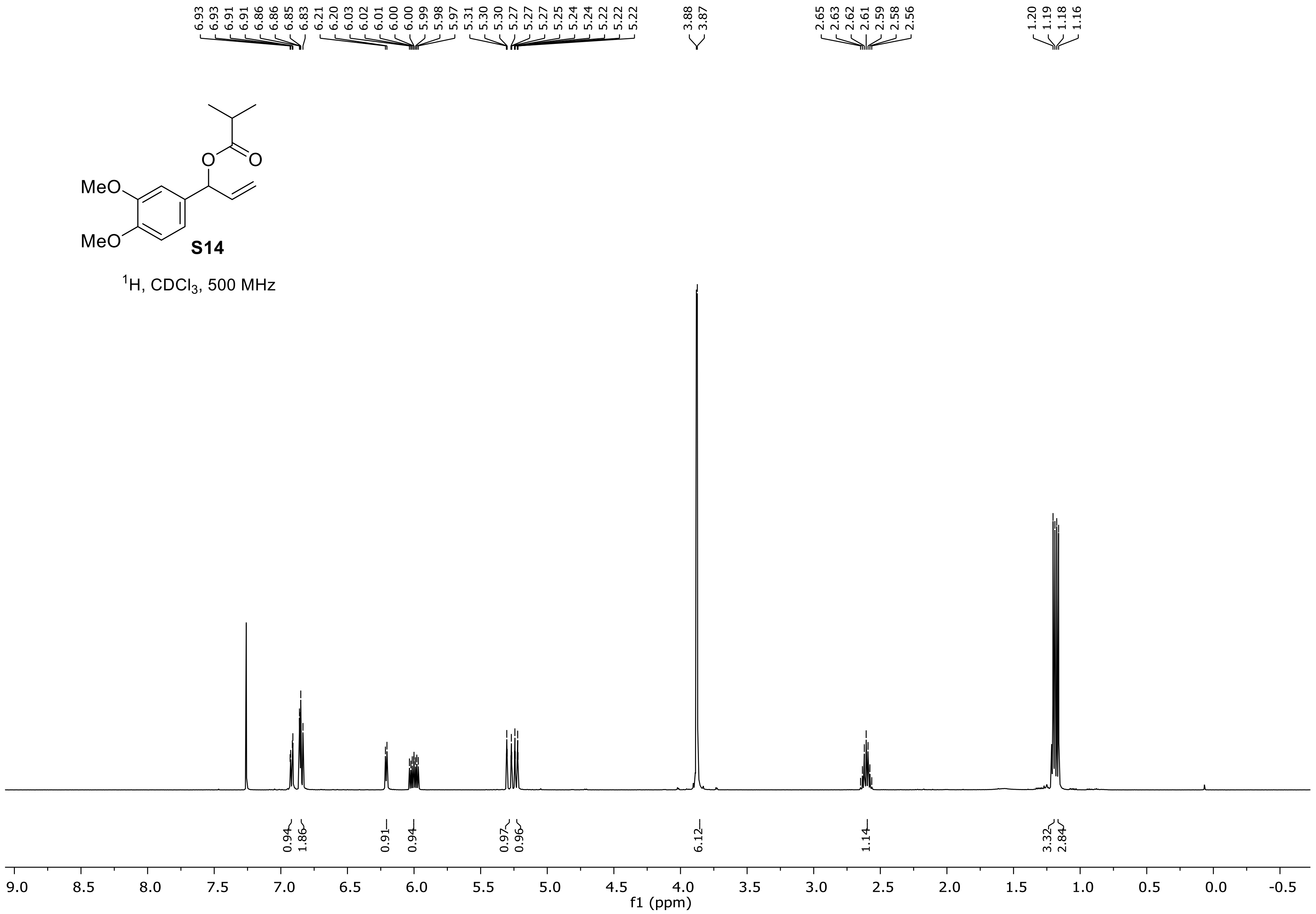
MeO
MeO
S14
1H, CDCl3, 500 MHz
6.93 6.93 6.91 6.91 6.86 6.86 6.85 6.83 6.21 6.20 6.03 6.02 6.01 6.00 6.00 5.99 5.98 5.97 5.31 5.30 5.27 5.27 5.27 5.25 5.24 5.24 5.22 5.22 5.22 3.88 3.87 2.65 2.63 2.62 2.61 2.59 2.58 2.56 1.20 1.19 1.18 1.16
0.94 1.86 0.91 0.94 0.97 0.96 6.12 1.14 3.32 2.84
9.0 8.5 8.0 7.5 7.0 6.5 6.0 5.5 5.0 4.5 4.0 3.5 3.0 2.5 2.0 1.5 1.0 0.5 0.0 -0.5
f1 (ppm)

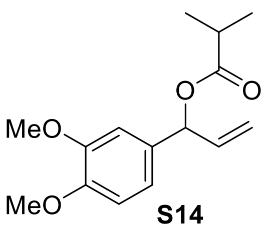

S14

^{13}C, $CDCl_3$, 126 MHz

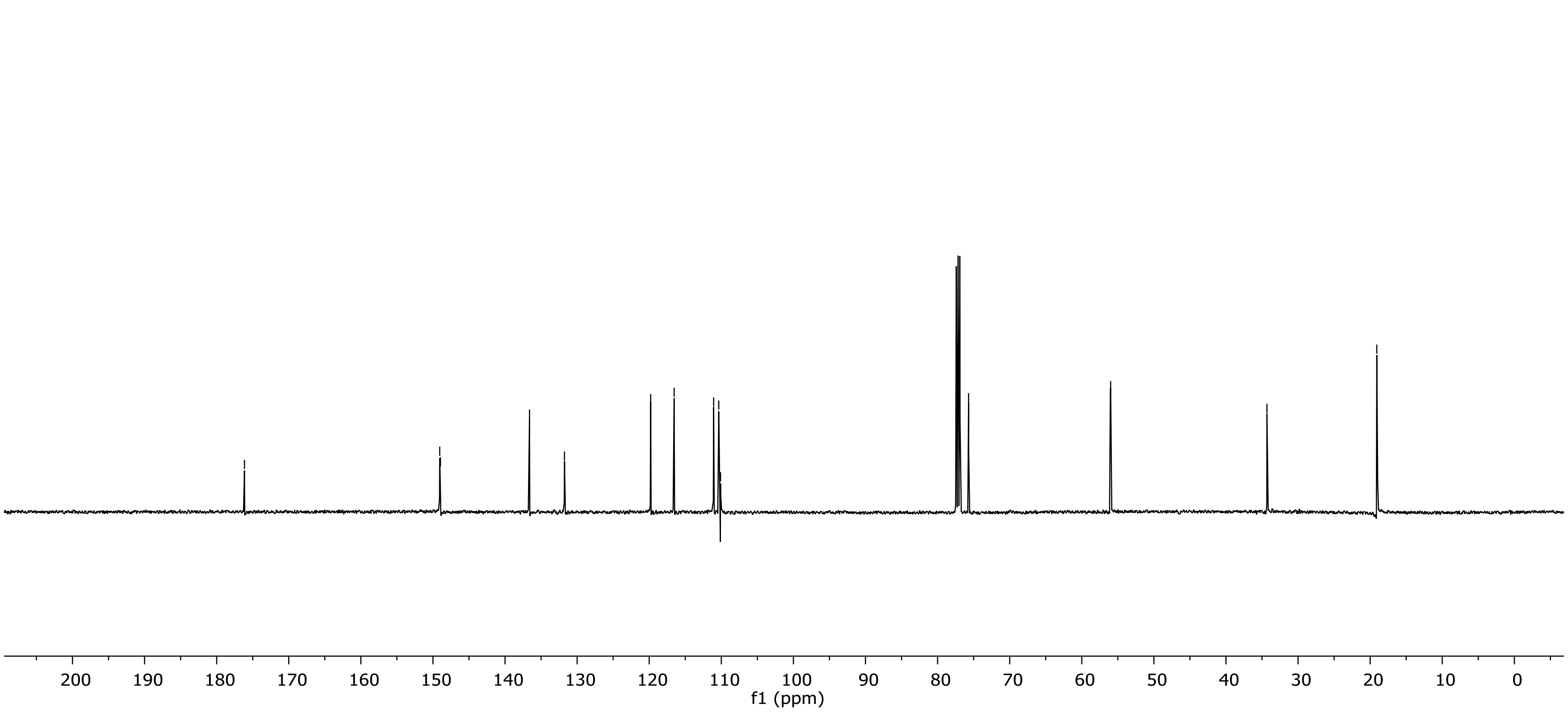

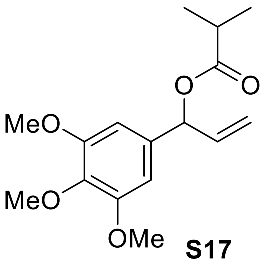

S17

^1H, $CDCl_3$, 500 MHz

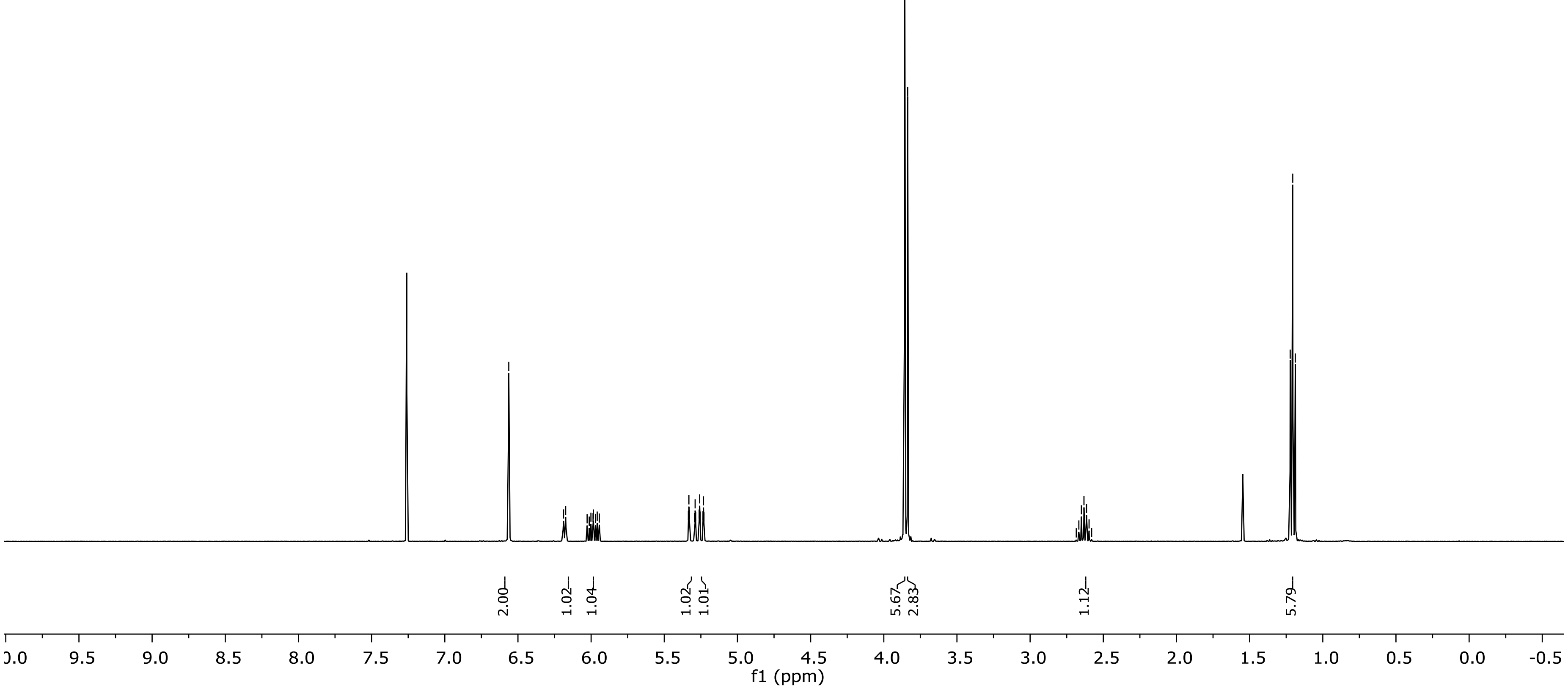

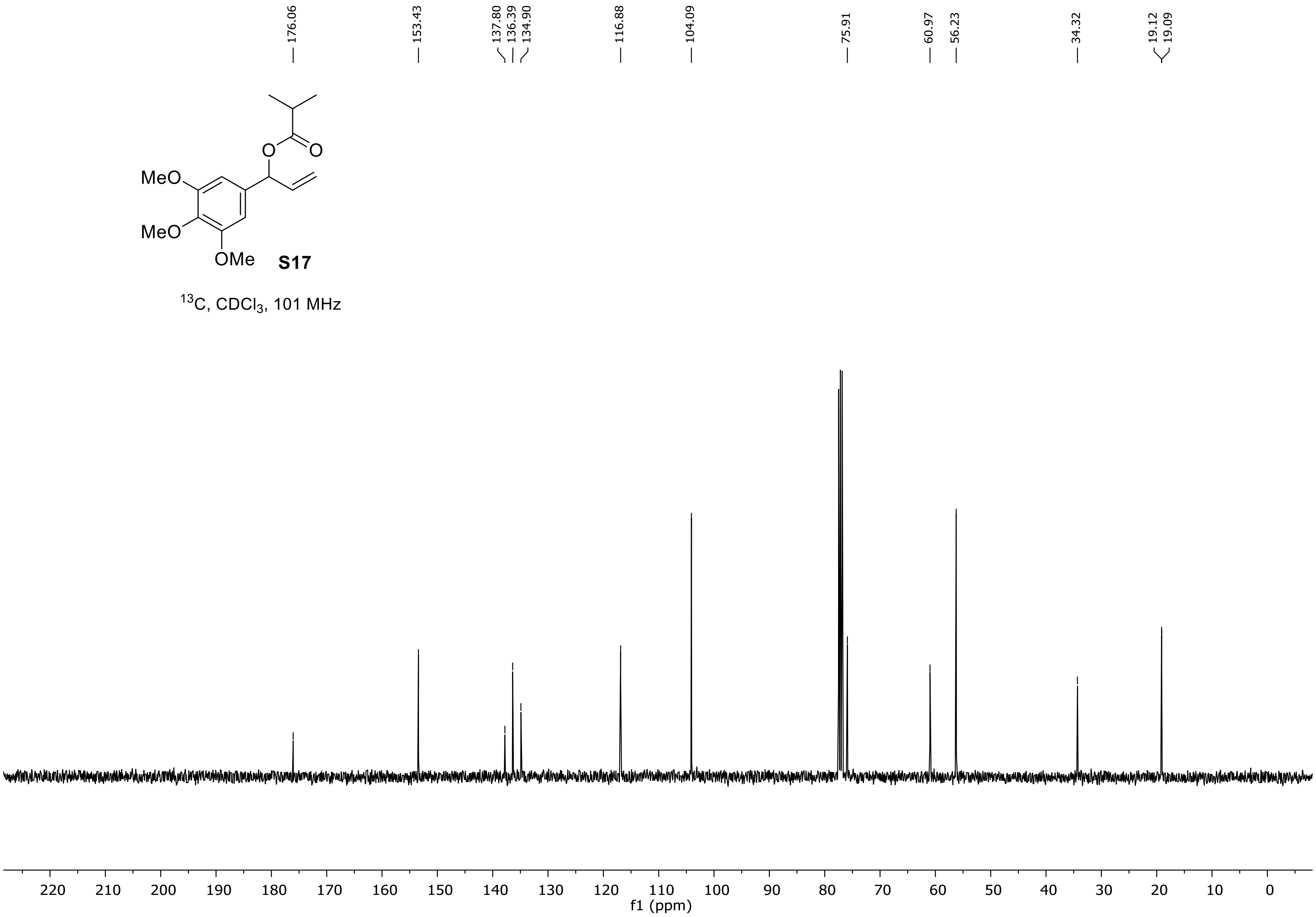
176.06
153.43
137.80
136.39
134.90
116.88
104.09
75.91
60.97
56.23
34.32
19.12
19.09
MeO
MeO
OMe
O
O
S17
^{13}C, $CDCl_3$, 101 MHz
220 210 200 190 180 170 160 150 140 130 120 110 100 90 80 70 60 50 40 30 20 10 0
f1 (ppm)

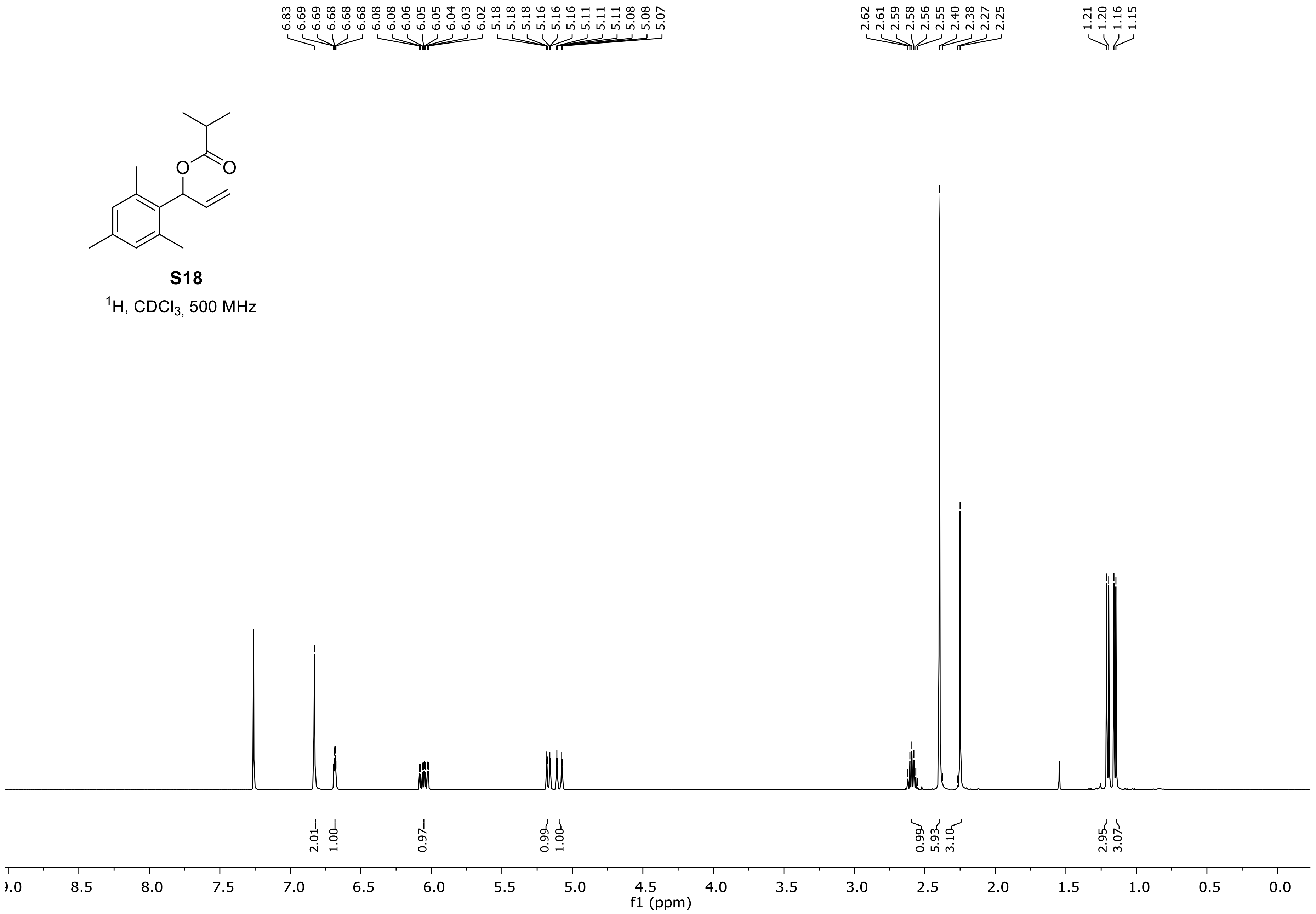
S18
1H, CDCl3, 500 MHz
f1 (ppm)

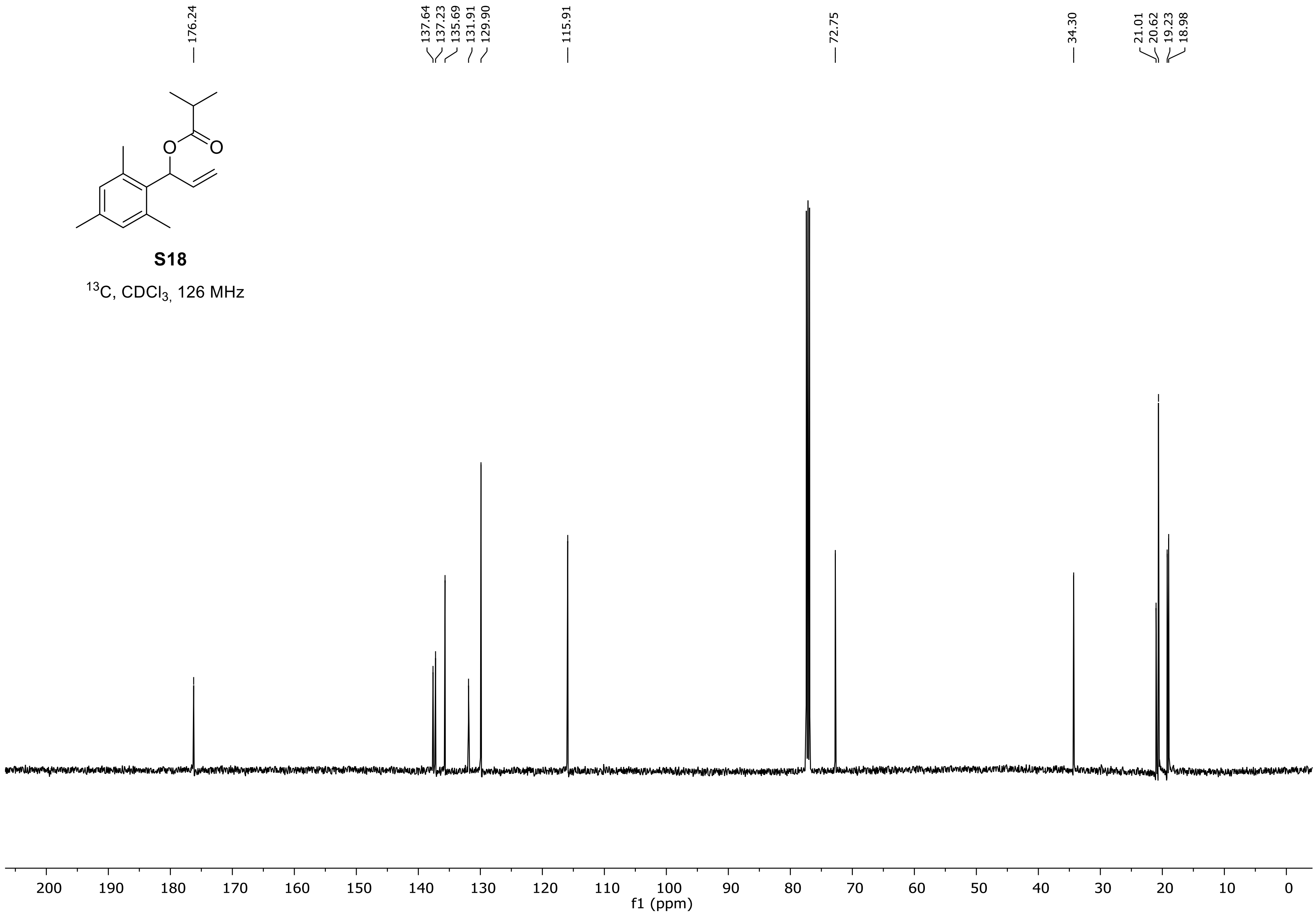
176.24
137.64
137.23
135.69
131.91
129.90
115.91
72.75
34.30
21.01
20.62
19.23
18.98
S18
13C, CDCl3, 126 MHz
200 190 180 170 160 150 140 130 120 110 100 90 80 70 60 50 40 30 20 10 0
f1 (ppm)

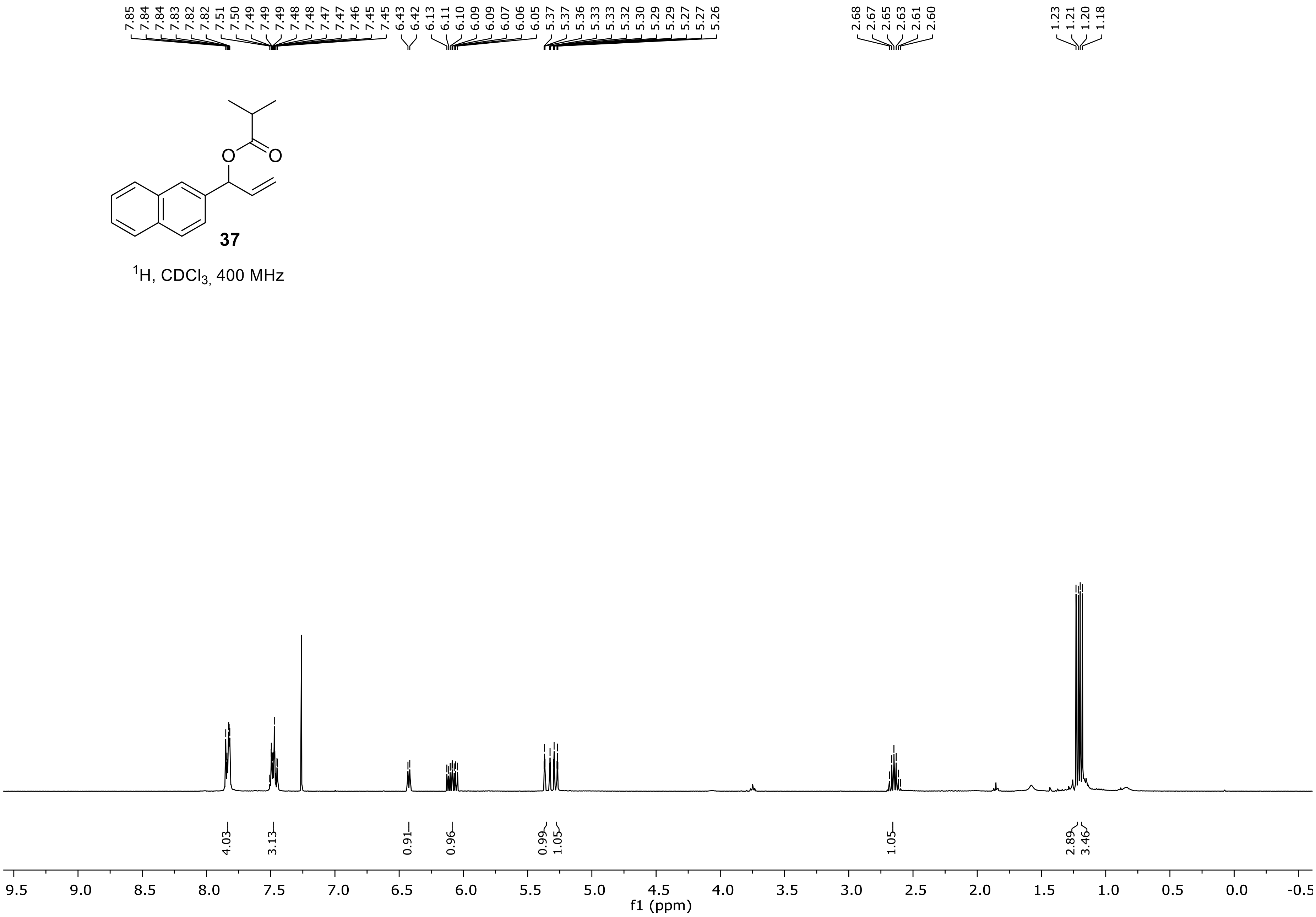

7.85 7.84 7.84 7.83 7.82 7.82 7.51 7.50 7.49 7.49 7.49 7.48 7.48 7.47 7.47 7.46 7.45 7.45 6.43 6.42 6.13 6.11 6.10 6.09 6.09 6.07 6.06 6.05 5.37 5.37 5.36 5.33 5.33 5.32 5.30 5.29 5.29 5.27 5.27 5.26
2.68 2.67 2.65 2.63 2.61 2.60
1.23 1.21 1.20 1.18
O
O
37
^{1}H, $CDCl_3$, 400 MHz
4.03 3.13 0.91 0.96 0.99 1.05 1.05 2.89 3.46
9.5 9.0 8.5 8.0 7.5 7.0 6.5 6.0 5.5 5.0 4.5 4.0 3.5 3.0 2.5 2.0 1.5 1.0 0.5 0.0 -0.5
f1 (ppm)

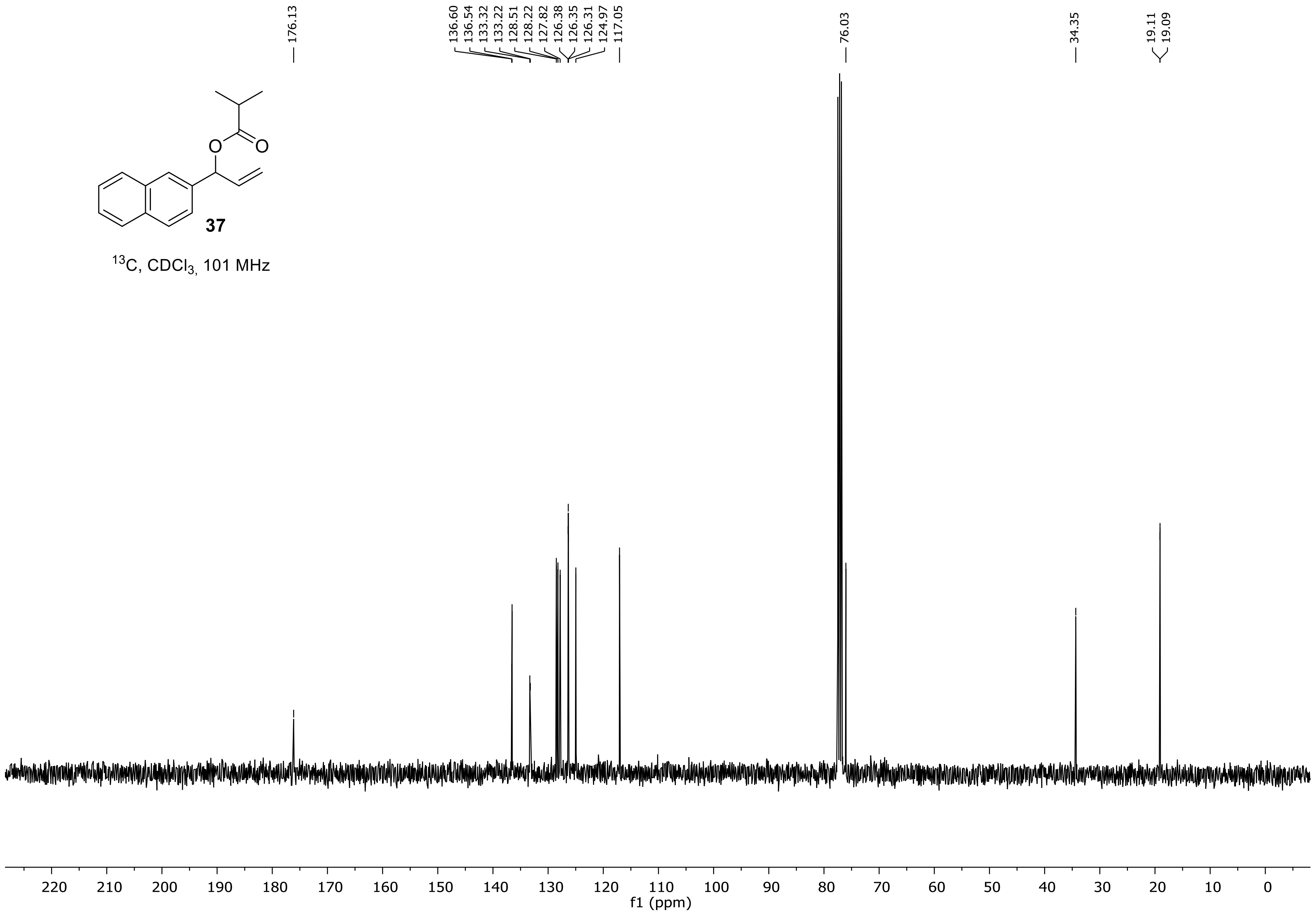
176.13
136.60
136.54
133.32
133.22
128.51
128.22
127.82
126.38
126.35
126.31
124.97
117.05
76.03
34.35
19.11
19.09
O
O
37
^{13}C, CDCl$_3$, 101 MHz
220 210 200 190 180 170 160 150 140 130 120 110 100 90 80 70 60 50 40 30 20 10 0
f1 (ppm)

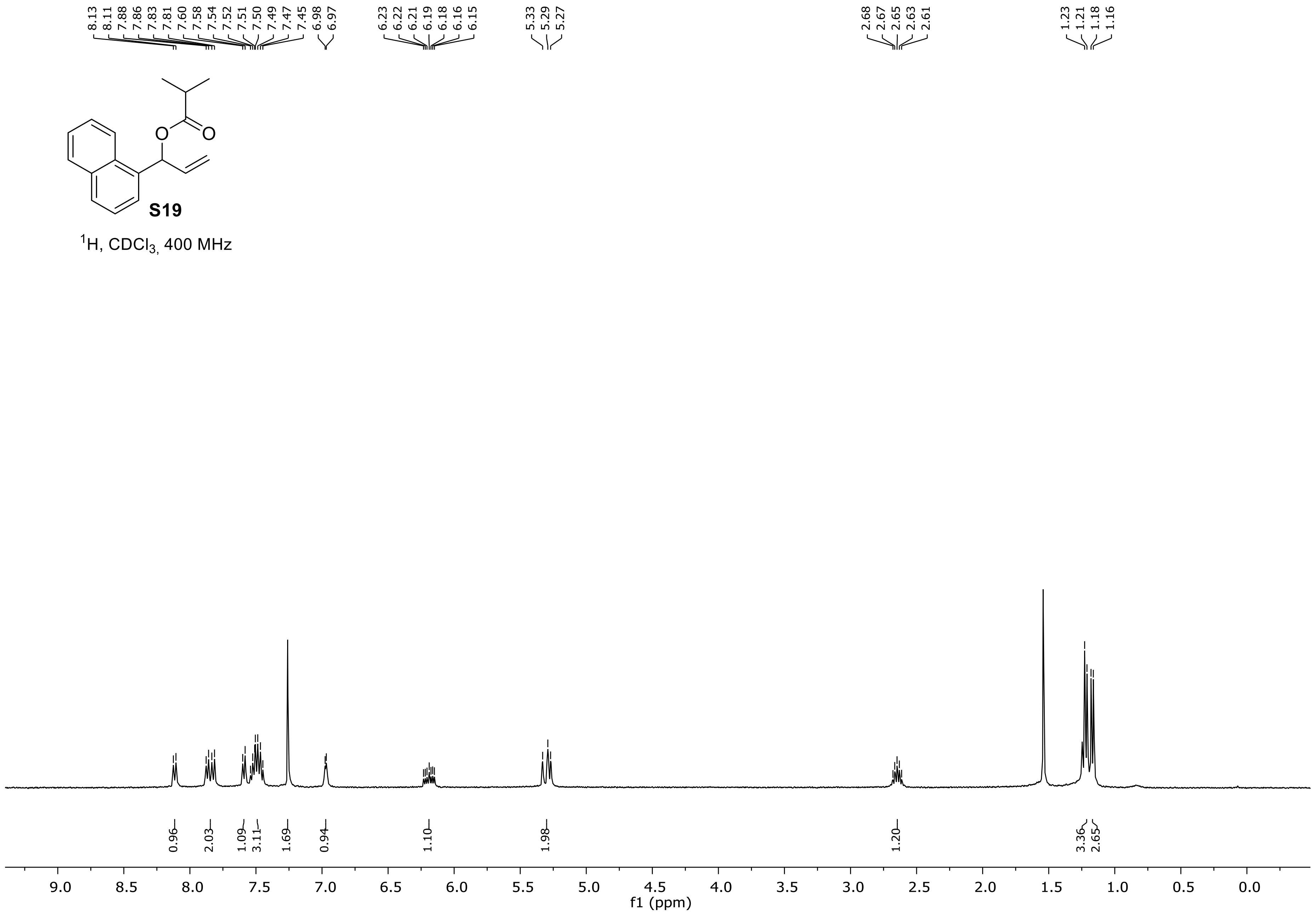
8.13 8.11 7.88 7.86 7.83 7.81 7.60 7.58 7.54 7.52 7.51 7.50 7.49 7.47 7.45 6.98 6.97
6.23 6.22 6.21 6.19 6.18 6.16 6.15
5.33 5.29 5.27
2.68 2.67 2.65 2.63 2.61
1.23 1.21 1.18 1.16
O
O
S19
^{1}H, CDCl$_3$, 400 MHz
0.96 2.03 1.09 3.11 1.69 0.94 1.10 1.98 1.20 3.36 2.65
9.0 8.5 8.0 7.5 7.0 6.5 6.0 5.5 5.0 4.5 4.0 3.5 3.0 2.5 2.0 1.5 1.0 0.5 0.0
f1 (ppm)

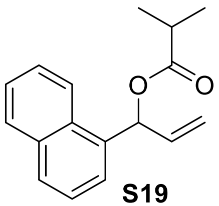

S19

^{13}C, $CDCl_3$, 126 MHz

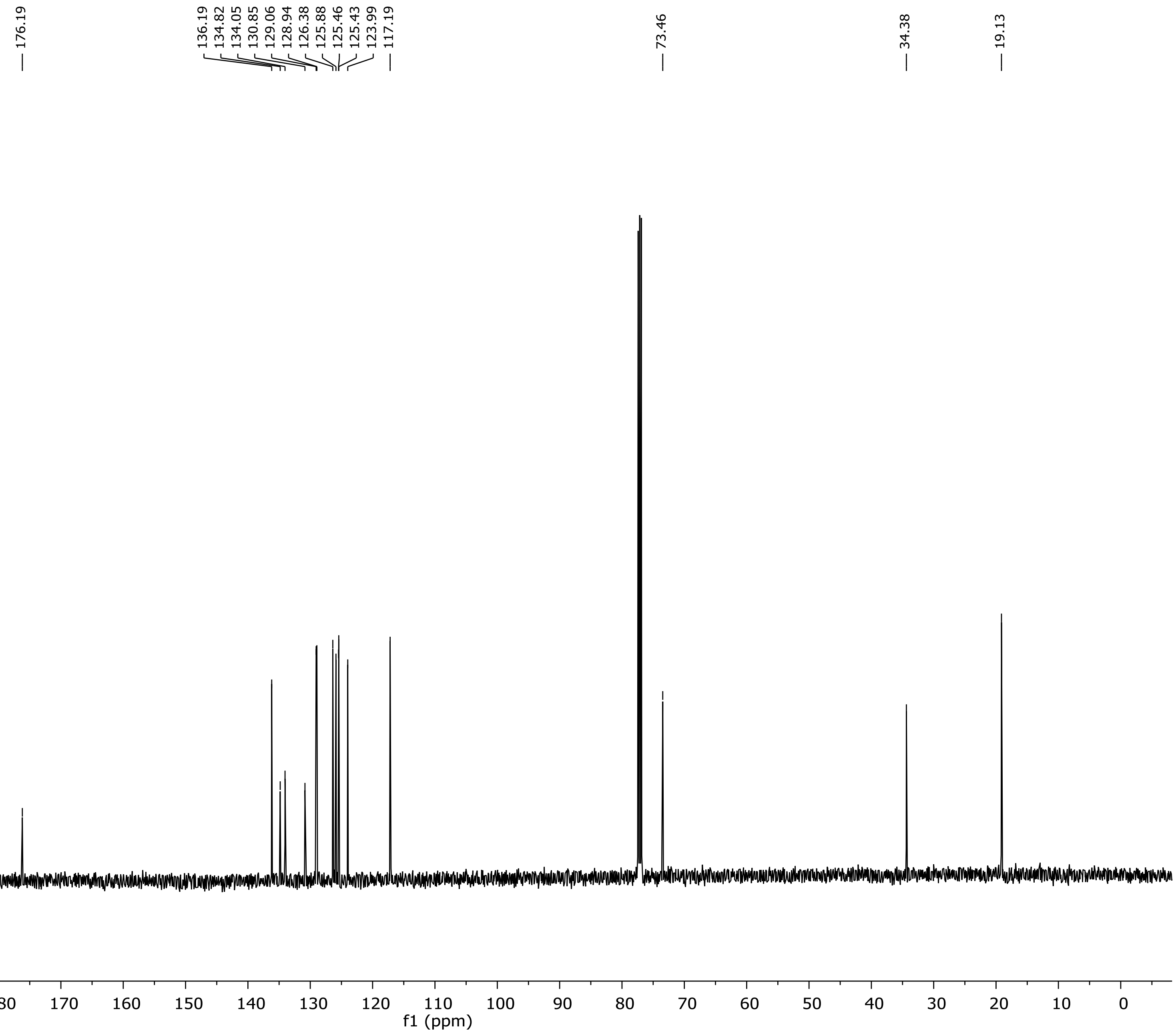

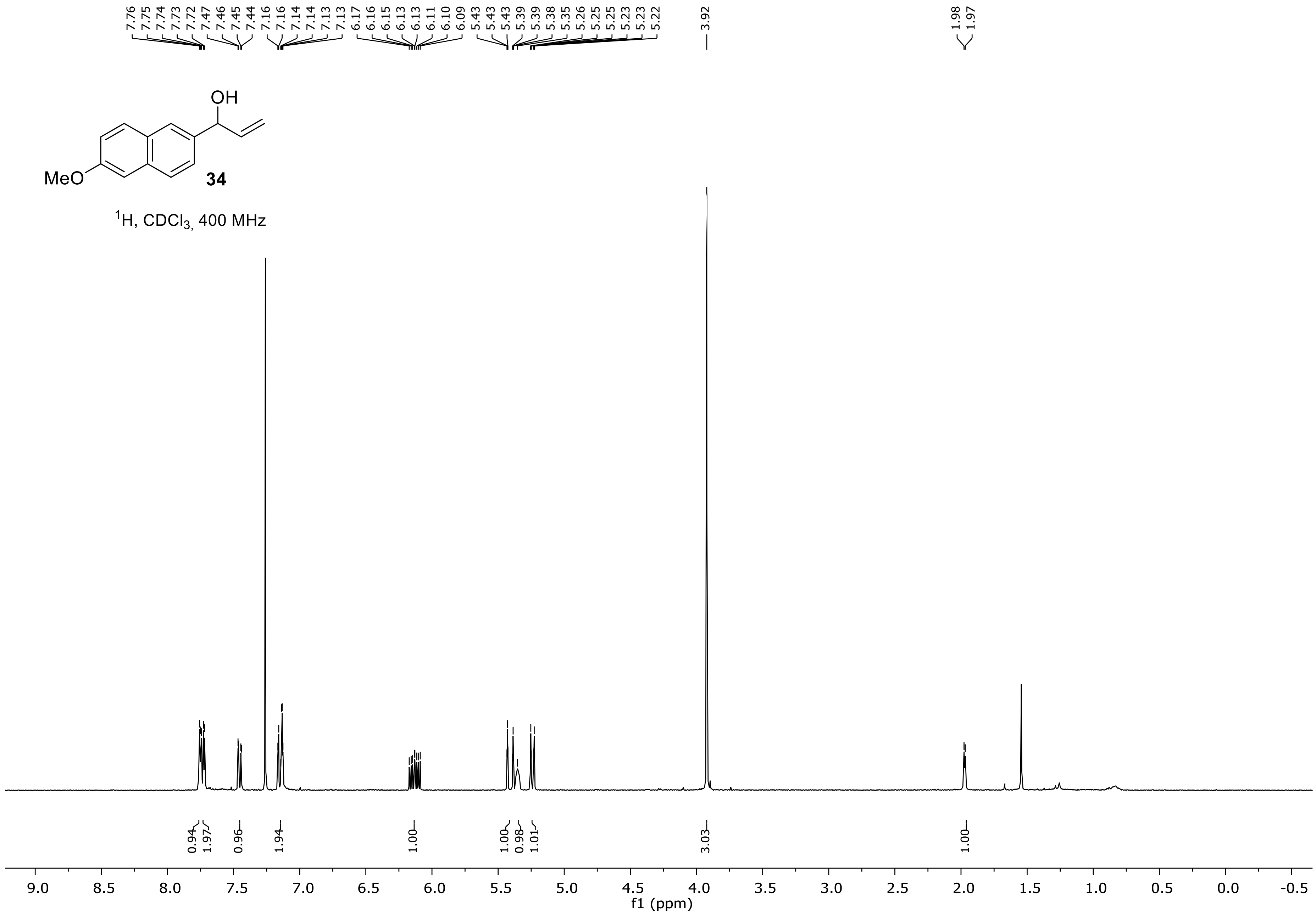
7.76 7.75 7.74 7.73 7.72 7.47 7.46 7.45 7.44 7.16 7.16 7.14 7.14 7.13 7.13 6.17 6.16 6.15 6.13 6.13 6.11 6.10 6.09 5.43 5.43 5.43 5.39 5.39 5.38 5.35 5.26 5.25 5.25 5.23 5.23 5.22 3.92 1.98 1.97
OH
MeO
34
^{1}H, $CDCl_3$, 400 MHz
0.94 1.97 0.96 1.94 1.00 1.00 0.98 1.01 3.03 1.00
9.0 8.5 8.0 7.5 7.0 6.5 6.0 5.5 5.0 4.5 4.0 3.5 3.0 2.5 2.0 1.5 1.0 0.5 0.0 -0.5
f1 (ppm)

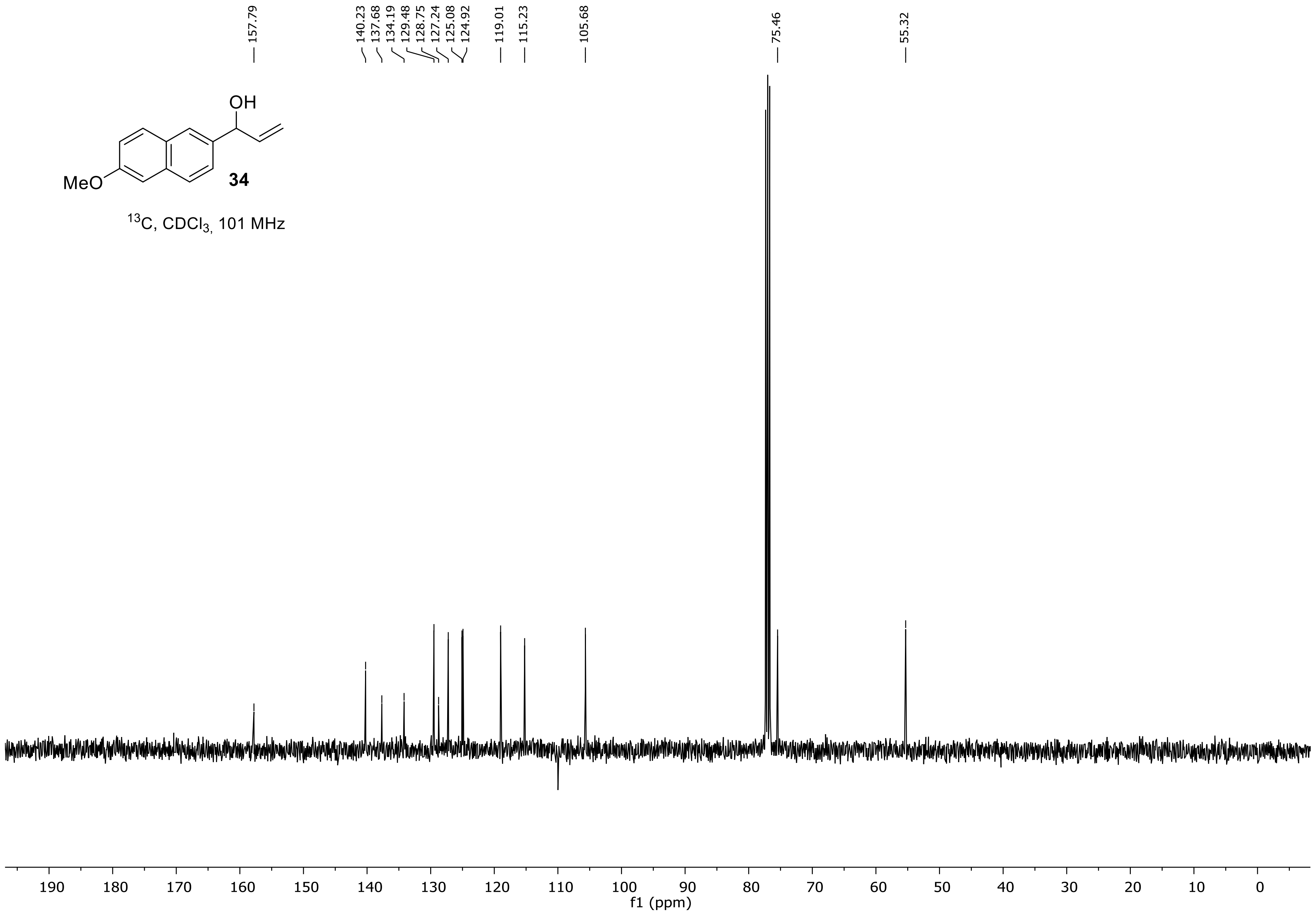
157.79
140.23
137.68
134.19
129.48
128.75
127.24
125.08
124.92
119.01
115.23
105.68
75.46
55.32
OH
MeO
34
^{13}C, $CDCl_3$, 101 MHz
190 180 170 160 150 140 130 120 110 100 90 80 70 60 50 40 30 20 10 0
f1 (ppm)

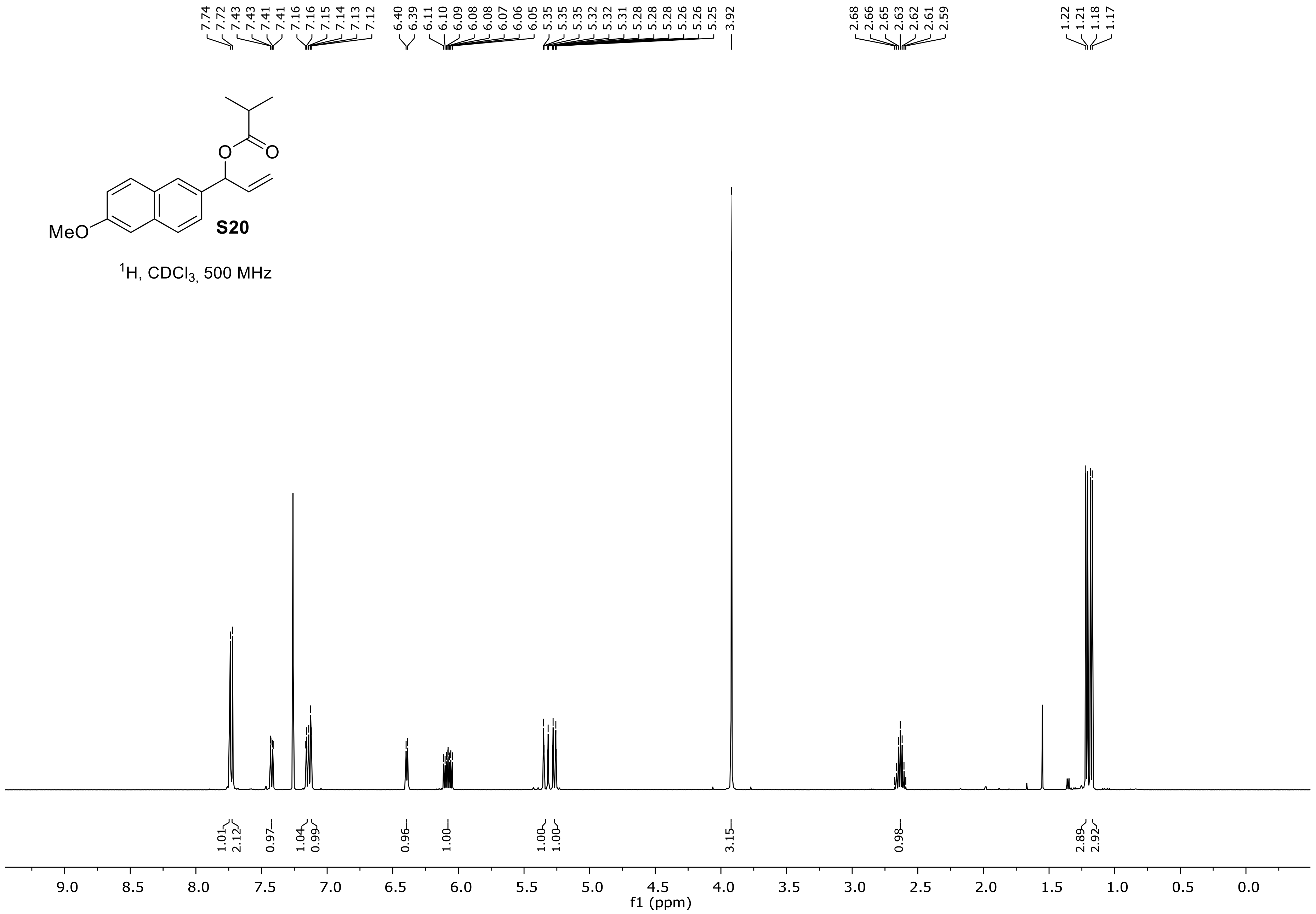
7.74
7.72
7.43
7.43
7.41
7.41
7.16
7.16
7.15
7.14
7.13
7.12
6.40
6.39
6.11
6.10
6.09
6.08
6.08
6.07
6.06
6.05
5.35
5.35
5.35
5.32
5.32
5.31
5.28
5.28
5.28
5.26
5.26
5.25
3.92
2.68
2.66
2.65
2.63
2.62
2.61
2.59
1.22
1.21
1.18
1.17
MeO
O
O
S20
^{1}H, CDCl3, 500 MHz
1.01
2.12
0.97
1.04
0.99
0.96
1.00
1.00
1.00
3.15
0.98
2.89
2.92
9.0
8.5
8.0
7.5
7.0
6.5
6.0
5.5
5.0
4.5
4.0
3.5
3.0
2.5
2.0
1.5
1.0
0.5
0.0
f1 (ppm)

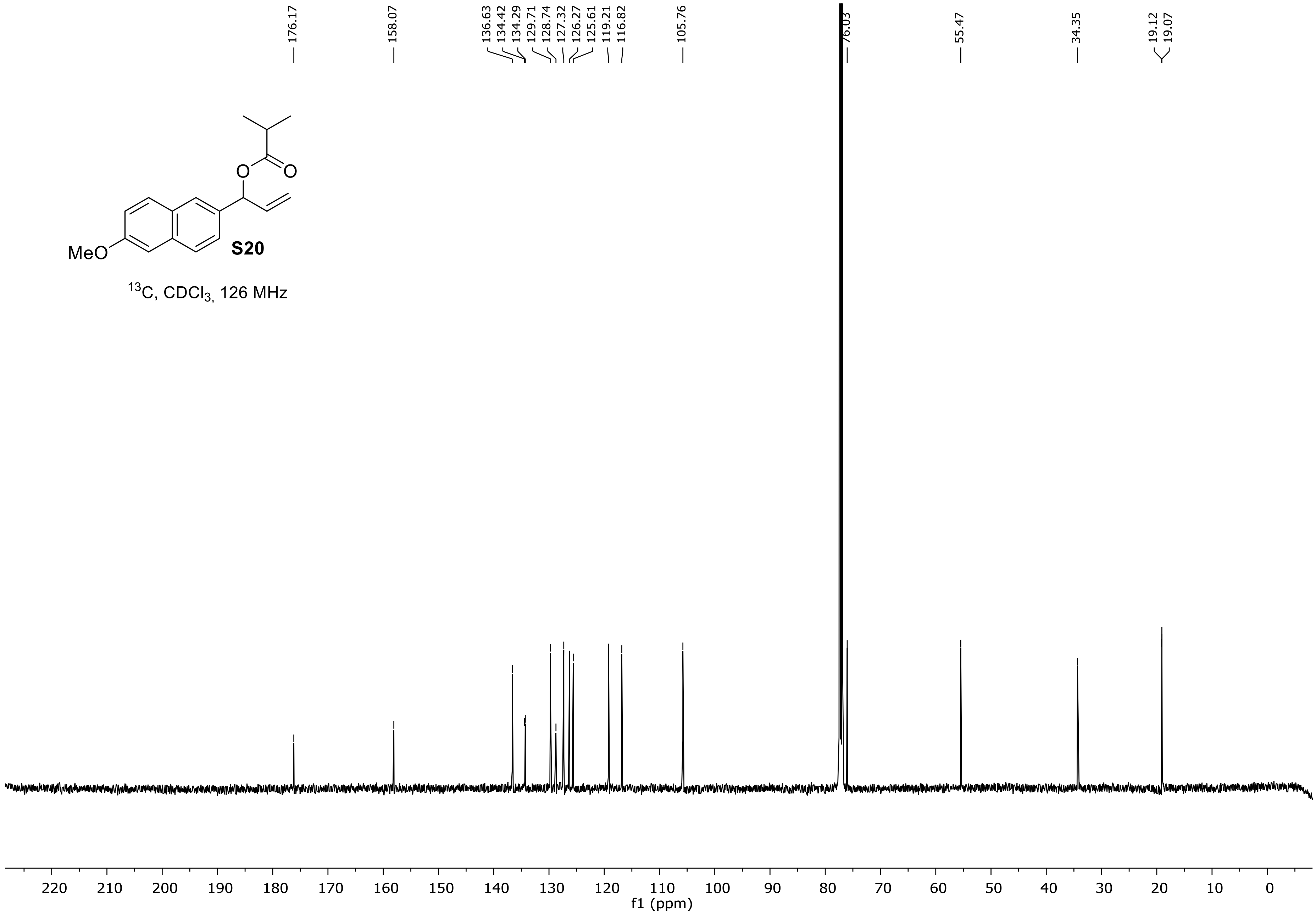
176.17
158.07
136.63
134.42
134.29
129.71
128.74
127.32
126.27
125.61
119.21
116.82
105.76
76.03
55.47
34.35
19.12
19.07
MeO
O
O
S20
13C, CDCl3, 126 MHz
220 210 200 190 180 170 160 150 140 130 120 110 100 90 80 70 60 50 40 30 20 10 0
f1 (ppm)

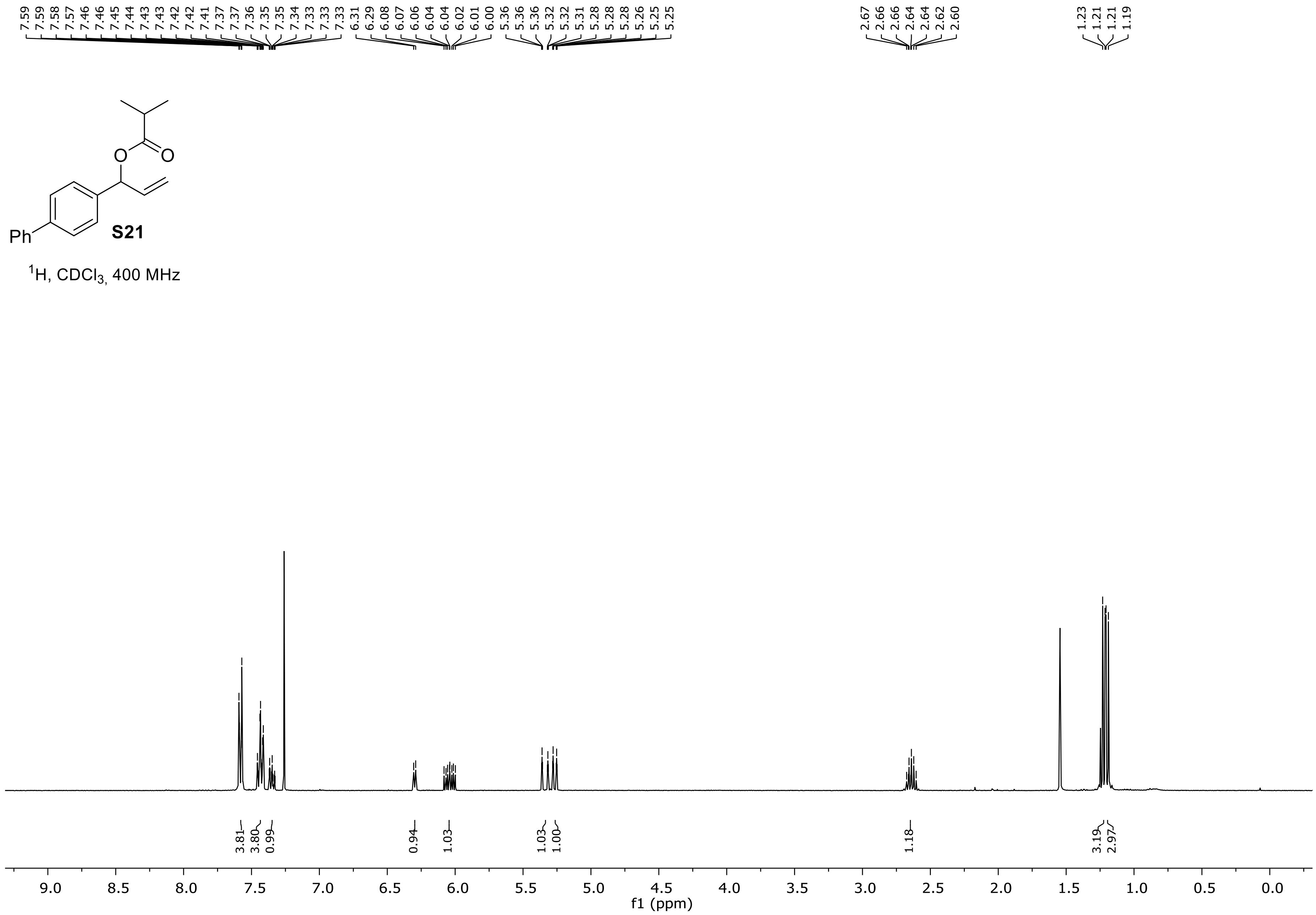
Ph
S21
^{1}H, $CDCl_3$, 400 MHz
f1 (ppm)

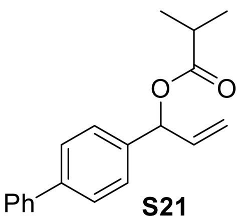

S21

^{13}C, $CDCl_3$, 126 MHz

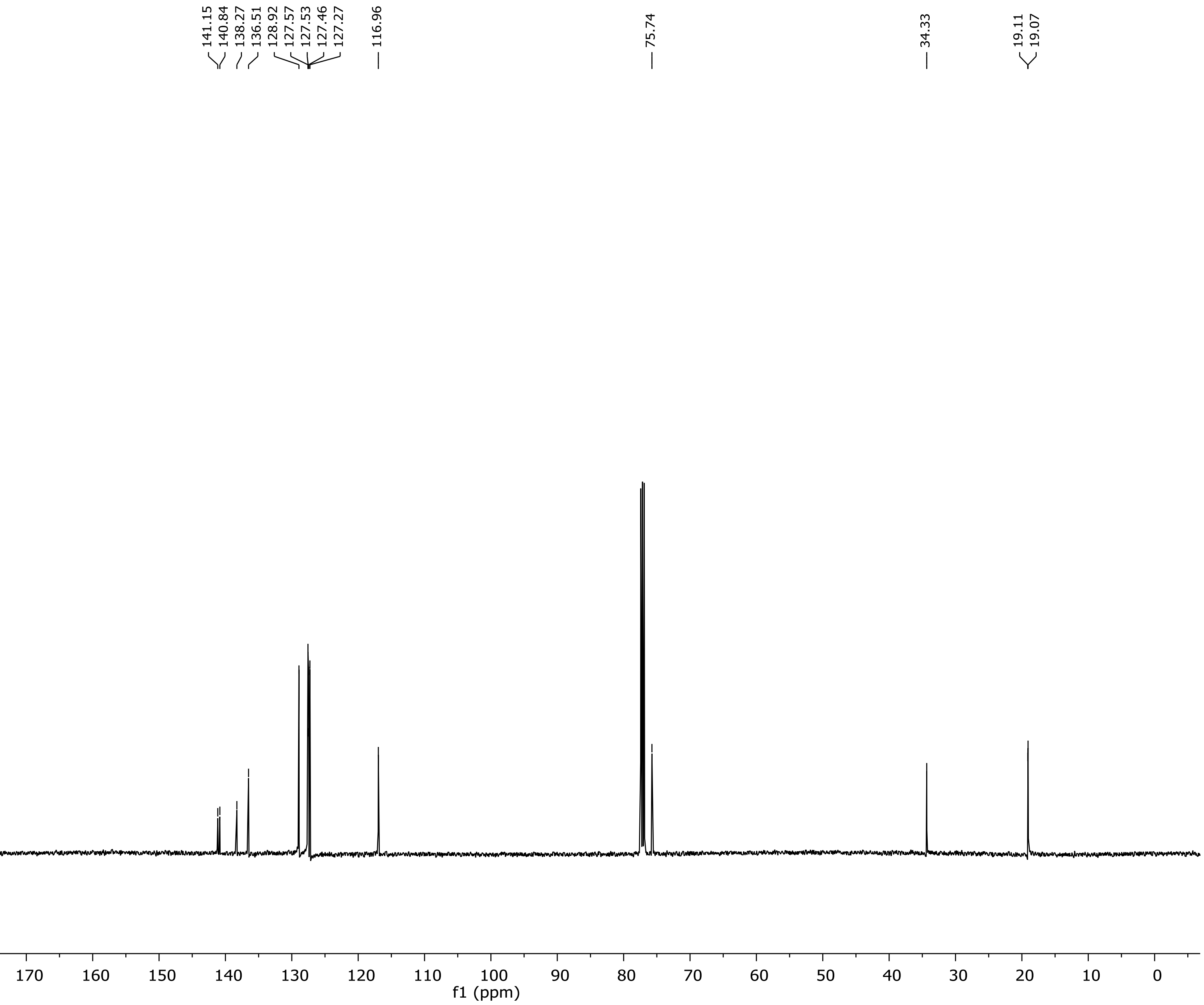

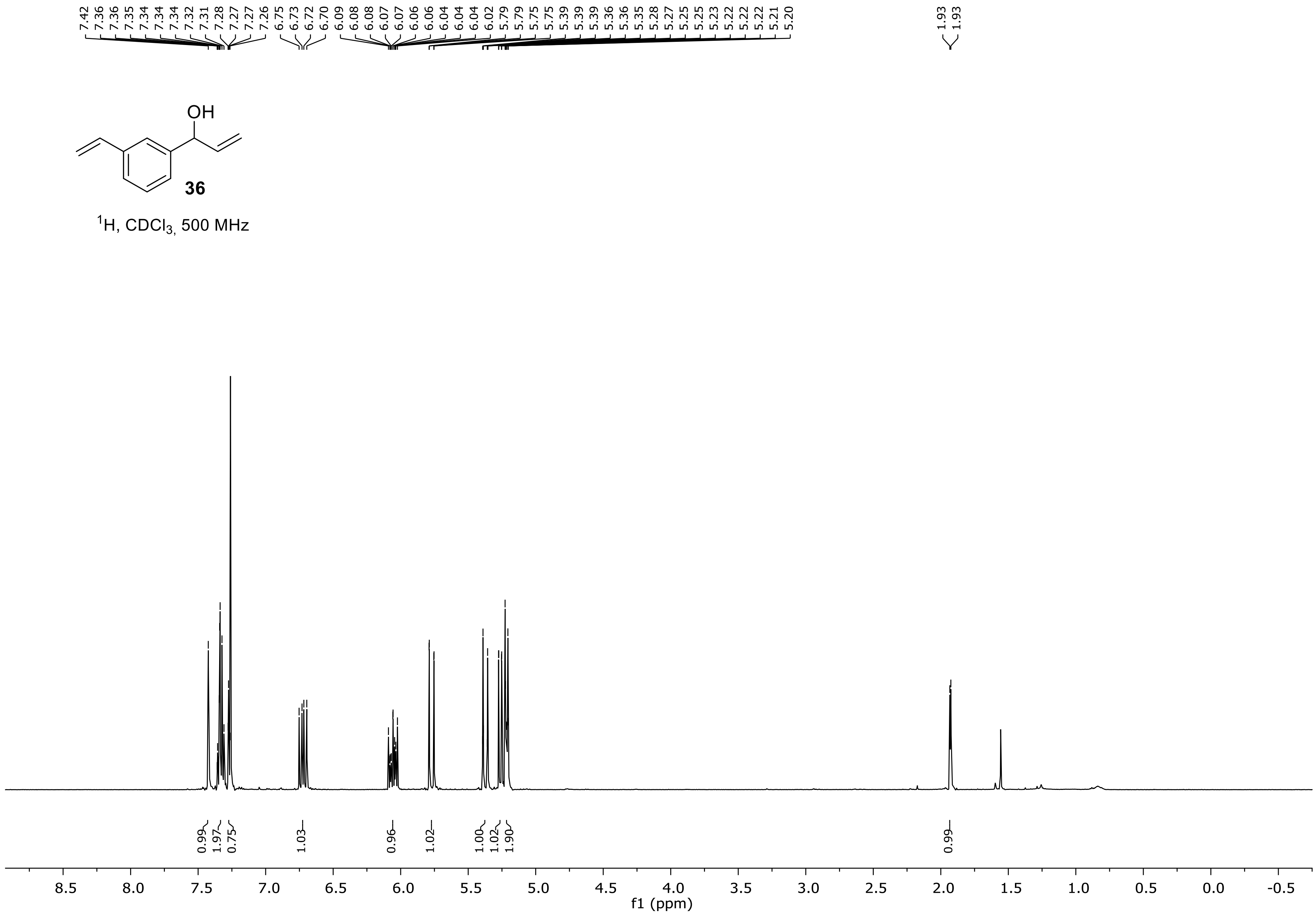
7.42 7.36 7.36 7.35 7.34 7.34 7.34 7.32 7.31 7.28 7.27 7.27 7.26 6.75 6.73 6.72 6.70 6.09 6.08 6.08 6.07 6.07 6.06 6.06 6.04 6.04 6.04 6.02 5.79 5.79 5.75 5.75 5.39 5.39 5.39 5.36 5.36 5.35 5.28 5.27 5.25 5.25 5.23 5.22 5.22 5.22 5.21 5.20
1.93 1.93
OH
36
^1H, $CDCl_3$, 500 MHz
0.99 1.97 0.75 1.03 0.96 1.02 1.00 1.02 1.90 0.99
8.5 8.0 7.5 7.0 6.5 6.0 5.5 5.0 4.5 4.0 3.5 3.0 2.5 2.0 1.5 1.0 0.5 0.0 -0.5
f1 (ppm)

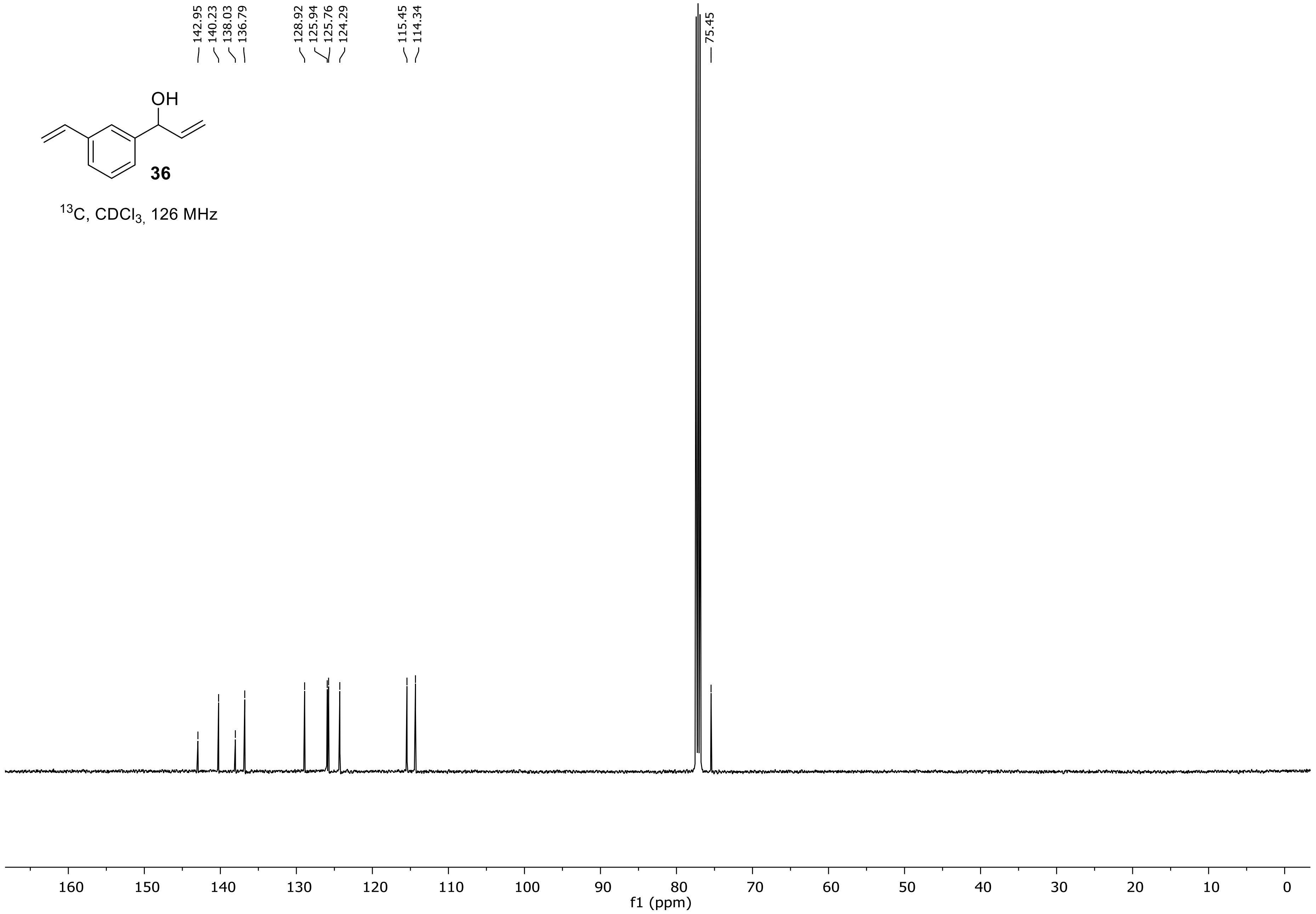
142.95
140.23
138.03
136.79
128.92
125.94
125.76
124.29
115.45
114.34
75.45
OH
36
^{13}C, $CDCl_3$, 126 MHz
160 150 140 130 120 110 100 90 80 70 60 50 40 30 20 10 0
f1 (ppm)

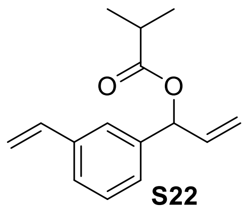

S22

^{1}H, $CDCl_3$, 400 MHz

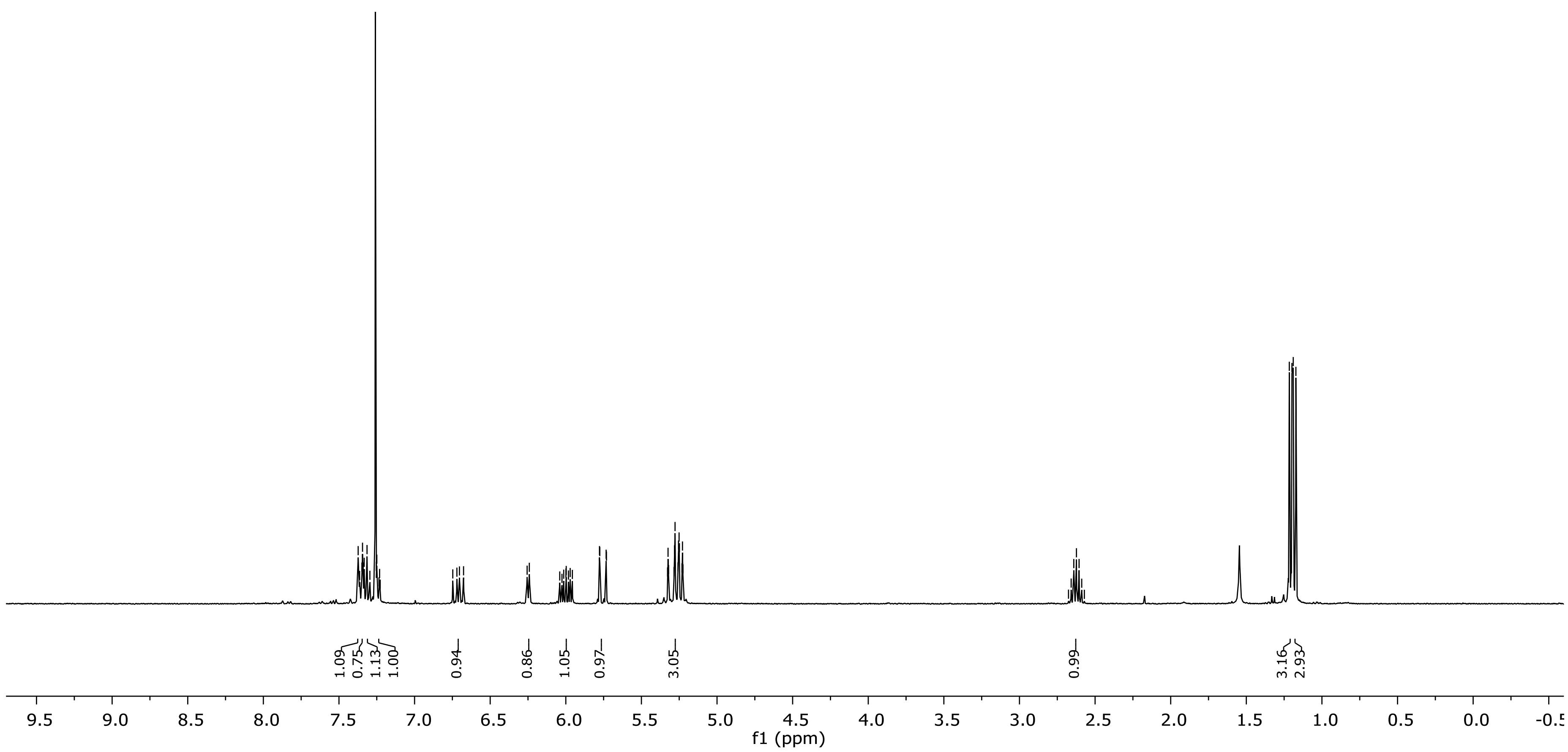

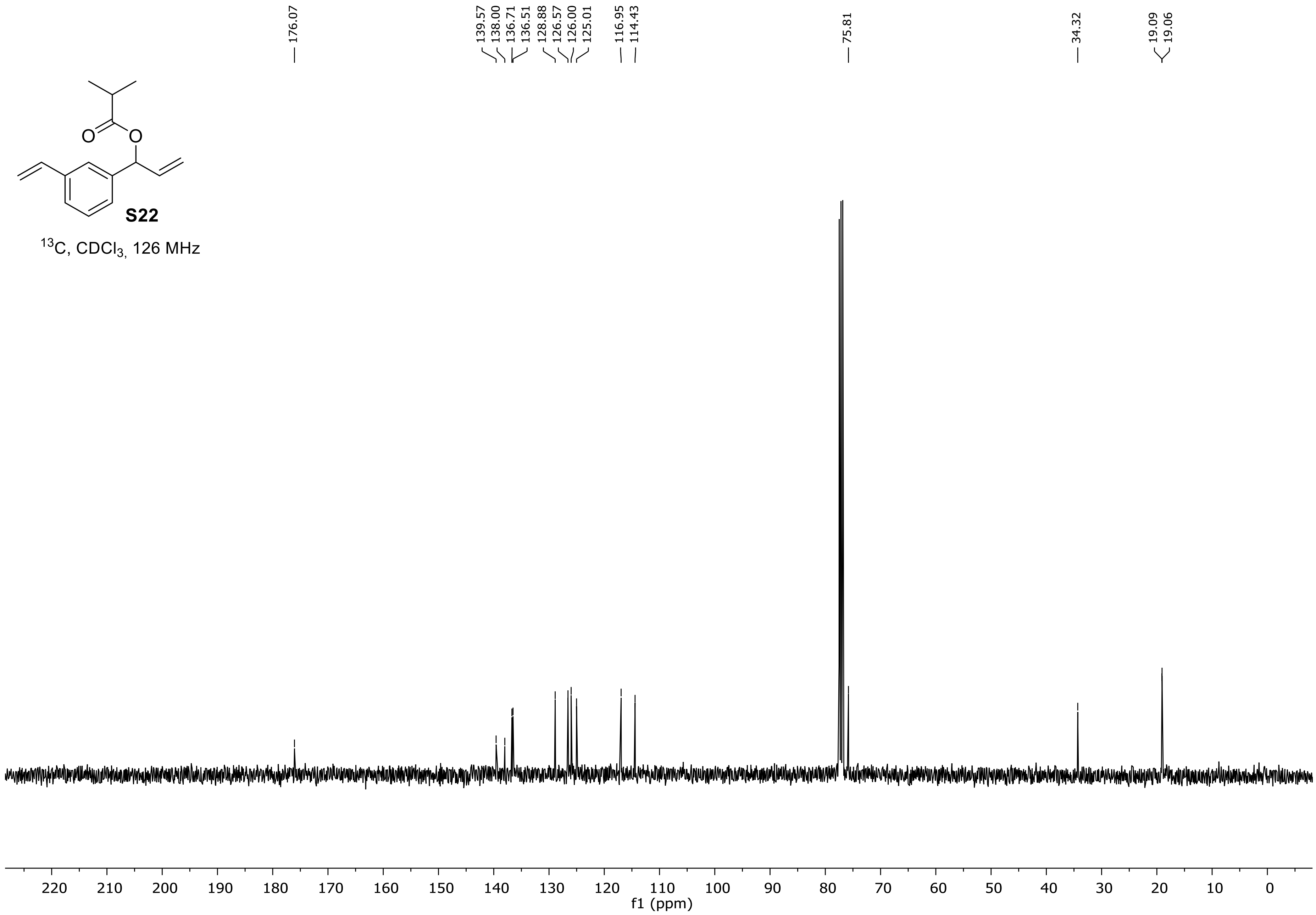
S22
^{13}C, $CDCl_3$, 126 MHz
176.07
139.57
138.00
136.71
136.51
128.88
126.57
126.00
125.01
116.95
114.43
75.81
34.32
19.09
19.06
220 210 200 190 180 170 160 150 140 130 120 110 100 90 80 70 60 50 40 30 20 10 0
f1 (ppm)

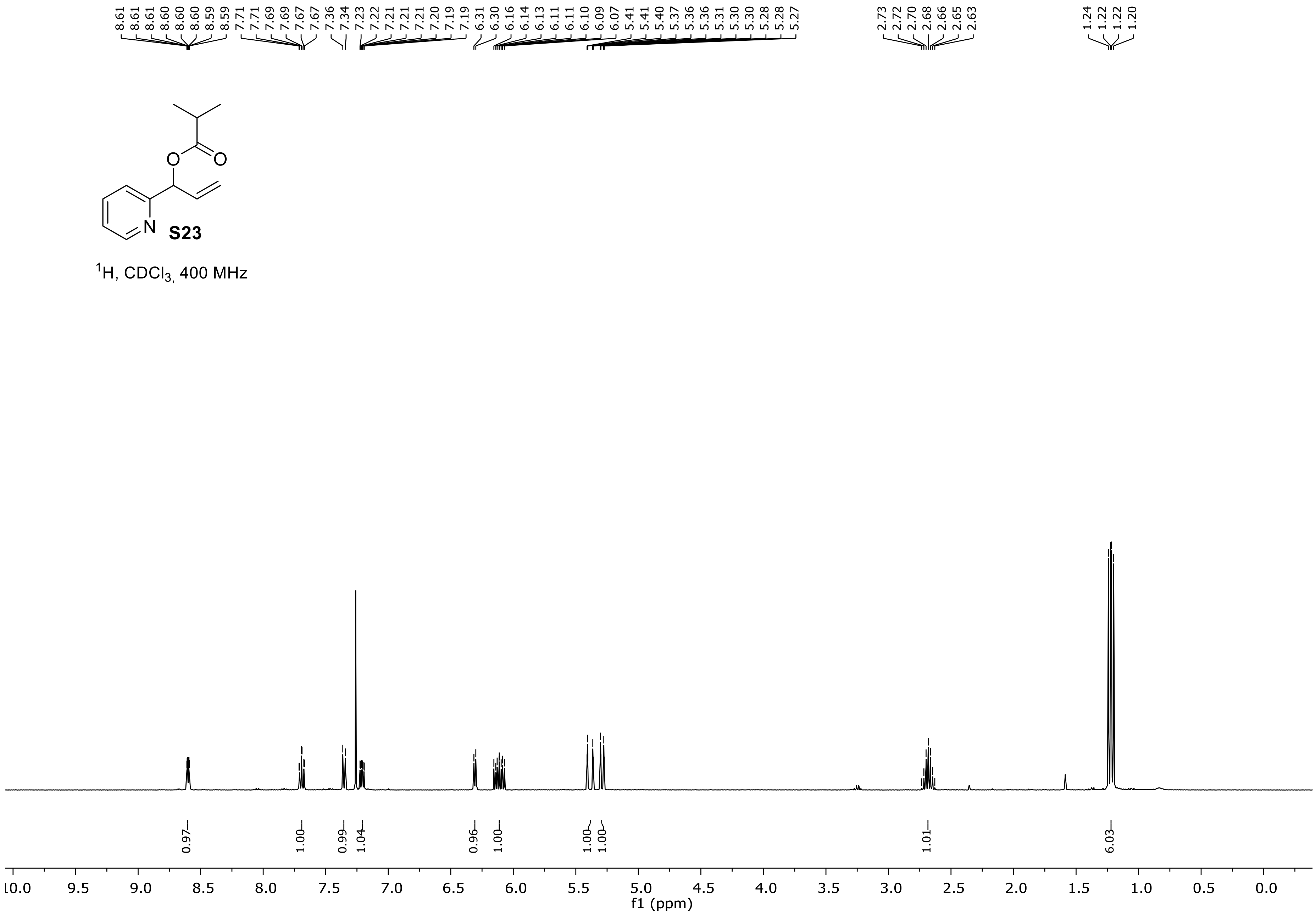
8.61 8.61 8.61 8.60 8.60 8.60 8.59 8.59 7.71 7.71 7.69 7.69 7.67 7.67 7.36 7.34 7.23 7.22 7.21 7.21 7.21 7.20 7.19 7.19 6.31 6.30 6.16 6.14 6.13 6.11 6.11 6.10 6.09 6.07 5.41 5.41 5.40 5.37 5.36 5.36 5.31 5.30 5.30 5.28 5.28 5.27
2.73 2.72 2.70 2.68 2.66 2.65 2.63
1.24 1.22 1.22 1.20
O
O
N
S23
^{1}H, $CDCl_3$, 400 MHz
0.97 1.00 0.99 1.04 0.96 1.00 1.00 1.00 1.01 6.03
10.0 9.5 9.0 8.5 8.0 7.5 7.0 6.5 6.0 5.5 5.0 4.5 4.0 3.5 3.0 2.5 2.0 1.5 1.0 0.5 0.0
f1 (ppm)

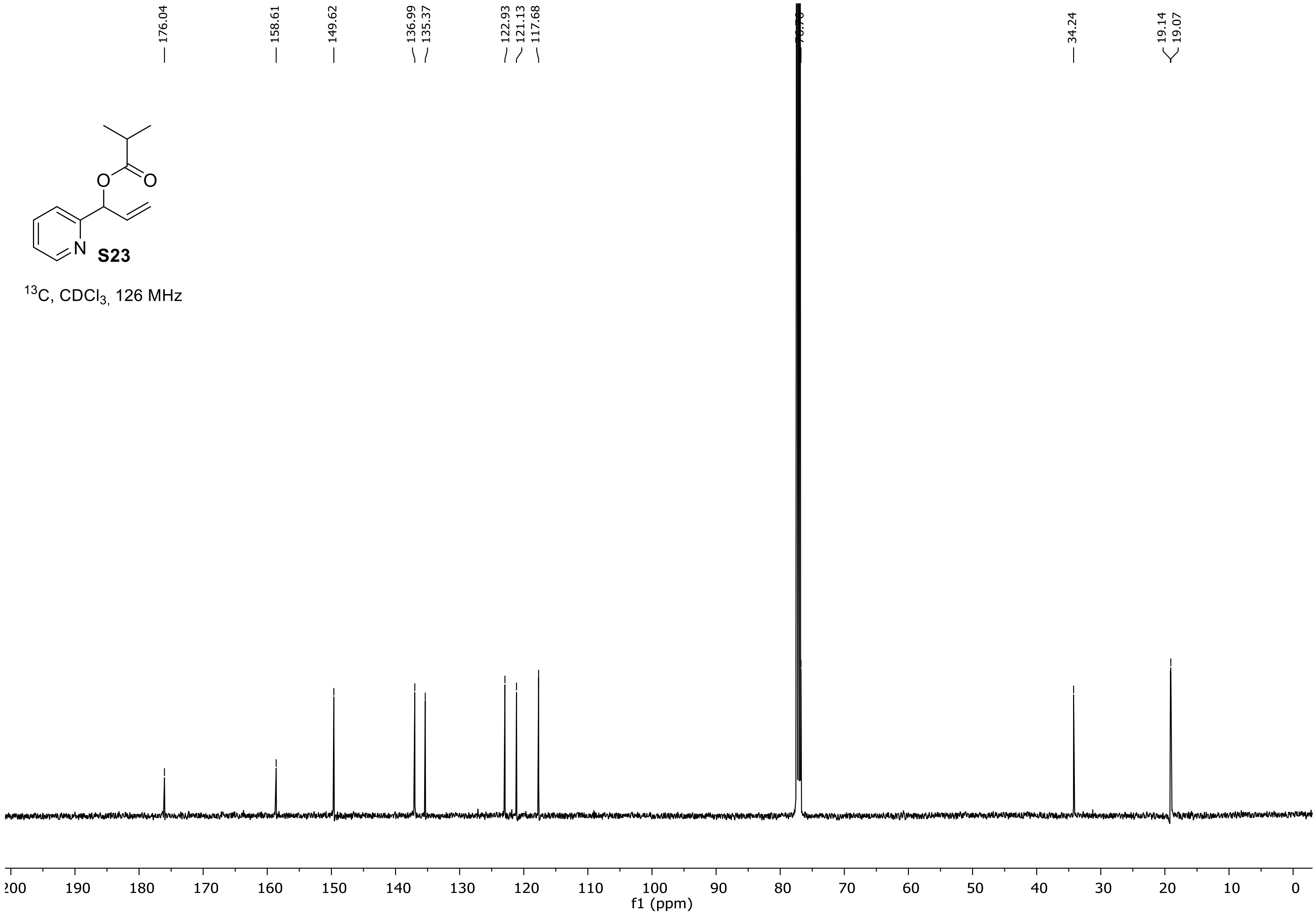
176.04
158.61
149.62
136.99
135.37
122.93
121.13
117.68
34.24
19.14
19.07
S23
^{13}C, $CDCl_3$, 126 MHz
200 190 180 170 160 150 140 130 120 110 100 90 80 70 60 50 40 30 20 10 0
f1 (ppm)

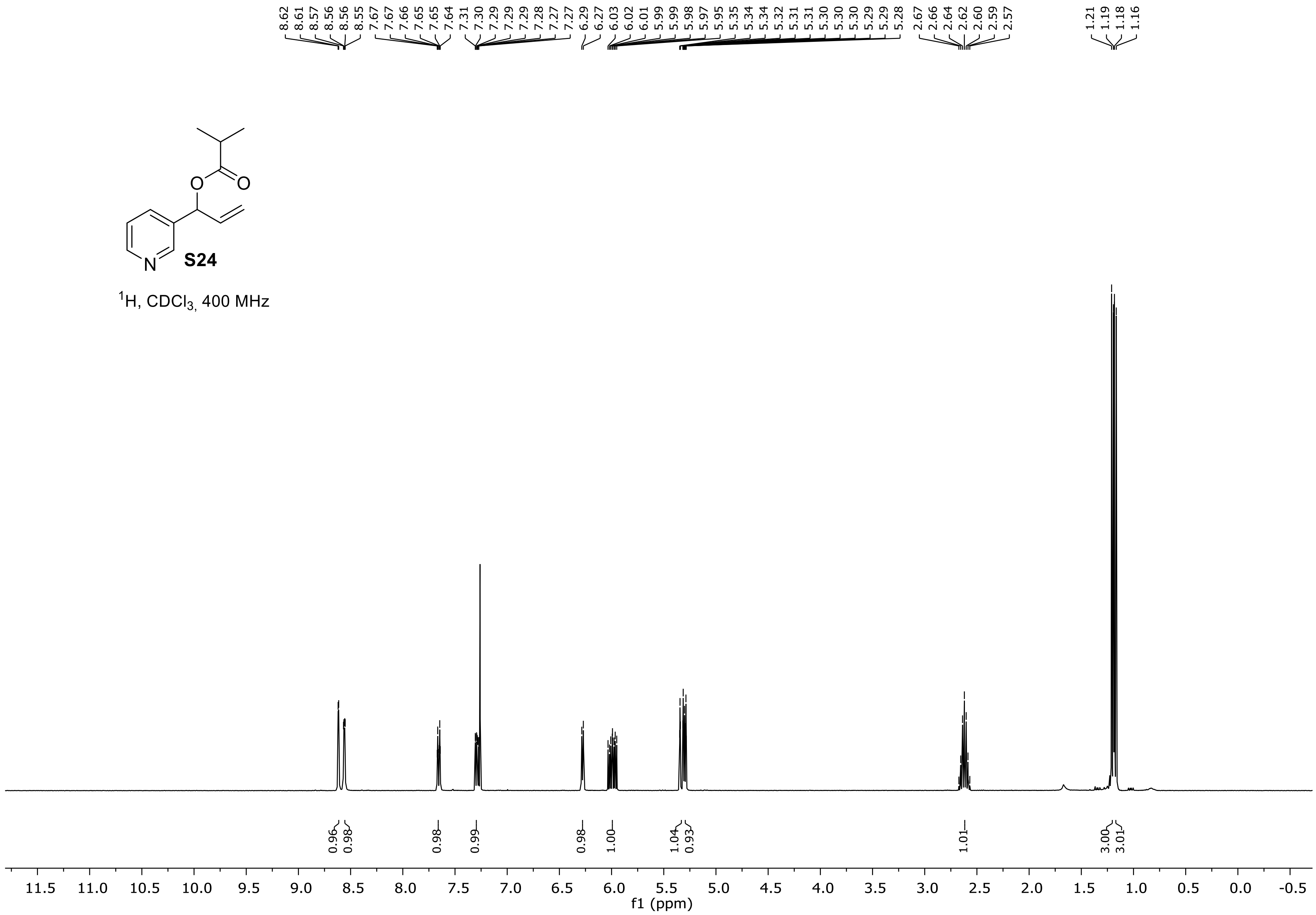

S24

^{1}H, $CDCl_3$, 400 MHz

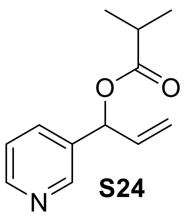

S24

^{13}C, $CDCl_3$, 101 MHz

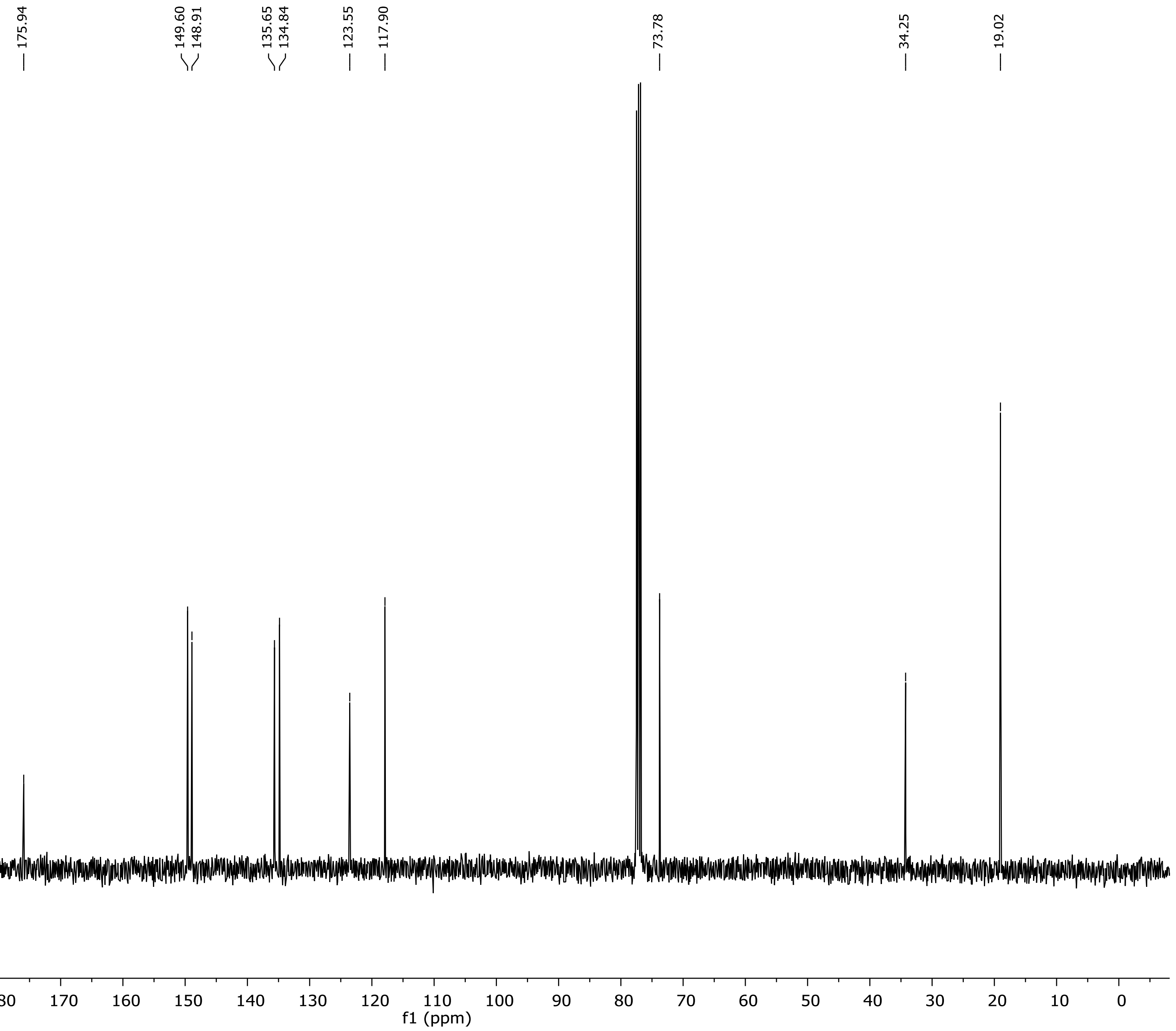

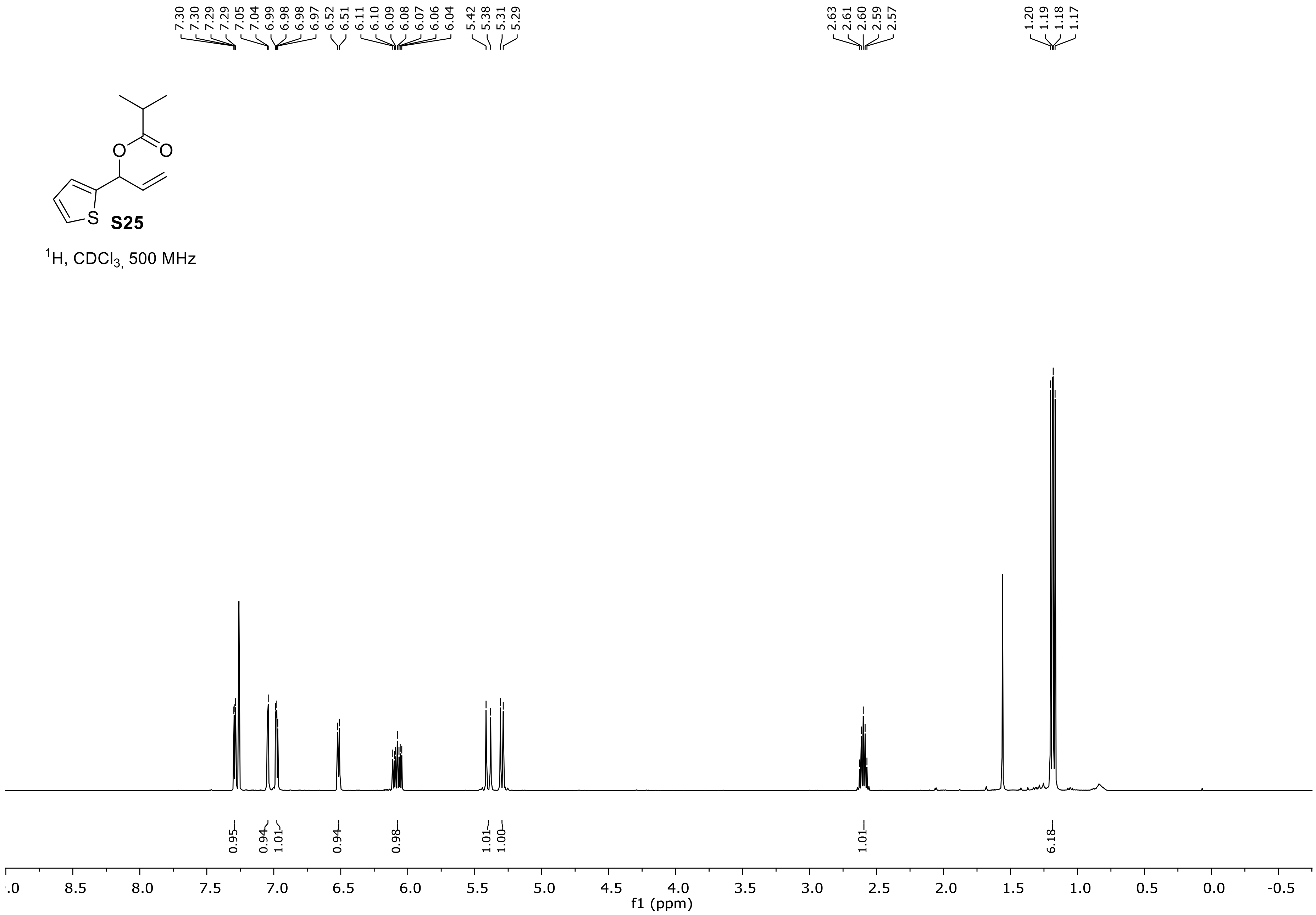
7.30
7.30
7.29
7.29
7.05
7.04
6.99
6.98
6.98
6.97
6.52
6.51
6.11
6.10
6.09
6.08
6.07
6.06
6.04
5.42
5.38
5.31
5.29
2.63
2.61
2.60
2.59
2.57
1.20
1.19
1.18
1.17
O
O
S
S25
^{1}H, $CDCl_3$, 500 MHz
0.95
0.94
1.01
0.94
0.98
1.01
1.00
1.01
6.18
8.5
8.0
7.5
7.0
6.5
6.0
5.5
5.0
4.5
4.0
3.5
3.0
2.5
2.0
1.5
1.0
0.5
0.0
-0.5
f1 (ppm)

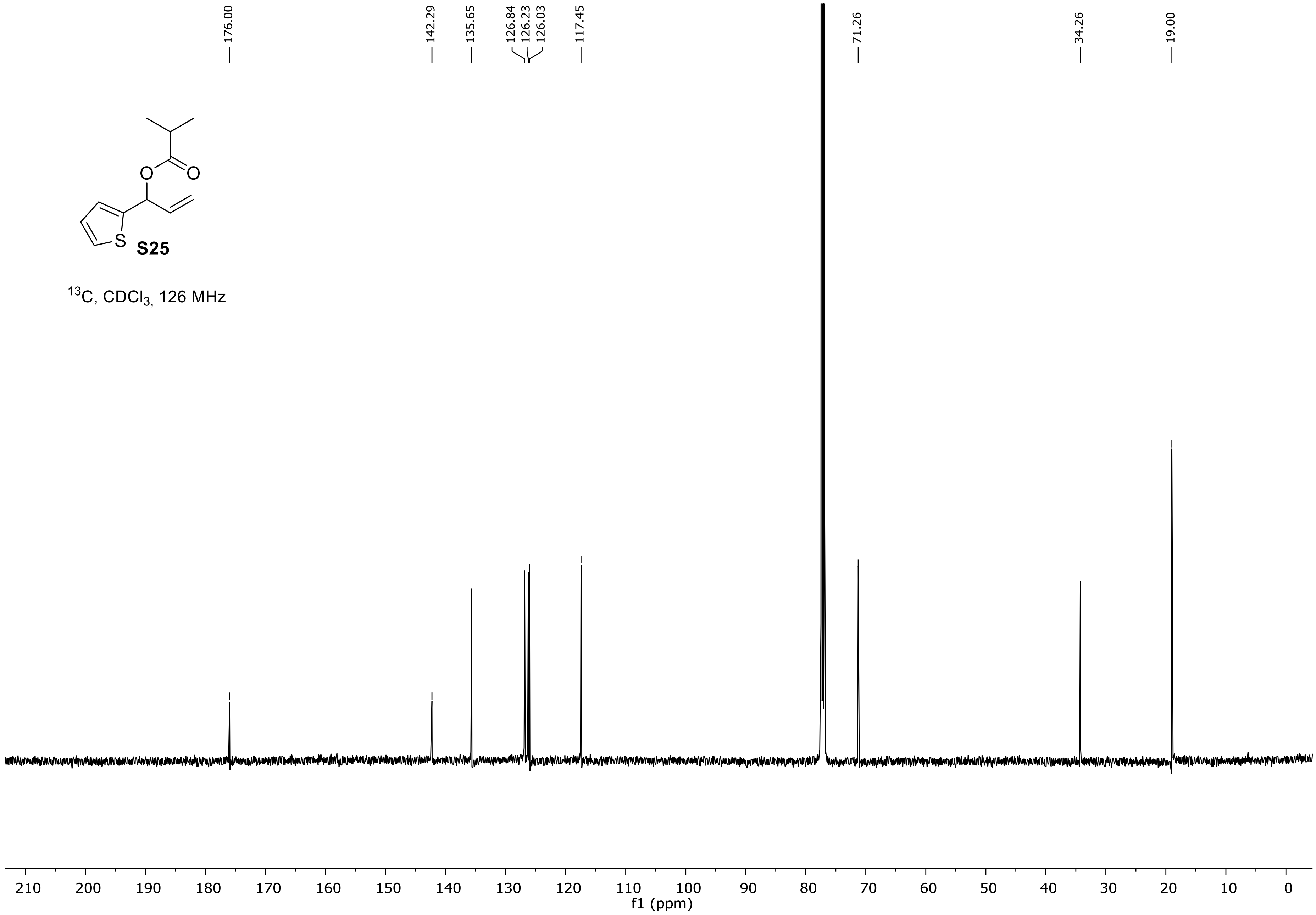
176.00
142.29
135.65
126.84
126.23
126.03
117.45
71.26
34.26
19.00
S25
^{13}C, $CDCl_3$, 126 MHz
210 200 190 180 170 160 150 140 130 120 110 100 90 80 70 60 50 40 30 20 10 0
f1 (ppm)

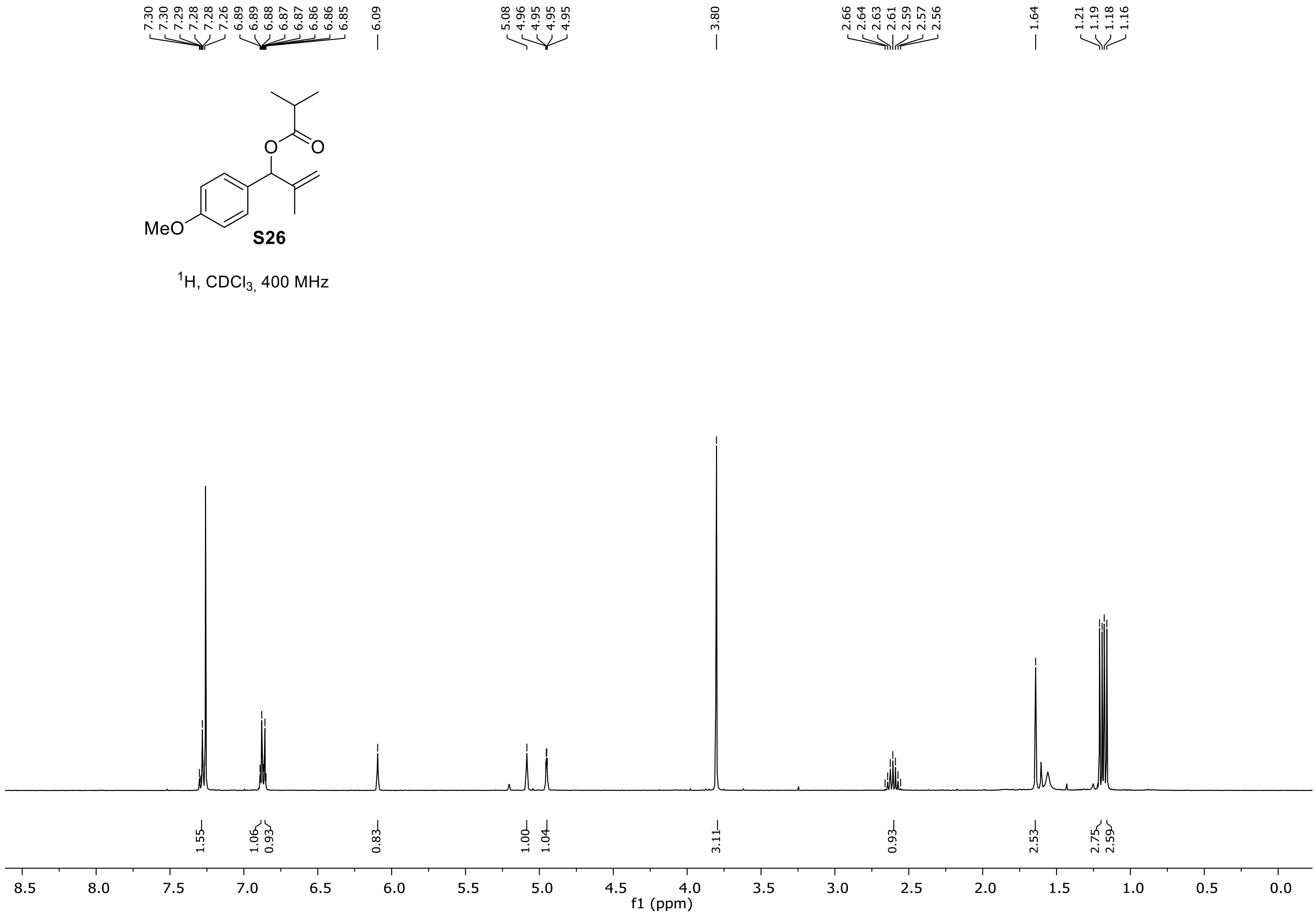
7.30
7.30
7.29
7.28
7.28
7.26
6.89
6.89
6.88
6.87
6.87
6.86
6.86
6.85
6.09
5.08
4.96
4.95
4.95
4.95
3.80
2.66
2.64
2.63
2.61
2.59
2.57
2.56
1.64
1.21
1.19
1.18
1.16
MeO
O
O
S26
^{1}H, $CDCl_3$, 400 MHz
1.55
1.06
0.93
0.83
1.00
1.04
3.11
0.93
2.53
2.75
2.59
8.5
8.0
7.5
7.0
6.5
6.0
5.5
5.0
4.5
4.0
3.5
3.0
2.5
2.0
1.5
1.0
0.5
0.0
f1 (ppm)

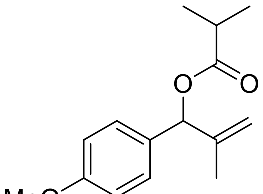

S26

^{13}C, $CDCl_3$, 126 MHz

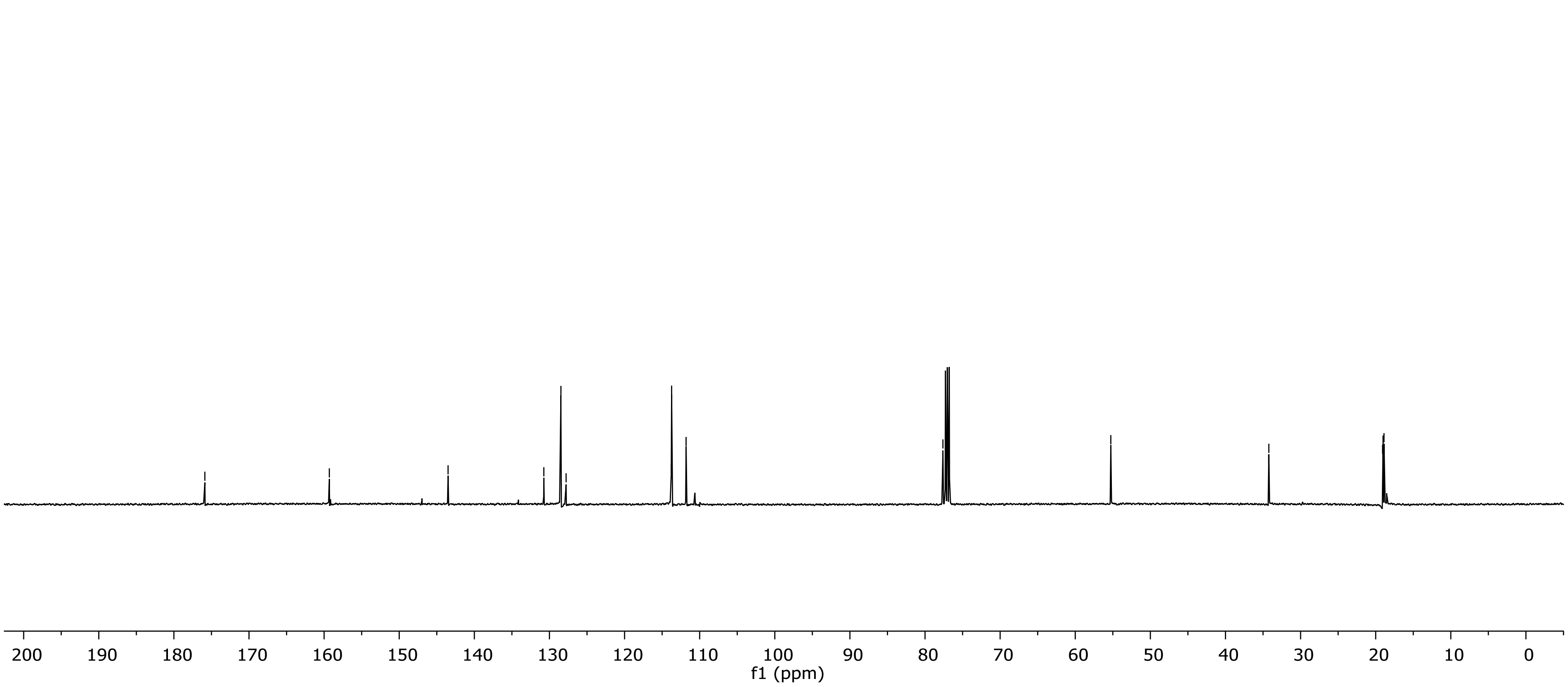

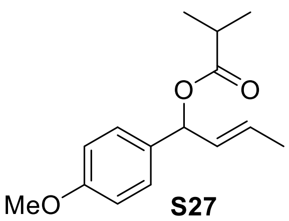

S27

^{1}H, $CDCl_3$, 400 MHz

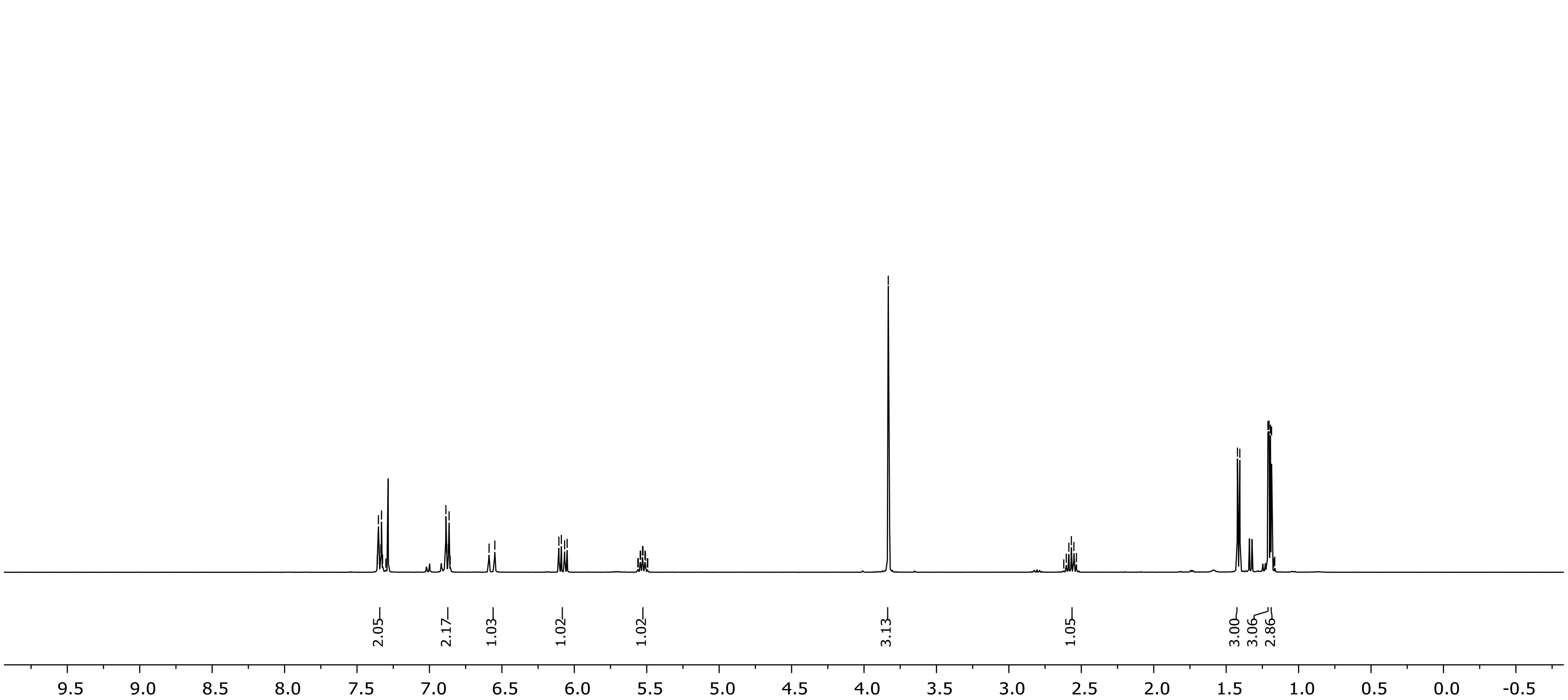

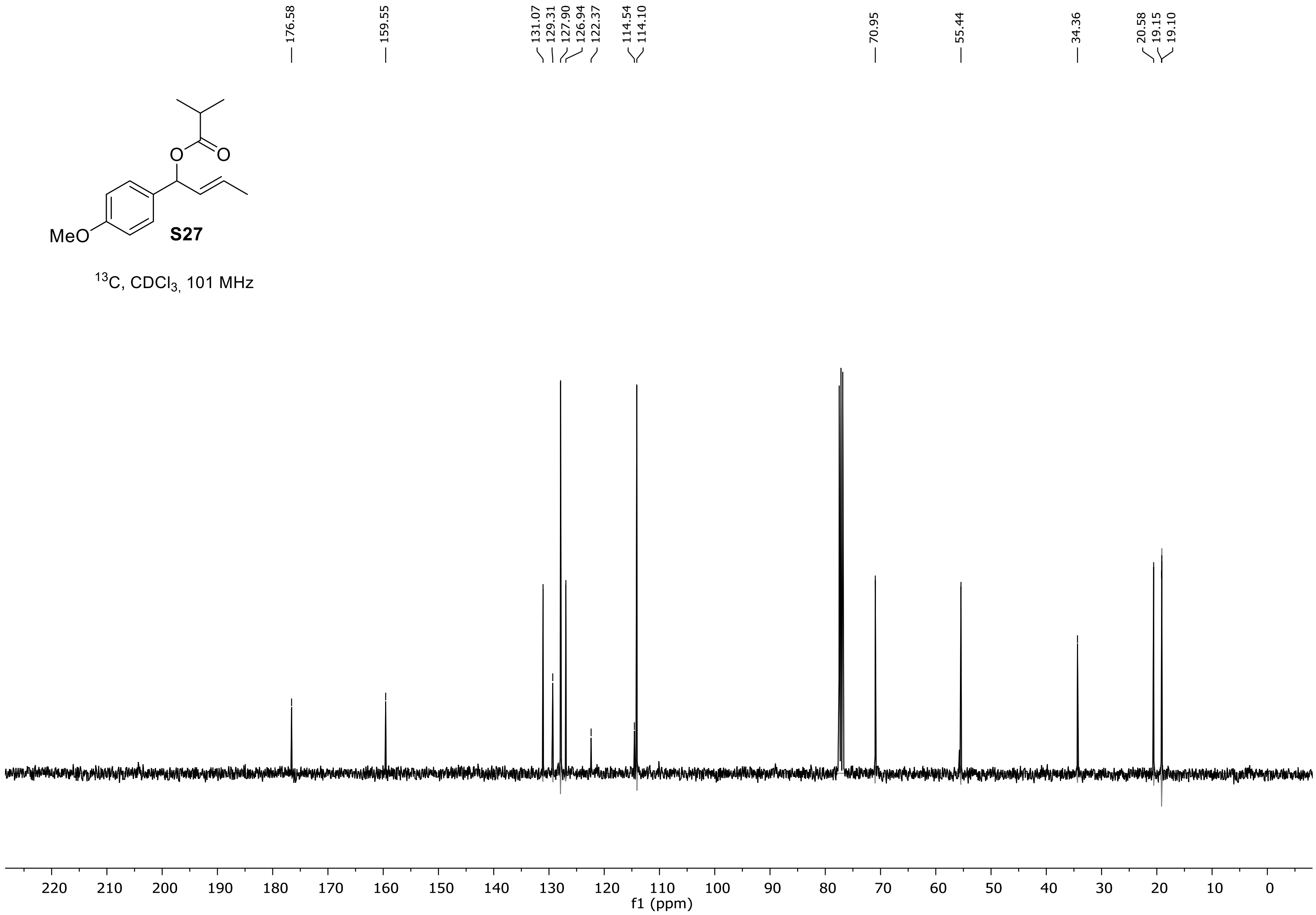
176.58
159.55
131.07
129.31
127.90
126.94
122.37
114.54
114.10
70.95
55.44
34.36
20.58
19.15
19.10
MeO
O
O
S27
^{13}C, $CDCl_3$, 101 MHz
220 210 200 190 180 170 160 150 140 130 120 110 100 90 80 70 60 50 40 30 20 10 0
f1 (ppm)

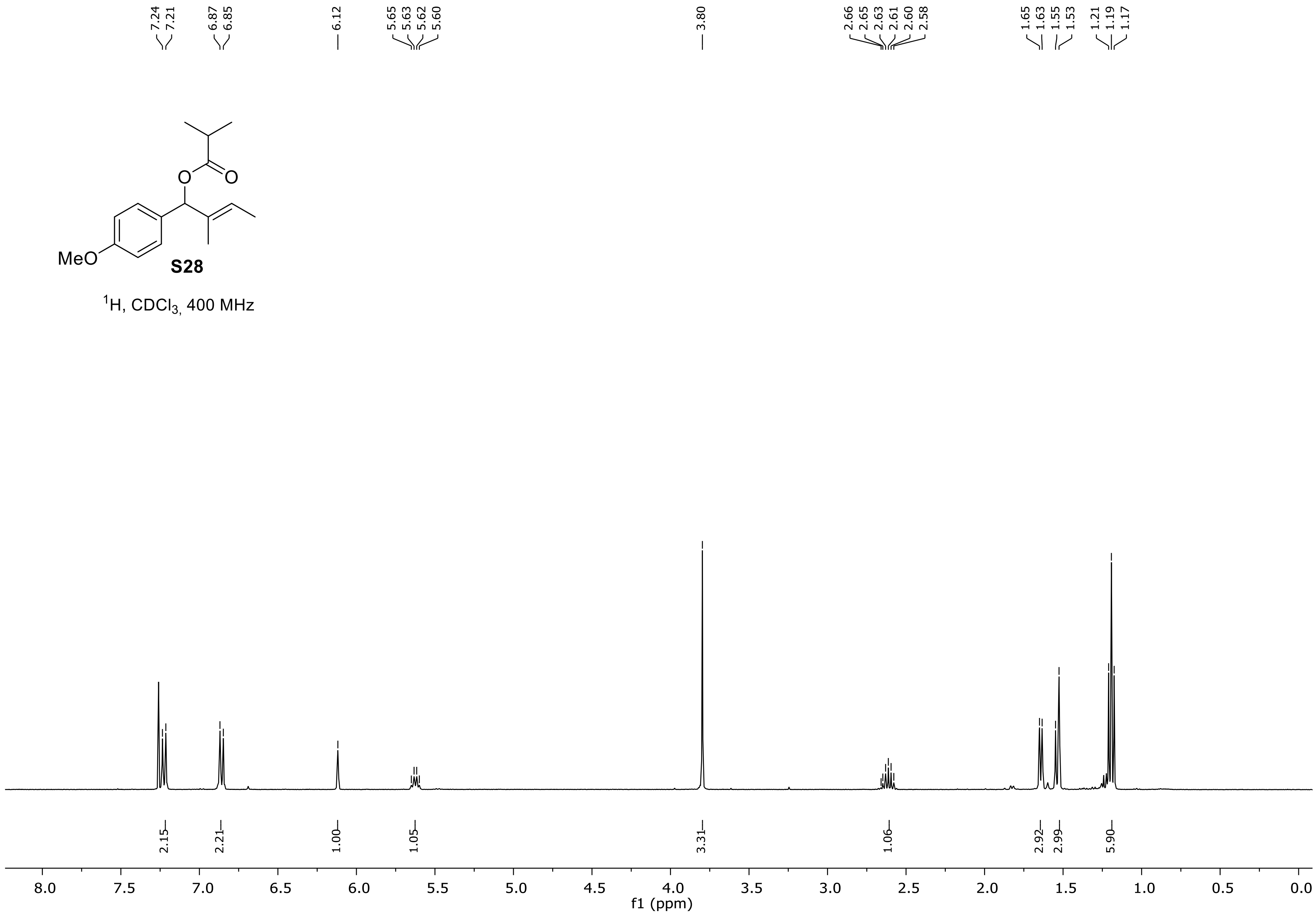
7.24
7.21
6.87
6.85
6.12
5.65
5.63
5.62
5.60
3.80
2.66
2.65
2.63
2.61
2.60
2.58
1.65
1.63
1.55
1.53
1.21
1.19
1.17
MeO
O
O
S28
^{1}H, CDCl$_3$, 400 MHz
2.15
2.21
1.00
1.05
3.31
1.06
2.92
2.99
5.90
8.0 7.5 7.0 6.5 6.0 5.5 5.0 4.5 4.0 3.5 3.0 2.5 2.0 1.5 1.0 0.5 0.0
f1 (ppm)

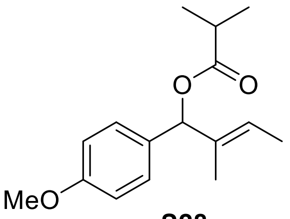

S28

^{13}C, $CDCl_3$, 101 MHz

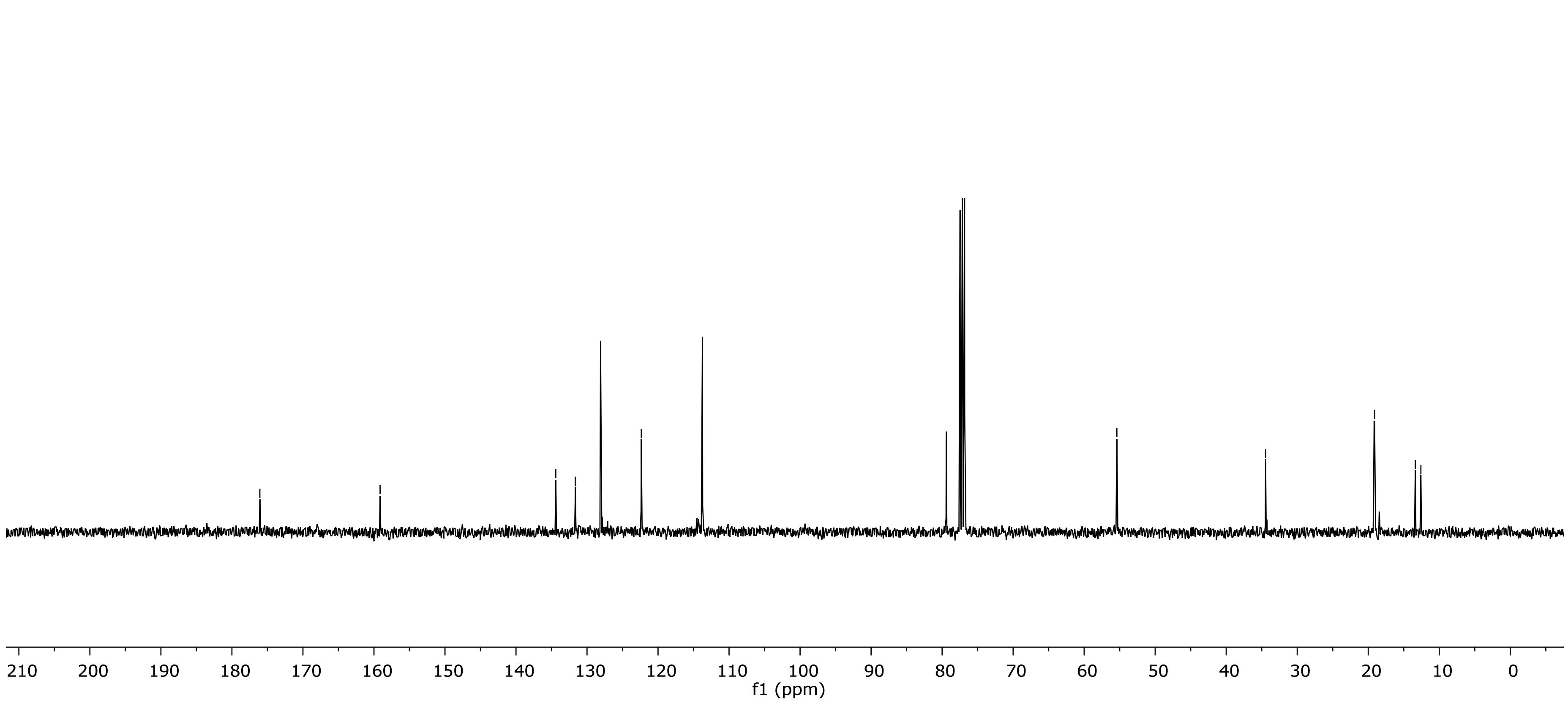

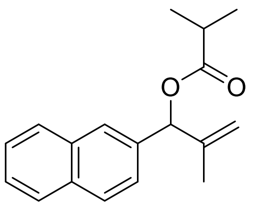

S29

^{1}H, $CDCl_3$, 500 MHz

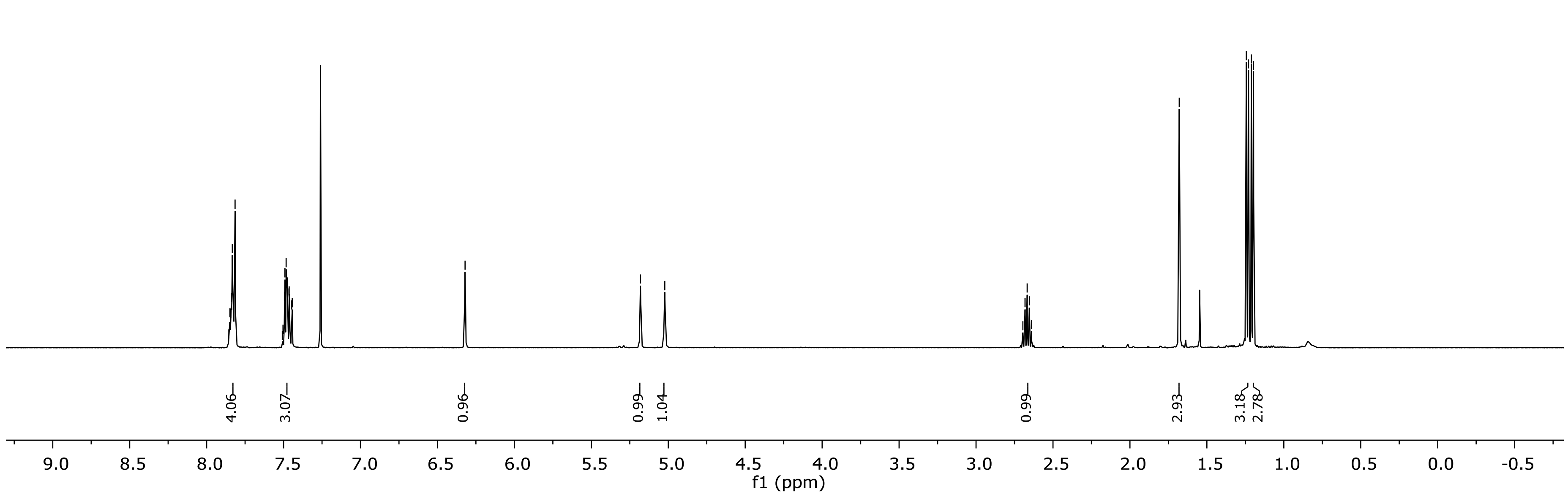

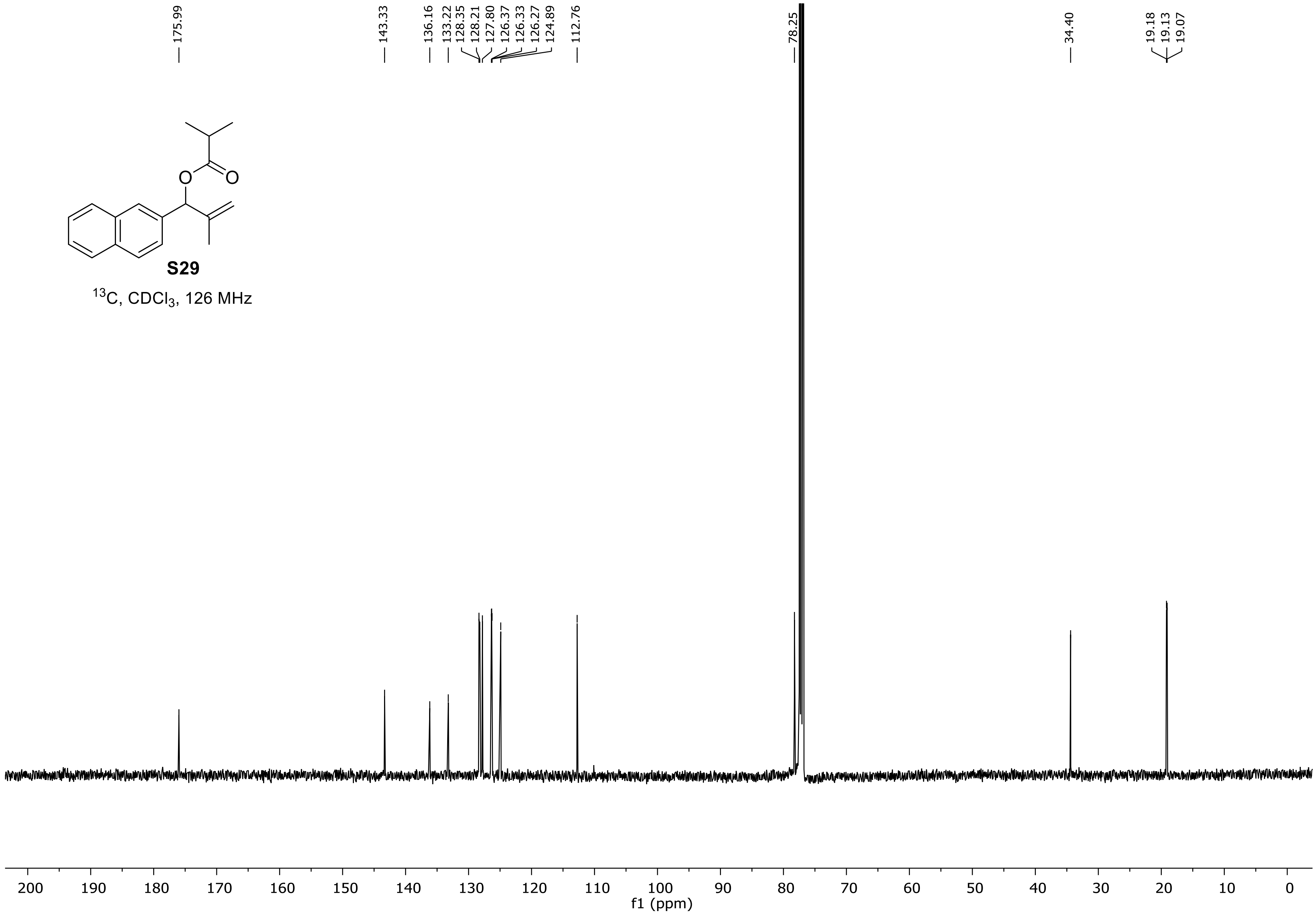
175.99
143.33
136.16
133.22
128.35
128.21
127.80
126.37
126.33
126.27
124.89
112.76
78.25
34.40
19.18
19.13
19.07
S29
13C, CDCl3, 126 MHz
200
190
180
170
160
150
140
130
120
110
100
90
80
70
60
50
40
30
20
10
0
f1 (ppm)

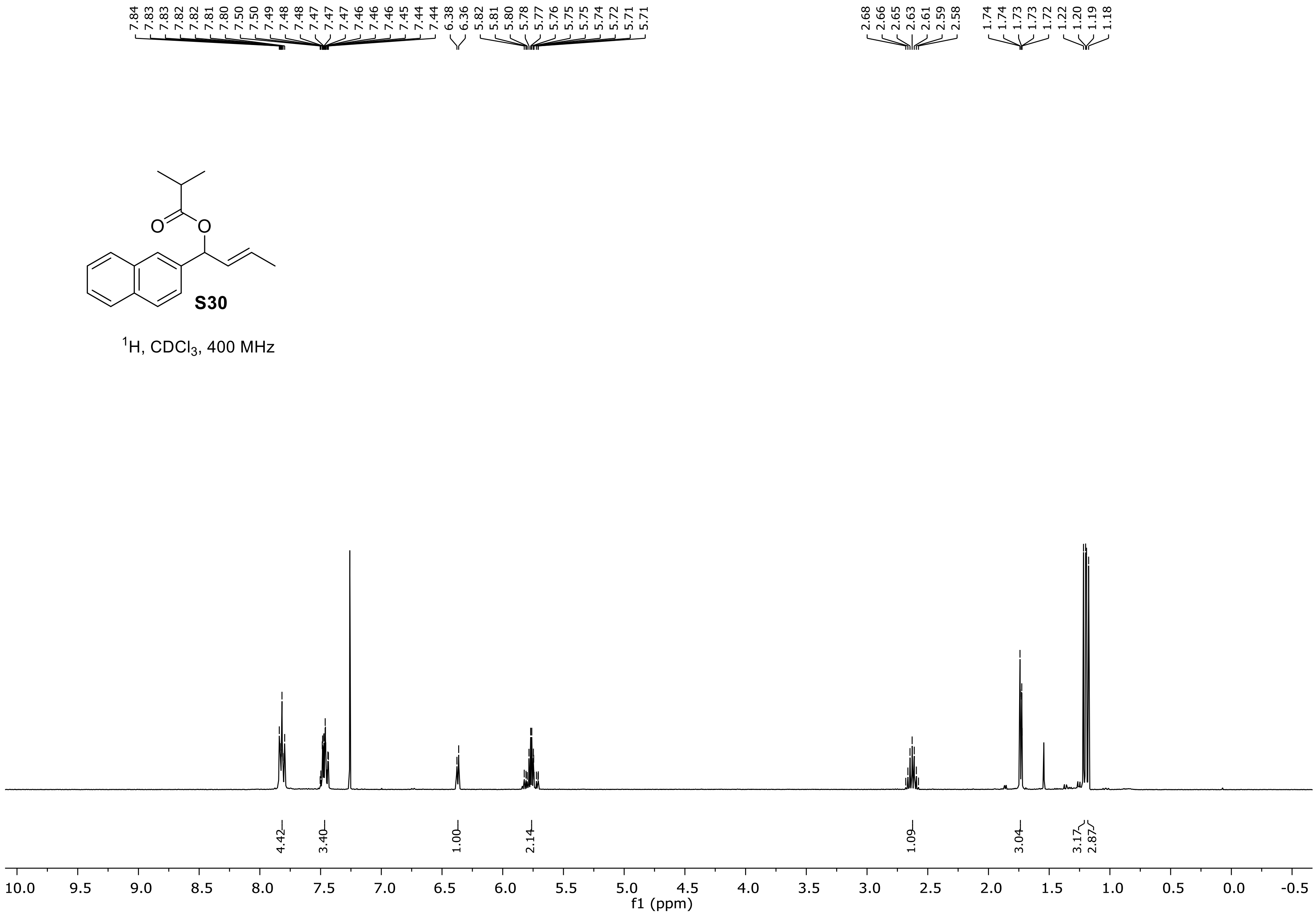
7.84 7.83 7.83 7.82 7.82 7.81 7.80 7.50 7.50 7.49 7.48 7.48 7.47 7.47 7.47 7.46 7.46 7.46 7.45 7.44 7.44 6.38 6.36 5.82 5.81 5.80 5.78 5.77 5.76 5.75 5.75 5.74 5.72 5.71 5.71
2.68 2.66 2.65 2.63 2.61 2.59 2.58 1.74 1.74 1.73 1.73 1.72 1.22 1.20 1.19 1.18
O
O
S30
^{1}H, CDCl$_3$, 400 MHz
4.42 3.40 1.00 2.14 1.09 3.04 3.17 2.87
10.0 9.5 9.0 8.5 8.0 7.5 7.0 6.5 6.0 5.5 5.0 4.5 4.0 3.5 3.0 2.5 2.0 1.5 1.0 0.5 0.0 -0.5
f1 (ppm)

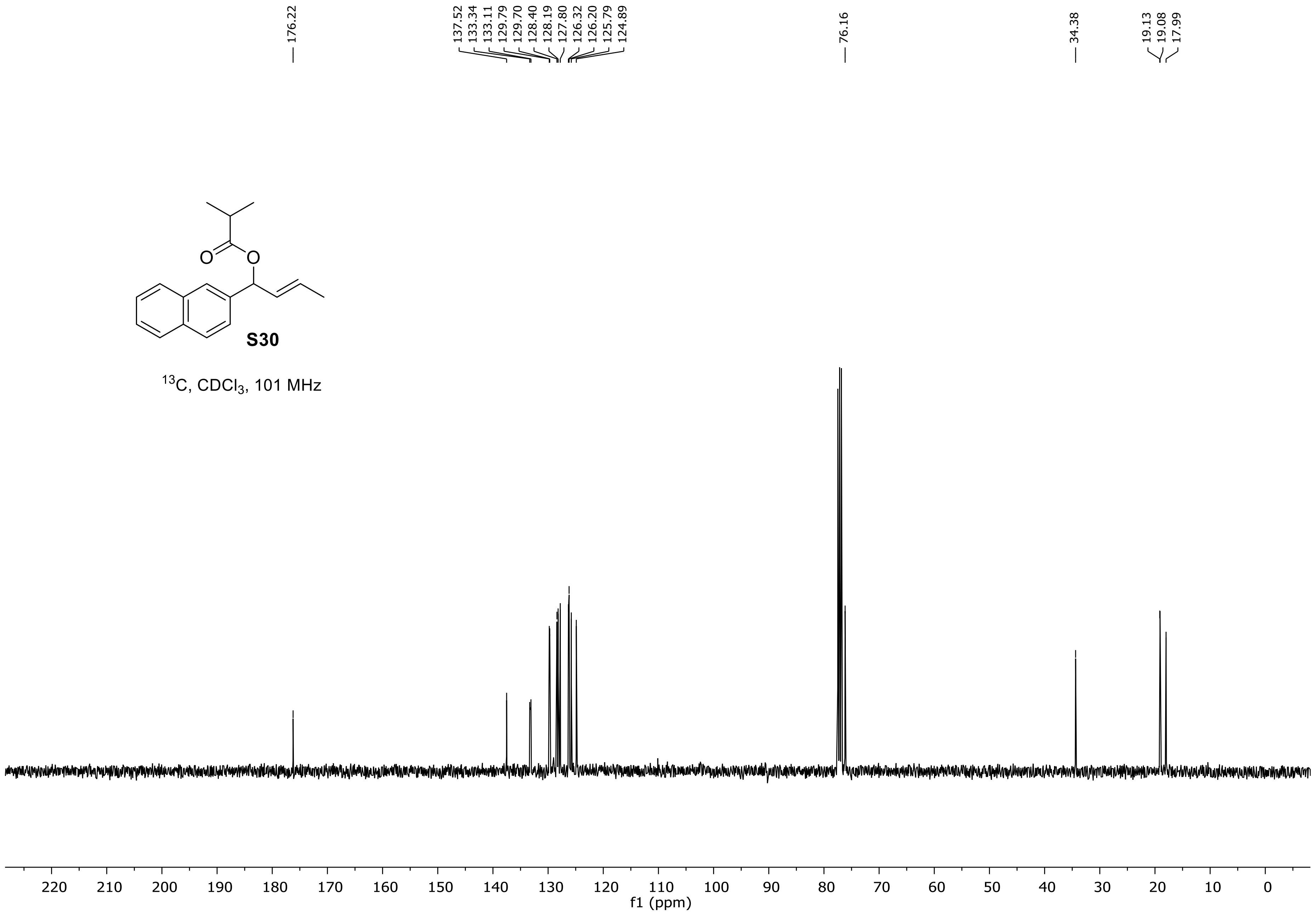
176.22
137.52
133.34
133.11
129.79
129.70
128.40
128.19
127.80
126.32
126.20
125.79
124.89
76.16
34.38
19.13
19.08
17.99
O
O
S30
^{13}C, CDCl$_3$, 101 MHz
220 210 200 190 180 170 160 150 140 130 120 110 100 90 80 70 60 50 40 30 20 10 0
f1 (ppm)

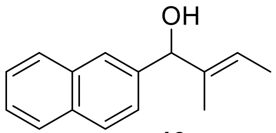

46

^{1}H, $CDCl_3$, 400 MHz

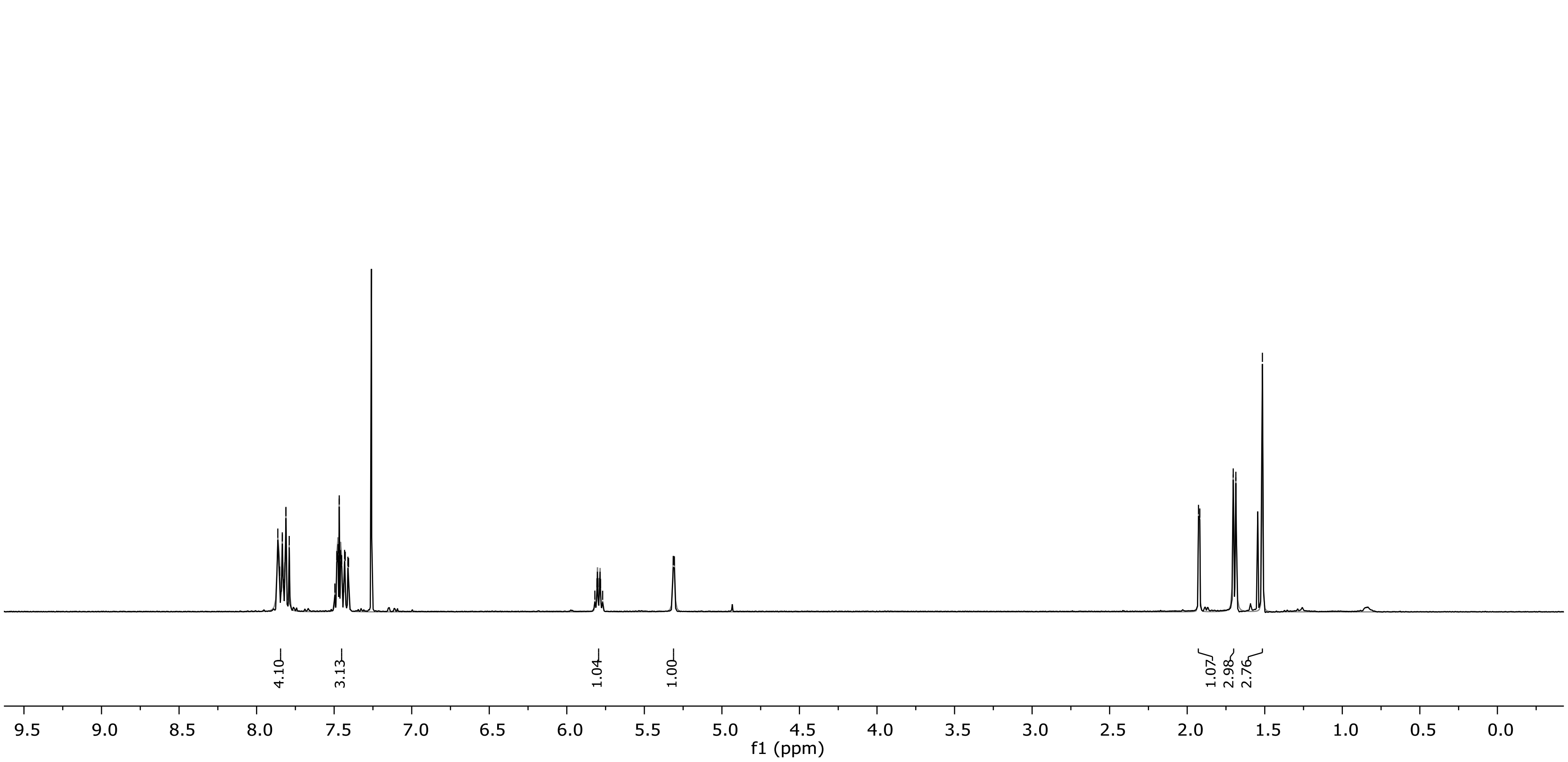

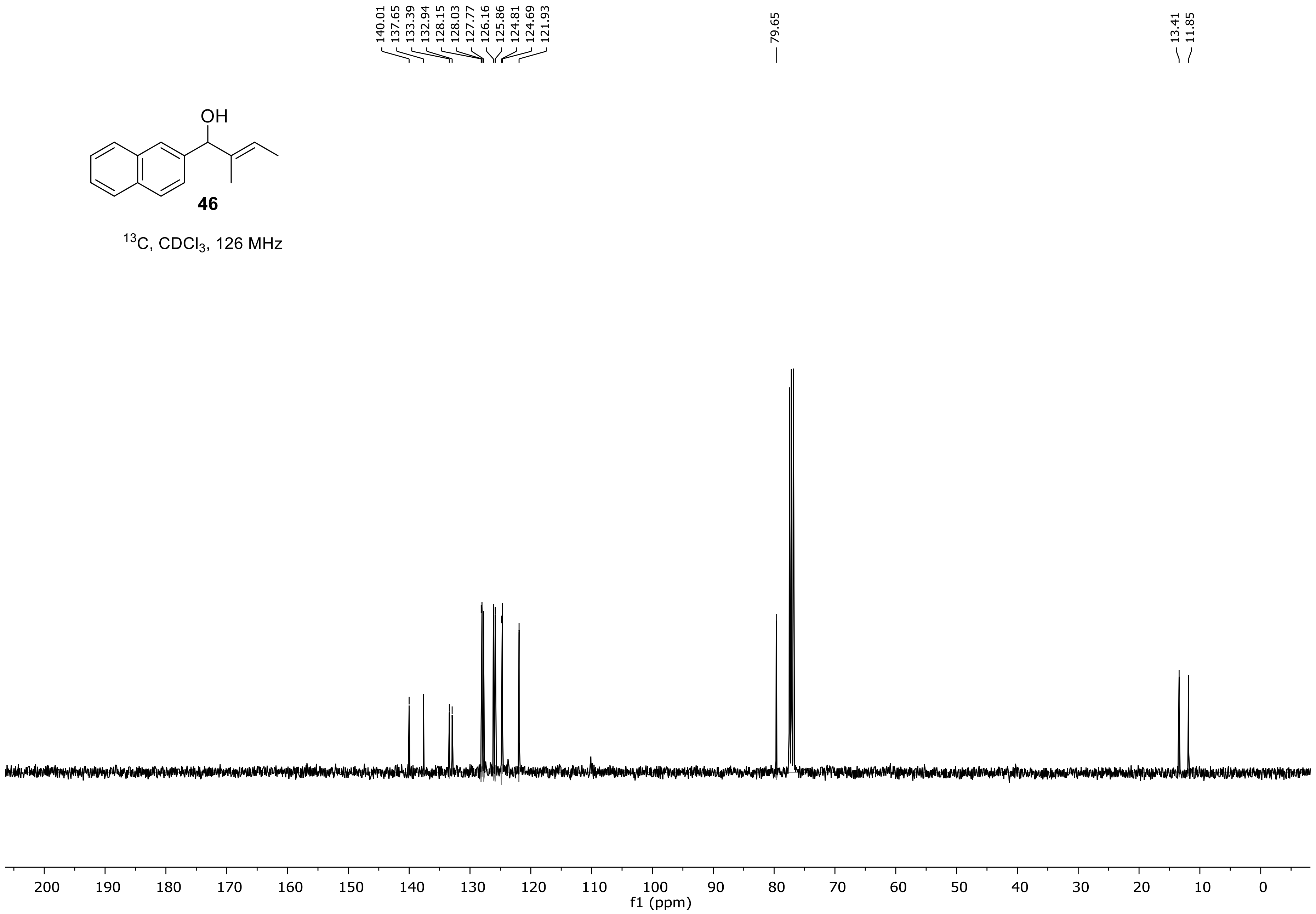
140.01
137.65
133.39
132.94
128.15
128.03
127.77
126.16
125.86
124.81
124.69
121.93
79.65
13.41
11.85
OH
46
^{13}C, $CDCl_3$, 126 MHz
200 190 180 170 160 150 140 130 120 110 100 90 80 70 60 50 40 30 20 10 0
f1 (ppm)

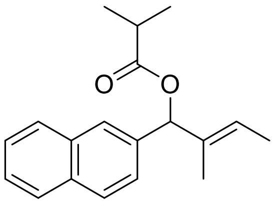

S31

^{1}H, $CDCl_3$, 500 MHz

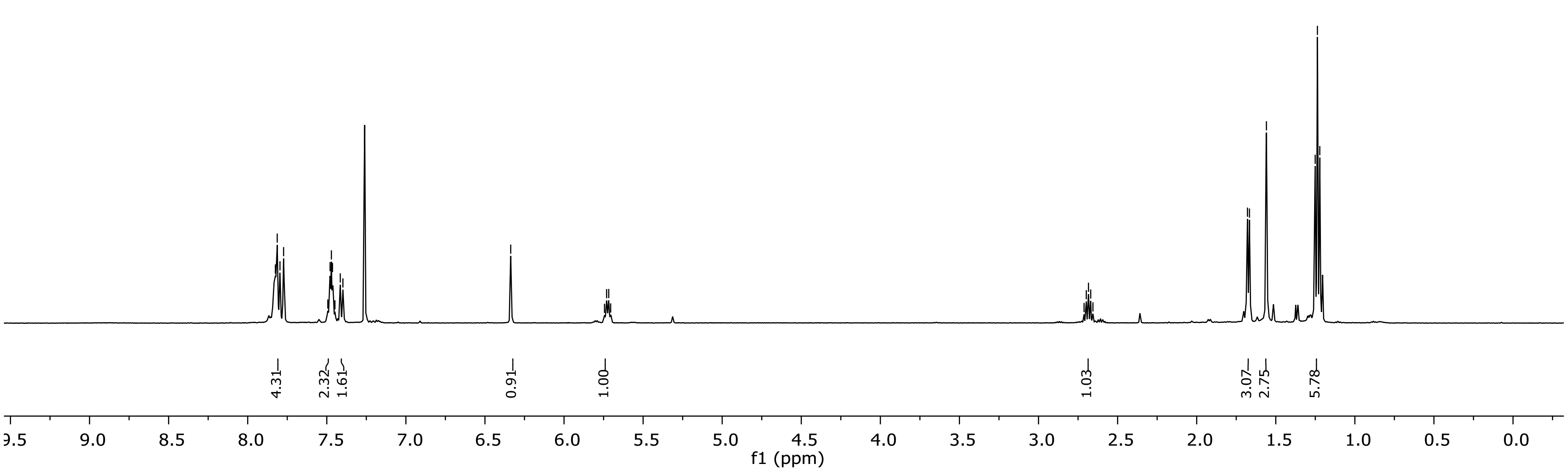

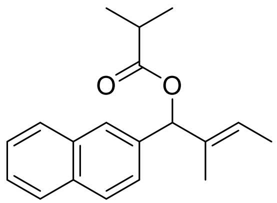

S31

^{13}C, $CDCl_3$, 101 MHz

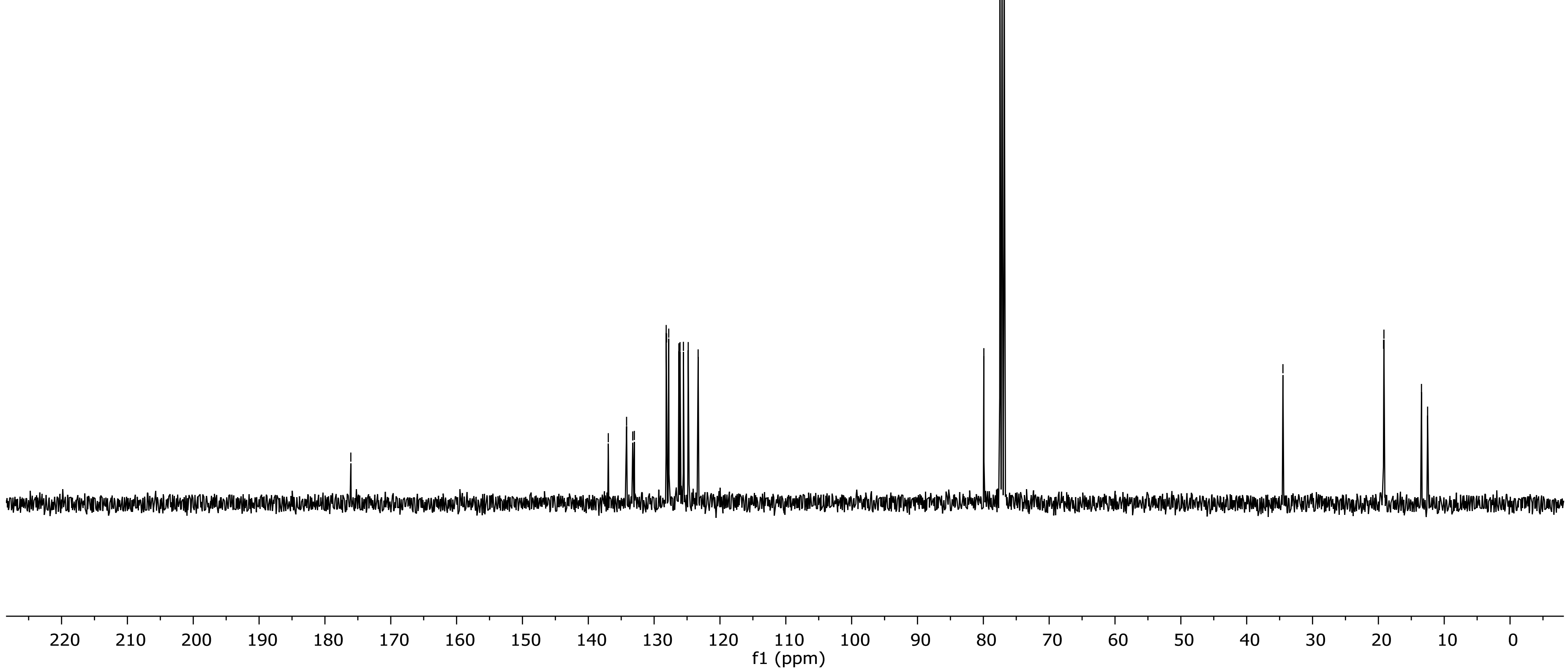

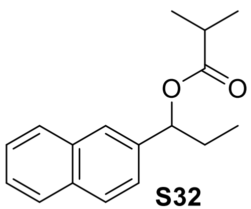

S32

^{1}H, $CDCl_3$, 500 MHz

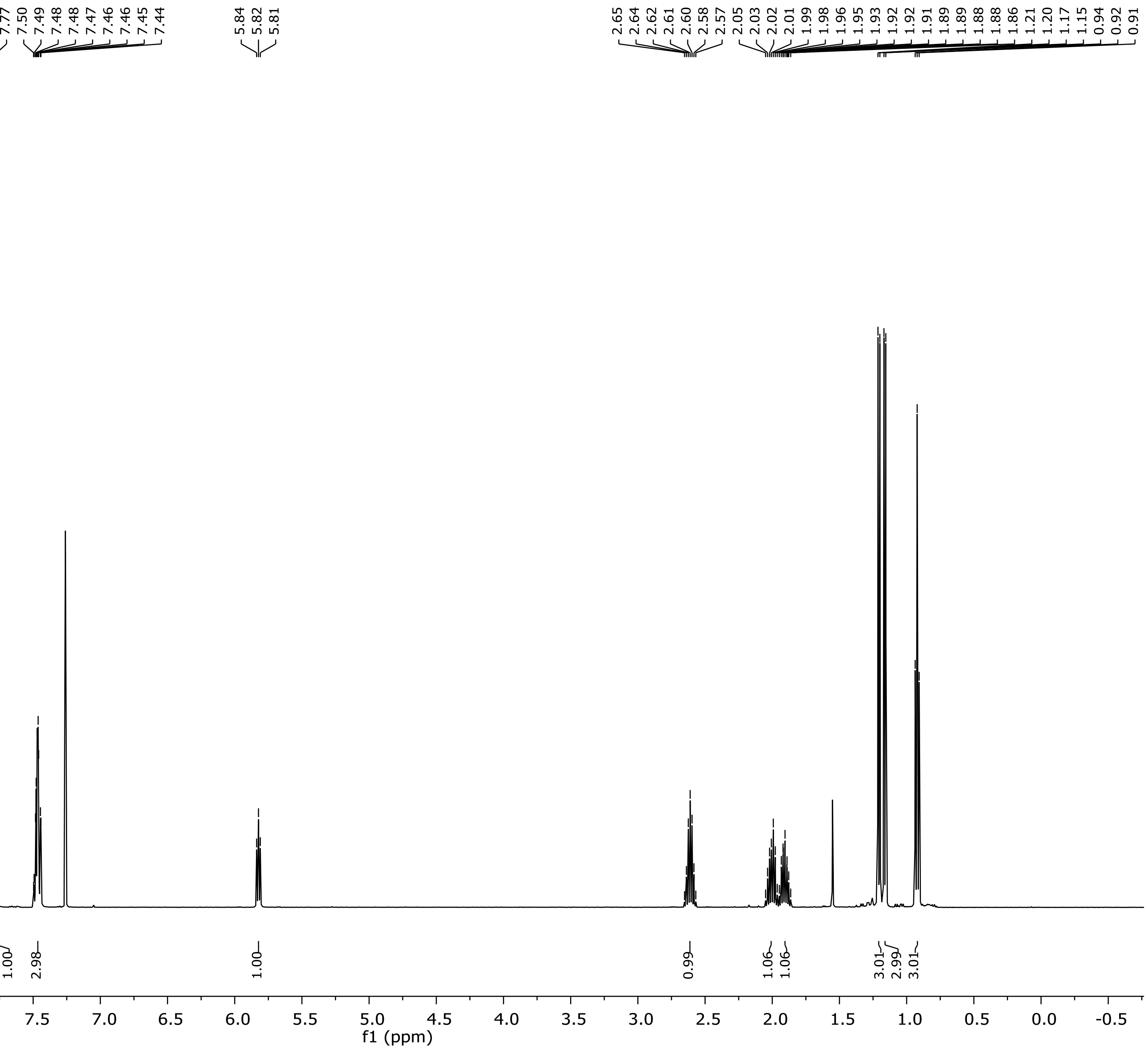

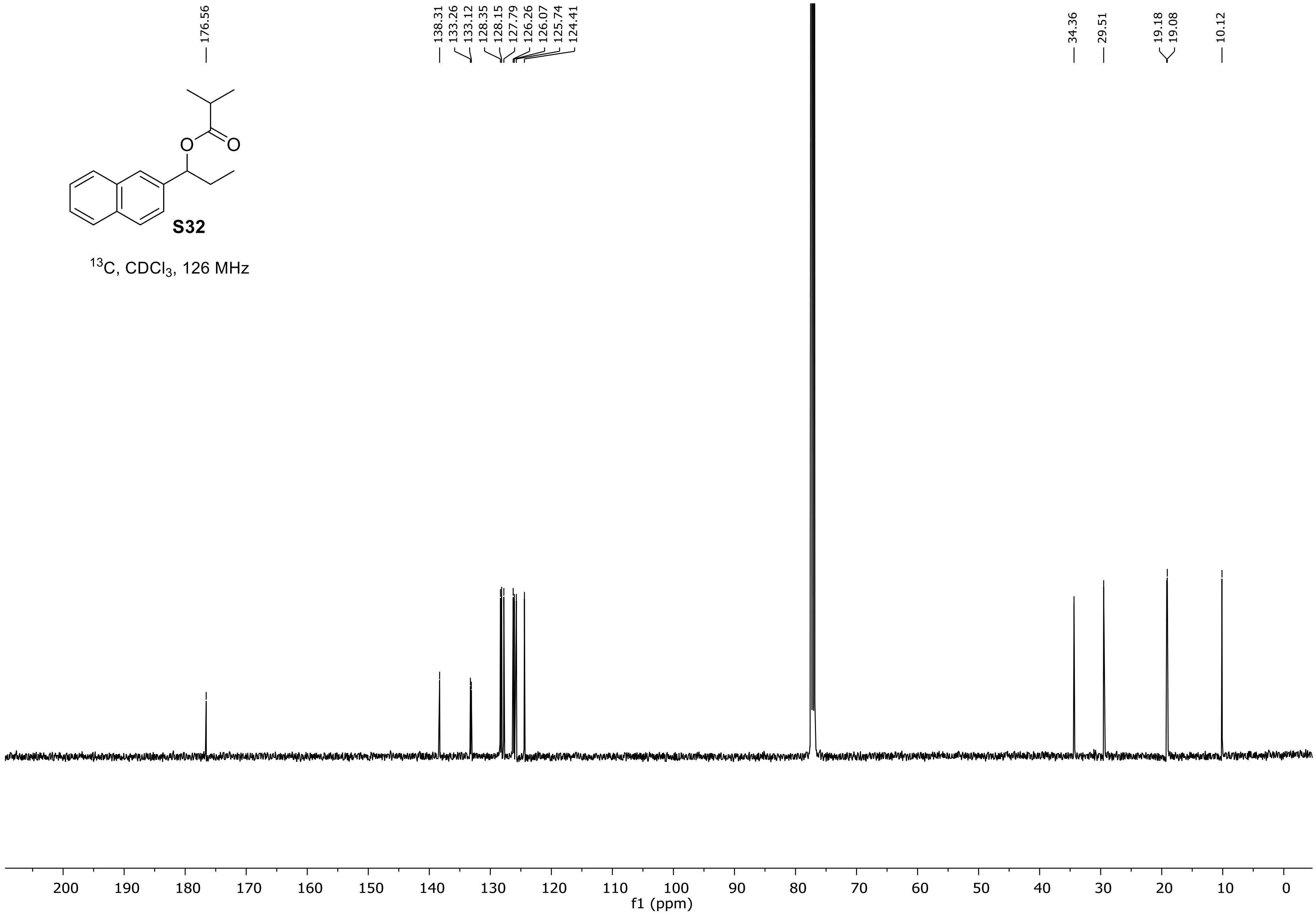
176.56
138.31
133.26
133.12
128.35
128.15
127.79
126.26
126.07
125.74
124.41
34.36
29.51
19.18
19.08
10.12
S32
^{13}C, $CDCl_3$, 126 MHz
f1 (ppm)

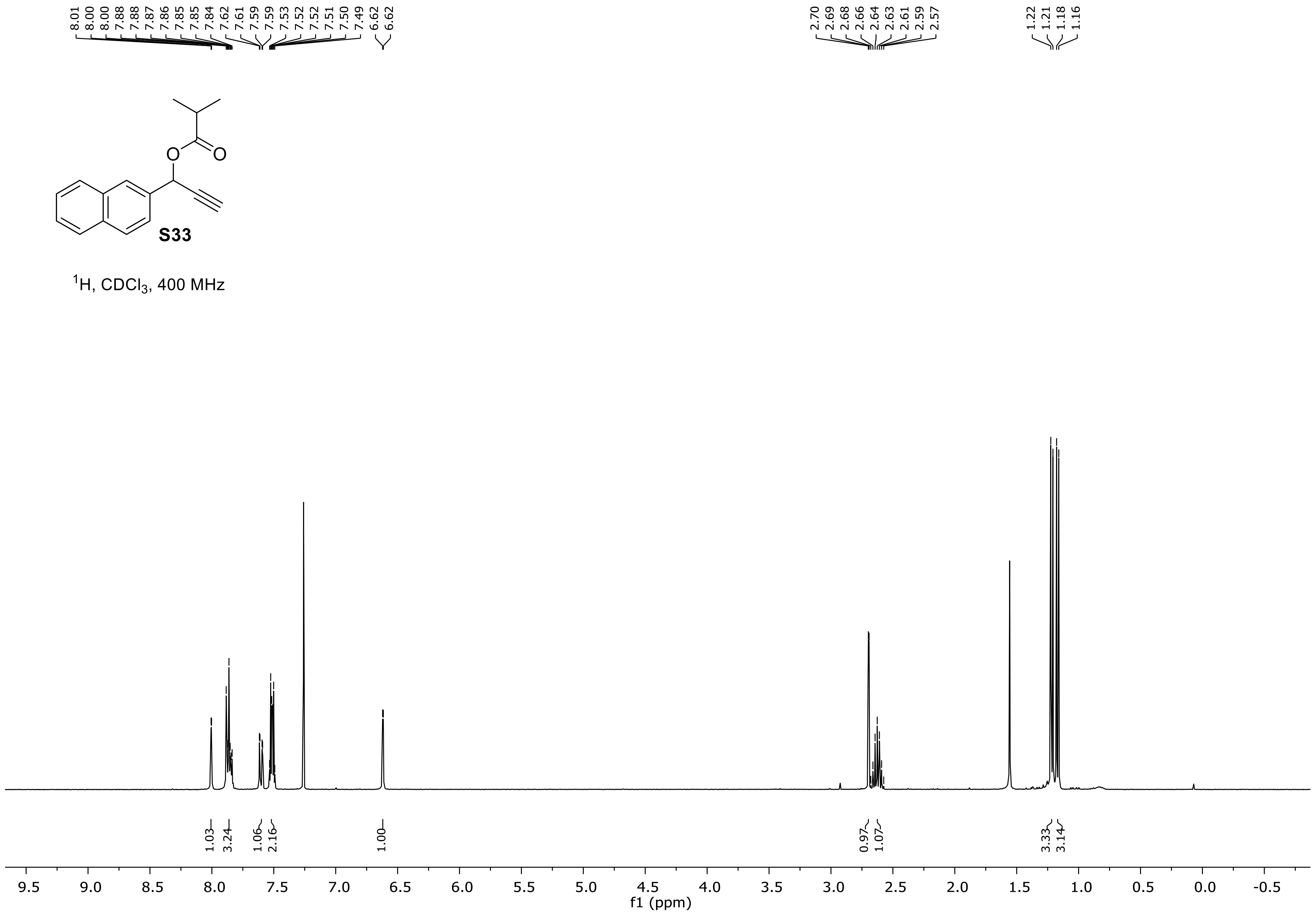
S33
1H, CDCl3, 400 MHz
f1 (ppm)

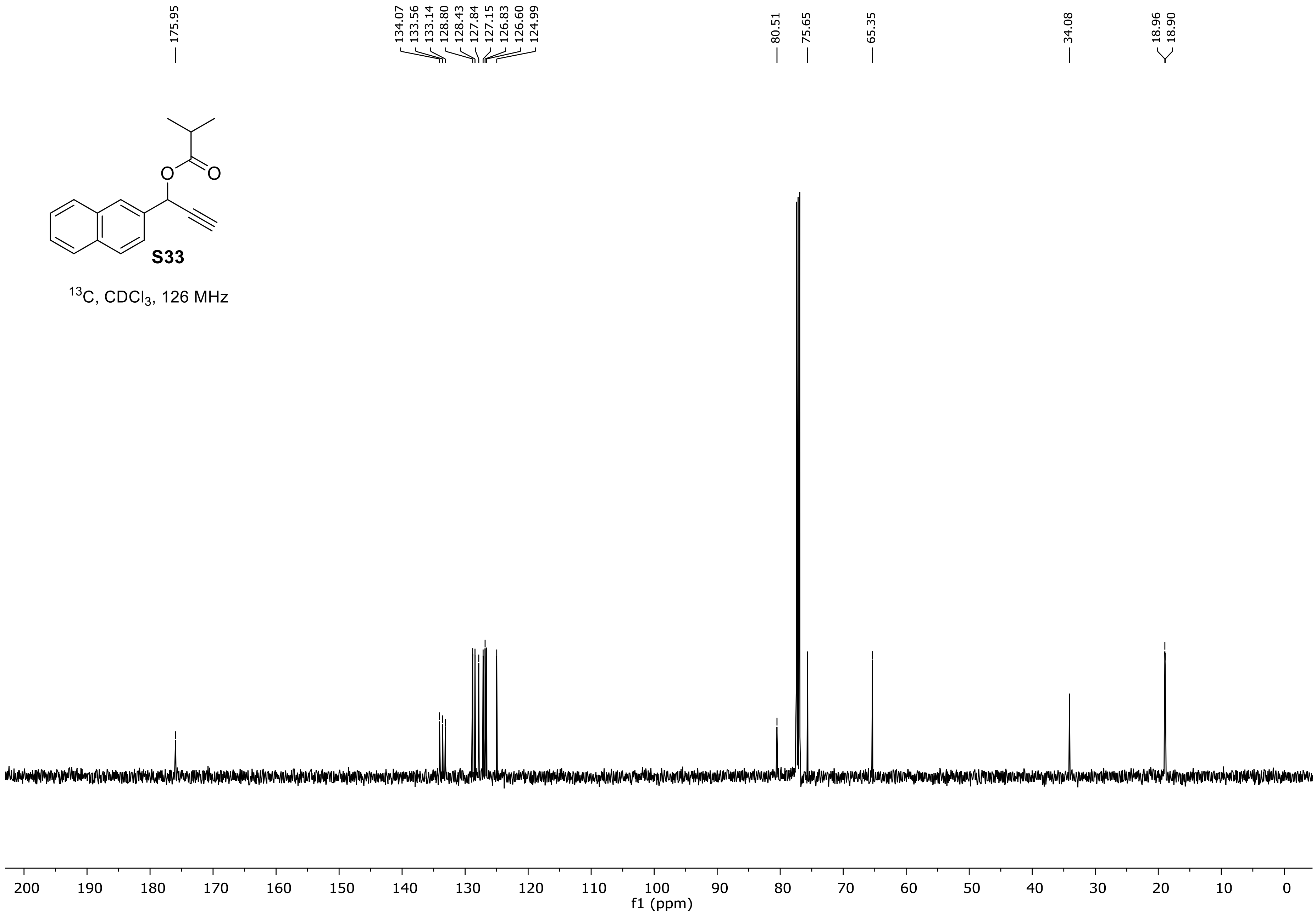
175.95
134.07
133.56
133.14
128.80
128.43
127.84
127.15
126.83
126.60
124.99
80.51
75.65
65.35
34.08
18.96
18.90
O
O
S33
^{13}C, CDCl$_3$, 126 MHz
200 190 180 170 160 150 140 130 120 110 100 90 80 70 60 50 40 30 20 10 0
f1 (ppm)

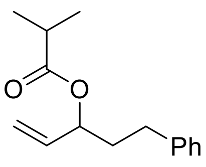

S34

^{1}H, $CDCl_3$, 500 MHz

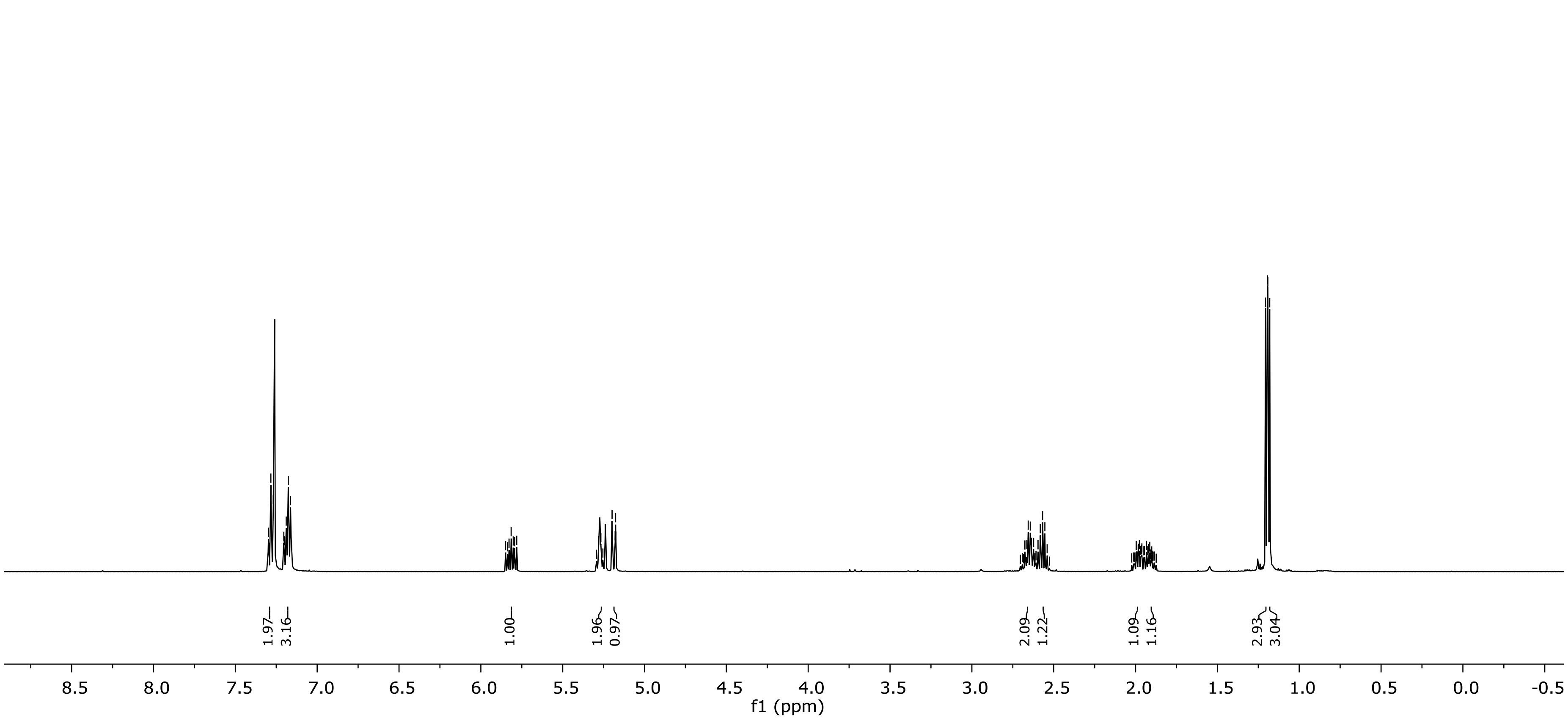

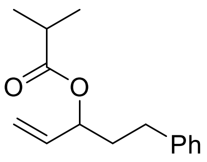

S34

^{13}C, $CDCl_3$, 126 MHz

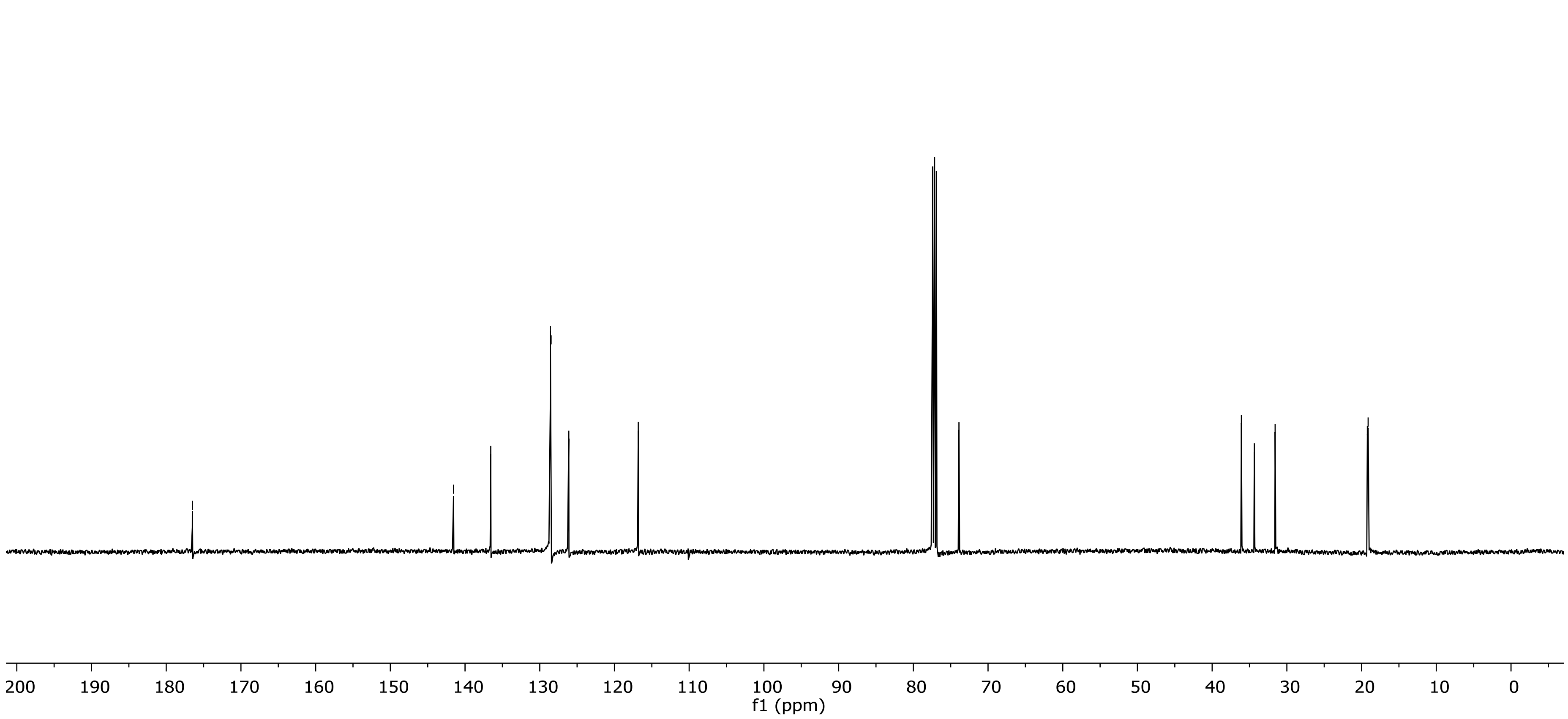

HPLC Traces

HPLC Data for **15**: Chiral HPLC analysis Chiralcel OD-H (95:5 hexane : IPA, flow rate 1.0 mL min^{-1}, 211 nm, 30 °C) t_R (*R*): 11.0 min, t_R (*S*): 13.8 min, 84 % *ee*.

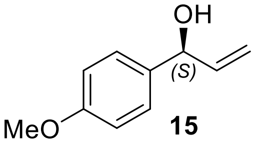

15

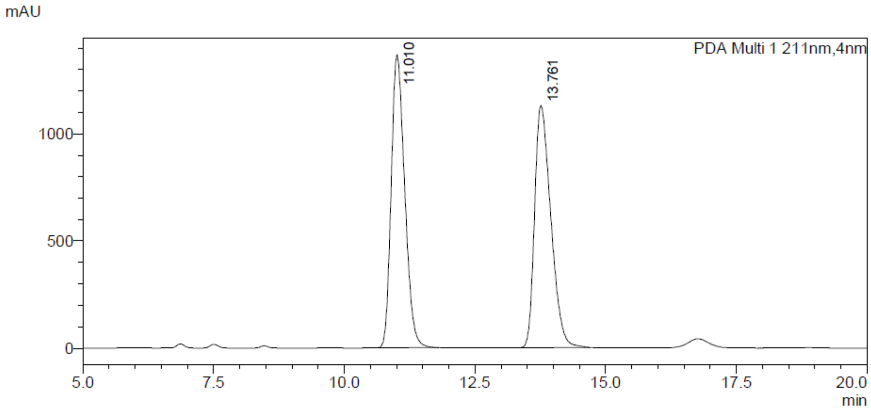

<Peak Table>

PDA Ch1 211nm

Peak#	Ret. Time	Area%
1	11.010	50.204
2	13.761	49.796
Total		100.000

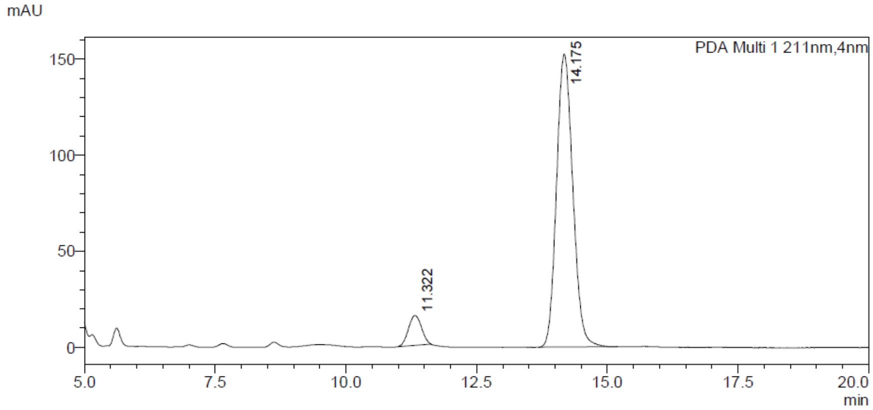

<Peak Table>

PDA Ch1 211nm

Peak#	Ret. Time	Area%
1	11.322	7.516
2	14.175	92.484
Total		100.000

HPLC Data for **S1**: Chiral HPLC analysis Chiralcel OJ-H (99:1 hexane : IPA, flow rate 1.0 mL min^{-1}, 211 nm, 30 °C) t_R (*R*): 8.7 min, t_R (*S*): 11.2 min, 83 % *ee*.

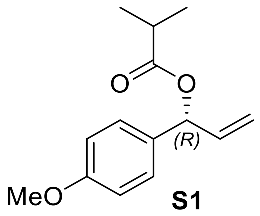

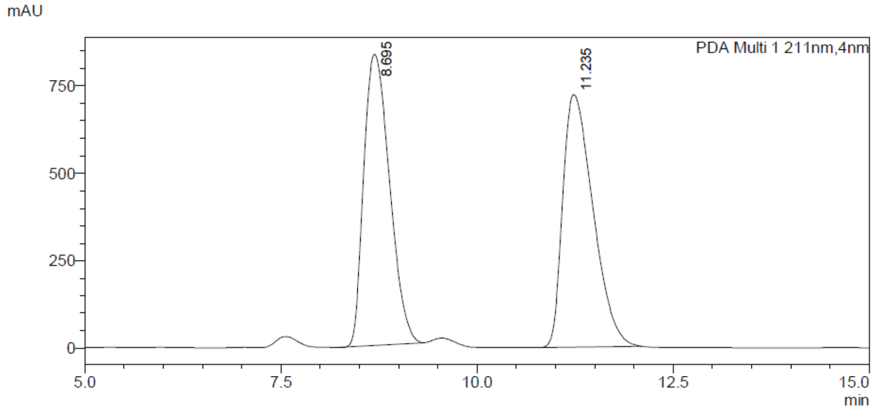

<Peak Table>

PDA Ch1 211nm

Peak#	Ret. Time	Area%
1	8.695	49.500
2	11.235	50.500
Total		100.000

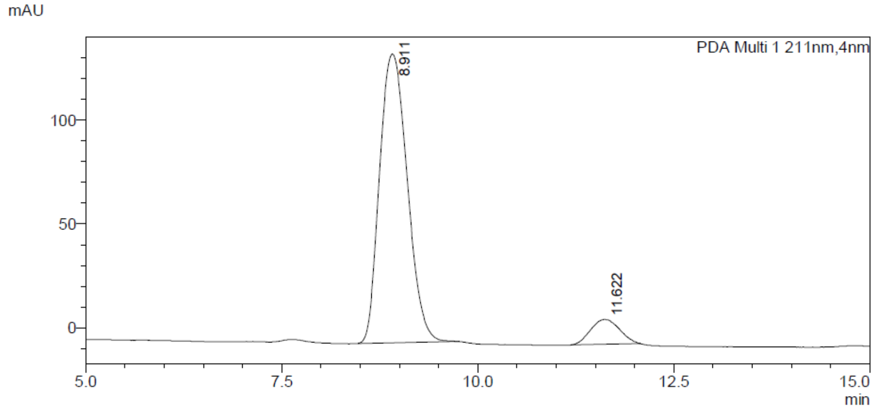

<Peak Table>

PDA Ch1 211nm

Peak#	Ret. Time	Area%
1	8.911	91.705
2	11.622	8.295
Total		100.000

HPLC Data for **19**: Chiral HPLC analysis Chiralpak IB (95:5 hexane : IPA, flow rate 0.8 mL min^{-1}, 211 nm, 30 °C) t_R (*R*): 8.6 min, t_R (*S*): 9.4 min, 64% *ee*.

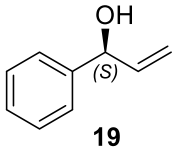

19

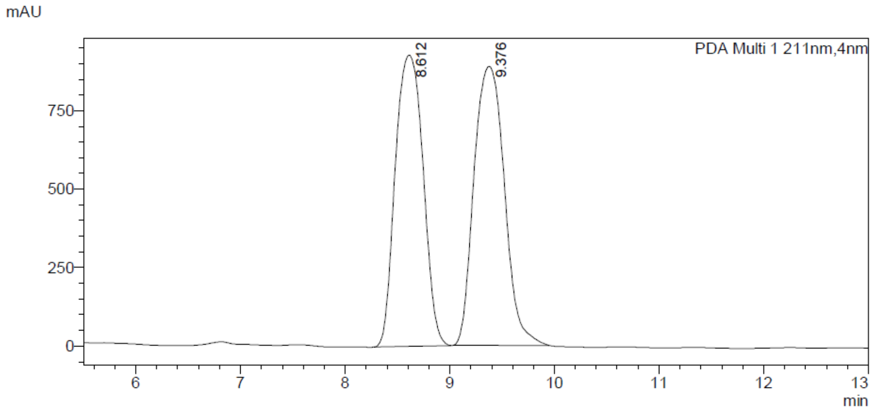

<Peak Table>

PDA Ch1 211nm

Peak#	Ret. Time	Area%
1	8.612	48.858
2	9.376	51.142
Total		100.000

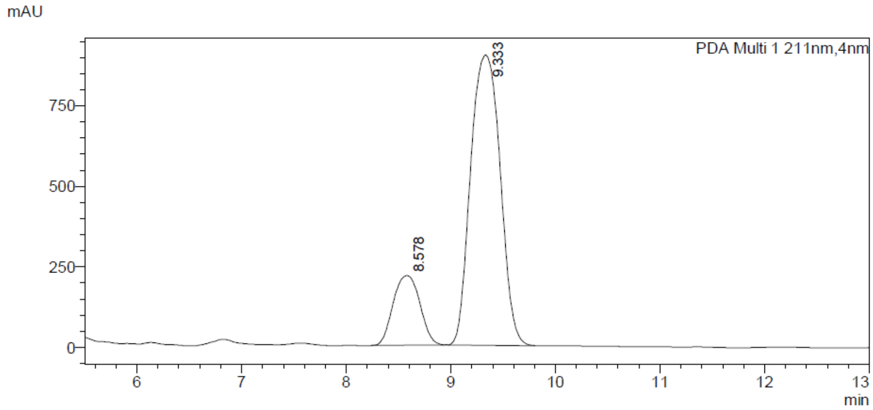

<Peak Table>

PDA Ch1 211nm

Peak#	Ret. Time	Area%
1	8.578	17.848
2	9.333	82.152
Total		100.000

HPLC Data for **S2**: Chiral HPLC analysis Chiralcel OJ-H (99:1 hexane : IPA, flow rate 1.0 mL min^{-1}, 220 nm, 30 °C) t_R (*R*): 5.8 min, t_R (*S*): 6.6 min, 90 % *ee*.

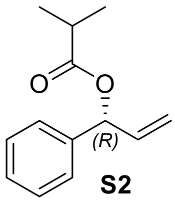

S2

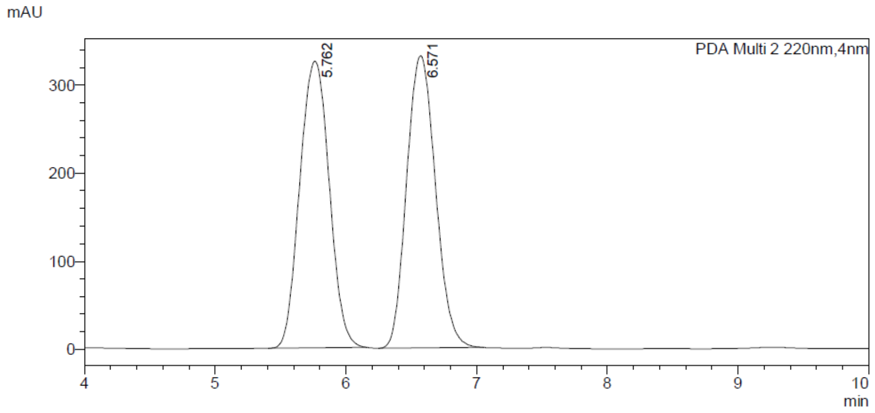

<Peak Table>

PDA Ch2 220nm

Peak#	Ret. Time	Area%
1	5.762	50.062
2	6.571	49.938
Total		100.000

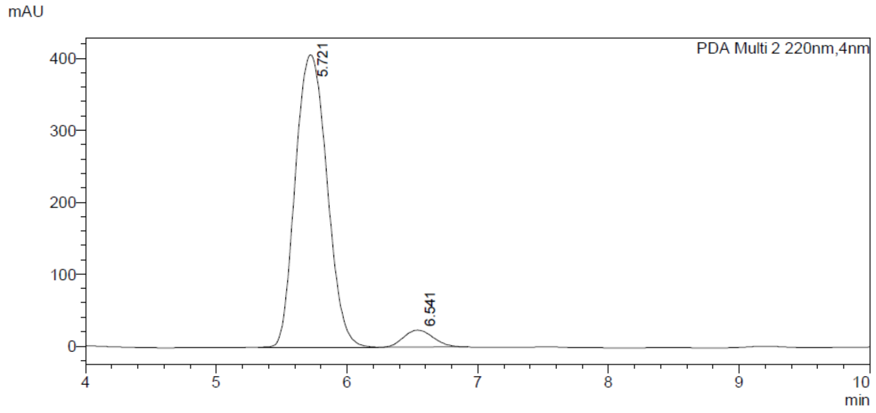

<Peak Table>

PDA Ch2 220nm

Peak#	Ret. Time	Area%
1	5.721	94.811
2	6.541	5.189
Total		100.000

HPLC Data for **20**: Chiral HPLC analysis Chiralpak AD-H (99:1 hexane : IPA, flow rate 1.0 mL min^{-1}, 211 nm, 30 °C) t_R (*R*): 20.5 min, t_R (*S*): 22.5 min, 66% *ee*.

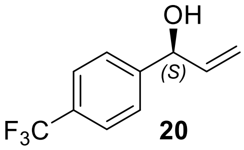

20

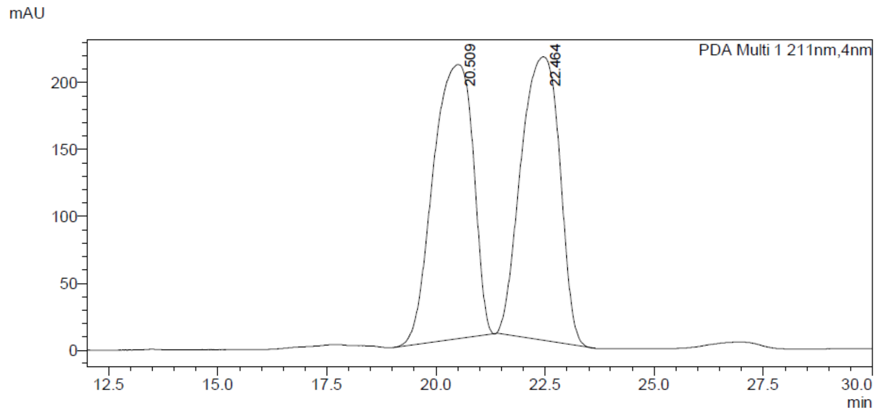

<Peak Table>

PDA Ch1 211nm

Peak#	Ret. Time	Area%
1	20.509	50.125
2	22.464	49.875
Total		100.000

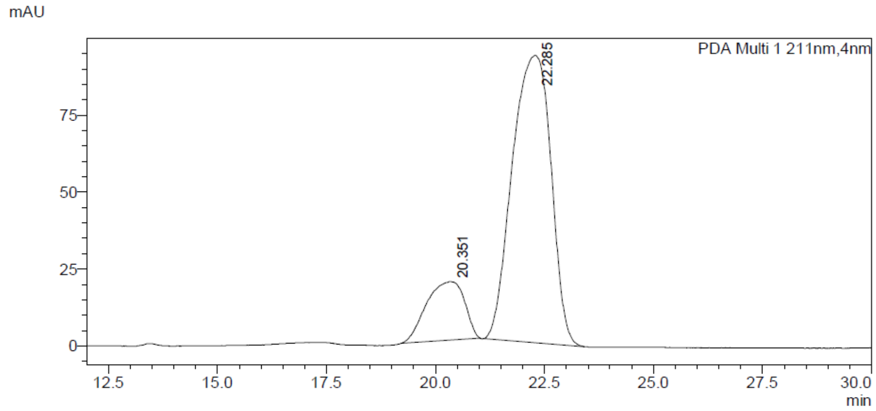

<Peak Table>

PDA Ch1 211nm

Peak#	Ret. Time	Area%
1	20.351	16.739
2	22.285	83.261
Total		100.000

HPLC Data for **S3**: Chiral HPLC analysis Chiralpak AD-H (99:1 hexane : IPA, flow rate 1.0 mL min^{-1}, 211 nm, 30 °C) t_R (*R*): 4.0 min, t_R (*S*): 5.2 min, 64% *ee*.

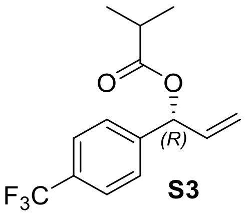

S3

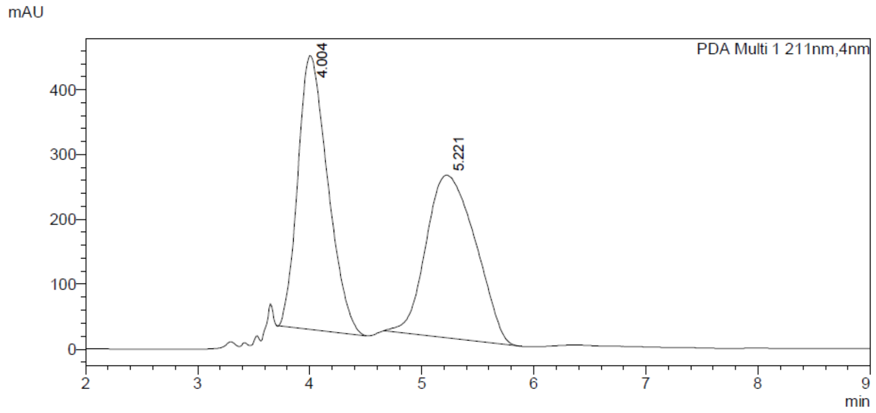

<Peak Table>

PDA Ch1 211nm

Peak#	Ret. Time	Area%
1	4.004	50.495
2	5.221	49.505
Total		100.000

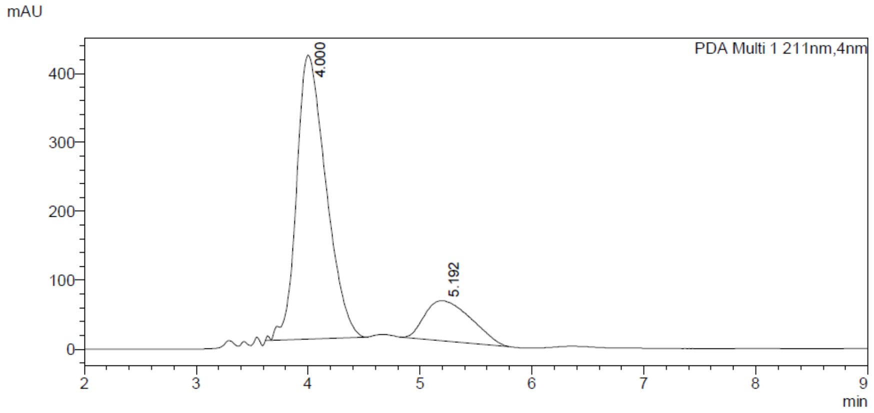

<Peak Table>

PDA Ch1 211nm

Peak#	Ret. Time	Area%
1	4.000	81.833
2	5.192	18.167
Total		100.000

HPLC Data for **S4**: Chiral HPLC analysis Chiralcel OJ-H (95:5 hexane : IPA, flow rate 1.0 mL min^{-1}, 220 nm, 30 °C) t_R (*R*): 4.5 min, t_R (*S*): 4.9 min, 81% *ee*.

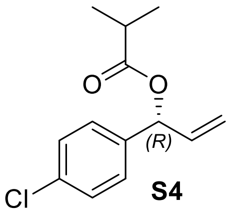

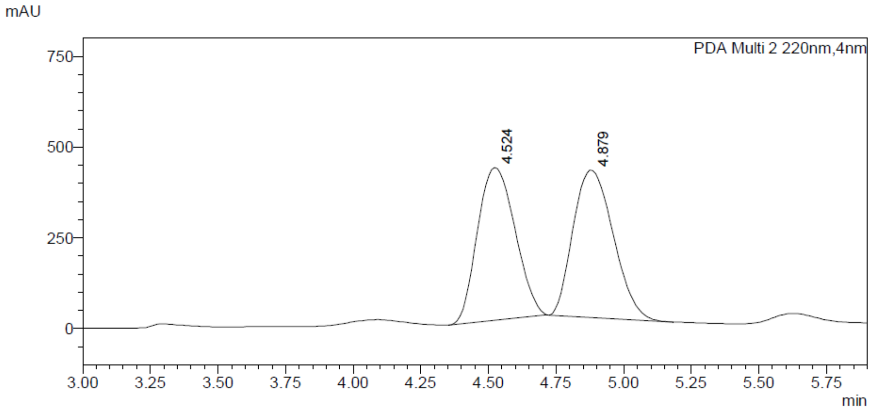

<Peak Table>

PDA Ch2 220nm

Peak#	Ret. Time	Area%
1	4.524	49.383
2	4.879	50.617
Total		100.000

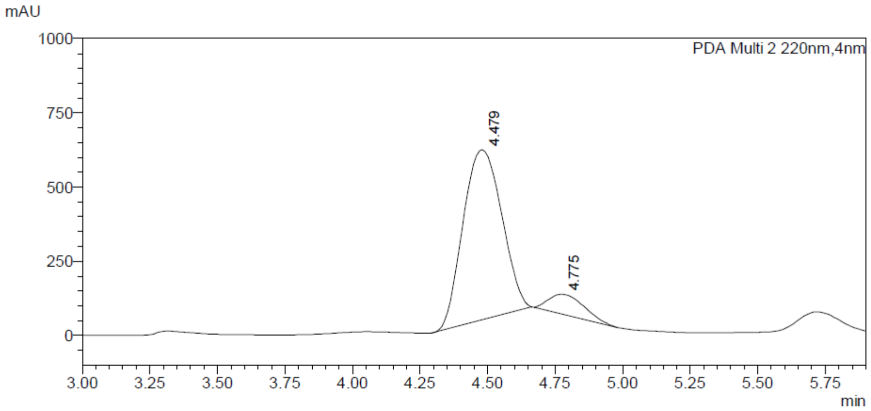

<Peak Table>

PDA Ch2 220nm

Peak#	Ret. Time	Area%
1	4.479	90.525
2	4.775	9.475
Total		100.000

HPLC Data for **21**: Chiral HPLC analysis Chiralpak AD-H (98:2 hexane : IPA, flow rate 1.0 mL min^{-1}, 254 nm, 30 °C) t_R (*R*): 17.8 min, t_R (*S*): 18.9 min, 37% *ee*.

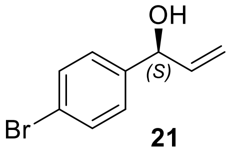

21

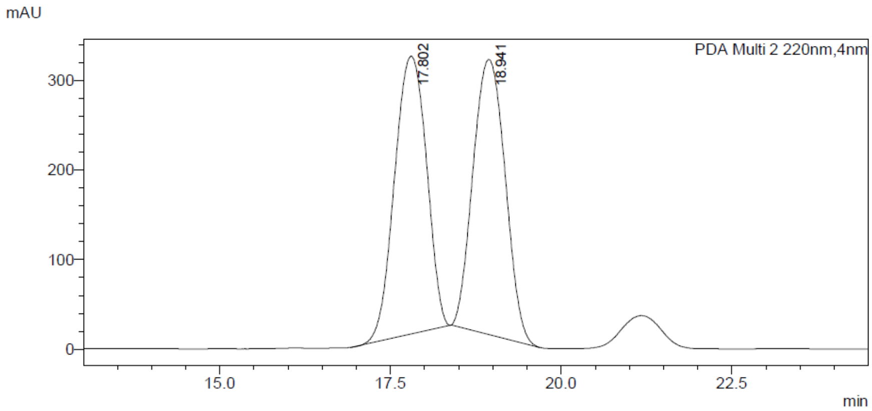

<Peak Table>

PDA Ch2 220nm

Peak#	Ret. Time	Area%
1	17.802	50.125
2	18.941	49.875
Total		100.000

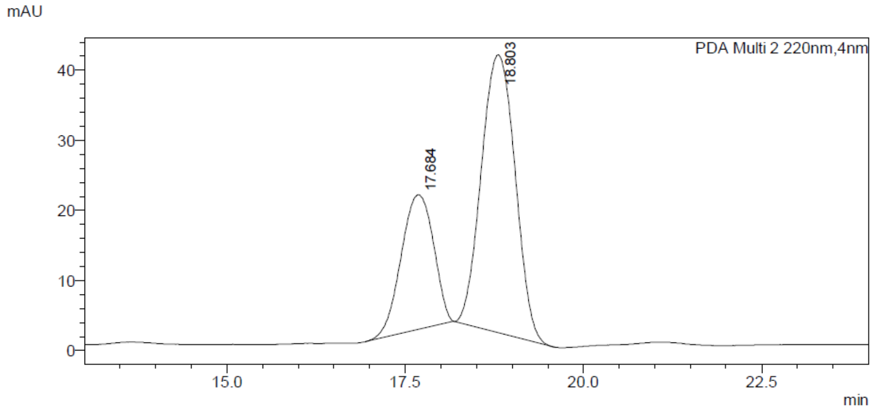

<Peak Table>

PDA Ch2 220nm

Peak#	Ret. Time	Area%
1	17.684	31.762
2	18.803	68.238
Total		100.000

HPLC Data for **S5**: Chiral HPLC analysis Chiralpak AD-H (99.8:0.2 hexane : IPA, flow rate 1.0 mL min^{-1}, 220 nm, 30 °C) t_R (*R*): 6.2 min, t_R (*S*): 7.5 min, 68% *ee*.

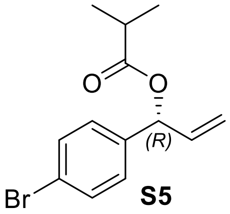

S5

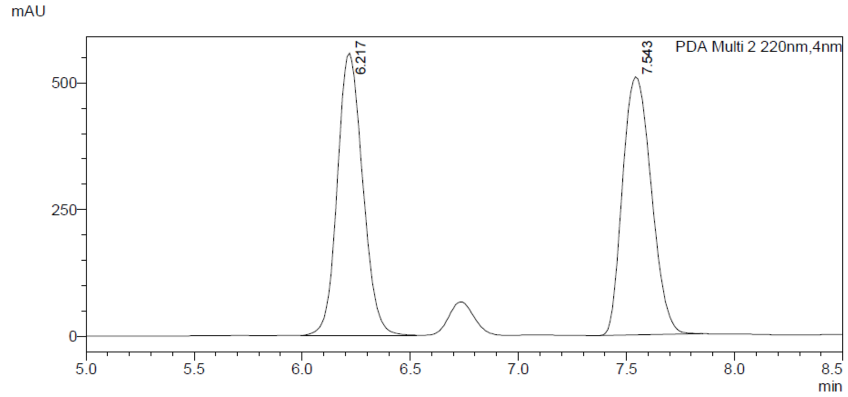

<Peak Table>

PDA Ch2 220nm

Peak#	Ret. Time	Area%
1	6.217	49.132
2	7.543	50.868
Total		100.000

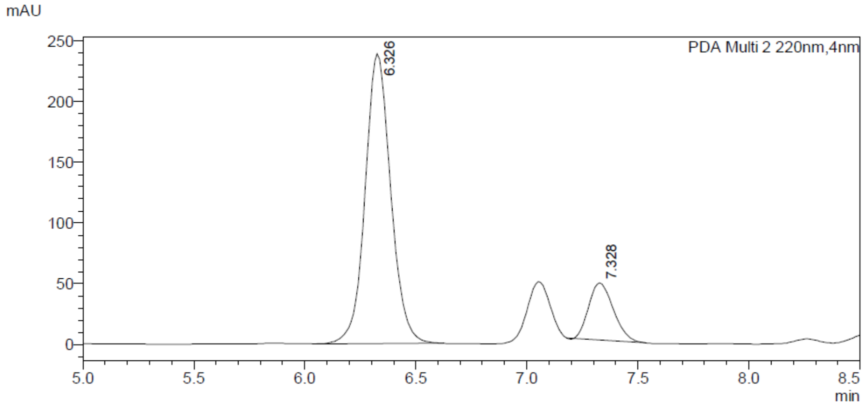

<Peak Table>

PDA Ch2 220nm

Peak#	Ret. Time	Area%
1	6.326	84.060
2	7.328	15.940
Total		100.000

HPLC Data for **23**: Chiral HPLC analysis Chiralcel OD-H (97.5:2.5 hexane : IPA, flow rate 0.5 mL min-1, 220 nm, 30 °C) t_R (*R*): 46.7 min, t_R (*S*): 50.3 min, 71% ee.

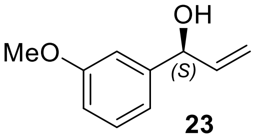

23

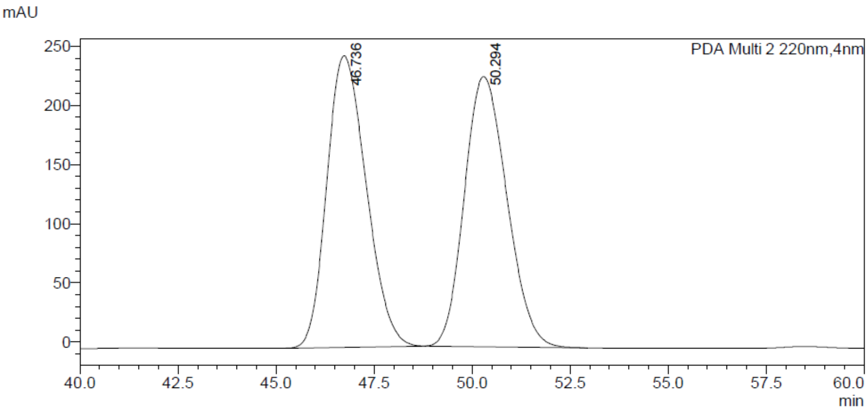

<Peak Table>

PDA Ch2 220nm

Peak#	Ret. Time	Area%
1	46.736	49.999
2	50.294	50.001
Total		100.000

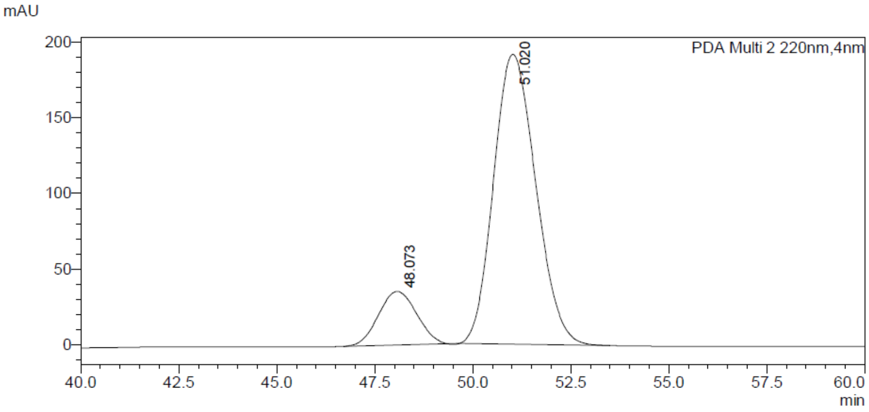

<Peak Table>

PDA Ch2 220nm

Peak#	Ret. Time	Area%
1	48.073	14.256
2	51.020	85.744
Total		100.000

HPLC Data for **S6**: Chiral HPLC analysis Chiralcel OJ-H (99:1 hexane : IPA, flow rate 1.0 mL min-1, 220 nm, 30 °C) t_R (*R*): 7.1 min, t_R (*S*): 8.1 min, 93 % ee.

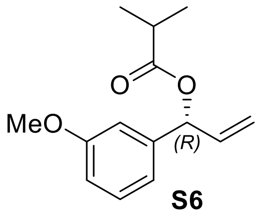

S6

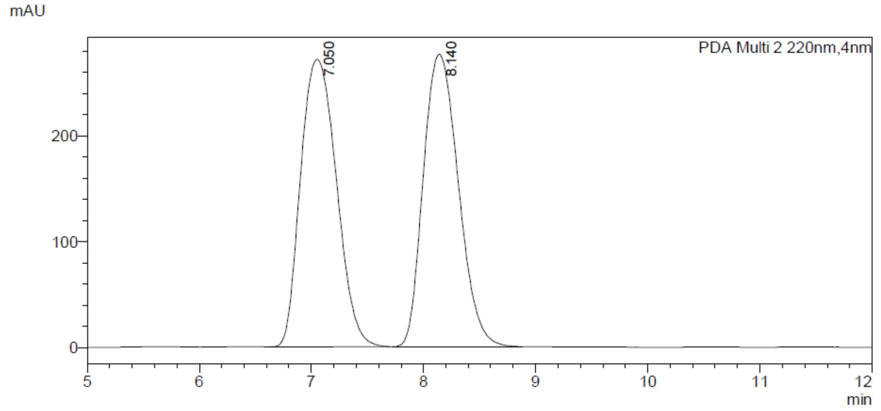

<Peak Table>

PDA Ch2 220nm

Peak#	Ret. Time	Area%
1	7.050	50.138
2	8.140	49.862
Total		100.000

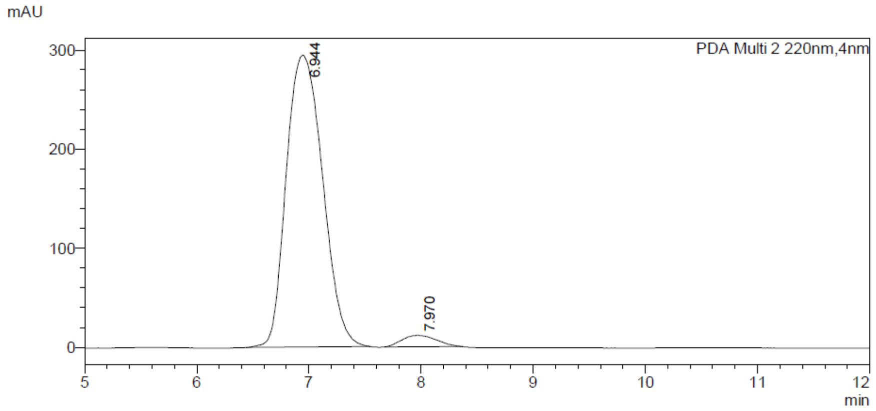

<Peak Table>

PDA Ch2 220nm

Peak#	Ret. Time	Area%
1	6.944	96.497
2	7.970	3.503
Total		100.000

HPLC Data for **24**: Chiral HPLC analysis Chiralcel OJ-H (99.8:0.2 hexane : IPA, flow rate 1.0 mL min^{-1}, 220 nm, 30 °C) t_R (*S*): 30.5 min, t_R (*R*): 35.0 min, 72% *ee*.

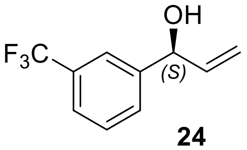

24

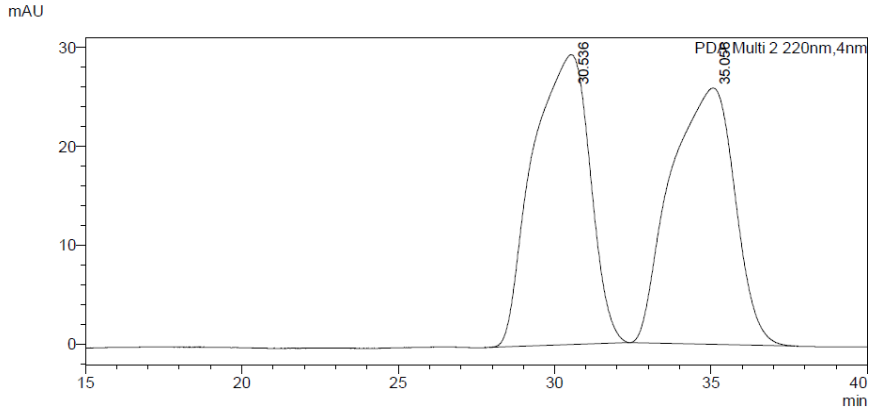

<Peak Table>

PDA Ch2 220nm

Peak#	Ret. Time	Area%
1	30.536	50.253
2	35.056	49.747
Total		100.000

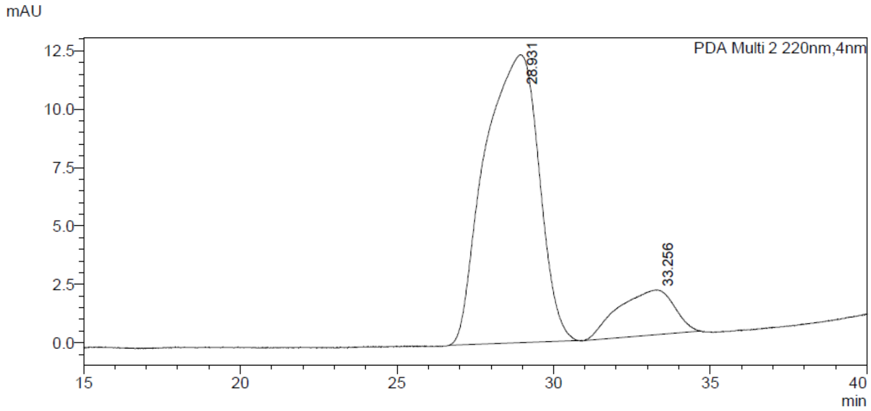

<Peak Table>

PDA Ch2 220nm

Peak#	Ret. Time	Area%
1	28.931	86.055
2	33.256	13.945
Total		100.000

HPLC Data for **S7**: Chiral HPLC analysis Chiralpak AD-H (99.8:0.2 hexane : IPA, flow rate 1.0 mL min^{-1}, 211 nm, 30 °C) t_R (*R*): 4.9 min, t_R (*S*): 5.2 min, 75% *ee*.

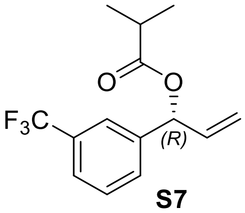

S7

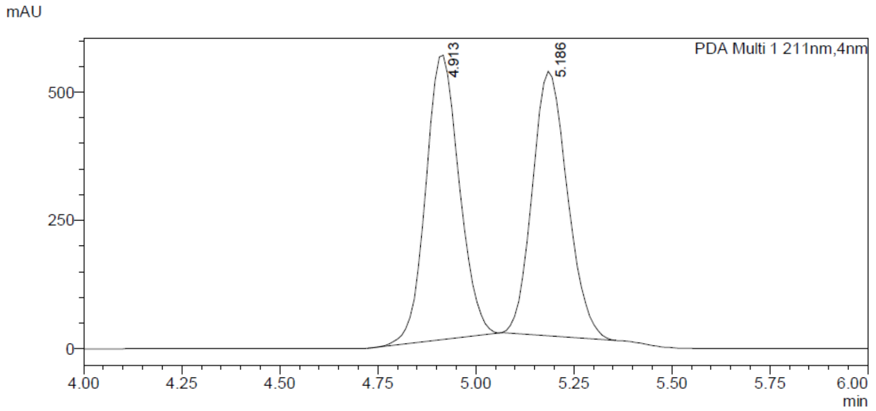

<Peak Table>

PDA Ch1 211nm

Peak#	Ret. Time	Area%
1	4.913	50.759
2	5.186	49.241
Total		100.000

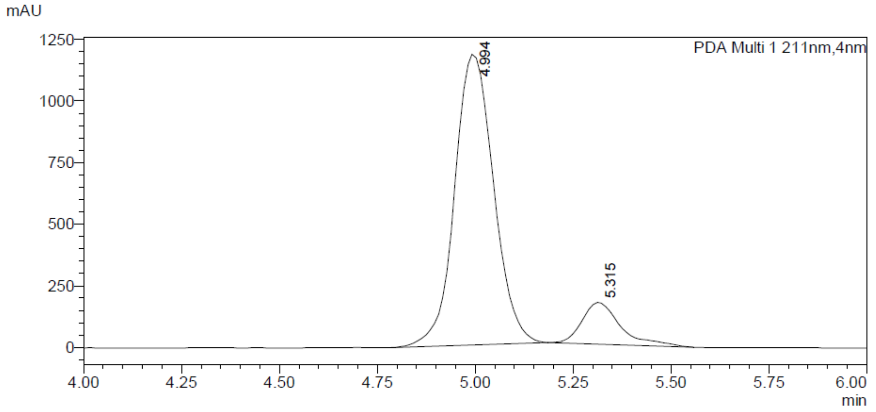

<Peak Table>

PDA Ch1 211nm

Peak#	Ret. Time	Area%
1	4.994	87.631
2	5.315	12.369
Total		100.000

HPLC Data for **S8**: Chiral HPLC analysis Chiralpak AD-H (99.8:0.2 hexane : IPA, flow rate 1.0 mL min^{-1}, 211 nm, 30 °C) t_R (*R*): 5.3 min, t_R (*S*): 5.7 min, 67 % *ee*.

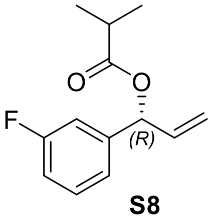

S8

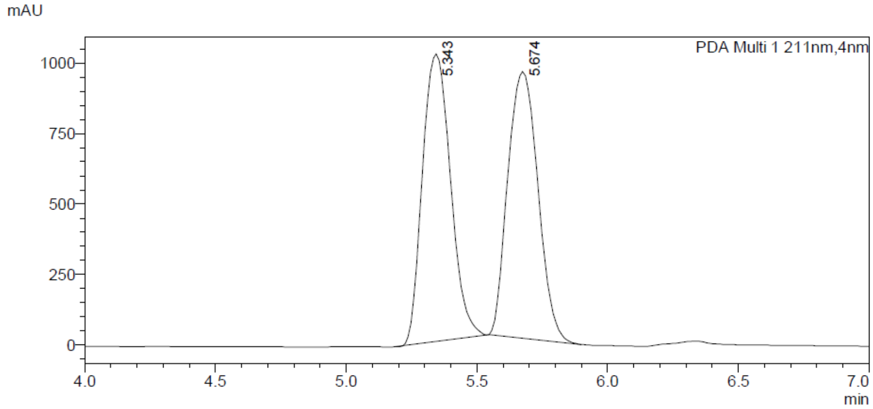

<Peak Table>

PDA Ch1 211nm

Peak#	Ret. Time	Area%
1	5.343	50.248
2	5.674	49.752
Total		100.000

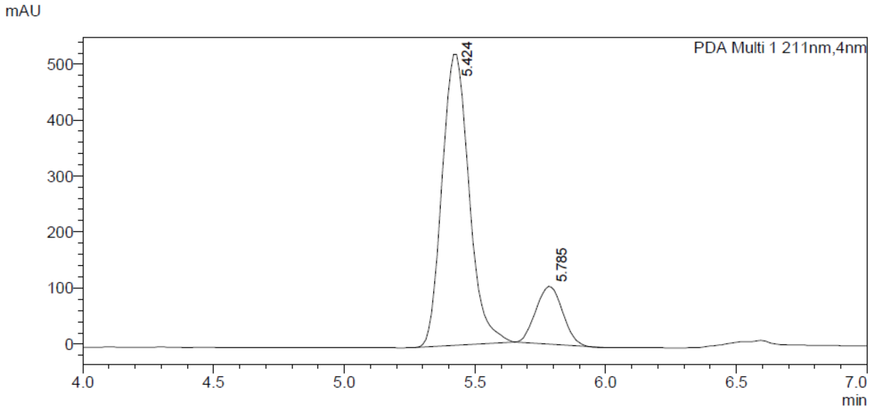

<Peak Table>

PDA Ch1 211nm

Peak#	Ret. Time	Area%
1	5.424	83.691
2	5.785	16.309
Total		100.000

HPLC Data for **26**: Chiral HPLC analysis Chiralcel OD-H (95:5 hexane : IPA, flow rate 0.5 mL min^{-1}, 270 nm, 30 °C) t_R (*R*): 28.808 min, t_R (*S*): 21.9 min, 90 % *ee*.

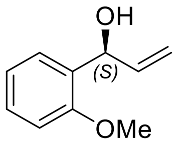

26

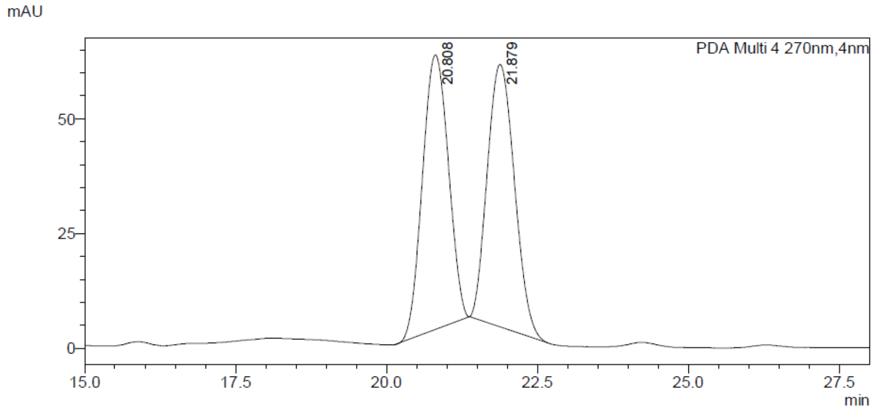

<Peak Table>

PDA Ch4 270nm

Peak#	Ret. Time	Area%
1	20.808	49.966
2	21.879	50.034
Total		100.000

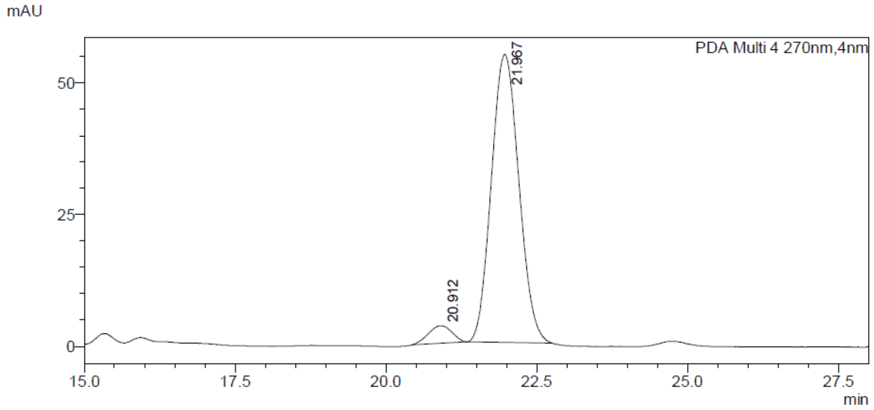

<Peak Table>

PDA Ch4 270nm

Peak#	Ret. Time	Area%
1	20.912	4.985
2	21.967	95.015
Total		100.000

HPLC Data for **27**: Chiral HPLC analysis Chiralcel OJ-H (95:5 hexane : IPA, flow rate 1.0 mL min^{-1}, 220 nm, 30 °C) t_R (*S*): 6.3 min, t_R (*R*): 7.4 min, 37% *ee*.

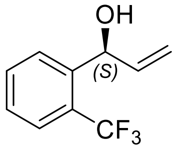

27

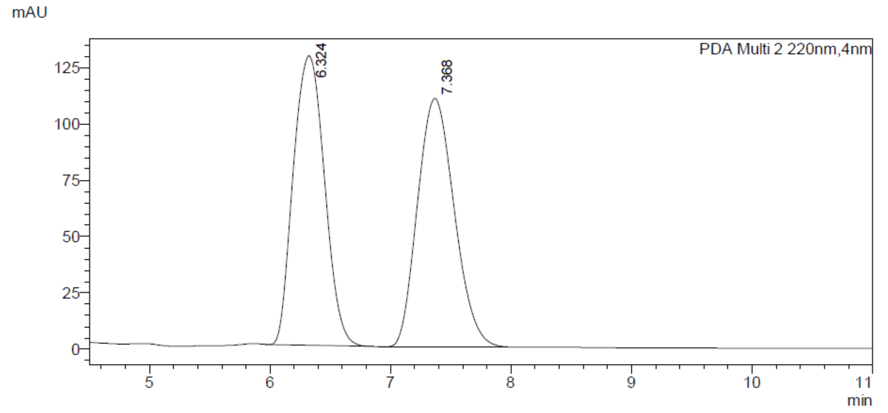

<Peak Table>

PDA Ch2 220nm

Peak#	Ret. Time	Area%
1	6.324	49.861
2	7.368	50.139
Total		100.000

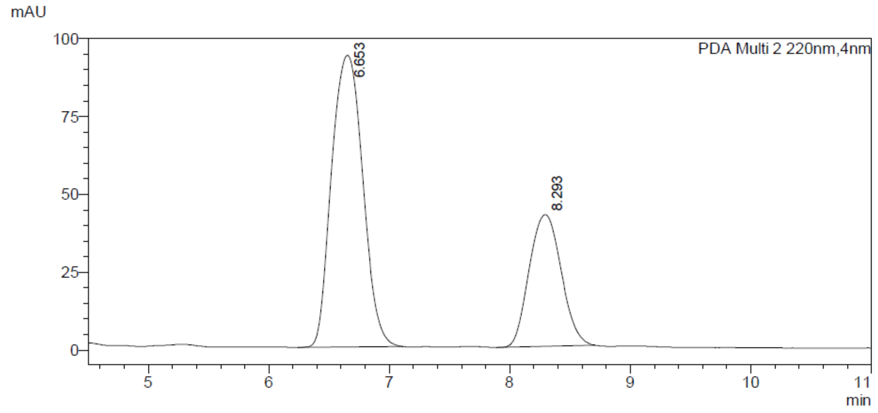

<Peak Table>

PDA Ch2 220nm

Peak#	Ret. Time	Area%
1	6.653	68.303
2	8.293	31.697
Total		100.000

HPLC Data for **S10**: Chiral HPLC analysis Chiralpak AD-H (99.8:0.2 hexane : IPA, flow rate 1.0 mL min^{-1}, 211 nm, 30 °C) t_R (*R*): 4.2 min, t_R (*S*): 4.5 min, 64% *ee*.

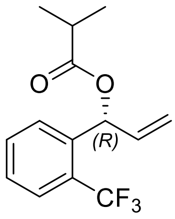

S10

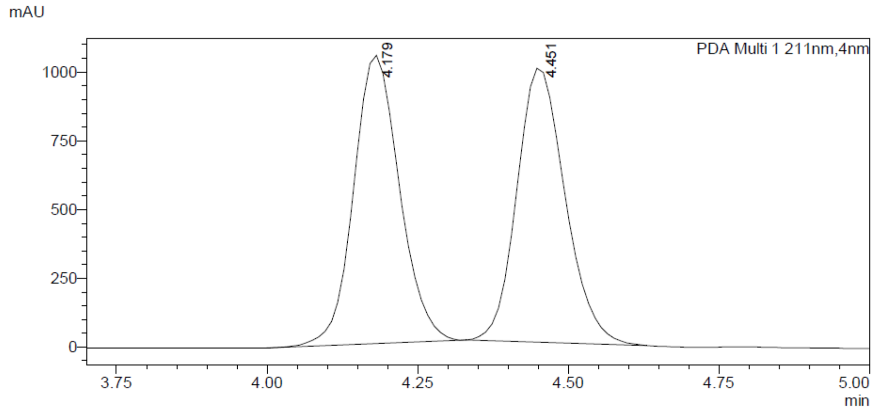

<Peak Table>

PDA Ch1 211nm

Peak#	Ret. Time	Area%
1	4.179	49.999
2	4.451	50.001
Total		100.000

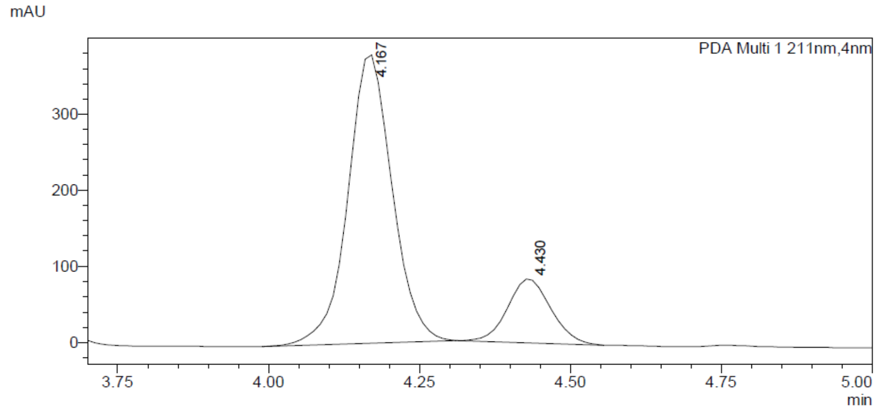

<Peak Table>

PDA Ch1 211nm

Peak#	Ret. Time	Area%
1	4.167	82.264
2	4.430	17.736
Total		100.000

HPLC Data for **28**: Chiral HPLC analysis Chiralcel OD-H (95:5 hexane : IPA, flow rate 1.0 mL min^{-1}, 211 nm, 30 °C) t_R (*R*): 14.2 min, t_R (*S*): 24.9 min, 56% *ee*.

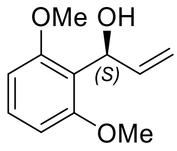

28

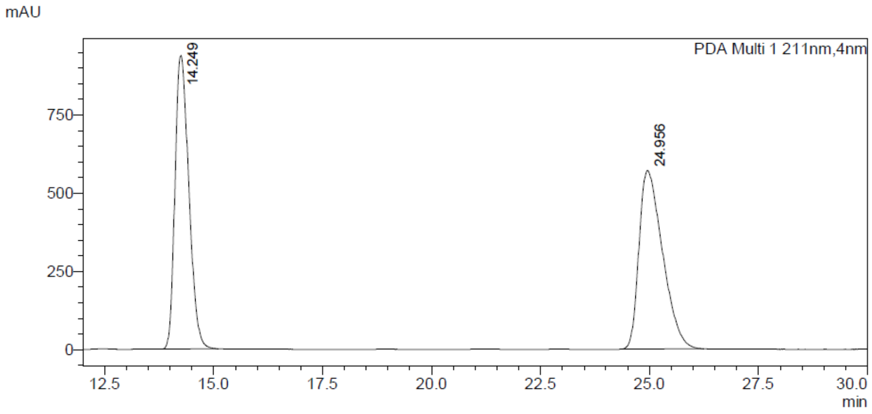

<Peak Table>

PDA Ch1 211nm

Peak#	Ret. Time	Area%
1	14.249	49.161
2	24.956	50.839
Total		100.000

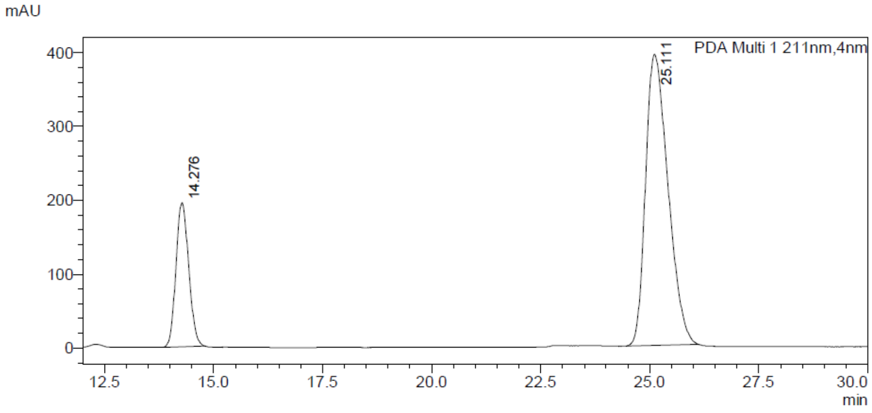

<Peak Table>

PDA Ch1 211nm

Peak#	Ret. Time	Area%
1	14.276	21.955
2	25.111	78.045
Total		100.000

HPLC Data for **S11**: Chiral HPLC analysis Chiralcel OJ-H (99:1 hexane : IPA, flow rate 1.0 mL min^{-1}, 211 nm, 30 °C) t_R (*S*): 6.9 min, t_R (*R*): 8.5 min, 97 % *ee*.

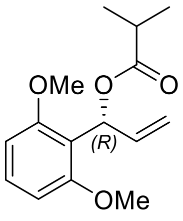

S11

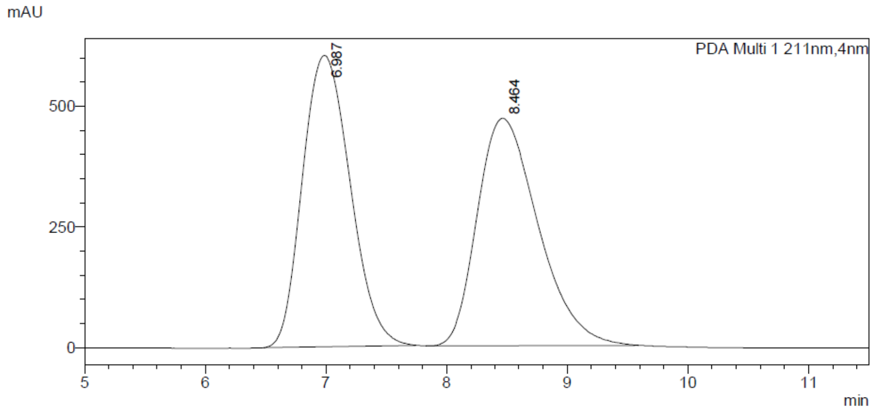

<Peak Table>

PDA Ch1 211nm

Peak#	Ret. Time	Area%
1	6.987	50.150
2	8.464	49.850
Total		100.000

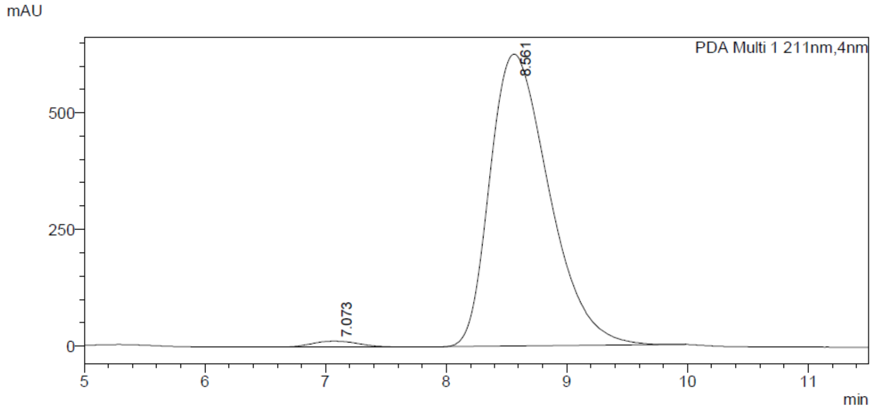

<Peak Table>

PDA Ch1 211nm

Peak#	Ret. Time	Area%
1	7.073	1.575
2	8.561	98.425
Total		100.000

HPLC Data for **29**: Chiral HPLC analysis Chiralcel OJ-H (92:8 hexane : IPA, flow rate 1.0 mL min^{-1}, 220 nm, 30 °C) t_R : 32.1 min, t_R : 35.2 min, >99% *ee*.

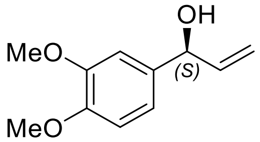

29

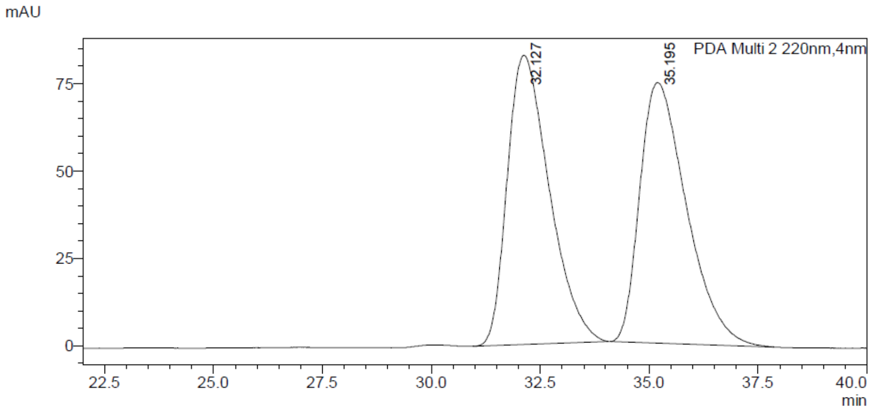

<Peak Table>

PDA Ch2 220nm

Peak#	Ret. Time	Area%
1	32.127	50.024
2	35.195	49.976
Total		100.000

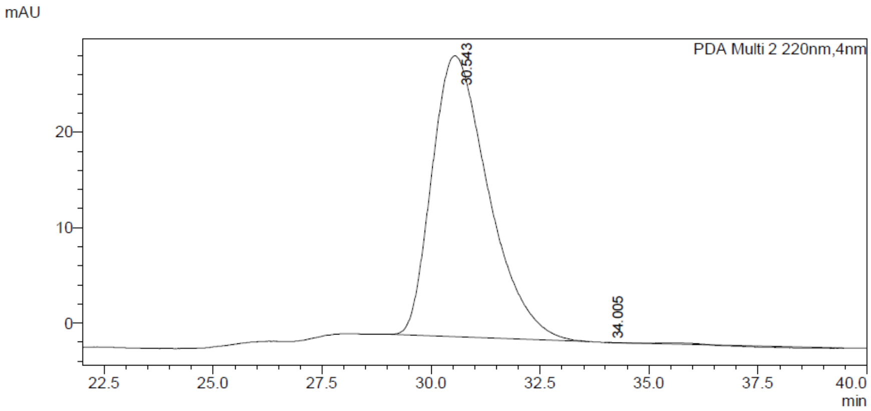

<Peak Table>

PDA Ch2 220nm

Peak#	Ret. Time	Area%
1	30.543	100.277
2	34.005	-0.277
Total		100.000

HPLC Data for **S14**: Chiral HPLC analysis Chiralcel OJ-H (99:1 hexane : IPA, flow rate 1.0 mL min^{-1}, 254 nm, 30 °C) t_R : 15.4 min, t_R : 18.3 min, 60% *ee*.

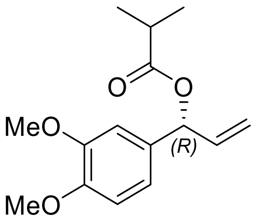

S14

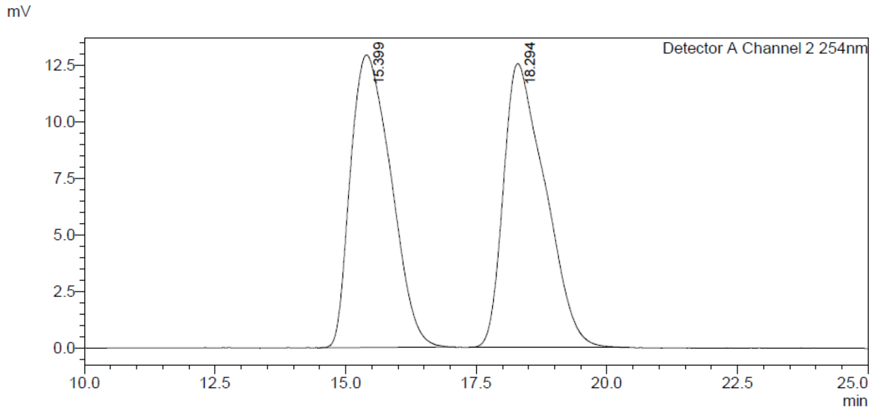

<Peak Table>

Detector A Channel 2 254nm

Peak#	Ret. Time	Area%
1	15.399	49.980
2	18.294	50.020
Total		100.000

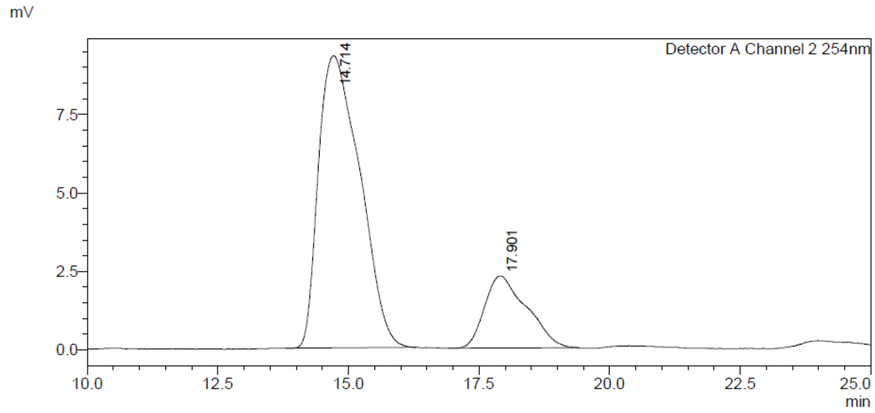

<Peak Table>

Detector A Channel 2 254nm

Peak#	Ret. Time	Area%
1	14.714	80.037
2	17.901	19.963
Total		100.000

HPLC Data for **30**: Chiral HPLC analysis Chiralpak AD-H (95:5 hexane : IPA, flow rate 1.0 mL min^{-1}, 211 nm, 30 °C) t_R (*S*): 22.2 min, t_R (*R*): 27.0 min, 87% *ee*.

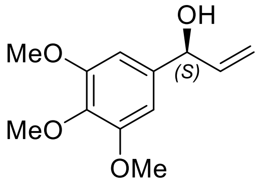

30

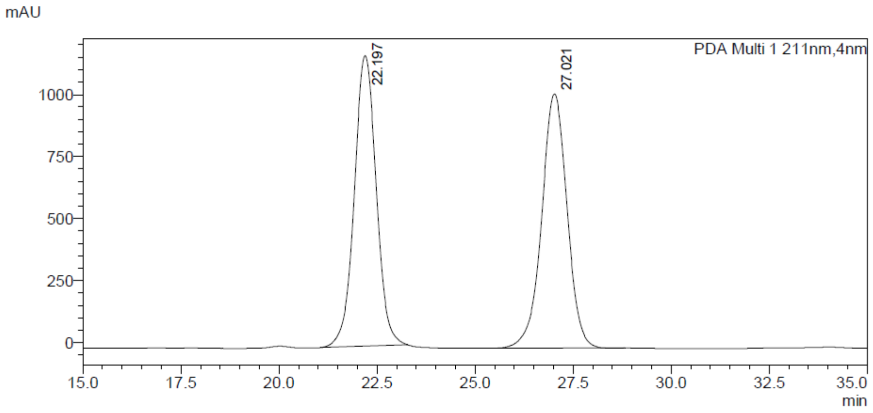

<Peak Table>

PDA Ch1 211nm

Peak#	Ret. Time	Area%
1	22.197	49.660
2	27.021	50.340
Total		100.000

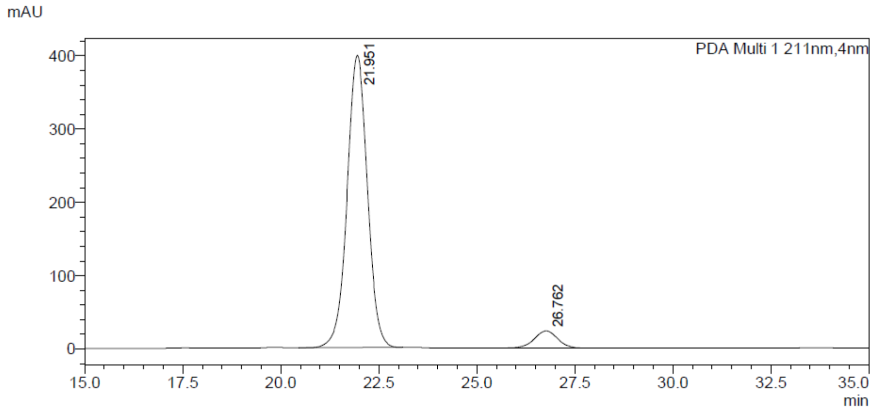

<Peak Table>

PDA Ch1 211nm

Peak#	Ret. Time	Area%
1	21.951	93.619
2	26.762	6.381
Total		100.000

HPLC Data for **S17**: Chiral HPLC analysis Chiralpak AD-H (99:1 hexane : IPA, flow rate 1.0 mL min^{-1}, 254 nm, 30 °C) t_R (*R*): 20.7 min, t_R (*S*): 29.9 min, 84% *ee*.

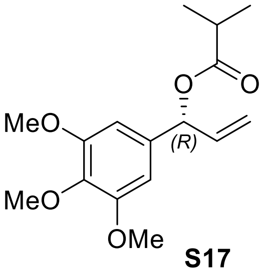

S17

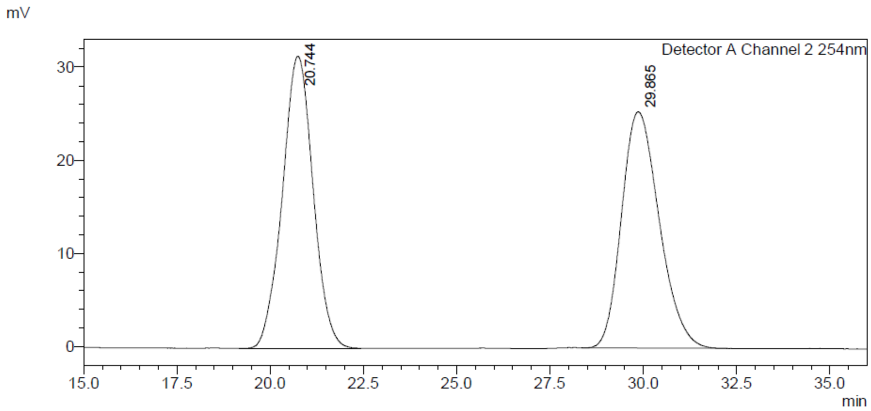

<Peak Table>

Detector A Channel 2 254nm

Peak#	Ret. Time	Area%
1	20.744	50.193
2	29.865	49.807
Total		100.000

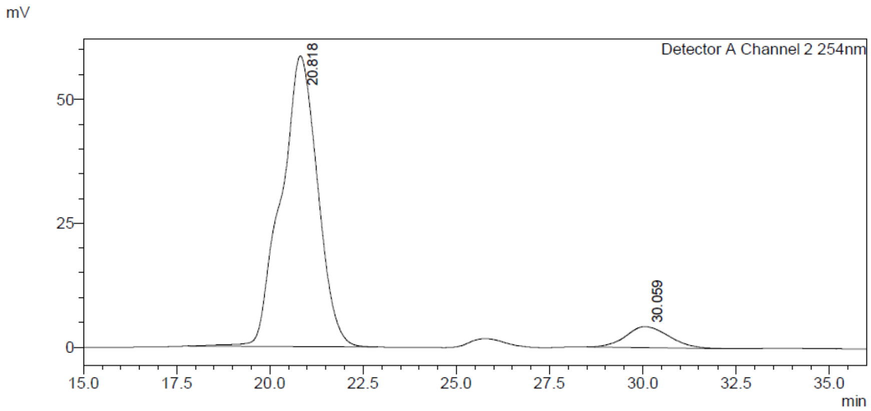

<Peak Table>

Detector A Channel 2 254nm

Peak#	Ret. Time	Area%
1	20.818	92.179
2	30.059	7.821
Total		100.000

HPLC Data for **31**: Chiral HPLC analysis Chiralcel OD-H (99:1 hexane : IPA, flow rate 1.0 mL min^{-1}, 211 nm, 30 °C) t_R (*R*): 16.1 min, t_R (*S*): 18.8 min, 22% *ee*.

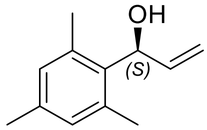

31

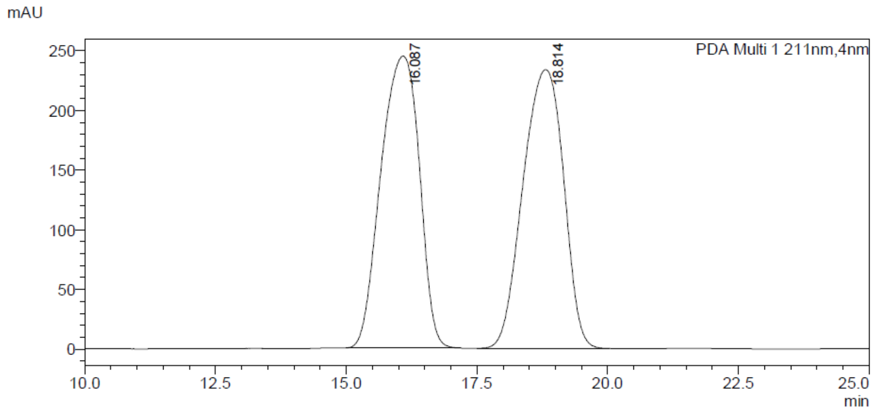

<Peak Table>

PDA Ch1 211nm

Peak#	Ret. Time	Area%
1	16.087	49.775
2	18.814	50.225
Total		100.000

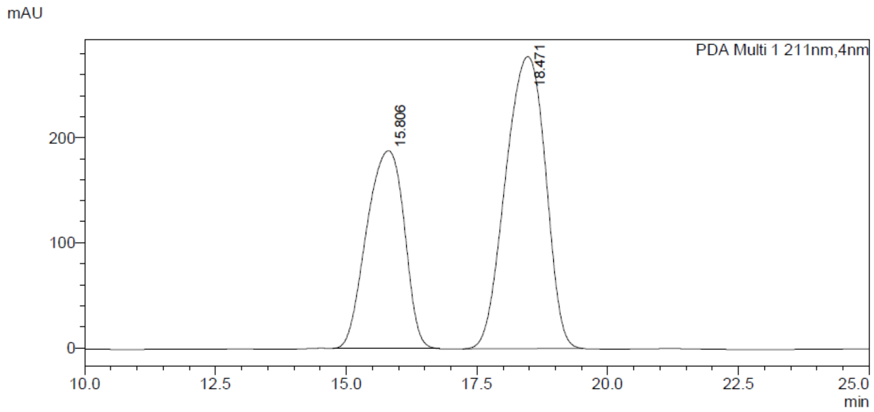

<Peak Table>

PDA Ch1 211nm

Peak#	Ret. Time	Area%
1	15.806	38.982
2	18.471	61.018
Total		100.000

HPLC Data for **S18**: Chiral HPLC analysis Chiralpak AD-H (99.5:0.5 hexane : IPA, flow rate 1.0 mL min^{-1}, 220 nm, 30 °C) t_R (*R*): 4.1 min, t_R (*S*): 4.5 min, 79 % *ee*.

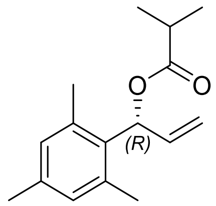

S18

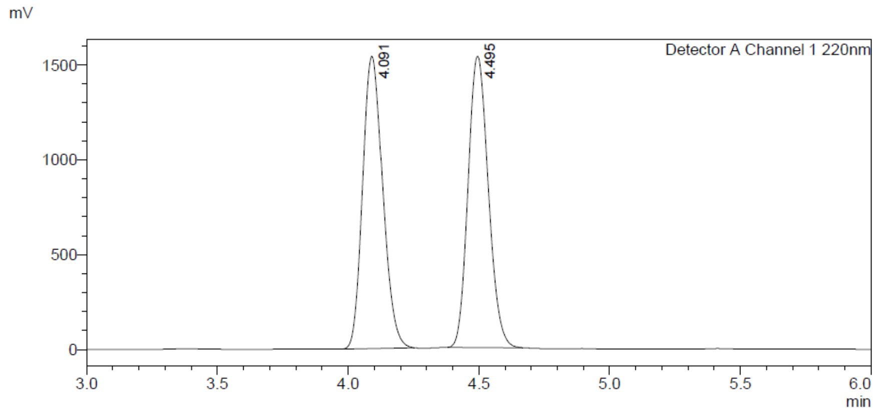

<Peak Table>

Detector A Channel 1 220nm

Peak#	Ret. Time	Area%
1	4.091	50.091
2	4.495	49.909
Total		100.000

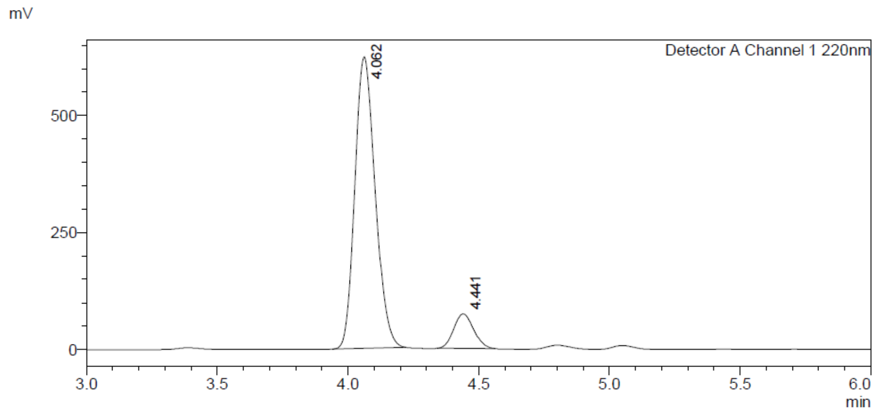

<Peak Table>

Detector A Channel 1 220nm

Peak#	Ret. Time	Area%
1	4.062	89.659
2	4.441	10.341
Total		100.000

HPLC Data for **32**: Chiral HPLC analysis Chiralcel OJ-H (80:20 hexane : IPA, flow rate 1.0 mL min^{-1}, 254 nm, 30 °C) t_R (*S*): 11.7 min, t_R (*R*): 14.1 min, 94% *ee*.

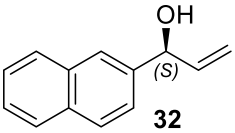

32

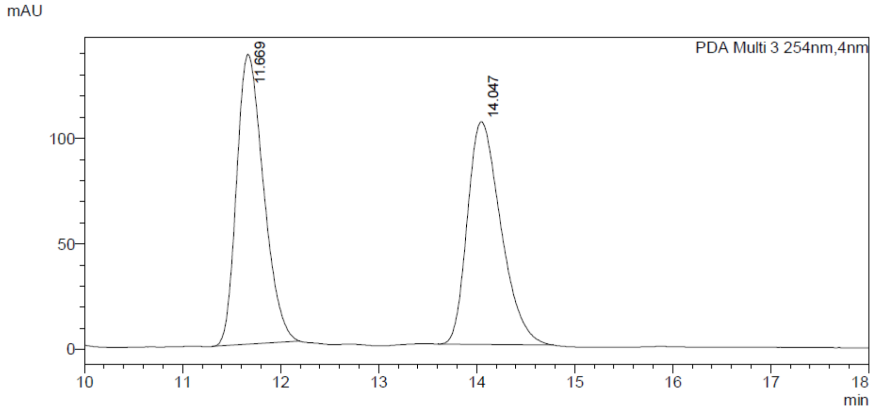

<Peak Table>

PDA Ch3 254nm

Peak#	Ret. Time	Area%
1	11.669	51.168
2	14.047	48.832
Total		100.000

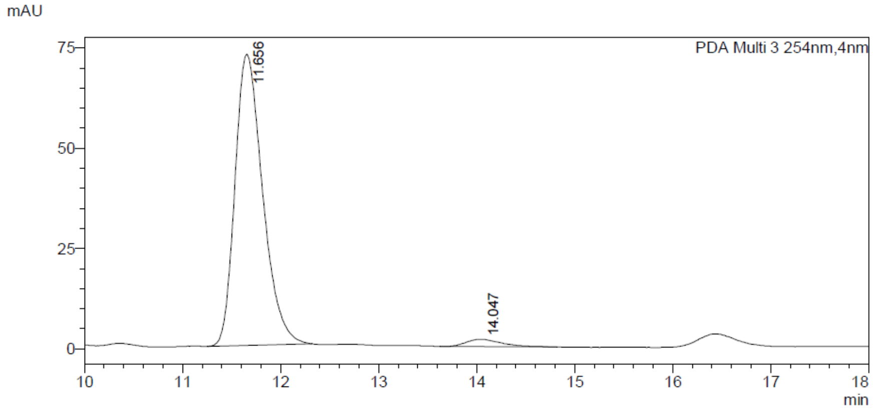

<Peak Table>

PDA Ch3 254nm

Peak#	Ret. Time	Area%
1	11.656	96.966
2	14.047	3.034
Total		100.000

HPLC Data for **37**: Chiral HPLC analysis Chiralcel OJ-H (95:5 hexane : IPA, flow rate 0.5 mL min^{-1}, 270 nm, 30 °C) t_R (*S*): 13.4 min, t_R (*R*): 15.8 min, >99 % *ee*.

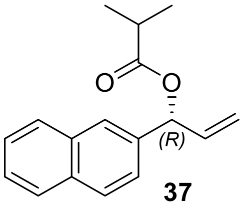

37

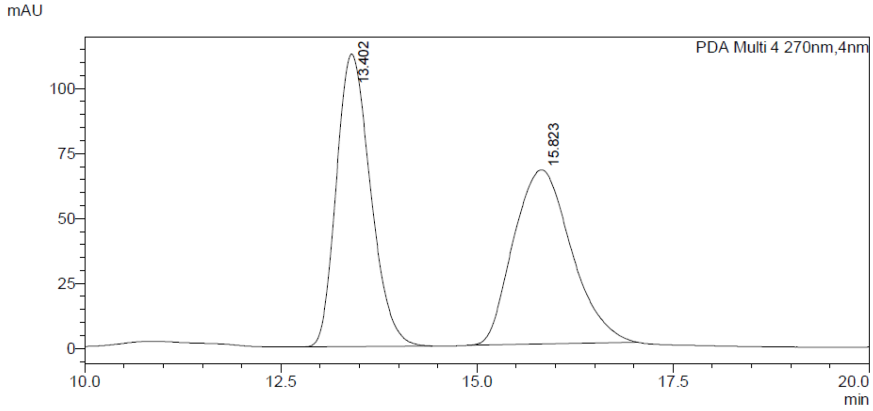

<Peak Table>

PDA Ch4 270nm

Peak#	Ret. Time	Area%
1	13.402	49.722
2	15.823	50.278
Total		100.000

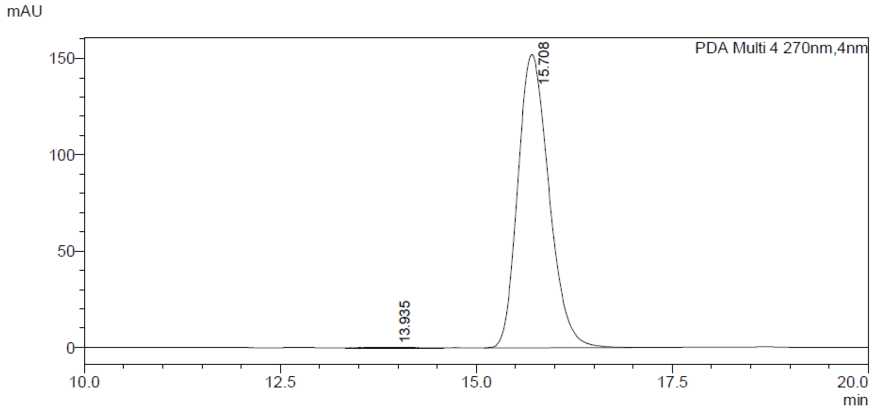

<Peak Table>

PDA Ch4 270nm

Peak#	Ret. Time	Area%
1	13.935	0.121
2	15.708	99.879
Total		100.000

HPLC Data for **33**: Chiral HPLC analysis Chiralcel OJ-H (80:20 hexane : IPA, flow rate 1.0 mL min^{-1}, 211 nm, 30 °C) t_R (*S*): 9.2 min, t_R (*R*): 11.8 min, 84% *ee*.

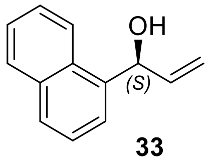

33

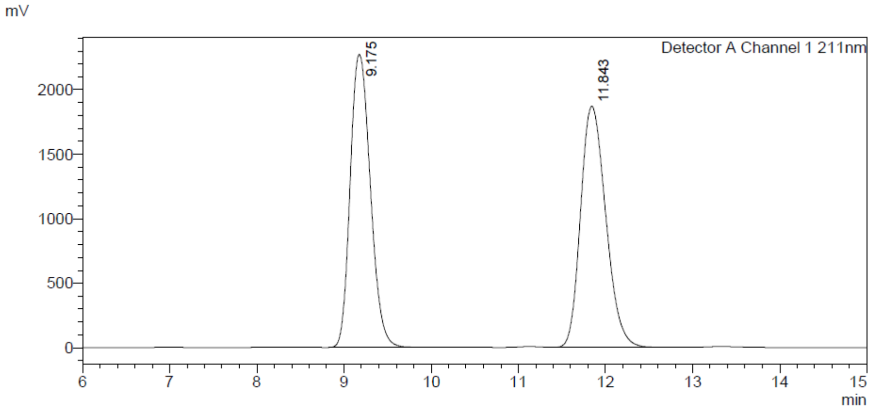

<Peak Table>

Detector A Channel 1 211nm

Peak#	Ret. Time	Area%
1	9.175	49.297
2	11.843	50.703
Total		100.000

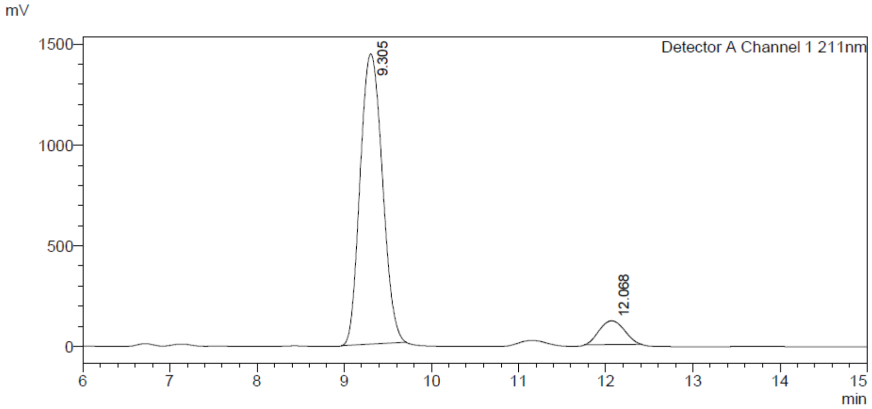

<Peak Table>

Detector A Channel 1 211nm

Peak#	Ret. Time	Area%
1	9.305	91.458
2	12.068	8.542
Total		100.000

HPLC Data for **S19**: Chiral HPLC analysis Chiralcel OJ-H (95:5 hexane : IPA, flow rate 0.5 mL min^{-1}, 211 nm, 30 °C) t_R (*R*): 12.3 min, t_R (*S*): 13.5 min, 96% *ee*.

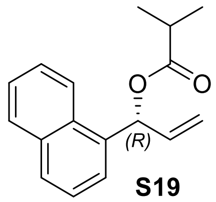

S19

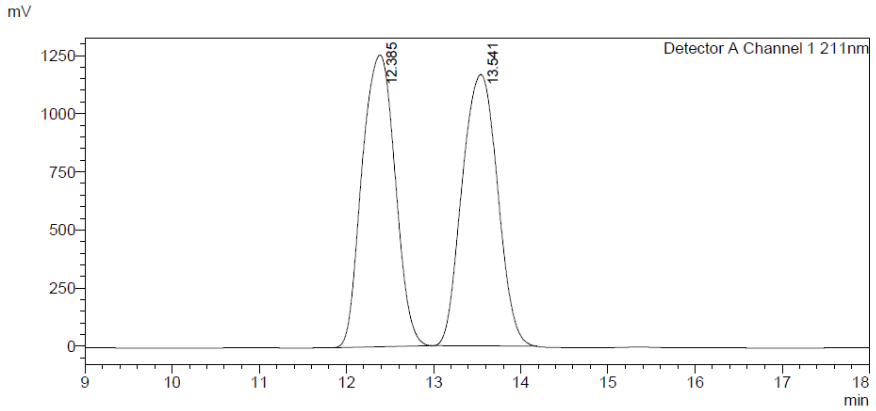

<Peak Table>

Detector A Channel 1 211nm

Peak#	Ret. Time	Area%
1	12.385	50.066
2	13.541	49.934
Total		100.000

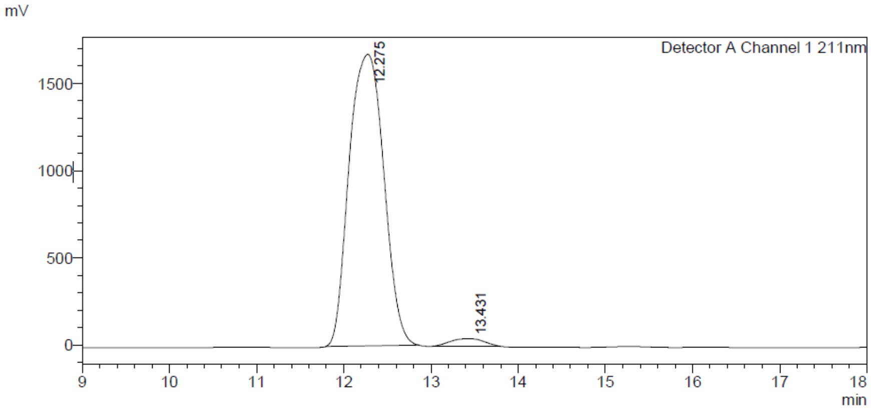

<Peak Table>

Detector A Channel 1 211nm

Peak#	Ret. Time	Area%
1	12.275	97.640
2	13.431	2.360
Total		100.000

HPLC Data for **34**: Chiral HPLC analysis Chiralcel OJ-H (80:20 hexane : IPA, flow rate 1.0 mL min^{-1}, 211 nm, 30 °C) t_R (*S*): 23.7 min, t_R (*R*): 36.9 min, 44% *ee*.

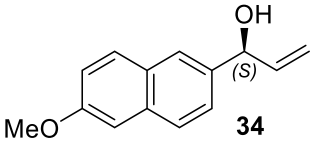

34

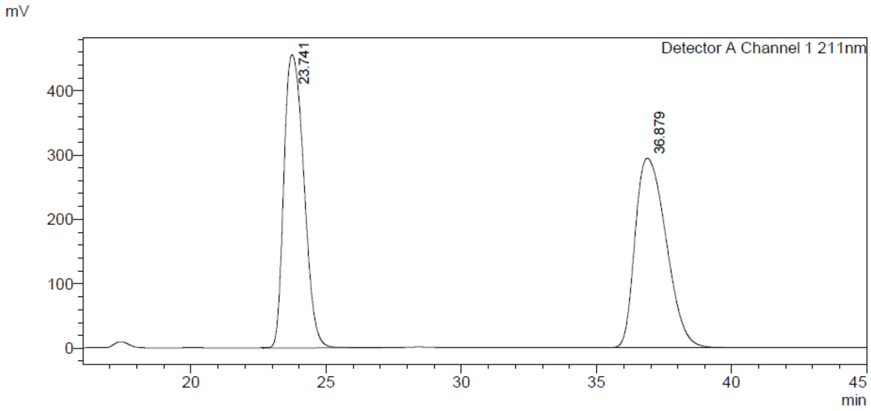

<Peak Table>

Detector A Channel 1 211nm

Peak#	Ret. Time	Area%
1	23.741	49.870
2	36.879	50.130
Total		100.000

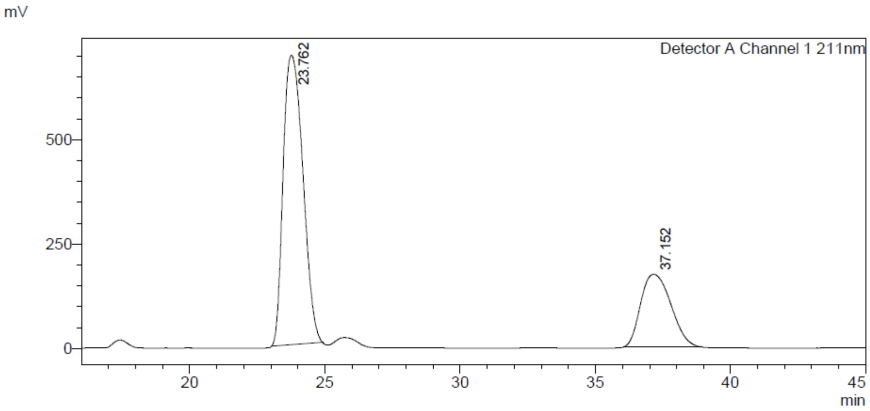

<Peak Table>

Detector A Channel 1 211nm

Peak#	Ret. Time	Area%
1	23.762	72.226
2	37.152	27.774
Total		100.000

HPLC Data for **S19**: Chiral HPLC analysis Chiralpak AD-H (99:1 hexane : IPA, flow rate 1.0 mL min^{-1}, 220 nm, 30 °C) t_R (*R*): 9.5 min, t_R (*S*): 12.6 min, 50% *ee*.

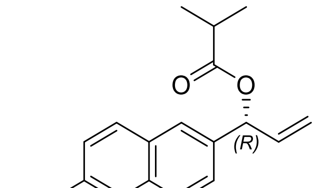

S19

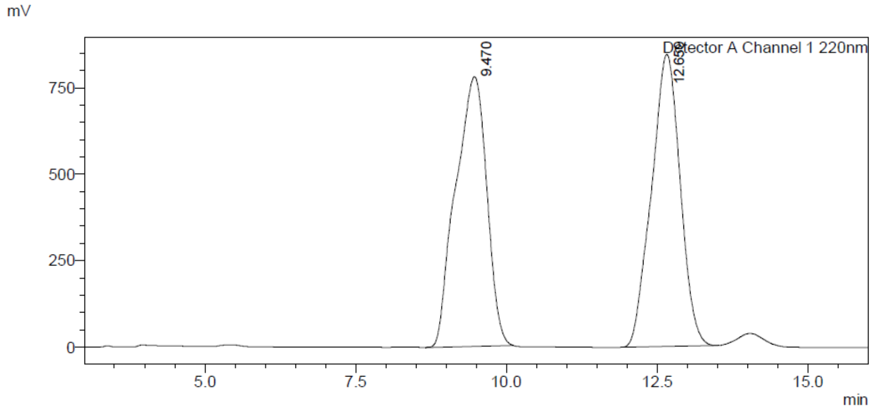

<Peak Table>

Detector A Channel 1 220nm

Peak#	Ret. Time	Area%
1	9.470	50.039
2	12.659	49.961
Total		100.000

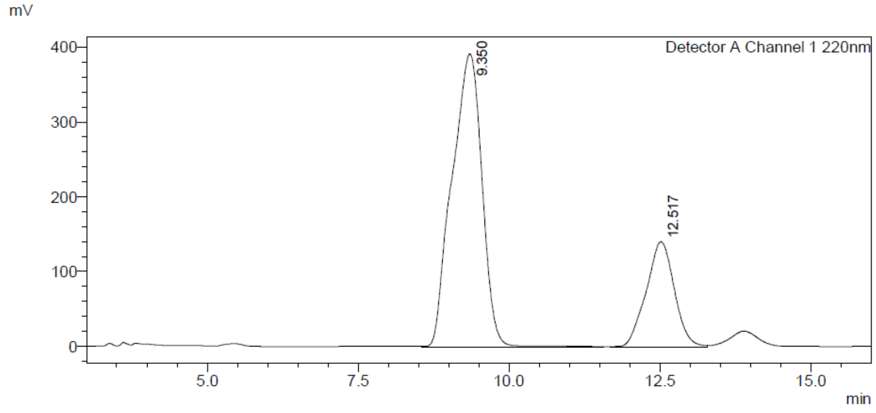

<Peak Table>

Detector A Channel 1 220nm

Peak#	Ret. Time	Area%
1	9.350	74.870
2	12.517	25.130
Total		100.000

HPLC Data for **35**: Chiral HPLC analysis Chiralcel OD-H (95:5 hexane : IPA, flow rate 1.0 mL min^{-1}, 254 nm, 30 °C) t_R (*R*): 13.6 min, t_R (*S*): 14.7 min, 56% *ee*.

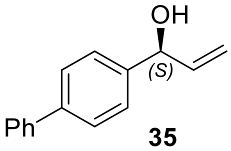

35

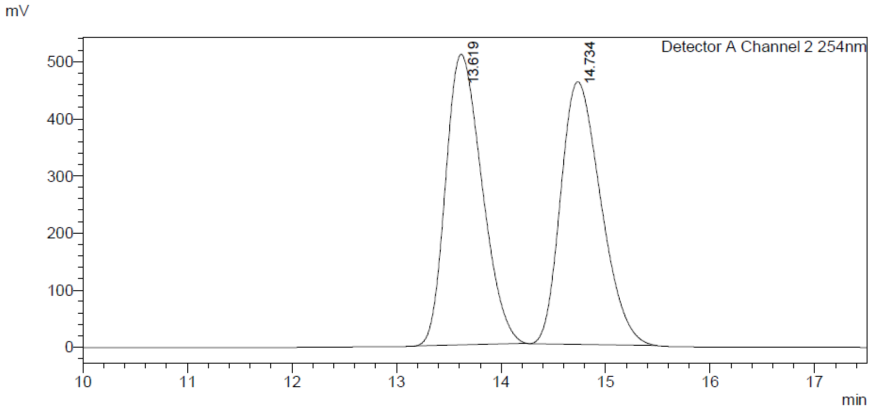

<Peak Table>

Detector A Channel 2 254nm

Peak#	Ret. Time	Area%
1	13.619	50.049
2	14.734	49.951
Total		100.000

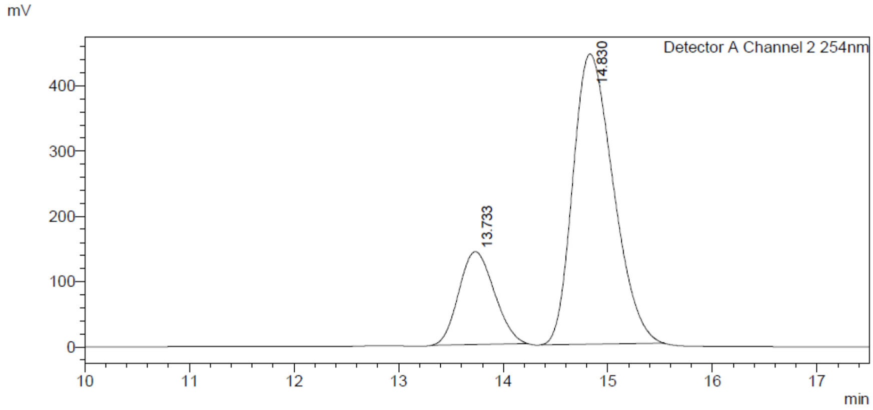

<Peak Table>

Detector A Channel 2 254nm

Peak#	Ret. Time	Area%
1	13.733	21.964
2	14.830	78.036
Total		100.000

HPLC Data for **S21**: Chiral HPLC analysis Chiralcel OD-H (99:1 hexane : IPA, flow rate 1.0 mL min^{-1}, 254 nm, 30 °C) t_R (*R*): 5.4 min, t_R (*S*): 6.3 min, 76 % *ee*.

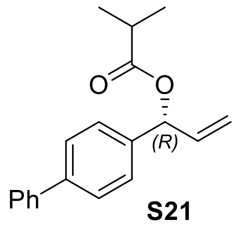

S21

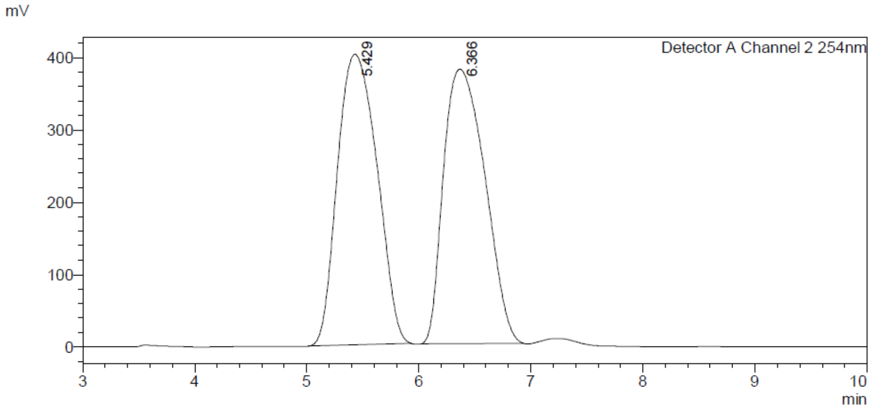

<Peak Table>

Detector A Channel 1 211nm

Peak#	Ret. Time	Area%
1	5.429	50.463
2	6.359	49.537
Total		100.000

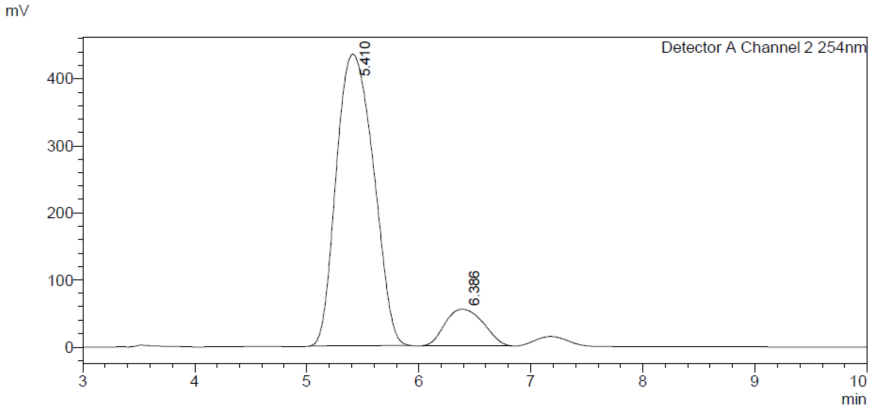

<Peak Table>

Detector A Channel 1 211nm

Peak#	Ret. Time	Area%
1	5.409	88.159
2	6.384	11.841
Total		100.000

HPLC Data for **36**: Chiral HPLC analysis Chiralcel OJ-H (80:20 hexane : IPA, flow rate 1.0 mL min^{-1}, 211 nm, 30 °C) t_R (*S*): 8.5 min, t_R (*R*): 9.6 min, 77% *ee*.

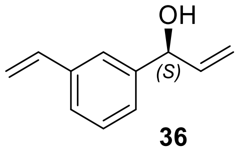

36

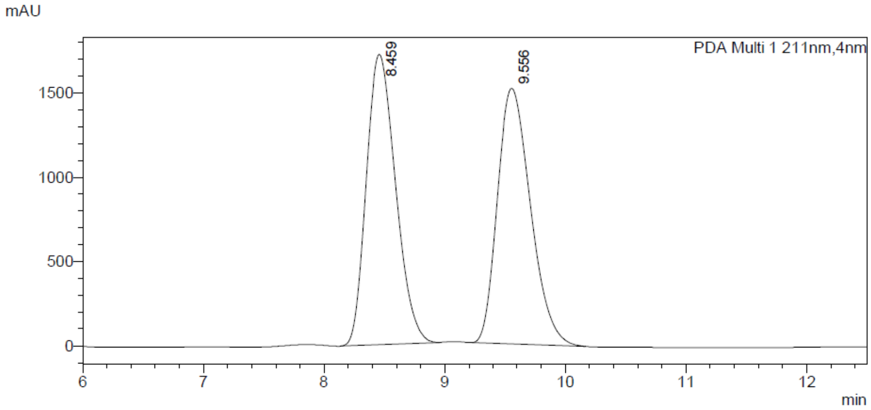

<Peak Table>

PDA Ch1 211nm

Peak#	Ret. Time	Area%
1	8.459	50.139
2	9.556	49.861
Total		100.000

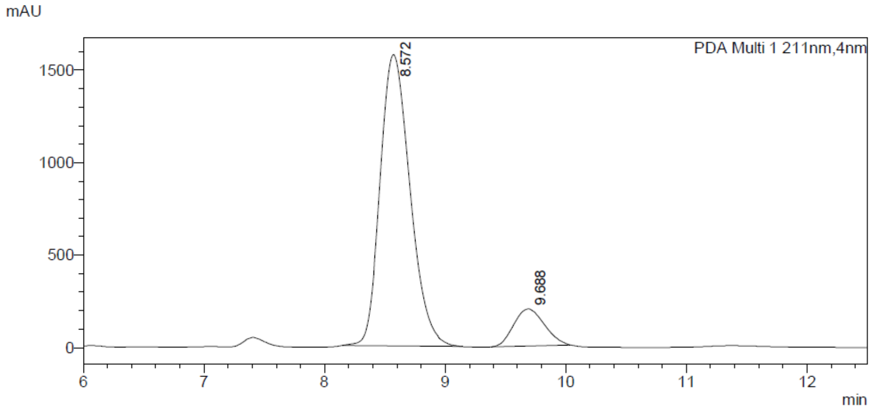

<Peak Table>

PDA Ch1 211nm

Peak#	Ret. Time	Area%
1	8.572	88.514
2	9.688	11.486
Total		100.000

HPLC Data for **S22**: Chiral HPLC analysis Chiralcel OJ-H (90:10 hexane : IPA, flow rate 1.0 mL min^{-1}, 211 nm, 30 °C) t_R (*R*): 4.4 min, t_R (*S*): 4.9 min, 83% *ee*.

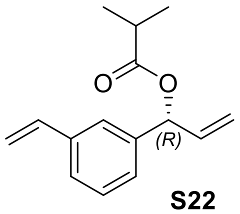

S22

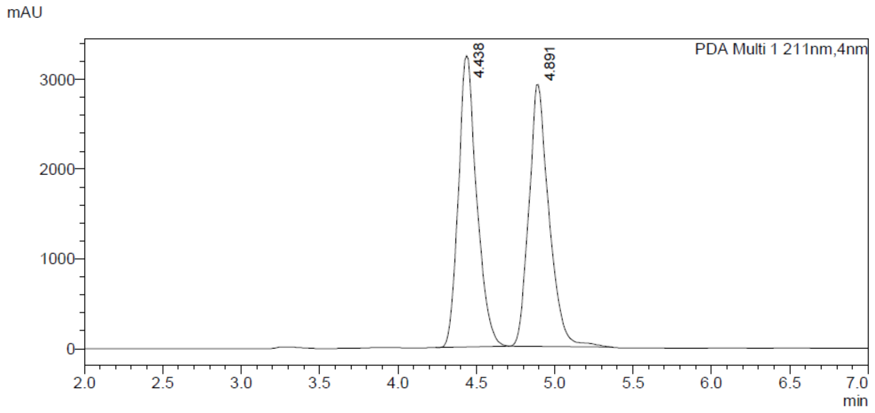

<Peak Table>

PDA Ch1 211nm

Peak#	Ret. Time	Area%
1	4.438	50.291
2	4.891	49.709
Total		100.000

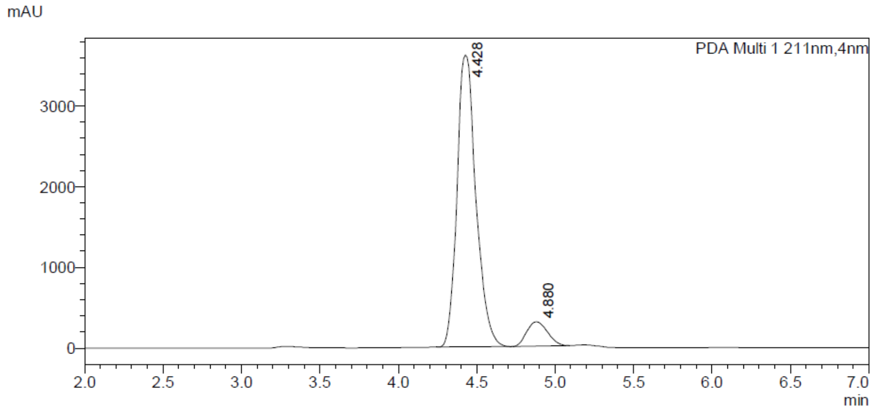

<Peak Table>

PDA Ch1 211nm

Peak#	Ret. Time	Area%
1	4.428	91.564
2	4.880	8.436
Total		100.000

HPLC Data for **38**: Chiral HPLC analysis Chiralcel OD-H (99:1 hexane : IPA, flow rate 1.0 mL min^{-1}, 254 nm, 30 °C) t_R (*R*): 17.4 min, t_R (*S*): 21.1 min, 34% *ee*.

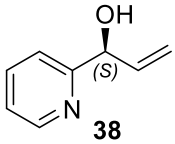

38

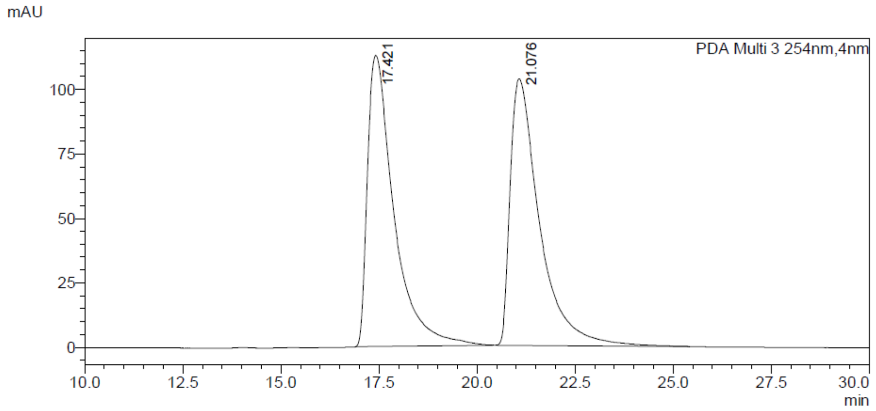

<Peak Table>

PDA Ch3 254nm

Peak#	Ret. Time	Area%
1	17.421	49.671
2	21.076	50.329
Total		100.000

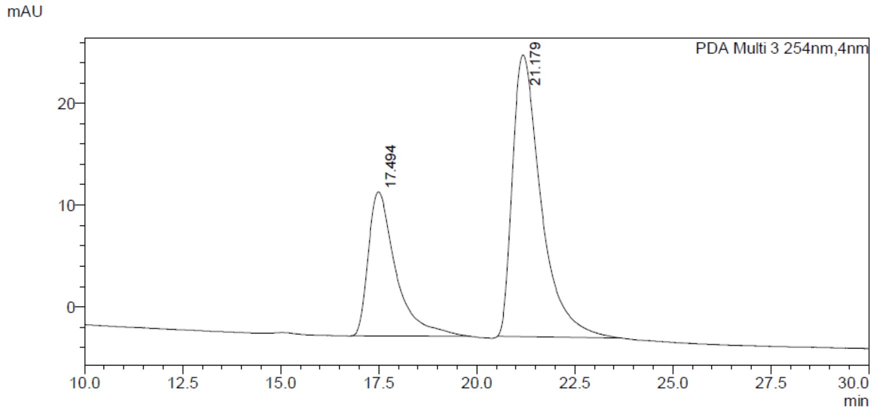

<Peak Table>

PDA Ch3 254nm

Peak#	Ret. Time	Area%
1	17.494	33.071
2	21.179	66.929
Total		100.000

HPLC Data for **S23**: Chiral HPLC analysis Chiralcel OD-H (99:1 hexane : IPA, flow rate 1.0 mL min^{-1}, 254 nm, 30 °C) t_R (*R*): 8.0 min, t_R (*S*): 9.4 min, 36% *ee*.

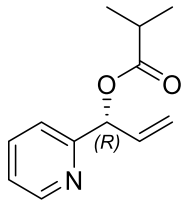

S23

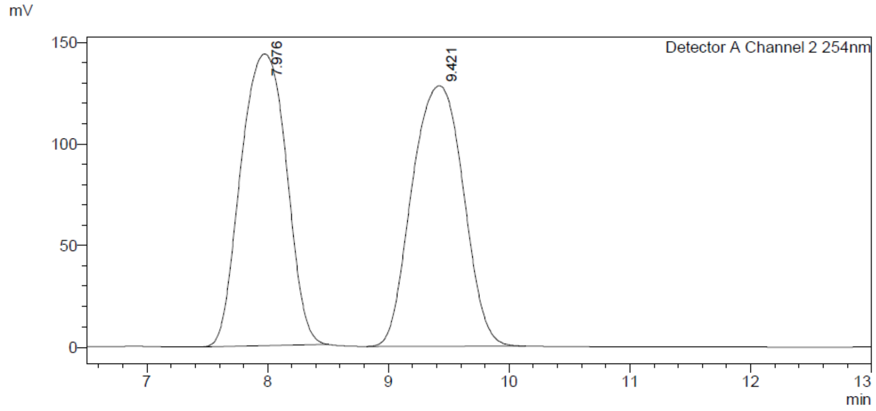

<Peak Table>

Detector A Channel 2 254nm

Peak#	Ret. Time	Area%
1	7.976	49.896
2	9.421	50.104
Total		100.000

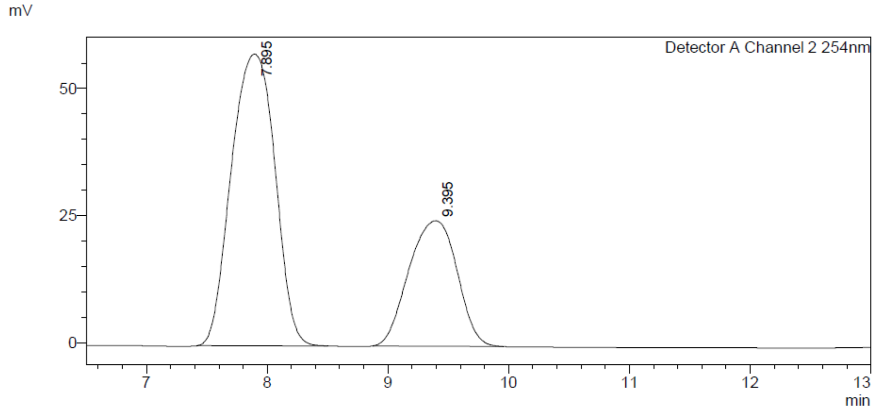

<Peak Table>

Detector A Channel 2 254nm

Peak#	Ret. Time	Area%
1	7.895	67.818
2	9.395	32.182
Total		100.000

HPLC Data for **39**: Chiral HPLC analysis Chiralcel OJ-H (90:10 hexane : IPA, flow rate 1.0 mL min^{-1}, 211 nm, 30 °C) t_R (*S*): 7.7 min, t_R (*R*): 9.1 min, 38% *ee*.

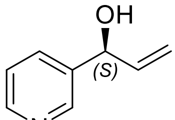

39

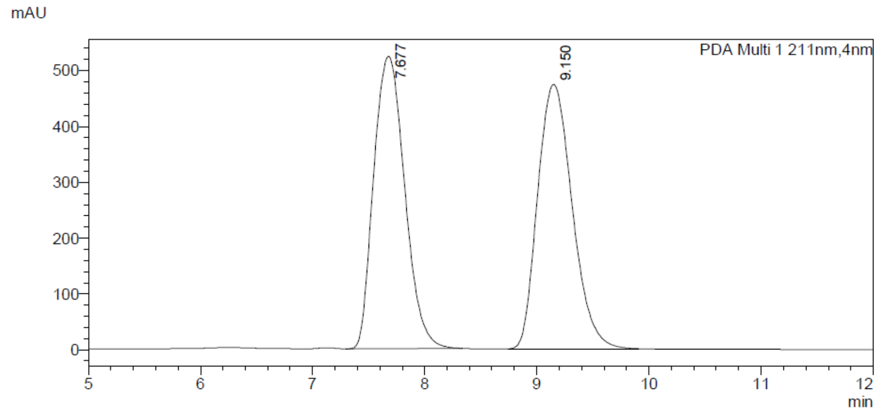

<Peak Table>

PDA Ch1 211nm

Peak#	Ret. Time	Area%
1	7.677	49.809
2	9.150	50.191
Total		100.000

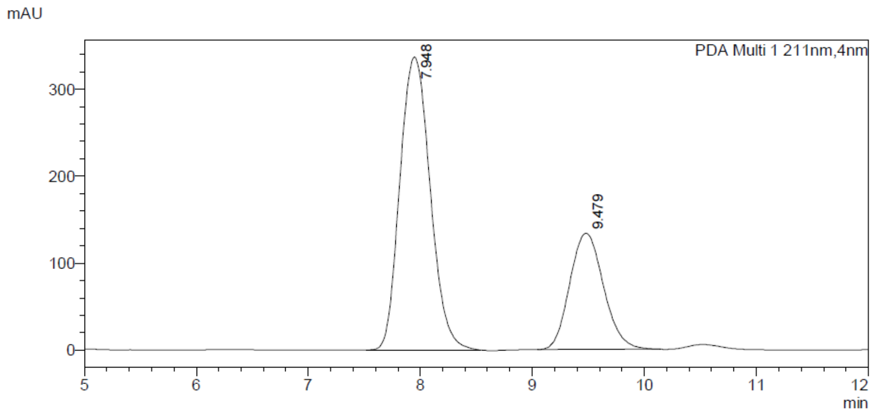

<Peak Table>

PDA Ch1 211nm

Peak#	Ret. Time	Area%
1	7.948	69.323
2	9.479	30.677
Total		100.000

HPLC Data for **S24**: Chiral HPLC analysis Chiralcel OJ-H (99:1 hexane : IPA, flow rate 1.0 mL min^{-1}, 254 nm, 30 °C) t_R (*R*): 8.4 min, t_R (*S*): 9.6 min, 46% *ee*.

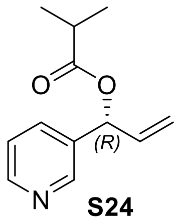

S24

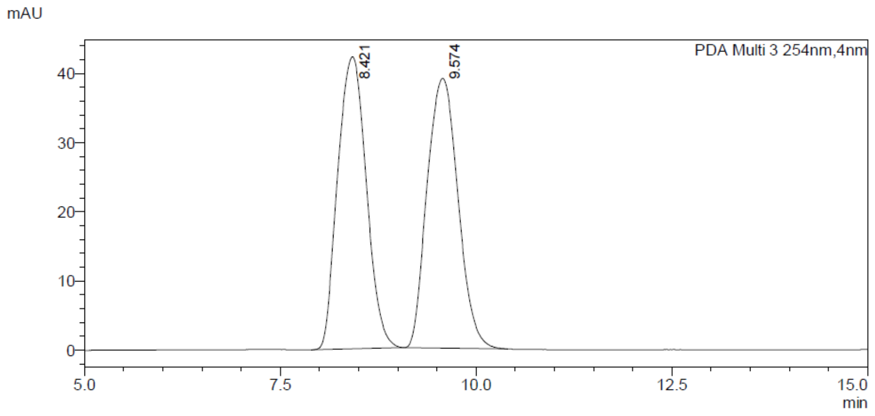

<Peak Table>

PDA Ch3 254nm

Peak#	Ret. Time	Area%
1	8.421	50.031
2	9.574	49.969
Total		100.000

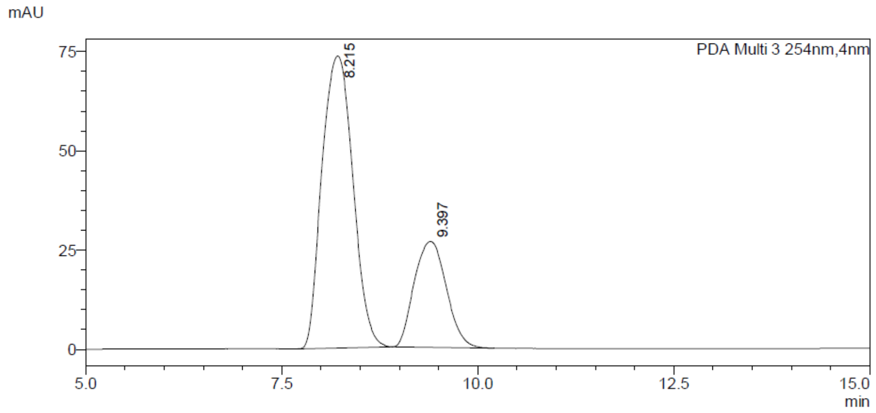

<Peak Table>

PDA Ch3 254nm

Peak#	Ret. Time	Area%
1	8.215	72.631
2	9.397	27.369
Total		100.000

HPLC Data for **40**: Chiral HPLC analysis Chiralcel OJ-H (99:1 hexane : IPA, flow rate 1.0 mL min^{-1}, 220 nm, 30 °C) t_R (*S*): 37.7 min, t_R (*R*): 44.8 min, 51% *ee*.

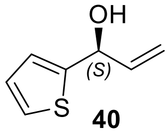

40

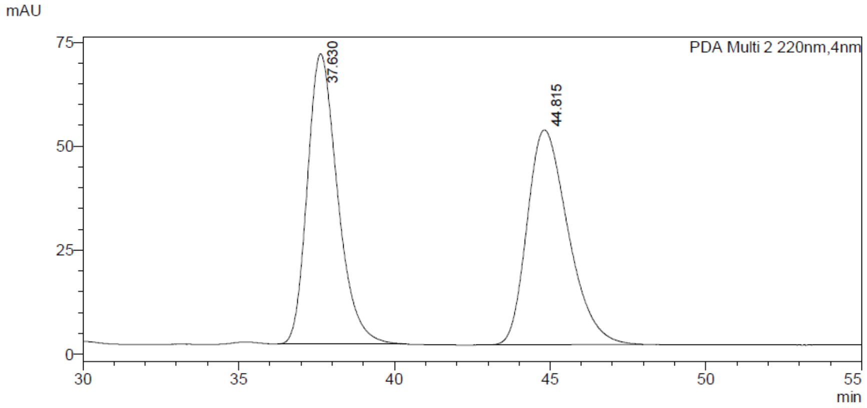

<Peak Table>

PDA Ch2 220nm

Peak#	Ret. Time	Area%
1	37.630	49.938
2	44.815	50.062
Total		100.000

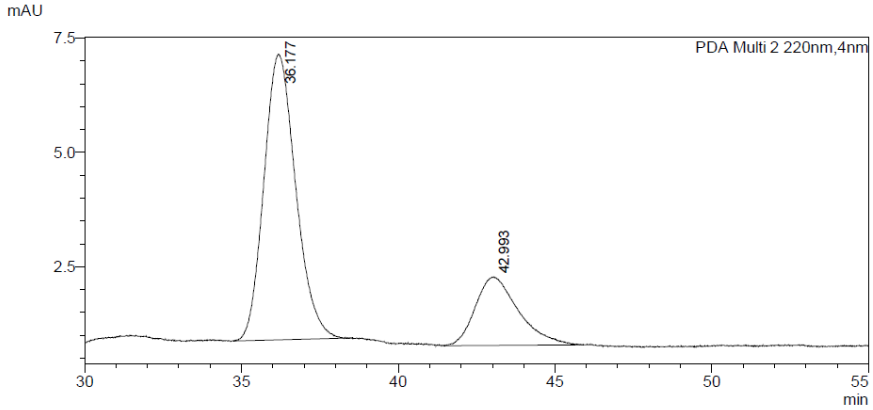

<Peak Table>

PDA Ch2 220nm

Peak#	Ret. Time	Area%
1	36.177	75.625
2	42.993	24.375
Total		100.000

HPLC Data for **S25**: Chiral HPLC analysis Chiralcel AD-H (99.8:0.2 hexane : IPA, flow rate 0.5 mL min^{-1}, 220 nm, 30 °C) t_R (*R*): 11.1 min, t_R (*S*): 12.1 min, 68% *ee*.

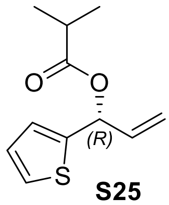

S25

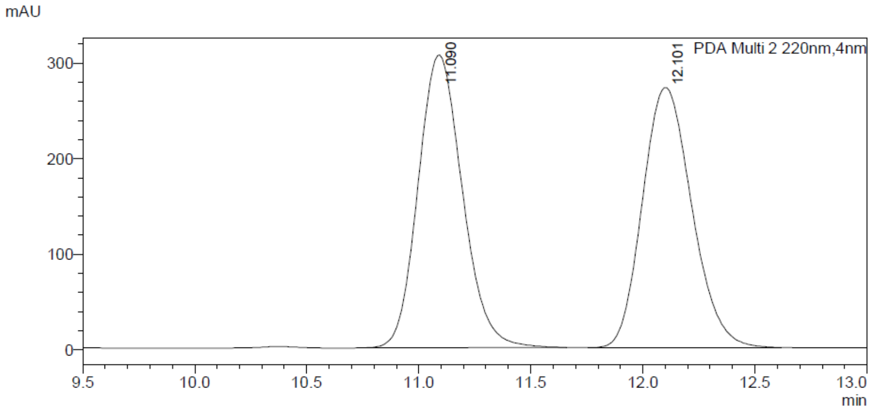

<Peak Table>

PDA Ch2 220nm

Peak#	Ret. Time	Area%
1	11.090	50.300
2	12.101	49.700
Total		100.000

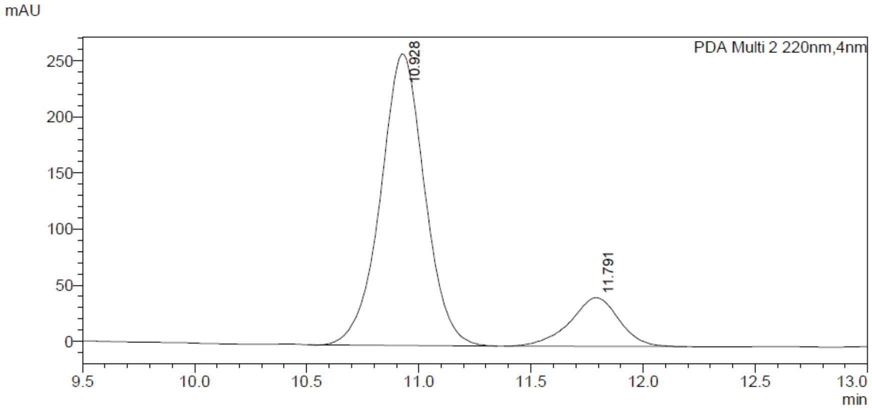

<Peak Table>

PDA Ch2 220nm

Peak#	Ret. Time	Area%
1	10.928	84.252
2	11.791	15.748
Total		100.000

HPLC Data for **41**: Chiral HPLC analysis Chiralcel OD-H (95:5 hexane : IPA, flow rate 1.0 mL min^{-1}, 211 nm, 30 °C) t_R (*R*): 10.3 min, t_R (*S*): 12.0 min, 58% *ee*.

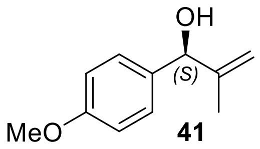

41

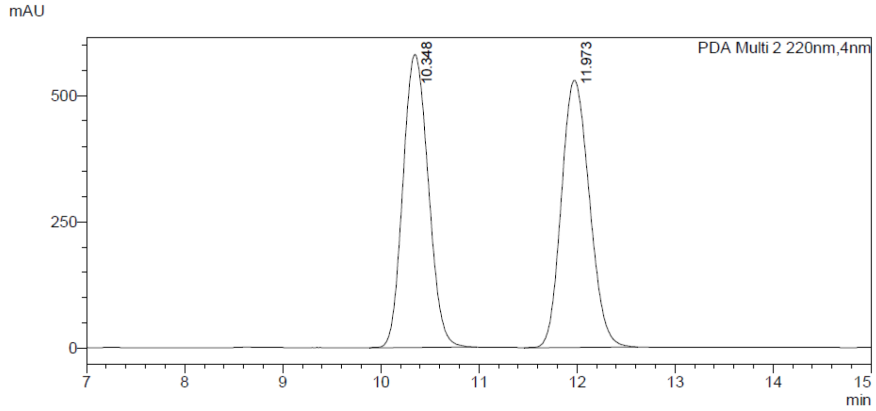

<Peak Table>

PDA Ch2 220nm

Peak#	Ret. Time	Area%
1	10.348	49.885
2	11.973	50.115
Total		100.000

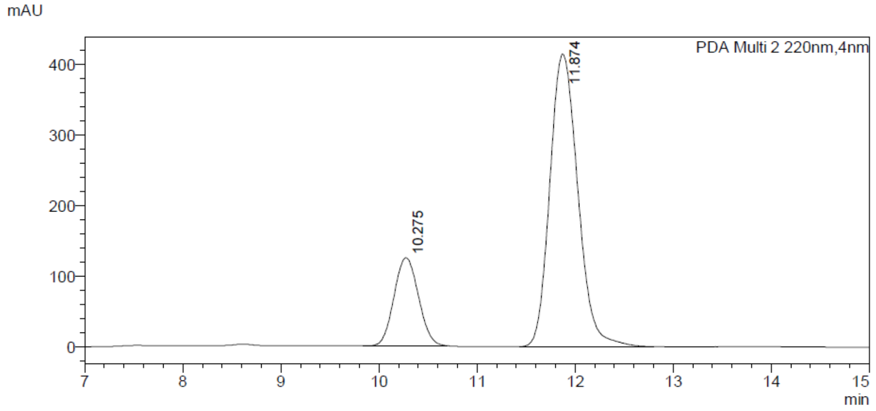

<Peak Table>

PDA Ch2 220nm

Peak#	Ret. Time	Area%
1	10.275	20.762
2	11.874	79.238
Total		100.000

HPLC Data for **S26**: Chiral HPLC analysis Chiralcel OJ-H (99:1 hexane : IPA, flow rate 1.0 mL min^{-1}, 211 nm, 30 °C) t_R (*R*): 7.4 min, t_R (*S*): 12.2 min, 67 % *ee*.

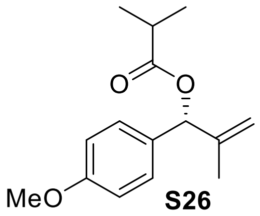

S26

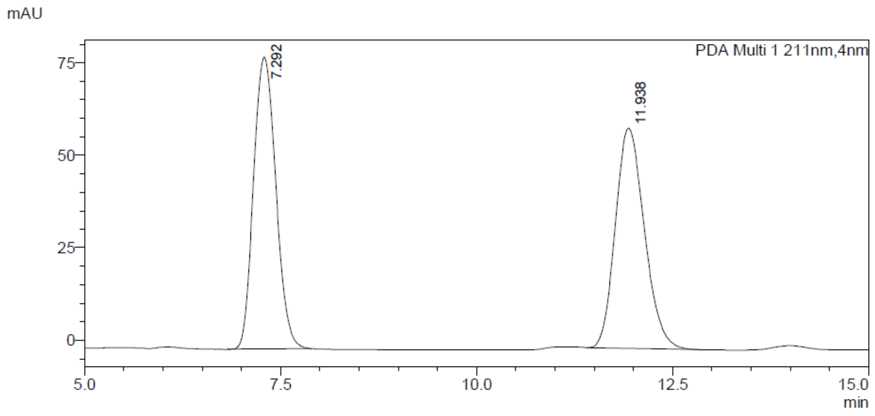

<Peak Table>

PDA Ch1 211nm

Peak#	Ret. Time	Area%
1	7.292	50.419
2	11.938	49.581
Total		100.000

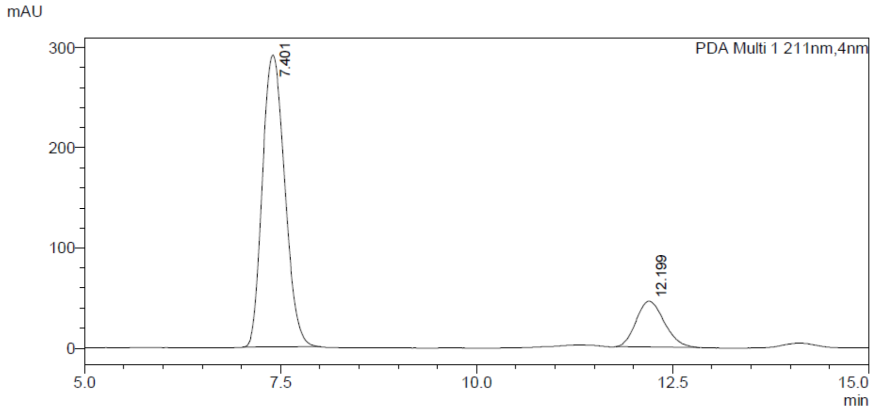

<Peak Table>

PDA Ch1 211nm

Peak#	Ret. Time	Area%
1	7.401	83.501
2	12.199	16.499
Total		100.000

HPLC Data for **42**: Chiral HPLC analysis Chiralcel OD-H (95:5 hexane : IPA, flow rate 1.0 mL min^{-1}, 211 nm, 30 °C) t_R (*R*): 11.2 min, t_R (*S*): 13.6 min, 46% *ee*.

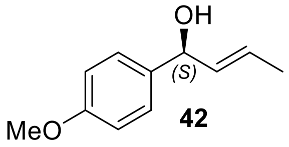

42

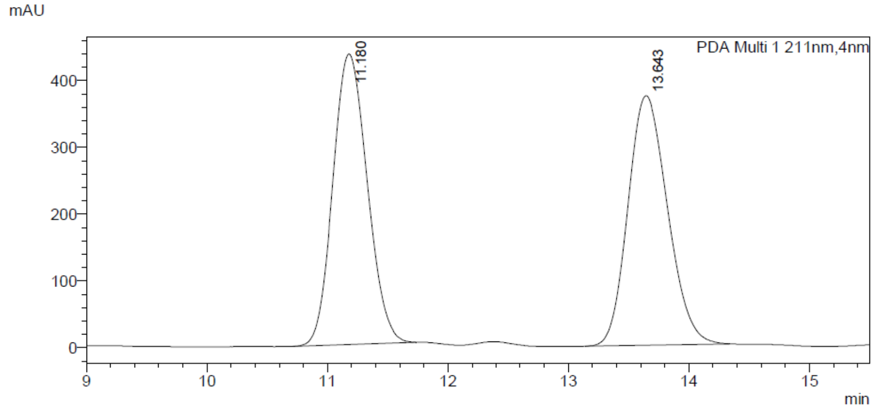

<Peak Table>

PDA Ch1 211nm

Peak#	Ret. Time	Area%
1	11.180	50.188
2	13.643	49.812
Total		100.000

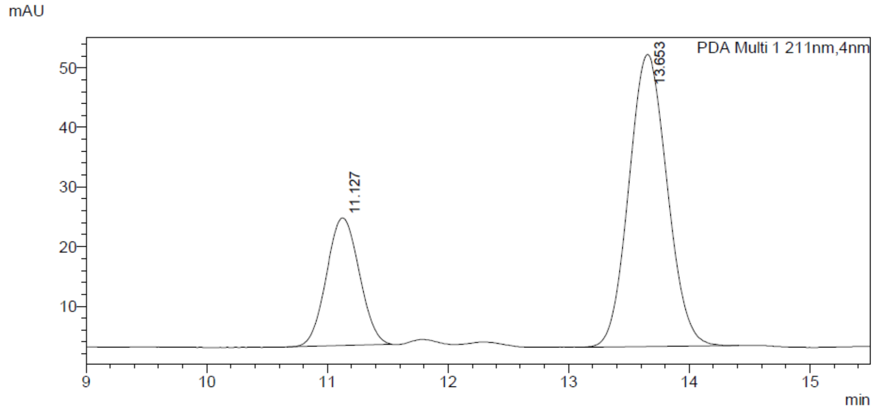

<Peak Table>

PDA Ch1 211nm

Peak#	Ret. Time	Area%
1	11.127	27.252
2	13.653	72.748
Total		100.000

HPLC Data for **S27**: Chiral HPLC analysis Chiralcel OJ-H (99:1 hexane : IPA, flow rate 1.0 mL min^{-1}, 254 nm, 30 °C) t_R (*S*): 18.9 min, t_R (*R*): 21.9 min, 30% *ee*.

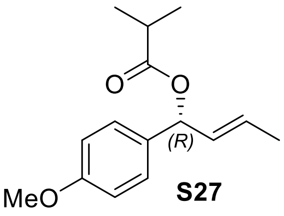

S27

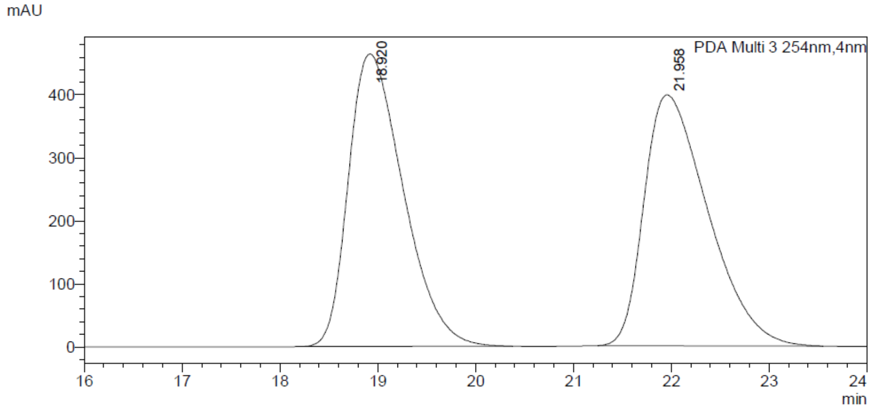

<Peak Table>

PDA Ch3 254nm

Peak#	Ret. Time	Area%
1	18.920	50.079
2	21.958	49.921
Total		100.000

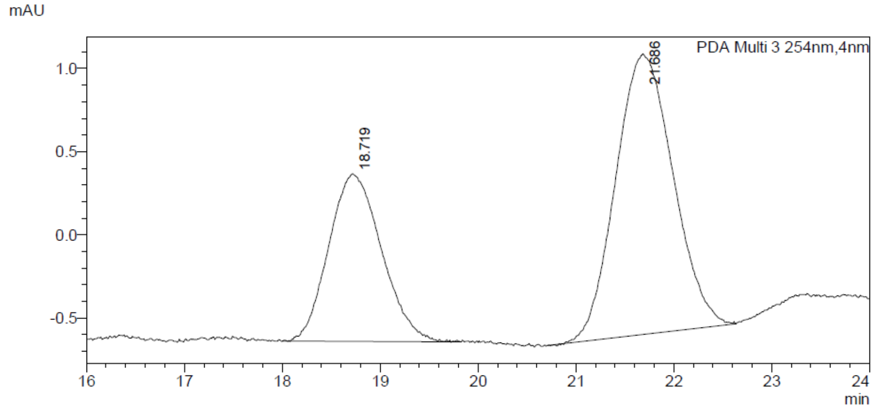

<Peak Table>

PDA Ch3 254nm

Peak#	Ret. Time	Area%
1	18.719	35.393
2	21.686	64.607
Total		100.000

HPLC Data for **43**: Chiral HPLC analysis Chiralcel OD-H (95:5 hexane : IPA, flow rate 1.0 mL min^{-1}, 211 nm, 30 °C) t_R (*R*): 10.6 min, t_R (*S*): 12.6 min, 24% *ee*.

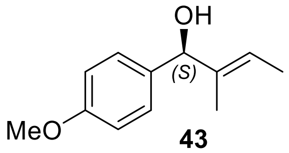

43

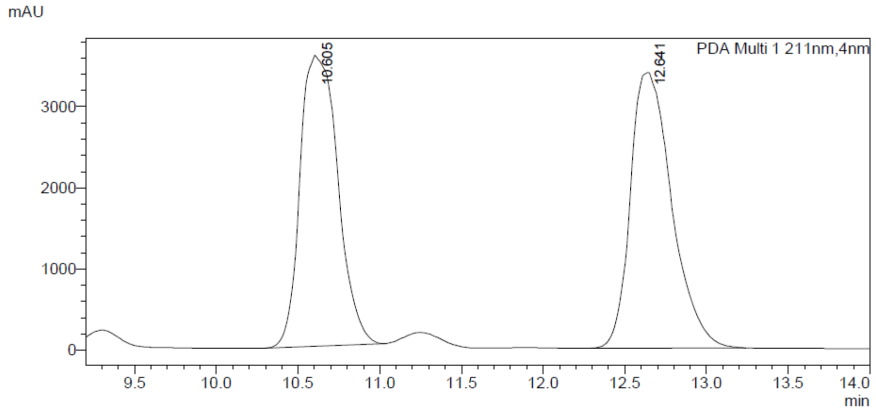

<Peak Table>

PDA Ch1 211nm

Peak#	Ret. Time	Area%
1	10.605	49.047
2	12.641	50.953
Total		100.000

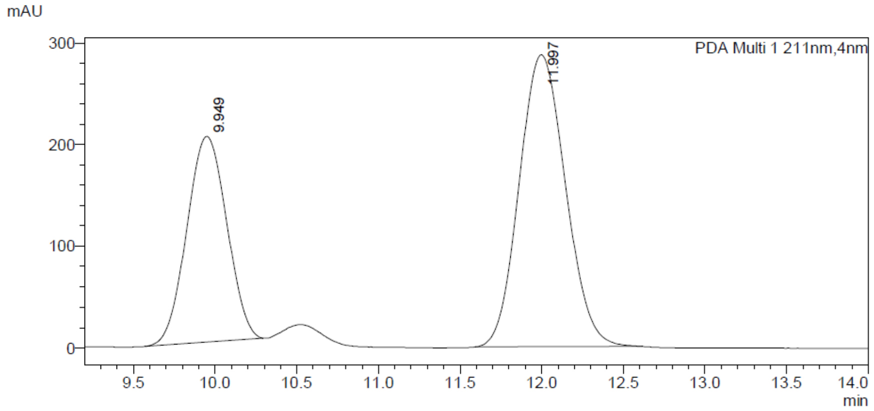

<Peak Table>

PDA Ch1 211nm

Peak#	Ret. Time	Area%
1	9.949	37.841
2	11.997	62.159
Total		100.000

.

HPLC Data for **S28**: Chiral HPLC analysis Chiralcel OJ-H (99:1 hexane : IPA, flow rate 1.0 mL min^{-1}, 220 nm, 30 °C) t_R (*R*): 6.7 min, t_R (*S*): 8.5 min, 40 % *ee*.

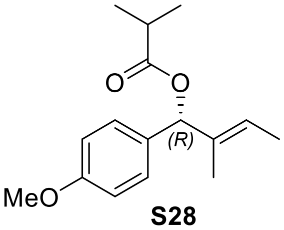

S28

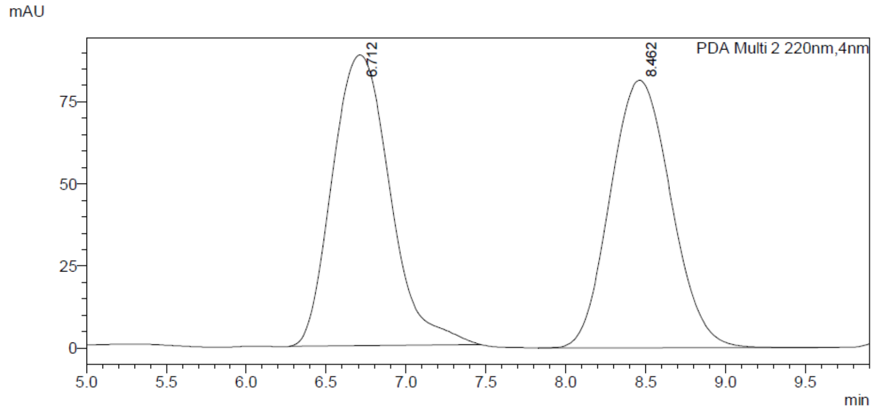

<Peak Table>

PDA Ch2 220nm

Peak#	Ret. Time	Area%
1	6.712	50.797
2	8.462	49.203
Total		100.000

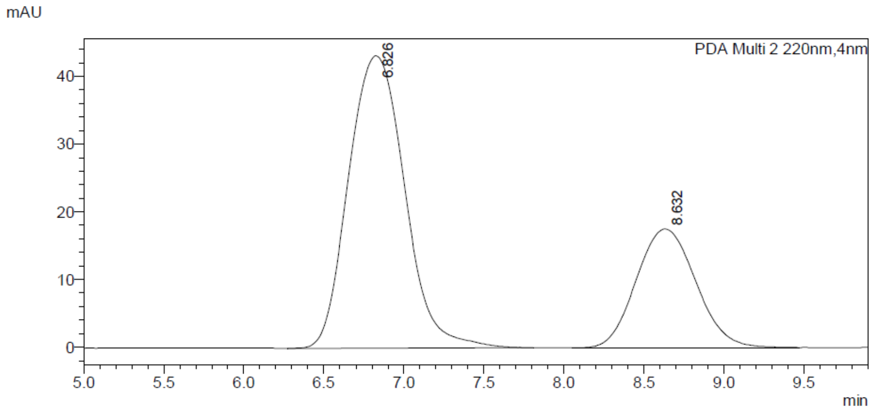

<Peak Table>

PDA Ch2 220nm

Peak#	Ret. Time	Area%
1	6.826	69.937
2	8.632	30.063
Total		100.000

HPLC Data for **44**: Chiral HPLC analysis Chiralcel OJ-H (80:20 hexane : IPA, flow rate 1.0 mL min^{-1}, 270 nm, 30 °C) t_R (*S*): 9.9 min, t_R (*R*): 10.9 min, 73% *ee*.

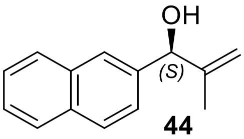

44

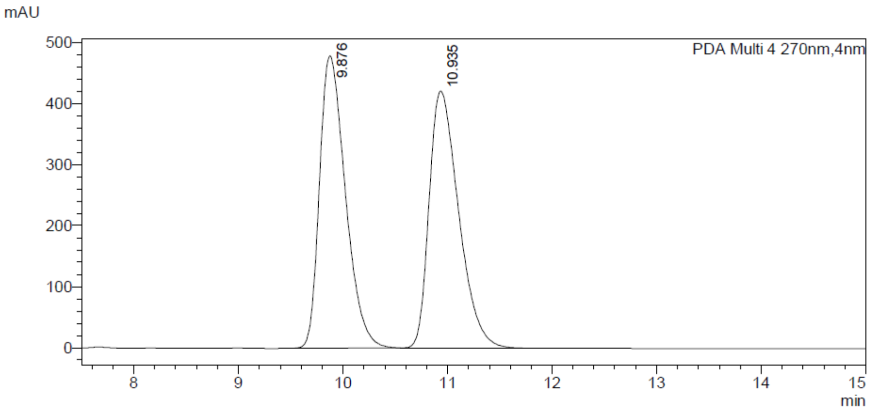

<Peak Table>

PDA Ch4 270nm

Peak#	Ret. Time	Area%
1	9.876	49.997
2	10.935	50.003
Total		100.000

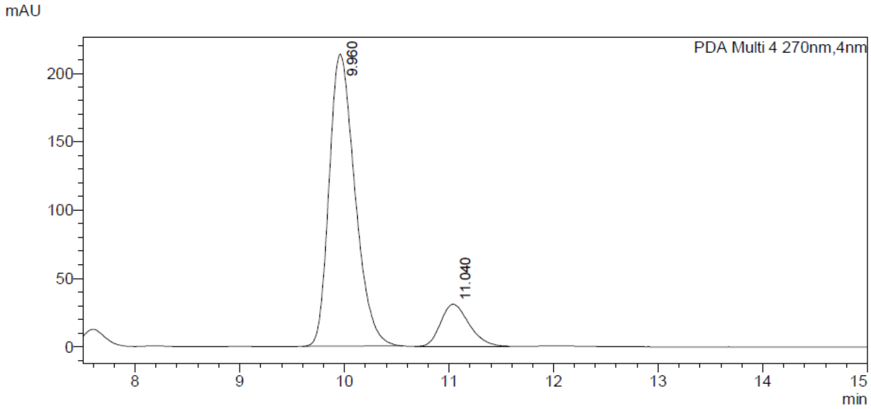

<Peak Table>

PDA Ch4 270nm

Peak#	Ret. Time	Area%
1	9.960	86.401
2	11.040	13.599
Total		100.000

HPLC Data for **S29**: Chiral HPLC analysis Chiralcel OJ-H (80:20 hexane : IPA, flow rate 1.0 mL min^{-1}, 211 nm, 30 °C) t_R (*R*): 5.1 min, t_R (*S*): 7.0 min, 83 % *ee*.

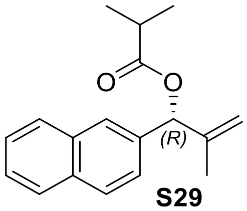

S29

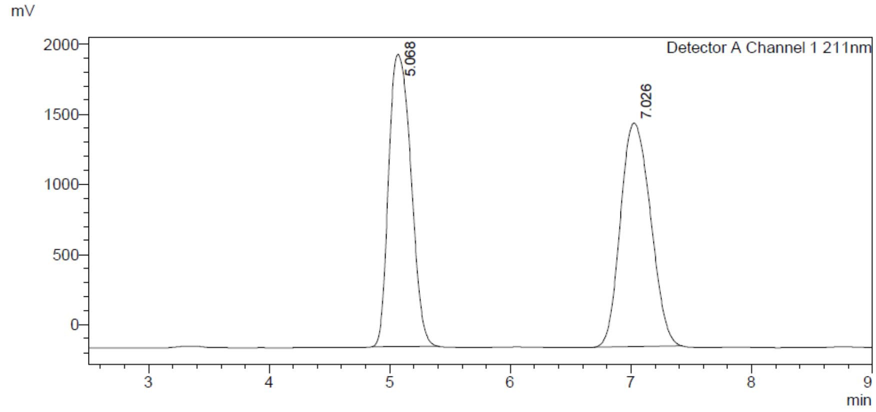

<Peak Table>

Detector A Channel 1 211nm

Peak#	Ret. Time	Area%
1	5.068	49.168
2	7.026	50.832
Total		100.000

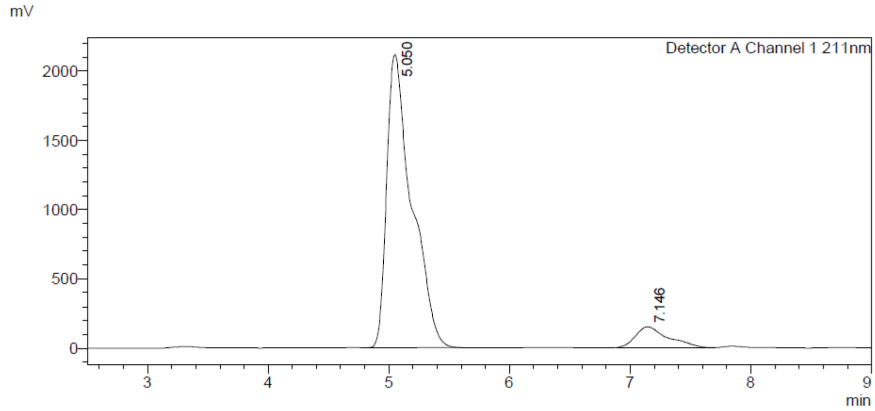

<Peak Table>

Detector A Channel 1 211nm

Peak#	Ret. Time	Area%
1	5.050	91.646
2	7.146	8.354
Total		100.000

HPLC Data for **45**: Chiral HPLC analysis Chiralcel OJ-H (80:20 hexane : IPA, flow rate 1.0 mL min^{-1}, 211 nm, 30 °C) t_R (*S*): 8.2 min, t_R (*R*): 10.2 min, 68% *ee*.

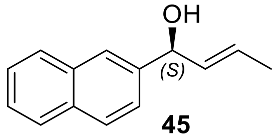

45

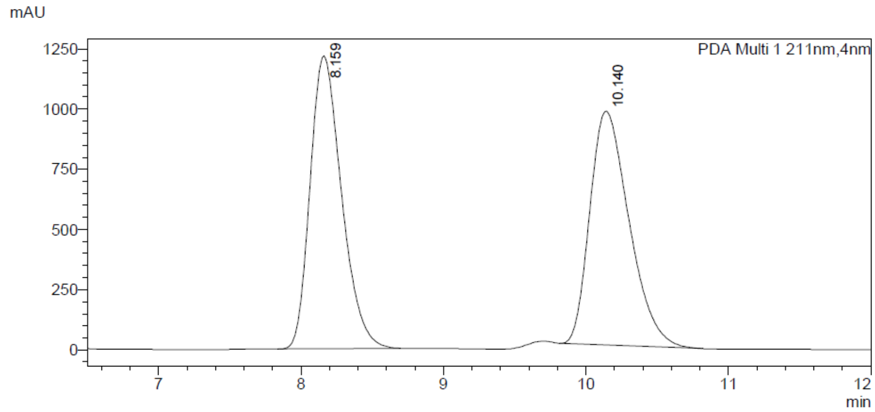

<Peak Table>

PDA Ch1 211nm

Peak#	Ret. Time	Area%
1	8.159	50.068
2	10.140	49.932
Total		100.000

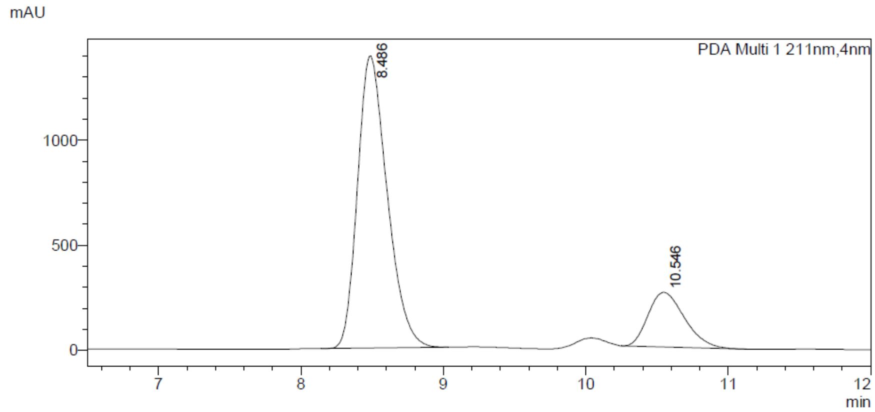

<Peak Table>

PDA Ch1 211nm

Peak#	Ret. Time	Area%
1	8.486	81.315
2	10.546	18.685
Total		100.000

HPLC Data for **S30**: Chiral HPLC analysis Chiralcel OJ-H (95:5 hexane : IPA, flow rate 0.5 mL min^{-1}, 211 nm, 30 °C) t_R (*R*): 11.0 min, t_R (*S*): 12.8 min, 60% *ee*.

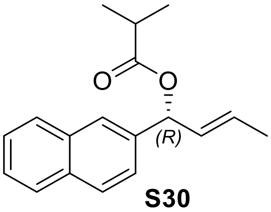

S30

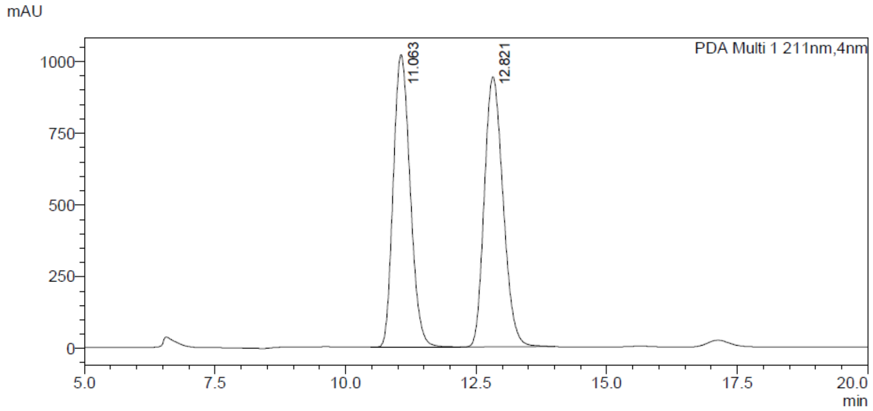

<Peak Table>

PDA Ch1 211nm

Peak#	Ret. Time	Area%
1	11.063	49.261
2	12.821	50.739
Total		100.000

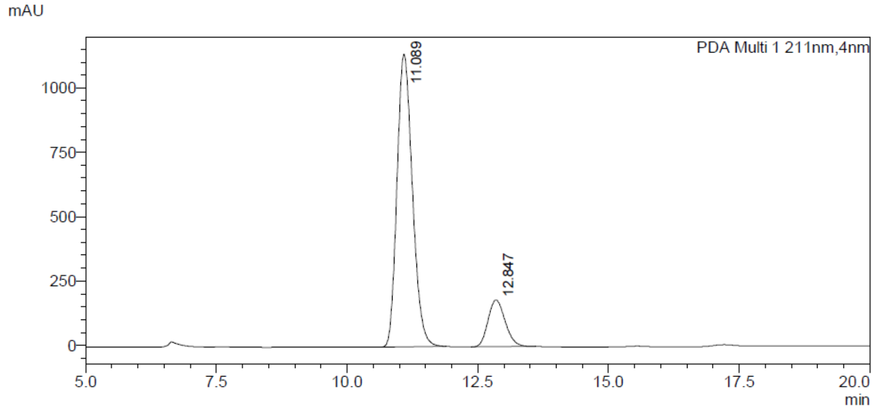

<Peak Table>

PDA Ch1 211nm

Peak#	Ret. Time	Area%
1	11.089	84.895
2	12.847	15.105
Total		100.000

HPLC Data for **46**: Chiral HPLC analysis Chiralcel OJ-H (80:20 hexane : IPA, flow rate 1.0 mL min^{-1}, 211 nm, 30 °C) t_R (*S*): 7.6 min, t_R (*R*): 8.4 min, 68% *ee*.

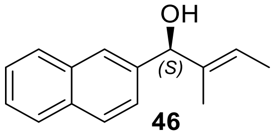

46

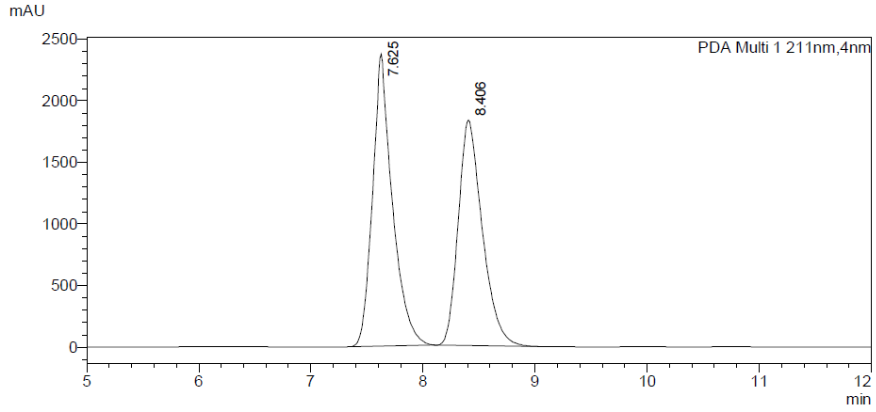

<Peak Table>

PDA Ch1 211nm

Peak#	Ret. Time	Area%
1	7.625	51.532
2	8.406	48.468
Total		100.000

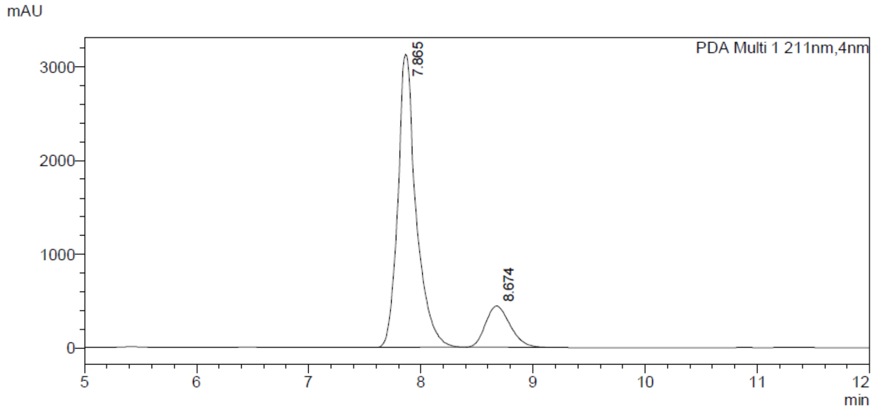

<Peak Table>

PDA Ch1 211nm

Peak#	Ret. Time	Area%
1	7.865	84.094
2	8.674	15.906
Total		100.000

HPLC Data for **S31**: Chiral HPLC analysis Chiralcel OJ-H (95:5 hexane : IPA, flow rate 0.5 mL min^{-1}, 211 nm, 30 °C) t_R (*R*): 11.0 min, t_R (*S*): 14.9 min, 60% *ee*.

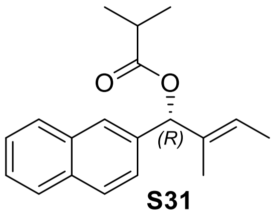

S31

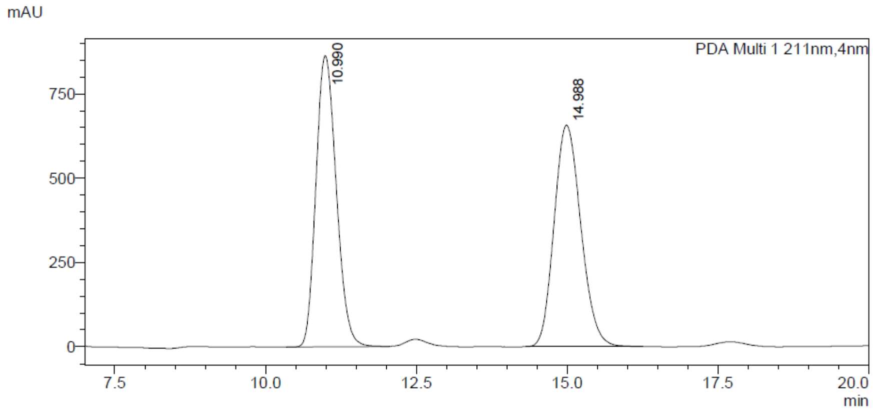

<Peak Table>

PDA Ch1 211nm

Peak#	Ret. Time	Area%
1	10.990	49.961
2	14.988	50.039
Total		100.000

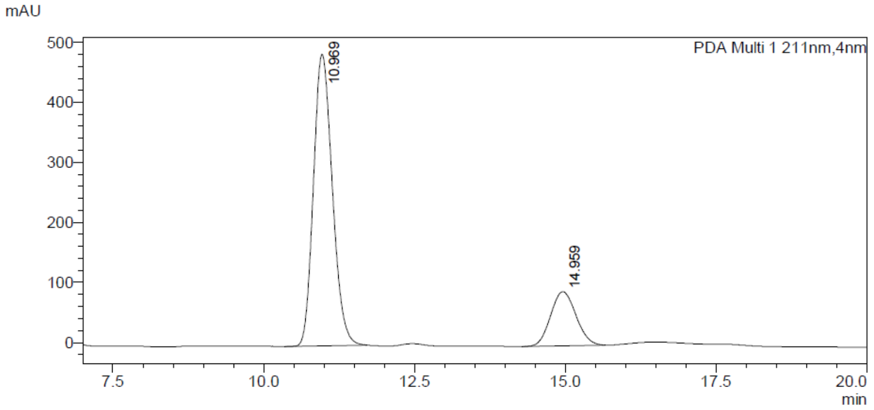

<Peak Table>

PDA Ch1 211nm

Peak#	Ret. Time	Area%
1	10.969	80.085
2	14.959	19.915
Total		100.000

HPLC Data for **47**: Chiral HPLC analysis Chiralcel OJ-H (80:20 hexane : IPA, flow rate 1.0 mL min^{-1}, 254 nm, 30 °C) t_R (*S*): 8.7 min, t_R (*R*): 11.3 min, >99% *ee*.

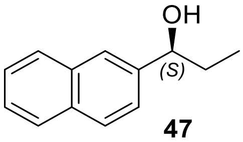

47

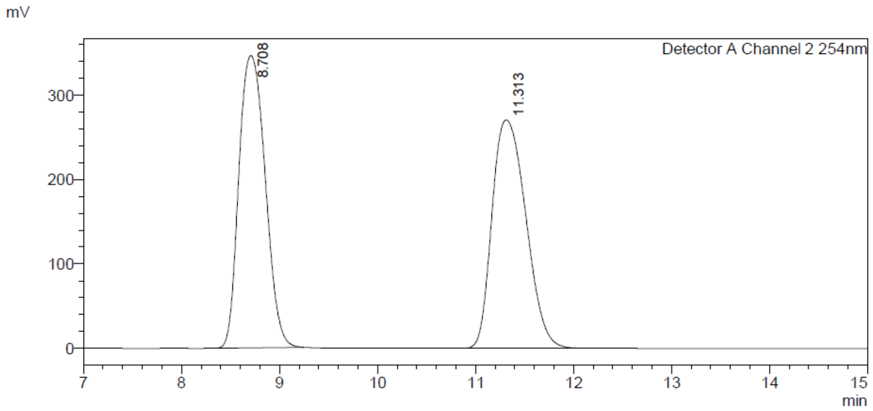

<Peak Table>

Detector A Channel 2 254nm

Peak#	Ret. Time	Area%
1	8.708	49.926
2	11.313	50.074
Total		100.000

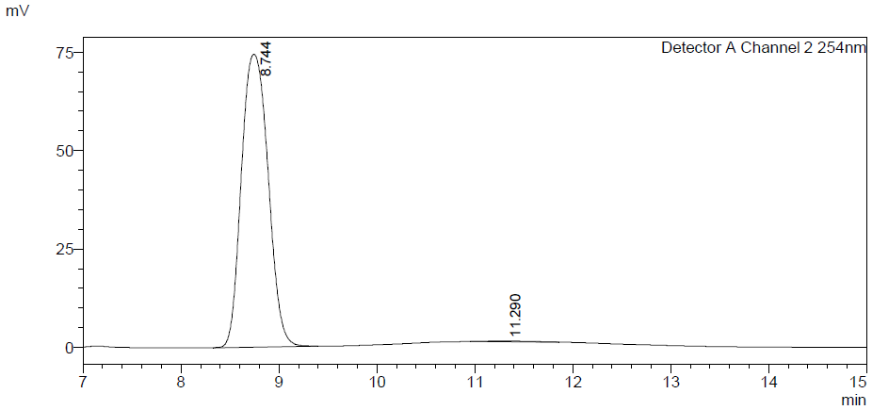

<Peak Table>

Detector A Channel 2 254nm

Peak#	Ret. Time	Area%
1	8.744	99.618
2	11.290	0.382
Total		100.000

HPLC Data for **S32**: Chiral HPLC analysis Chiralcel OJ-H (90:10 hexane : IPA, flow rate 0.8 mL min^{-1}, 220 nm, 30 °C) t_R (*R*): 7.2 min, t_R (*S*): 9.3 min, 93 % *ee*.

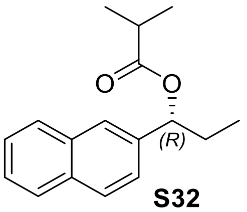

S32

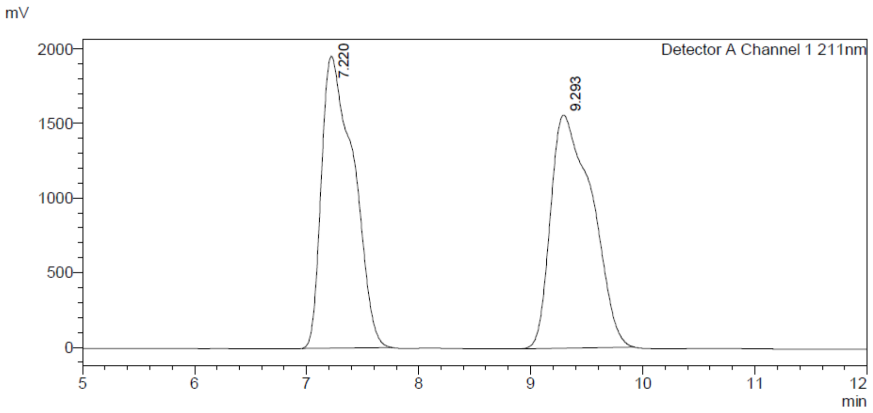

<Peak Table>

Detector A Channel 1 211nm

Peak#	Ret. Time	Area%
1	7.220	49.202
2	9.293	50.798
Total		100.000

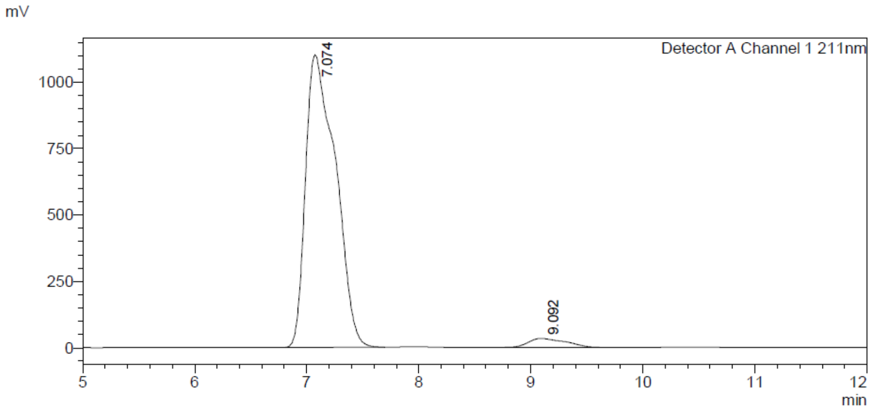

<Peak Table>

Detector A Channel 1 211nm

Peak#	Ret. Time	Area%
1	7.074	96.298
2	9.092	3.702
Total		100.000

HPLC Data for **48**: Chiral HPLC analysis Chiralcel OJ-H (80:20 hexane : IPA, flow rate 1.0 mL min^{-1}, 211 nm, 30 °C) t_R (*S*): 22.1 min, t_R (*R*): 25.5 min, 32% *ee*.

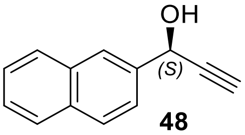

48

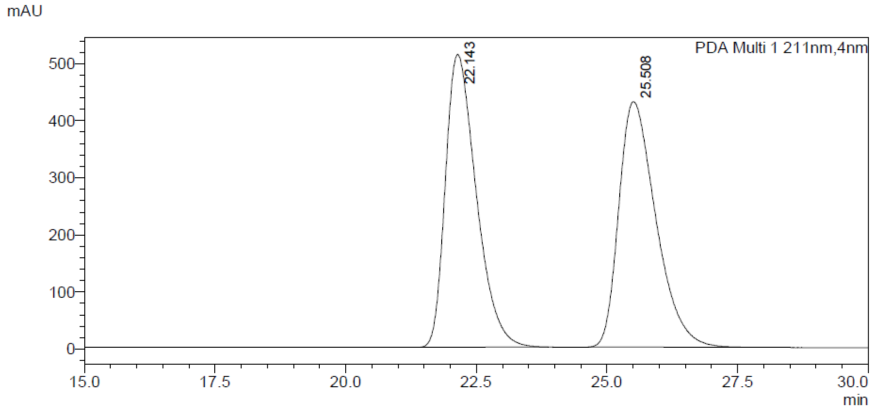

<Peak Table>

PDA Ch1 211nm

Peak#	Ret. Time	Area%
1	22.143	49.839
2	25.508	50.161
Total		100.000

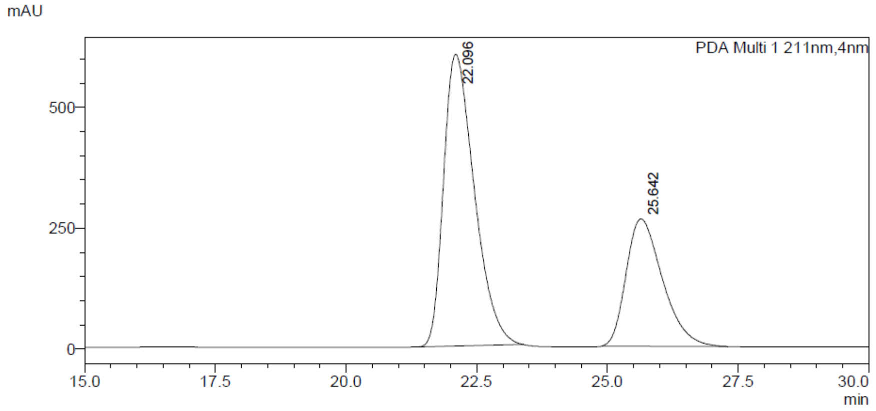

<Peak Table>

PDA Ch1 211nm

Peak#	Ret. Time	Area%
1	22.096	66.066
2	25.642	33.934
Total		100.000

HPLC Data for **S33**: Chiral HPLC analysis Chiralcel OJ-H (90:10 hexane : IPA, flow rate 1.0 mL min^{-1}, 270 nm, 30 °C) t_R (*R*): 21.4 min, t_R (*S*): 23.4 min, 28% *ee*.

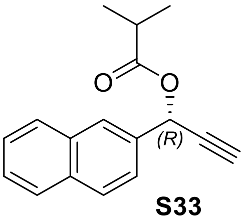

S33

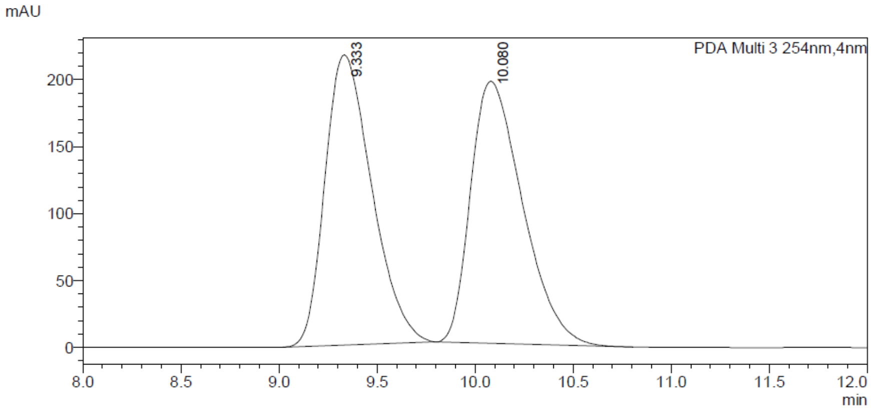

<Peak Table>

PDA Ch3 254nm

Peak#	Ret. Time	Area%
1	9.333	49.958
2	10.080	50.042
Total		100.000

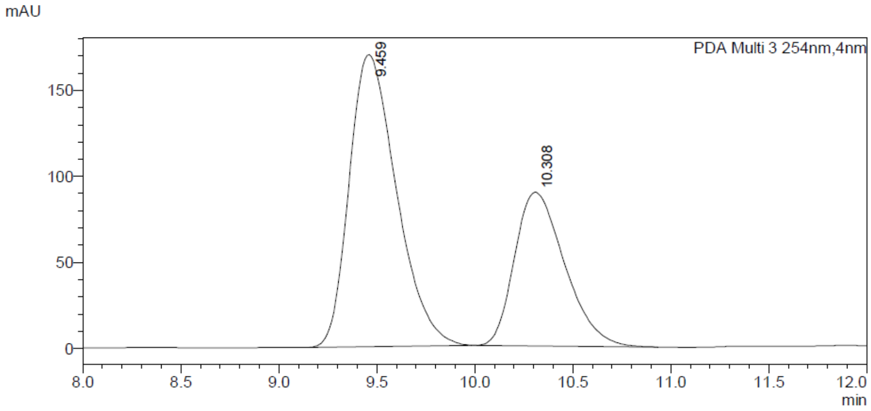

<Peak Table>

PDA Ch3 254nm

Peak#	Ret. Time	Area%
1	9.459	64.144
2	10.308	35.856
Total		100.000

HPLC Data for **49**: Chiral HPLC analysis Chiralcel OD-H (88:12 hexane : IPA, flow rate 1.0 mL min^{-1}, 211 nm, 35 °C) t_R (*S*): 6.2 min, t_R (*R*): 7.6 min, 21% *ee*.

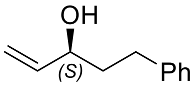

49

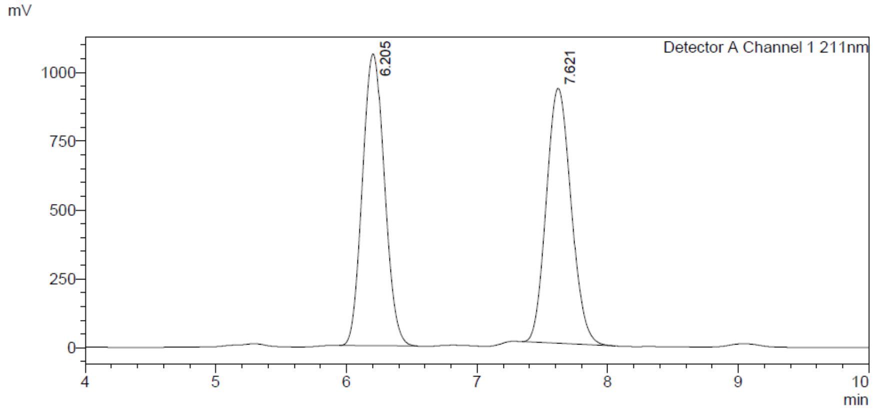

<Peak Table>

Detector A Channel 1 211nm

Peak#	Ret. Time	Area%
1	6.205	50.223
2	7.621	49.777
Total		100.000

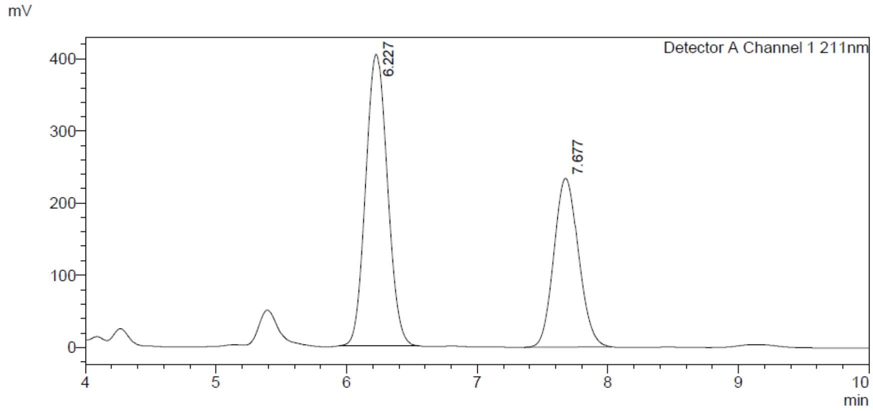

<Peak Table>

Detector A Channel 1 211nm

Peak#	Ret. Time	Area%
1	6.227	60.439
2	7.677	39.561
Total		100.000

HPLC Data for **S34**: Chiral HPLC analysis Chiralcel OJ-H (99:1 hexane : IPA, flow rate 1.0 mL min^{-1}, 220 nm, 30 °C) t_R (*S*): 6.2 min, t_R (*R*): 7.1 min, 36% *ee*.

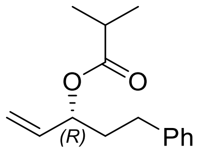

S34

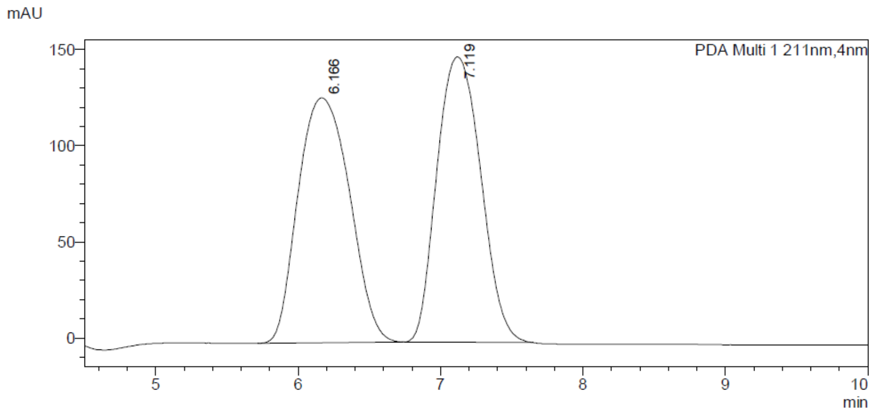

<Peak Table>

PDA Ch1 211nm

Peak#	Ret. Time	Area%
1	6.166	50.122
2	7.119	49.878
Total		100.000

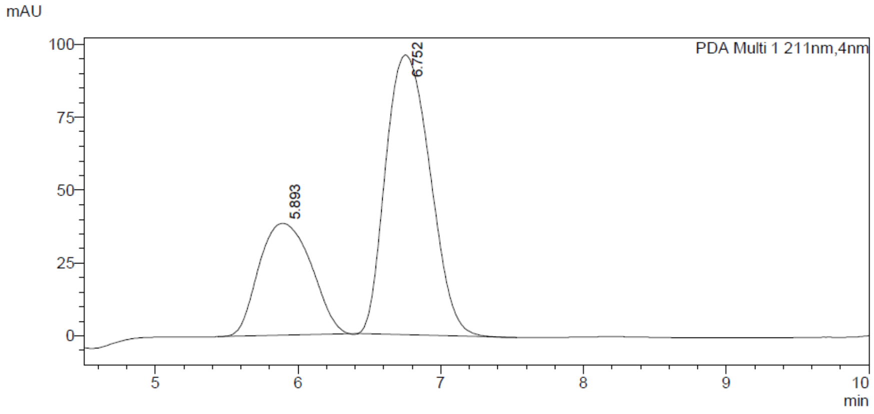

<Peak Table>

PDA Ch1 211nm

Peak#	Ret. Time	Area%
1	5.893	31.975
2	6.752	68.025
Total		100.000

Preparative Kinetic Resolution:

HPLC Data for **32**: Chiral HPLC analysis Chiralcel OJ-H (80:20 hexane : IPA, flow rate 1.0 mL min^{-1}, 254 nm, 30 °C) t_R (*S*): 11.7 min, t_R (*R*): 14.1 min, 98% *ee*.

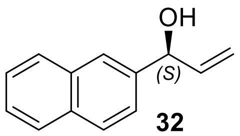

32

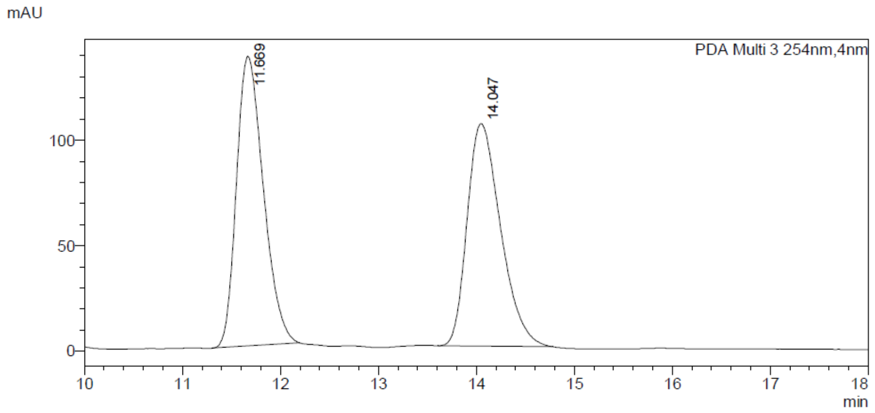

<Peak Table>

PDA Ch3 254nm

Peak#	Ret. Time	Area%
1	11.669	51.168
2	14.047	48.832
Total		100.000

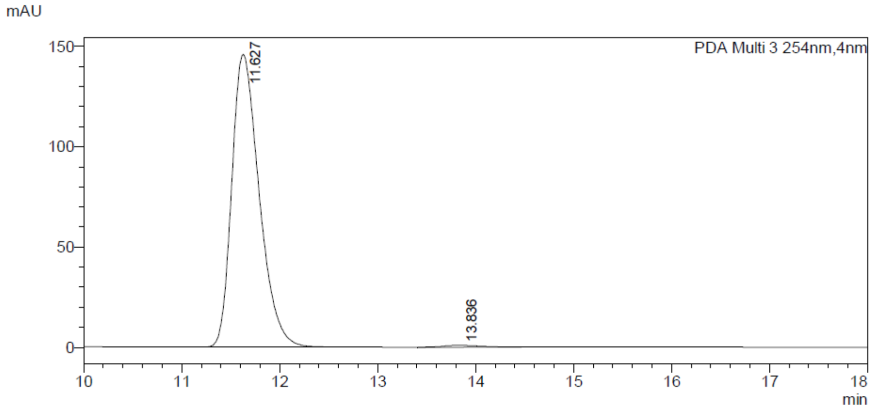

<Peak Table>

PDA Ch3 254nm

Peak#	Ret. Time	Area%
1	11.627	99.145
2	13.836	0.855
Total		100.000

HPLC Data for **37**: Chiral HPLC analysis Chiralcel OJ-H (95:5 hexane : IPA, flow rate 0.5 mL min^{-1}, 270 nm, 30 °C) t_R (*S*): 13.4 min, t_R (*R*): 15.8 min, >99 % *ee*.

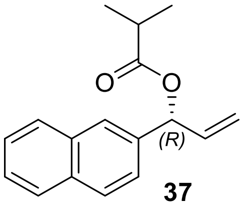

37

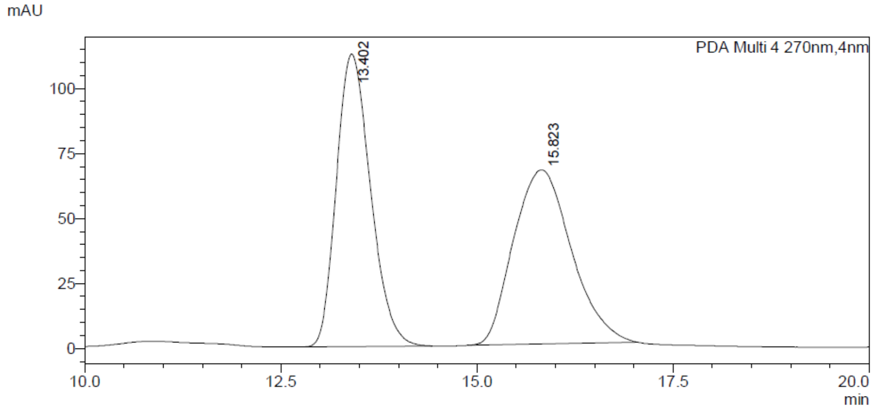

<Peak Table>

PDA Ch4 270nm

Peak#	Ret. Time	Area%
1	13.402	49.722
2	15.823	50.278
Total		100.000

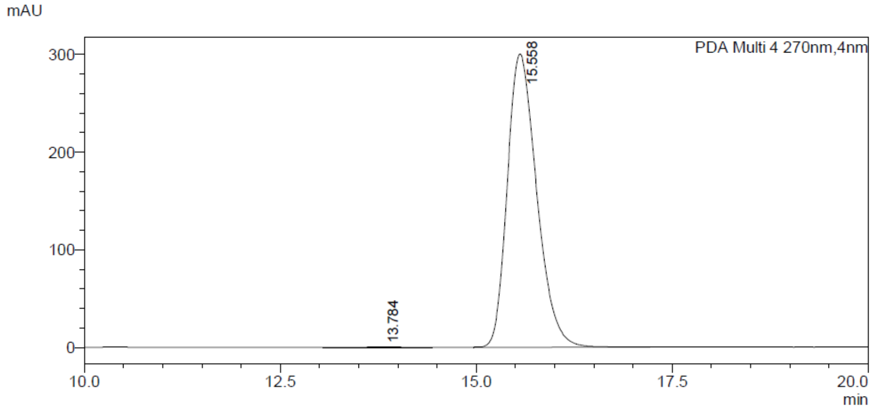

<Peak Table>

PDA Ch4 270nm

Peak#	Ret. Time	Area%
1	13.784	0.063
2	15.558	99.937
Total		100.000
